# Measuring contraceptive method mix, prevalence, and demand satisfied by age and marital status in 204 countries and territories, 1970–2019: a systematic analysis for the Global Burden of Disease Study 2019

**DOI:** 10.1016/S0140-6736(22)00936-9

**Published:** 2022-07-23

**Authors:** Annie Haakenstad, Olivia Angelino, Caleb M S Irvine, Zulfiqar A Bhutta, Kelly Bienhoff, Corinne Bintz, Kate Causey, M Ashworth Dirac, Nancy Fullman, Emmanuela Gakidou, Thomas Glucksman, Simon I Hay, Nathaniel J Henry, Ira Martopullo, Ali H Mokdad, John Everett Mumford, Stephen S Lim, Christopher J L Murray, Rafael Lozano

**Affiliations:** a Institute for Health Metrics and Evaluation, University of Washington, Seattle, WA, USA; b Department of Health Metrics Sciences, University of Washington, Seattle, WA, USA; c Centre for Global Child Health, University of Toronto, Toronto, ON, Canada; d Centre of Excellence in Women and Child Health, Aga Khan University, Karachi, Pakistan; e Swedish Family Medicine, First Hill, Seattle, WA, USA

## Abstract

**Background:**

Meeting the contraceptive needs of women of reproductive age is beneficial for the health of women and children, and the economic and social empowerment of women. Higher rates of contraceptive coverage have been linked to the availability of a more diverse range of contraceptive methods. We present estimates of the contraceptive prevalence rate (CPR), modern contraceptive prevalence rate (mCPR), demand satisfied, and the method of contraception used for both partnered and unpartnered women for 5-year age groups in 204 countries and territories between 1970 and 2019.

**Methods:**

We used 1162 population-based surveys capturing contraceptive use among women between 1970 and 2019, in which women of reproductive age (15–49 years) self-reported their, or their partner's, current use of contraception for family planning purposes. Spatiotemporal Gaussian process regression was used to generate estimates of the CPR, mCPR, demand satisfied, and method mix by age and marital status. We assessed how age-specific mCPR and demand satisfied changed with the Socio-demographic Index (SDI), a measure of social and economic development, using the meta-regression Bayesian, regularised, trimmed method from the Global Burden of Diseases, Injuries, and Risk Factors Study.

**Findings:**

In 2019, 162·9 million (95% uncertainty interval [UI] 155·6–170·2) women had unmet need for contraception, of whom 29·3% (27·9–30·6) resided in sub-Saharan Africa and 27·2% (24·4–30·3) resided in south Asia. Women aged 15–19 years (64·8% [62·9–66·7]) and 20–24 years (71·9% [68·9–74·2]) had the lowest rates of demand satisfied, with 43·2 million (95% UI 39·3–48·0) women aged 15–24 years with unmet need in 2019. The mCPR and demand satisfied among women aged 15–19 years were substantially lower than among women aged 20–49 years at SDI values below 60 (on a 0–100 scale), but began to equalise as SDI increased above 60. Between 1970 and 2019, the global mCPR increased by 20·1 percentage points (95% UI 18·7–21·6). During this time, traditional methods declined as a proportion of all contraceptive methods, whereas the use of implants, injections, female sterilisation, and condoms increased. Method mix differs substantially depending on age and geography, with the share of female sterilisation increasing with age and comprising more than 50% of methods in use in south Asia. In 28 countries, one method was used by more than 50% of users in 2019.

**Interpretation:**

The dominance of one contraceptive method in some locations raises the question of whether family planning policies should aim to expand method mix or invest in making existing methods more accessible. Lower rates of demand satisfied among women aged 15–24 years are also concerning because unintended pregnancies before age 25 years can forestall or eliminate education and employment opportunities that lead to social and economic empowerment. Policy makers should strive to tailor family planning programmes to the preferences of the groups with the most need, while maintaining the programmes used by existing users.

**Funding:**

Bill & Melinda Gates Foundation.

## Introduction

Pregnancy and childbirth resulted in nearly 200 000 deaths among women of reproductive age (15–49 years) in 2019.^1^ Use of contraception is associated with reductions in maternal mortality and neonatal mortality due to the avoidance of unintended and adolescent pregnancies, and contributes to spacing, timing, and limiting births.^2^ Access to contraception also empowers women and supports the pursuit of gender equity.^3^ Access to contraception has been shown to increase paid employment among women and generate other social and economic benefits,^4^, ^5^, ^6^, ^7^, ^8^, ^9^, ^10^, ^11^, ^12^ partly because delaying childbearing to later in life allows women to pursue education and gain work experience.^13^ Access to modern contraception is also a strategy to reduce costs for health systems and households by decreasing expenditure on health care associated with maternal and child health.^14^

**Research in context**

**Evidence before this study**

Family planning indicators have been estimated by the United Nations Department of Economic and Social Affairs (UN-DESA), the Guttmacher Institute, Avenir Health, and the UN Population Fund. Substantial increases in the modern contraceptive prevalence rate (mCPR) and demand satisfied have been reported since 1980. Less is known about how the types of methods in use differ across geographies, marital status, and age, which is essential for identifying groups with unmet need and developing policies to address demand. We searched PubMed from database inception to Oct 15, 2021, for peer-reviewed studies using the search term “contraceptive method mix”. We found seven studies that assessed contraceptive method mix across more than 50 countries. Existing studies examined method mix to a limited extent over time, by age or marital status, and disaggregated method mix by sector (eg, public *vs* private facilities) and household characteristics (education, wealth quintile, and rural or urban residence). A data brief by UN-DESA reported method mix by region, marital status, and for two timepoints (1994 and 2019), but omitted age. No existing studies have assessed contraceptive method mix by age and marital status simultaneously and systematically for 204 countries and territories or tracked changes between 1970 and 2019 continuously.

**Added value of this study**

This study estimated, for the first time, the types of contraceptive methods used among women of reproductive age (15–49 years) disaggregated by 5-year age groups and marital status between 1970 and 2019, as part of a systematic analysis for all 204 countries and territories included in the Global Burden of Disease Study 2019. We estimated the contraceptive prevalence rate (CPR), mCPR, contraceptive method mix, and Sustainable Development Goal indicator 3.7.1 (need for family planning met with modern methods, or demand satisfied). Relative to existing studies, we used flexible methods suited to estimation across locations and time: spatiotemporal Gaussian process regression produces estimates that follow data closely where they exist and, where data are unavailable, extrapolate between datapoints with covariates and incorporate information from locations and years close in proximity. Estimates of family planning indicators enabled assessment of how method mix and contraceptive coverage have changed over time for groups with distinct needs. Our study also examined the extent to which family planning indicators are connected to social and economic development.

**Implications of all the available evidence**

Since 1970, the use of contraception has increased substantially worldwide, underpinned by a shift from traditional methods to more effective modern methods. Despite this increase, more than 160 million women have a need for contraception that is not currently met by existing family planning programmes. More than 40 million of women with unmet need were aged 15–24 years, which represents a crucial period of life during which educational attainment and training often occur. Countries with low demand satisfied in this age group might fail to benefit from the social and economic benefits and empowerment of women enabled by contraception. To address this demand, family planning programmes must determine whether existing methods are equally preferred and accessible to younger women in comparison to older women. Globally, younger women are more likely to use condoms and the oral contraceptive pill, whereas older women are more likely to use female sterilisation. Using location-specific estimates of method mix by age and marital status from this study, decision makers should consider whether expanding method choice or improving access to the existing methods offered would be more effective in reaching the groups with the most unmet need, including current users who might have unmet need in the future.

Expanding access and use of contraception is a key commitment in a number of international initiatives, including the Millennium Development Goals, Every Woman Every Child, Family Planning 2020 (FP2020), and the 2030 Sustainable Development Goals (SDGs). Millennium Development Goal 5.B focused on universal access to reproductive health, relying on measures of the contraceptive prevalence rate (CPR), unmet need for family planning, and other indicators.^15^ FP2020 committed to increasing the number of additional users of modern contraception by 120 million between 2012 and 2020 in 69 countries.^16^ SDG indicator 3.7.1 focuses on the proportion of women of reproductive age whose need for family planning is satisfied with modern methods and aims for universal access.^17^ In support of these initiatives, an estimated US$1·1 billion is disbursed annually as development assistance for family planning in addition to the $1·7 billion invested in family planning by governments in low-income and middle-income countries.^18^

Robust tracking of use, trends, and contraceptive method mix is crucial to attaining aspirations for family planning and realising the benefits of contraception for women's health and empowerment. The Population Division of the United Nations Department of Economic and Social Affairs (UN-DESA) has been a key source for family planning indicators,^19^, ^20^, ^21^, ^22^ with estimates showing that major increases in use and reductions in unmet need have occurred worldwide since 1980.^23^, ^24^, ^25^, ^26^, ^27^ An analysis by Avenir Health of FP2020 countries showed the mCPR exceeded the trend expected based on trajectories before the launch of the initiative.^28^ Additional studies by the Guttmacher Institute, the UN Population Fund, and other authors groups have also retrospectively estimated and forecasted family planning indicators.^29^, ^30^, ^31^, ^32^, ^33^, ^34^, ^35^, ^36^, ^37^

However, key gaps remain in the understanding of global trends of contraceptive use and need for contraception. Existing analyses of contraception coverage and method mix over time and across countries have not examined indicators by location, age, and marital status simultaneously or assessed changes over time continuously. Analyses by UN-DESA assessed contraceptive method mix by region, marital status, and for two timepoints (1994 and 2019), but omitted age.^38^ Other studies have assessed method mix by select combinations of time, region, age, and marital status,^39^ but have not assessed all factors simultaneously. Analyses of method mix have documented the dominance of certain contraceptive methods in some countries;^40^ how modern contraceptive prevalence (mCPR) increases as method mix expands;^41^ and disaggregated method mix by sector (public *vs* private) and household characteristics (rural or urban residence, wealth quintile, and education).^42^ To date, no studies have examined method mix by age and marital status simultaneously and systematically for 204 countries and territories and continuously between 1970 and 2019. Such estimates are crucial to understanding how use of contraception differs by age and marital status, and how changes in method mix over time are connected to the realisation of family planning goals.

Our study therefore extends the existing evidence base with regard to the types of contraception used in the past 50 years according to the marital status and age among women of reproductive age. We estimated the CPR, mCPR, demand satisfied, and the method of contraception used for both partnered and unpartnered women for 5-year age groups in 204 countries and territories between 1970 and 2019. Updated and comprehensive information on contraception use provides decision makers with a comprehensive view of changes in contraceptive use over time and supports the identification of the policies required for improving access and reducing unmet need.

## Methods

### Overview and data sources

This analysis complies with the Guidelines for Accurate and Transparent Health Estimates Reporting statement (appendix p 7).^43^

We used microdata and tabulated reports from 1162 nationally or subnationally representative surveys done between 1970 and 2019, in which women aged 15–49 years self-reported their, or their partner's, current use of contraception for family planning purposes. We obtained data from the Demographic and Health Surveys, Multiple Indicator Cluster Surveys, Performance Monitoring for Action surveys, Generations and Gender Programme surveys, World Fertility Surveys, Reproductive Health Surveys, Contraceptive Prevalence Surveys, and Pan Arab Project for Child Development Family Health surveys, in addition to other country-specific family planning surveys. More information about all surveys used and their sampling strategies and weighting approaches (made available from the data collection agencies) is available on the Global Health Data Exchange GBD 2019 website. We excluded sources that did not contain self-reported data or only interviewed male partners, only asked about single methods such as condoms, exclusively sampled girls in schools, or were otherwise not representative at the national or GBD-defined subnational level (eg, health facility exit interviews). Surveys that only sampled ever-partnered women were restricted to currently partnered women for use in our estimates of partnered estimates, when possible, or were otherwise excluded. To generate nationally representative estimates and SEs from the microdata, we applied survey weights provided by the respective data collection institutions. Additional information on the surveys used and data processing is included in the appendix (pp 12–18).

### Definitions of family planning indicators

Partnered women refers to women who self-identify as legally or formally married, living in a union, or otherwise partnered. Unpartnered women (often referred to as unmarried) included women who have never been married or are separated, divorced, or widowed. We modelled partnered women and unpartnered women separately because some surveys only captured partnered women and because trends and levels of contraception indicators tend to differ between the two groups. Need for contraception was also defined differently for partnered and unpartnered women.

The contraceptive prevalence rate (CPR) was defined as the proportion of all women of reproductive age (15–49 years) who were currently using, or whose partner is using, at least one method of contraception, and the mCPR was defined as the proportion of all women of reproductive age using modern contraceptive methods. Modern methods of contraception include male or female sterilisation (ie, vasectomy or tubal ligation), oral contraceptive pills, male or female condoms, diaphragms, spermicides and sponges, hormonal or non-hormonal intrauterine devices, implants, injections, contraceptive patches and rings, and emergency contraceptives. All other family planning methods were considered traditional, including the lactational amenorrhoea method, withdrawal, calendar methods (rhythm or standard days), douches, periodic abstinence, and other methods. Some methods are rarely or never used in some settings, however, we estimated all methods for all settings for completeness and because in some locations across the world specific methods are more commonly used. The CPR and mCPR do not consider the needs of women, which are fundamental for determining gaps in coverage.

Demand satisfied for family planning was defined as the number of women using modern contraception as a proportion of women of reproductive age with a need for family planning. Need was defined as women who were sexually active or partnered, fecund, and did not want a child in the next 2 years, or were currently pregnant or postpartum amenorrhoeic from a birth in the past 2 years who wished to have delayed or prevented their current or most recent pregnancy. Unmet need was defined as the proportion of women of reproductive age who were not currently using, or whose partner was not using, any method of contraception, but had a need for family planning. The denominator in demand satisfied was women with a need for family planning, versus the denominator for unmet need, which was all women, regardless of need. Details on the calculation of demand satisfied and unmet need are included in the appendix (pp 19–20).^44^

Contraceptive method mix was estimated as the proportion of women who were using, or whose partner was using, a specific contraceptive method out of all contraceptive use. If women reported using more than one method, we followed published approaches that roughly prioritised methods based on effectiveness.^45^ For example, if a woman reported using an intrauterine device and a male condom, only intrauterine device was counted as the method of choice (appendix p 12).

We estimated the prevalence of ten different modern contraceptive method types, in order of prioritisation based on effectiveness: female sterilisation, male sterilisation, intrauterine devices, injections, implants, oral contraceptive pills, condom, diaphragm, emergency contraception, and other modern methods.^46^ Four traditional method types were estimated: lactational amenorrhoea method, rhythm method, withdrawal method, and other traditional methods.

### Addressing missing survey questions used to determine need

Some surveys were missing one or more questions required to determine need for family planning: women's fecundity, desire for future children, post-partum amenorrhoeic status, or want for current or previous pregnancy. Questions about these characteristics were not posed to any respondents in some surveys. To address the paucity of information about key determinants of need, we adjusted surveys missing a component with information from complete surveys for each age group using the crosswalk package (version 0.1.0) in R developed for the GBD study.^1^, ^46^ We estimated the mean difference by age group in a quantity of interest with and without a specific item for surveys that had all the information required. We applied this mean difference to estimates from surveys where a question was missing to adjust the quantity of interest for the missing information. Further details on this approach are provided in the appendix (pp 20–32).

### Modelling

To estimate each quantity of interest for all 204 countries and territories between 1970 and 2019, we used Spatiotemporal Gaussian process regression (ST-GPR),^47^ a highly flexible estimation method used extensively in the GBD study that closely follows data where they exist and uses covariates and spatiotemporal patterns to generate the best estimate of quantities of interest where data are not available.^1^ ST-GPR synthesises data with high variability by incorporating covariates and incorporating information across geography, age groups, and time to produce comprehensive time series estimates with corresponding uncertainty. Where data were missing for a specific age group, year, and location, estimates were informed by proximal timepoints, age groups, and geographies. Thus, a key assumption is that locations in close proximity have similar levels and trends in family planning outcomes. Our approach thus differed from other existing approaches, such as those used by UN-DESA, by being more flexible, allowing our estimates to be closer to the observed points within a country; or, where country-specific points were not available, by informing estimates using data from nearby geographies and covariates rather than regional or global averages. Further information on these differences is included in the appendix (pp 51–53).

The first stage of the model fit a linear mixed-effects regression with fixed effects on specified covariates and random effects (intercepts) on geography (location, GBD region, and GBD super-region). A normal distribution was used for the random effects. The second stage smoothed the residuals between the regression fit and the data using a locally weighted polynomial regression function across time, age, and geography to generate a non-linear trend that better followed available data in each location. The third stage used that trend as a mean function in a Gaussian process regression to account for input data variance and model uncertainty, and to generate 1000 draws, which were used to produce 95% uncertainty intervals [UIs] around the final estimates.

We fit separate ST-GPR models for partnered and unpartnered women for each family planning indicator. Each first stage model selected between two possible suites of covariates based on goodness of fit (minimum root mean squared error). The first suite included mean years of education among all women and the natural logarithm (base e) of 10-year lag-distributed income per capita and the second suite included the Socio-demographic Index (SDI).^48^, ^49^ All models also fit separate intercept terms for each age group (age 15–19, 20–24, 25–29, 30–34, 35–39, 40–44, and 45–49 years) and random effects for location (using a normal distribution), GBD region, and GBD super-region. Since fewer datapoints were available to estimate unmet need, we included the CPR as a covariate in those models. Similarly, models of family planning indicators for unpartnered women tended to have fewer datapoints, so the corresponding indicator for partnered women was included as a covariate in all unpartnered models. Additional details on the modelling process and the specific covariates used for each of the first-stage ST-GPR models are included in the appendix (pp 32–45).

We constrained quantities of interest to estimated prevalence and used a nested proportion approach to ensure all quantities of interest were internally consistent, which for example, guaranteed that the mCPR did not exceed the CPR. We modelled the following metrics: CPR; the share of all women using a given method; and the share of women defined as having a need for contraception divided by non-users. To ensure the sum of all methods did not exceed the CPR, we raked the sum of individual methods to the CPR for each country and year. The raked estimates of all modern methods were then totalled to produce the mCPR. The modelled results of the proportion of non-users with unmet need were multiplied by 1–CPR to generate estimates of unmet need. Share of need met with modern methods (SDG 3.7.1) was then computed by dividing the mCPR by the CPR plus the share of women with unmet need. After generating age-specific estimates of the modelled indicators for partnered and unpartnered women, we aggregated to all women using the modelled marital status estimates and women aged 15–49 years using GBD age group population estimates. We then generated age-standardised estimates for women aged 15–49 years in all locations and years, weighting estimates from each age group by the relative population size of that age group globally in 2019. To depict trends over time, we estimated the absolute change between 1970 and 2019 by subtracting 1970 estimates from 2019 estimates.

### Family planning and social and economic development

We examined broad trends in family planning using SDI, a composite indicator of social and economic development.^49^ SDI is the geometric mean of indices of the total fertility rate among women younger than 25 years, mean education for those aged 15 years or older, and lag-distributed income per capita. Higher SDI values correspond to higher levels of development (range 0–100). SDI levels were categorised as low (0·0 to <45·5), low-middle (45·5 to <60·8), middle (60·8 to <69·0), high-middle (69·0 to <80·5), and high (80·5 to 100·0). We examined the association between demand satisfied and the mCPR according to SDI levels for all locations for the period of 1970–2019 to assess how these indicators changed with social and economic development. We used the GBD study meta-regression Bayesian, regularised, trimmed method for analyses for age-standardised women of reproductive age and for each age group, with demand satisfied or the mCPR as the response variables and SDI as the predictor.^50^

### Uncertainty analysis

We estimated uncertainty by generating 1000 draws through ST-GPR for each family planning indicator. We defined the 95% UI by taking the 0·025 quantile for the lower bound of uncertainty and the 0·975 quantile for the upper bound of uncertainty. The mean was taken across the draws to calculate the point estimate. Analyses were done using R (version 4.0.5).

### Role of the funding source

The funder of the study had no role in the study design, data collection, data analysis, data interpretation, or writing of the report.

## Results

### Family planning indicators across time and locations

**Figure 1:**
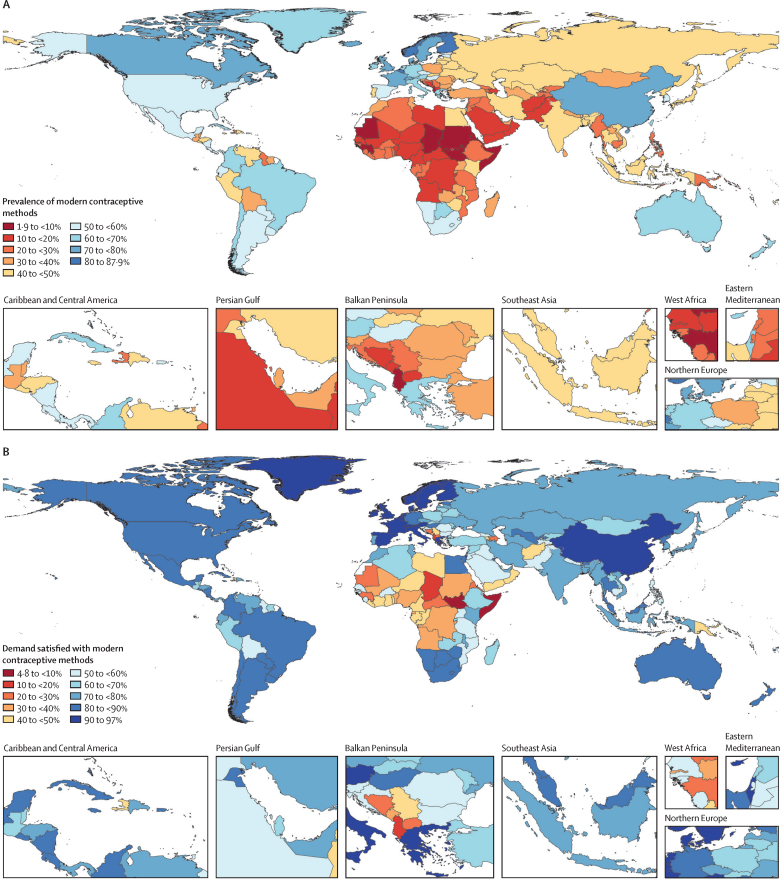
Prevalence of modern contraceptive methods among women of reproductive age (15–49 years) and demand satisfied with modern contraceptive methods by location, 2019

In 2019, average contraceptive prevalence was 51·9% (95% UI 51·0–52·8), the global mCPR was 47·7% (46·9–48·6), and demand satisfied was 79·1% (78·5–79·8), with considerable differences observed across countries and territories (table 1). The mCPR ranged from 1·9% in South Sudan to 87·9% in Norway in 2019 (figure 1). Countries in southeast Asia, east Asia, and Oceania had the highest rates of average mCPR (64·7% [63·3–65·9]) and demand satisfied (90·4% [89·5–91·2]), while countries in the sub-Saharan Africa super-region had the lowest values for mCPR (23·6% [23·1–24·2]) and demand satisfied (52·0% [51·2–52·8]) on average in 2019.

**Table 1:** Contraceptive prevalence, modern contraceptive prevalence, demand satisfied with modern methods, and unmet need for any method, 2019, and absolute percentage point changes for the period 1970 to 2019

		**CPR, 2019**	Absolute change in CPR, 1970–2019*	**mCPR, 2019**	Absolute change in mCPR, 1970–2019*	**Demand satisfied, 2019**	Absolute change in demand satisfied, 1970–2019*	**Unmet need, 2019**	Absolute change in unmet need, 1970–2019*
**Global**		**51·9% (51·0 to 52·8)**	**18·7 (17·1 to 20·3)**	**47·7% (46·9 to 48·6)**	**20·1 (18·7 to 21·6)**	**79·1% (78·5 to 79·8)**	**24·3 (22·6 to 26·1)**	**8·3% (8·0 to 8·7)**	**−8·7 (−9·5 to −7·8)**
	Low SDI	25·9% (25·2 to 26·6)	16·1 (15·0 to 17·1)	22·0% (21·4 to 22·6)	16·9 (16·2 to 17·6)	48·7% (47·8 to 49·6)	35·3 (34·0 to 36·6)	19·2% (18·8 to 19·6)	−8·7 (−9·6 to −7·9)
	Low-middle SDI	40·5% (38·1 to 43·0)	25·4 (21·9 to 28·5)	36·2% (34·0 to 38·4)	26·0 (23·3 to 28·7)	71·2% (69·0 to 73·2)	44·8 (41·0 to 48·5)	10·3% (9·4 to 11·3)	−13·0 (−14·8 to −11·2)
	Middle SDI	51·0% (49·9 to 52·0)	24·9 (23·0 to 26·9)	45·8% (44·7 to 46·9)	25·8 (24·1 to 27·4)	78·5% (77·5 to 79·3)	33·8 (31·5 to 36·1)	7·4% (7·1 to 7·8)	−11·4 (−12·5 to −10·4)
	High-middle SDI	69·2% (67·7 to 70·4)	28·0 (25·1 to 31·2)	65·7% (64·2 to 66·8)	30·5 (27·6 to 33·5)	89·1% (88·2 to 89·9)	26·5 (23·0 to 30·4)	4·5% (4·0 to 5·1)	−10·6 (−12·6 to −8·7)
	High SDI	66·2% (64·5 to 67·8)	14·3 (11·9 to 16·8)	61·7% (60·3 to 63·3)	15·7 (13·3 to 18·1)	87·2% (86·5 to 87·9)	11·2 (9·9 to 12·5)	4·6% (4·3 to 4·9)	−4·1 (−4·7 to −3·4)
**Central Europe, eastern Europe, and central Asia**		**50·5% (48·5 to 52·2)**	**15·0 (11·6 to 18·1)**	**40·4% (38·8 to 42·0)**	**17·3 (14·6 to 19·8)**	**68·0% (66·5 to 69·5)**	**23·4 (20·4 to 26·4)**	**8·9% (8·3 to 9·7)**	**−7·3 (−8·8 to −5·8)**
Central Asia		42·2% (40·2 to 44·1)	13·2 (10·1 to 16·4)	35·9% (34·1 to 37·8)	14·8 (12·1 to 17·6)	71·2% (69·6 to 72·6)	22·1 (18·8 to 25·4)	8·3% (7·7 to 8·9)	−5·7 (−7·0 to −4·5)
	Armenia	39·6% (35·9 to 43·5)	9·6 (2·7 to 16·6)	20·2% (17·4 to 23·4)	9·8 (5·9 to 13·9)	42·8% (38·0 to 47·5)	18·8 (11·9 to 25·9)	7·4% (6·2 to 8·9)	−5·7 (−8·8 to −2·8)
	Azerbaijan	39·8% (35·2 to 44·5)	12·8 (5·3 to 20·3)	12·1% (9·7 to 14·9)	5·8 (2·7 to 9·1)	27·2% (22·8 to 32·3)	9·9 (3·3 to 16·0)	4·6% (3·6 to 5·7)	−4·6 (−6·7 to −2·4)
	Georgia	29·5% (25·9 to 33·0)	7·5 (1·4 to 13·5)	21·7% (18·8 to 24·7)	9·3 (4·8 to 13·3)	50·1% (45·4 to 54·8)	17·0 (8·7 to 24·6)	13·9% (12·1 to 15·7)	−1·6 (−4·8 to 1·8)
	Kazakhstan	44·2% (40·3 to 47·9)	13·8 (6·8 to 20·9)	41·7% (38·0 to 45·3)	17·0 (10·8 to 23·2)	78·0% (74·5 to 81·1)	24·6 (17·4 to 32·3)	9·3% (7·9 to 10·8)	−6·5 (−9·7 to −3·3)
	Kyrgyzstan	29·6% (26·3 to 33·2)	5·0 (−1·6 to 11·3)	27·7% (24·6 to 31·1)	6·3 (0·4 to 11·9)	64·6% (60·2 to 68·9)	9·4 (0·8 to 18·6)	13·3% (11·8 to 14·9)	−0·9 (−3·9 to 1·9)
	Mongolia	40·8% (37·0 to 44·7)	15·5 (8·7 to 22·0)	38·2% (34·6 to 41·8)	19·3 (13·8 to 24·5)	67·6% (63·6 to 71·6)	29·4 (21·4 to 37·2)	15·6% (13·6 to 17·7)	−8·2 (−12·0 to −4·4)
	Tajikistan	22·5% (19·8 to 25·7)	5·2 (−0·6 to 11·1)	20·3% (17·8 to 23·3)	5·6 (0·6 to 10·9)	52·8% (48·2 to 57·7)	14·3 (4·3 to 24·8)	16·0% (14·2 to 17·7)	−4·7 (−8·1 to −1·4)
	Turkmenistan	35·4% (32·3 to 38·6)	8·1 (1·9 to 14·7)	32·8% (29·8 to 35·7)	10·3 (4·8 to 15·9)	76·9% (73·7 to 79·8)	19·6 (12·3 to 27·9)	7·2% (6·2 to 8·2)	−4·7 (−7·3 to −2·3)
	Uzbekistan	52·3% (47·6 to 56·7)	16·6 (9·0 to 24·0)	49·2% (44·7 to 53·4)	17·6 (10·7 to 24·5)	86·1% (83·1 to 88·6)	19·1 (12·9 to 26·4)	4·8% (3·6 to 6·2)	−6·5 (−9·4 to −4·1)
Central Europe		52·3% (50·7 to 54·0)	21·1 (18·2 to 23·8)	38·3% (36·8 to 39·9)	20·9 (19·0 to 22·9)	60·5% (58·9 to 62·1)	26·3 (23·8 to 28·9)	10·9% (10·1 to 11·7)	−8·6 (−10·1 to −7·2)
	Albania	39·1% (34·5 to 43·5)	14·6 (7·3 to 21·1)	7·1% (5·7 to 8·7)	4·0 (2·2 to 5·8)	14·3% (12·0 to 17·2)	7·0 (3·7 to 10·4)	10·5% (8·8 to 12·2)	−7·5 (−11·0 to −3·9)
	Bosnia and Herzegovina	37·0% (31·8 to 42·5)	25·1 (18·6 to 31·2)	15·2% (12·5 to 18·1)	12·3 (9·3 to 15·5)	25·8% (21·6 to 30·3)	19·9 (15·2 to 24·8)	21·8% (18·1 to 25·3)	−14·9 (−19·9 to −10·0)
	Bulgaria	57·1% (51·7 to 62·1)	17·8 (9·8 to 26·2)	37·6% (33·3 to 41·9)	18·8 (13·4 to 24·5)	56·8% (51·8 to 61·7)	23·8 (17·1 to 30·9)	9·0% (6·9 to 11·2)	−8·3 (−12·4 to −4·3)
	Croatia	46·0% (40·0 to 51·7)	17·8 (9·6 to 25·8)	29·5% (25·2 to 34·1)	15·5 (9·9 to 20·8)	51·6% (46·2 to 57·2)	21·9 (14·3 to 29·7)	11·2% (8·7 to 13·8)	−7·8 (−11·8 to −4·0)
	Czechia	58·2% (53·1 to 63·1)	14·0 (6·4 to 21·8)	50·6% (46·1 to 55·1)	18·8 (12·1 to 25·3)	76·5% (72·6 to 80·0)	19·8 (13·0 to 26·6)	7·9% (6·2 to 9·9)	−3·9 (−7·1 to −0·9)
	Hungary	59·9% (55·1 to 65·0)	9·2 (2·0 to 16·8)	53·2% (48·5 to 57·8)	16·2 (10·2 to 22·6)	81·1% (77·9 to 83·8)	17·5 (12·7 to 22·8)	5·7% (4·3 to 7·2)	−1·7 (−3·7 to 0·5)
	Montenegro	21·8% (18·4 to 25·9)	8·1 (2·3 to 13·4)	15·6% (12·9 to 18·7)	8·3 (4·9 to 11·8)	39·7% (34·8 to 44·9)	20·4 (13·6 to 27·3)	17·4% (15·5 to 19·2)	−6·5 (−9·6 to −3·5)
	North Macedonia	41·9% (37·3 to 46·6)	30·9 (24·9 to 36·6)	15·5% (13·5 to 17·5)	9·7 (6·9 to 12·4)	25·6% (22·8 to 28·5)	13·6 (8·9 to 17·8)	18·6% (15·6 to 21·5)	−18·6 (−22·9 to −14·2)
	Poland	48·1% (44·3 to 51·9)	24·5 (18·0 to 30·4)	39·2% (35·7 to 42·4)	23·9 (19·2 to 28·7)	63·6% (59·7 to 67·3)	30·8 (23·6 to 37·9)	13·5% (11·7 to 15·6)	−9·4 (−13·1 to −5·7)
	Romania	59·8% (54·2 to 65·1)	28·1 (20·1 to 35·8)	39·7% (35·1 to 43·9)	29·4 (24·3 to 34·4)	58·6% (54·1 to 62·9)	38·3 (32·3 to 44·0)	7·9% (5·9 to 10·1)	−10·7 (−14·4 to −7·0)
	Serbia	50·7% (47·2 to 54·1)	24·2 (17·5 to 30·6)	25·8% (23·2 to 28·3)	14·6 (10·8 to 18·1)	42·8% (39·9 to 45·8)	21·0 (15·5 to 25·5)	9·5% (8·3 to 11·1)	−15·4 (−19·0 to −11·5)
	Slovakia	59·0% (53·5 to 64·0)	20·5 (12·3 to 28·2)	47·4% (42·6 to 52·1)	20·1 (13·7 to 26·5)	66·9% (62·1 to 71·2)	18·6 (11·8 to 25·7)	11·8% (9·6 to 14·4)	−6·1 (−10·0 to −2·3)
	Slovenia	50·6% (45·4 to 56·1)	15·3 (7·4 to 23·3)	35·5% (30·9 to 40·5)	15·4 (9·7 to 21·4)	59·6% (54·7 to 64·9)	18·7 (12·1 to 25·7)	8·8% (7·0 to 10·8)	−4·9 (−8·1 to −1·6)
Eastern Europe		53·5% (50·1 to 56·8)	14·5 (8·6 to 20·1)	43·7% (40·4 to 46·7)	17·2 (12·7 to 21·9)	70·7% (68·0 to 73·4)	21·7 (16·8 to 26·7)	8·2% (7·0 to 9·6)	−6·7 (−9·2 to −4·1)
	Belarus	48·5% (44·5 to 52·6)	15·2 (8·4 to 22·5)	42·4% (38·8 to 45·8)	17·8 (12·2 to 23·3)	68·9% (65·4 to 72·0)	21·5 (14·7 to 28·4)	12·9% (11·2 to 14·7)	−5·4 (−8·9 to −2·0)
	Estonia	57·4% (52·7 to 62·0)	16·1 (8·7 to 23·7)	53·9% (49·5 to 58·4)	18·7 (12·0 to 25·7)	84·9% (82·0 to 87·5)	17·8 (12·1 to 23·9)	6·0% (4·8 to 7·4)	−5·0 (−7·7 to −2·5)
	Latvia	55·5% (50·6 to 60·3)	13·1 (5·3 to 20·9)	48·0% (43·5 to 52·5)	17·7 (10·9 to 24·3)	76·7% (73·0 to 79·9)	21·7 (15·1 to 28·2)	7·1% (5·6 to 8·8)	−5·6 (−9·0 to −2·5)
	Lithuania	48·0% (43·1 to 53·2)	19·5 (11·1 to 27·2)	41·0% (36·6 to 45·6)	22·6 (16·2 to 28·3)	68·3% (63·6 to 72·7)	33·2 (25·1 to 40·1)	12·0% (9·5 to 14·5)	−11·8 (−15·9 to −7·3)
	Moldova	54·1% (49·2 to 58·4)	14·7 (7·8 to 22·0)	39·5% (35·5 to 43·8)	14·3 (8·2 to 20·2)	61·9% (57·4 to 66·5)	12·0 (4·3 to 19·0)	9·8% (7·9 to 11·7)	−1·3 (−4·0 to 1·5)
	Russia	53·6% (48·8 to 58·0)	16·5 (8·3 to 24·3)	43·9% (39·6 to 48·0)	18·9 (12·5 to 25·0)	70·9% (67·1 to 74·6)	23·4 (16·5 to 30·5)	8·3% (6·6 to 10·2)	−7·3 (−10·9 to −3·5)
	Ukraine	54·4% (50·0 to 58·8)	8·7 (0·6 to 16·2)	43·5% (39·7 to 47·8)	12·5 (6·2 to 18·9)	71·0% (67·0 to 74·8)	17·7 (11·0 to 24·5)	6·8% (5·5 to 8·2)	−5·5 (−8·4 to −2·4)
**High income**		**66·3% (64·9 to 67·8)**	**15·1 (12·8 to 17·6)**	**62·2% (60·8 to 63·6)**	**16·8 (14·6 to 18·9)**	**87·5% (86·9 to 88·1)**	**11·2 (10·0 to 12·3)**	**4·7% (4·4 to 5·0)**	**−3·5 (−4·1 to −3·0)**
Australasia		68·5% (64·1 to 72·6)	20·0 (12·3 to 27·1)	64·6% (60·3 to 68·3)	23·5 (16·8 to 29·8)	86·2% (84·1 to 88·0)	19·7 (15·2 to 24·5)	6·4% (5·3 to 7·7)	−6·8 (−9·2 to −4·4)
	Australia	68·4% (63·2 to 73·0)	21·7 (12·7 to 30·1)	64·6% (59·7 to 69·0)	25·4 (17·4 to 33·0)	86·2% (83·7 to 88·4)	21·5 (16·0 to 27·2)	6·5% (5·1 to 8·0)	−7·4 (−10·3 to −4·6)
	New Zealand	68·8% (63·5 to 73·7)	12·2 (3·6 to 20·7)	64·4% (59·5 to 69·0)	15·1 (7·4 to 22·6)	85·9% (83·6 to 88·0)	12·1 (7·8 to 17·1)	6·1% (4·8 to 7·6)	−4·0 (−6·8 to −1·6)
High-income Asia Pacific		58·7% (54·7 to 62·7)	13·7 (7·7 to 19·7)	51·5% (47·9 to 55·2)	14·4 (9·6 to 19·6)	75·7% (73·2 to 78·2)	14·0 (9·9 to 18·2)	9·3% (7·9 to 10·6)	−5·7 (−7·9 to −3·6)
	Brunei	61·9% (56·1 to 68·0)	13·9 (5·3 to 23·1)	52·9% (47·7 to 57·9)	16·3 (9·3 to 23·4)	73·3% (69·5 to 76·7)	18·4 (12·8 to 23·9)	10·2% (8·2 to 12·5)	−8·3 (−11·9 to −4·3)
	Japan	51·4% (45·5 to 57·4)	1·8 (−6·0 to 9·9)	45·9% (40·5 to 51·2)	3·9 (−2·5 to 11·4)	73·7% (69·7 to 77·4)	7·3 (2·1 to 13·1)	10·8% (8·8 to 12·8)	−2·8 (−5·6 to −0·1)
	Singapore	87·5% (84·5 to 90·1)	36·7 (29·7 to 43·1)	82·0% (79·1 to 84·6)	41·4 (35·2 to 47·2)	90·1% (88·5 to 91·5)	27·3 (22·9 to 31·8)	3·5% (2·6 to 4·6)	−10·2 (−12·5 to −7·7)
	South Korea	70·2% (65·9 to 74·4)	44·4 (38·0 to 50·4)	59·3% (55·2 to 63·4)	42·3 (37·1 to 47·2)	77·2% (74·3 to 80·0)	40·6 (35·1 to 46·5)	6·7% (5·5 to 8·0)	−14·0 (−16·7 to −11·2)
High-income North America		65·1% (62·2 to 68·3)	12·9 (7·5 to 17·9)	60·4% (57·6 to 63·6)	12·2 (7·2 to 16·9)	88·8% (87·5 to 90·0)	4·5 (2·4 to 6·5)	2·9% (2·5 to 3·3)	−2·1 (−2·9 to −1·3)
	Canada	72·8% (67·8 to 77·0)	14·8 (6·7 to 22·5)	70·8% (66·0 to 75·0)	15·8 (8·0 to 23·3)	89·2% (86·6 to 91·3)	9·2 (4·6 to 13·7)	6·6% (5·1 to 8·4)	−4·2 (−7·0 to −1·5)
	Greenland	68·5% (63·4 to 73·8)	13·8 (6·0 to 21·6)	64·0% (59·4 to 69·1)	14·0 (6·6 to 21·1)	90·8% (89·3 to 92·2)	6·0 (3·3 to 8·8)	2·0% (1·6 to 2·6)	−2·3 (−3·3 to −1·3)
	USA	64·3% (60·9 to 67·8)	12·7 (6·7 to 18·2)	59·3% (56·2 to 62·7)	11·8 (6·2 to 17·0)	88·8% (87·3 to 90·0)	3·9 (1·6 to 6·1)	2·5% (2·1 to 3·0)	−1·9 (−2·8 to −1·0)
Southern Latin America		62·0% (60·2 to 63·9)	19·6 (14·9 to 24·4)	60·5% (58·7 to 62·3)	20·8 (16·3 to 25·3)	84·7% (83·6 to 85·8)	14·3 (10·6 to 18·5)	9·4% (8·6 to 10·1)	−4·5 (−6·4 to −2·7)
	Argentina	60·7% (58·6 to 62·7)	18·7 (12·7 to 25·1)	59·1% (57·1 to 61·1)	19·6 (14·2 to 25·6)	83·6% (82·1 to 85·0)	14·0 (8·8 to 19·9)	10·1% (9·2 to 11·0)	−4·7 (−7·3 to −2·1)
	Chile	66·2% (62·0 to 70·5)	22·6 (16·1 to 29·3)	64·7% (60·6 to 68·9)	24·3 (18·1 to 30·5)	87·8% (85·3 to 89·9)	15·1 (10·4 to 20·3)	7·4% (6·1 to 9·0)	−4·4 (−7·0 to −1·9)
	Uruguay	58·4% (53·1 to 63·6)	15·7 (8·1 to 23·7)	56·9% (51·7 to 62·0)	17·4 (10·4 to 24·9)	83·0% (79·5 to 86·0)	12·8 (7·0 to 19·1)	10·2% (8·4 to 12·1)	−3·4 (−6·4 to −0·5)
Western Europe		71·2% (69·6 to 72·8)	16·8 (14·0 to 19·6)	68·4% (66·8 to 70·0)	20·2 (17·6 to 22·7)	91·6% (91·0 to 92·1)	12·5 (11·1 to 13·8)	3·5% (3·2 to 3·8)	−3·0 (−3·6 to −2·3)
	Andorra	73·4% (68·0 to 77·9)	11·3 (3·7 to 18·9)	71·0% (65·9 to 75·4)	14·6 (7·4 to 21·9)	93·5% (92·2 to 94·6)	8·0 (5·5 to 10·7)	2·5% (1·9 to 3·3)	−1·3 (−2·4 to −0·1)
	Austria	72·2% (68·4 to 75·4)	24·7 (17·4 to 31·9)	69·8% (66·2 to 73·0)	26·5 (19·8 to 33·1)	90·0% (88·3 to 91·4)	16·3 (11·7 to 21·3)	5·4% (4·4 to 6·6)	−5·9 (−8·4 to −3·5)
	Belgium	83·6% (80·1 to 86·6)	10·9 (5·5 to 16·9)	80·6% (77·1 to 83·6)	13·3 (7·9 to 19·1)	94·5% (93·6 to 95·4)	6·1 (4·1 to 8·5)	1·6% (1·2 to 2·1)	−1·8 (−2·7 to −0·9)
	Cyprus	72·7% (67·7 to 77·5)	30·9 (22·9 to 39·1)	70·0% (65·1 to 74·6)	34·0 (26·6 to 41·5)	92·7% (91·2 to 93·9)	18·5 (14·5 to 22·8)	2·8% (2·1 to 3·8)	−3·8 (−5·4 to −2·4)
	Denmark	77·5% (73·2 to 81·6)	10·5 (3·5 to 18·0)	75·3% (71·0 to 79·4)	14·5 (7·9 to 21·3)	94·7% (93·7 to 95·5)	8·6 (6·3 to 10·9)	2·0% (1·5 to 2·6)	−1·5 (−2·6 to −0·5)
	Finland	82·3% (78·6 to 85·5)	20·5 (13·7 to 27·2)	81·3% (77·6 to 84·4)	21·3 (14·6 to 27·9)	96·7% (96·1 to 97·3)	6·0 (4·3 to 8·2)	1·7% (1·3 to 2·2)	−2·6 (−3·8 to −1·6)
	France	79·0% (74·6 to 83·0)	22·0 (14·6 to 29·7)	76·5% (72·3 to 80·5)	31·4 (25·0 to 38·4)	91·6% (89·7 to 93·2)	24·7 (20·4 to 29·4)	4·5% (3·3 to 5·9)	−5·8 (−8·4 to −3·3)
	Germany	64·8% (59·8 to 70·1)	7·8 (−0·3 to 16·1)	63·2% (58·4 to 68·4)	9·5 (1·7 to 17·2)	89·5% (87·3 to 91·5)	7·6 (3·7 to 12·2)	5·8% (4·6 to 7·2)	−2·8 (−5·2 to −0·6)
	Greece	67·8% (62·0 to 73·1)	21·4 (13·0 to 31·0)	64·9% (59·5 to 69·9)	24·9 (17·1 to 33·2)	91·3% (89·7 to 92·8)	16·4 (12·5 to 20·6)	3·2% (2·4 to 4·2)	−3·7 (−5·5 to −2·0)
	Iceland	76·5% (71·7 to 80·7)	15·5 (7·7 to 22·8)	74·2% (69·6 to 78·3)	20·0 (12·5 to 26·8)	94·5% (93·5 to 95·3)	10·9 (8·4 to 13·7)	2·0% (1·6 to 2·7)	−1·9 (−3·0 to −0·8)
	Ireland	67·4% (61·3 to 72·5)	28·4 (19·1 to 36·8)	64·2% (58·7 to 69·2)	30·4 (22·0 to 37·8)	91·0% (89·3 to 92·4)	15·8 (11·8 to 19·8)	3·1% (2·4 to 4·1)	−2·8 (−4·2 to −1·4)
	Israel	72·4% (67·4 to 77·2)	14·3 (5·9 to 21·9)	69·6% (64·7 to 74·2)	18·4 (11·1 to 25·5)	92·6% (91·2 to 93·8)	11·1 (8·0 to 14·3)	2·7% (2·0 to 3·5)	−1·9 (−3·2 to −0·7)
	Italy	69·4% (63·7 to 74·4)	20·0 (12·0 to 28·2)	66·5% (61·1 to 71·3)	23·5 (16·1 to 30·9)	91·7% (90·1 to 93·1)	13·8 (10·5 to 17·3)	3·2% (2·3 to 4·1)	−2·7 (−4·2 to −1·2)
	Luxembourg	79·4% (75·2 to 83·2)	16·4 (9·0 to 23·6)	77·0% (72·9 to 80·8)	20·4 (13·7 to 27·2)	94·5% (93·5 to 95·4)	10·2 (7·8 to 12·9)	2·1% (1·6 to 2·8)	−2·0 (−3·2 to −0·9)
	Malta	70·1% (64·4 to 75·5)	33·1 (23·7 to 41·5)	67·2% (61·8 to 72·5)	35·7 (27·3 to 43·3)	91·5% (89·8 to 93·0)	19·4 (14·9 to 23·9)	3·3% (2·5 to 4·3)	−3·3 (−4·8 to −1·6)
	Monaco	85·1% (81·6 to 88·1)	12·3 (6·4 to 18·7)	82·9% (79·5 to 85·8)	15·1 (9·5 to 21·2)	95·7% (95·0 to 96·4)	6·0 (4·3 to 8·0)	1·5% (1·1 to 2·0)	−1·3 (−2·1 to −0·5)
	Netherlands	67·3% (61·7 to 72·2)	13·8 (6·2 to 21·0)	63·1% (57·8 to 67·8)	14·4 (7·3 to 21·2)	90·2% (88·5 to 91·7)	5·8 (3·0 to 8·4)	2·6% (2·0 to 3·5)	−1·6 (−2·6 to −0·4)
	Norway	89·4% (86·8 to 91·7)	10·2 (5·3 to 15·0)	87·9% (85·4 to 90·2)	12·3 (7·4 to 17·0)	97·0% (96·5 to 97·5)	4·8 (3·4 to 6·1)	1·2% (0·9 to 1·6)	−1·6 (−2·5 to −0·8)
	Portugal	63·4% (58·9 to 67·5)	31·1 (23·7 to 38·4)	49·1% (45·1 to 53·2)	29·8 (24·2 to 35·0)	73·3% (69·0 to 77·0)	25·4 (19·0 to 31·4)	3·6% (2·9 to 4·5)	−4·5 (−6·2 to −2·9)
	San Marino	76·4% (71·7 to 80·6)	9·5 (2·5 to 16·6)	74·1% (69·7 to 78·3)	13·7 (7·0 to 20·5)	94·3% (93·2 to 95·3)	8·6 (6·3 to 11·1)	2·2% (1·6 to 2·9)	−1·5 (−2·6 to −0·4)
	Spain	59·6% (55·5 to 63·1)	22·0 (14·8 to 29·2)	57·2% (53·4 to 60·7)	27·5 (21·0 to 33·8)	90·1% (88·8 to 91·3)	22·7 (18·2 to 27·6)	3·9% (3·3 to 4·6)	−2·6 (−4·1 to −1·1)
	Sweden	75·2% (70·8 to 79·4)	16·9 (9·7 to 24·6)	70·7% (66·3 to 74·6)	18·7 (12·2 to 25·8)	90·7% (89·2 to 92·0)	8·2 (5·4 to 11·2)	2·8% (2·2 to 3·4)	−1·9 (−3·0 to −0·9)
	Switzerland	80·5% (76·4 to 84·1)	6·1 (−0·3 to 12·5)	78·5% (74·6 to 82·2)	9·6 (3·5 to 15·6)	95·3% (94·4 to 96·1)	5·7 (3·9 to 7·6)	1·9% (1·4 to 2·5)	−0·7 (−1·5 to 0·2)
	UK	76·8% (72·5 to 80·6)	14·7 (7·3 to 21·4)	74·0% (69·9 to 77·7)	15·9 (8·9 to 22·3)	95·1% (94·0 to 95·9)	4·6 (2·8 to 6·6)	1·1% (0·8 to 1·4)	−1·0 (−1·6 to −0·4)
**Latin America and Caribbean**		**58·3% (56·6 to 59·9)**	**29·6 (26·8 to 32·2)**	**53·7% (52·1 to 55·2)**	**31·2 (28·6 to 33·5)**	**81·7% (80·7 to 82·7)**	**34·2 (31·3 to 37·1)**	**7·4% (6·9 to 7·9)**	**−11·2 (−12·5 to −9·9)**
Andean Latin America		53·5% (52·2 to 54·7)	31·5 (28·0 to 34·7)	42·1% (40·9 to 43·2)	32·0 (30·1 to 33·8)	69·9% (68·7 to 71·0)	47·2 (43·9 to 50·1)	6·7% (6·2 to 7·3)	−15·5 (−17·5 to −13·7)
	Bolivia	46·6% (43·6 to 49·5)	25·1 (19·4 to 30·8)	33·2% (30·8 to 35·7)	26·7 (23·7 to 29·7)	59·3% (56·2 to 62·4)	45·6 (40·9 to 50·3)	9·4% (7·9 to 10·9)	−16·7 (−20·4 to −12·9)
	Ecuador	58·3% (54·8 to 61·4)	35·6 (29·5 to 41·3)	51·8% (48·6 to 54·8)	36·1 (31·2 to 40·7)	79·9% (77·4 to 82·2)	43·3 (35·8 to 50·2)	6·5% (5·3 to 7·8)	−13·6 (−16·8 to −10·2)
	Peru	53·3% (52·1 to 54·5)	31·5 (26·7 to 35·8)	40·0% (39·0 to 41·2)	31·3 (28·9 to 33·6)	67·5% (66·3 to 68·8)	47·7 (43·2 to 51·8)	6·0% (5·5 to 6·5)	−16·0 (−18·7 to −13·2)
Caribbean		47·1% (45·8 to 48·4)	17·6 (14·8 to 20·5)	45·1% (43·9 to 46·3)	19·4 (16·8 to 22·1)	73·0% (71·6 to 74·2)	22·6 (19·4 to 26·0)	14·8% (14·0 to 15·6)	−6·8 (−8·3 to −5·2)
	Antigua and Barbuda	58·7% (53·5 to 63·5)	30·5 (23·3 to 36·8)	56·6% (51·5 to 61·4)	34·4 (27·8 to 40·3)	85·4% (82·7 to 87·9)	31·2 (25·3 to 37·0)	7·5% (6·0 to 9·1)	−5·1 (−7·8 to −2·5)
	The Bahamas	61·1% (56·1 to 65·3)	18·6 (11·9 to 25·5)	59·7% (54·9 to 63·9)	19·8 (13·3 to 26·6)	87·9% (85·3 to 90·1)	15·8 (10·0 to 21·5)	6·8% (5·5 to 8·3)	−6·0 (−8·7 to −3·0)
	Barbados	50·1% (45·4 to 54·5)	18·0 (10·8 to 25·1)	46·9% (42·5 to 51·2)	19·2 (12·7 to 25·7)	70·1% (65·2 to 74·5)	27·4 (19·4 to 35·1)	16·8% (13·9 to 20·0)	−16·0 (−21·1 to −10·4)
	Belize	40·6% (37·3 to 44·0)	15·0 (8·4 to 21·1)	38·3% (35·2 to 41·6)	17·7 (12·1 to 23·1)	63·6% (59·6 to 67·5)	26·3 (18·3 to 33·8)	19·6% (17·2 to 21·8)	−10·0 (−14·5 to −5·3)
	Bermuda	68·9% (63·9 to 73·2)	27·4 (19·9 to 34·5)	68·0% (63·1 to 72·3)	29·7 (22·5 to 36·2)	89·8% (87·4 to 91·8)	21·7 (15·8 to 27·7)	6·9% (5·5 to 8·5)	−7·9 (−11·1 to −4·9)
	Cuba	68·8% (66·2 to 71·2)	26·9 (19·3 to 33·9)	67·8% (65·2 to 70·1)	27·5 (20·1 to 34·3)	86·4% (84·8 to 87·9)	23·0 (16·4 to 29·7)	9·6% (8·5 to 10·7)	−12·0 (−15·4 to −8·7)
	Dominica	51·1% (46·3 to 55·9)	22·5 (16·3 to 28·7)	50·2% (45·4 to 54·9)	26·2 (20·2 to 32·0)	85·2% (82·1 to 87·9)	29·9 (23·4 to 36·7)	7·8% (6·3 to 9·2)	−6·9 (−9·7 to −4·3)
	Dominican Republic	51·1% (48·7 to 53·5)	29·2 (24·6 to 34·0)	49·7% (47·3 to 52·0)	31·5 (27·4 to 35·9)	79·8% (77·7 to 81·8)	35·4 (29·0 to 42·3)	11·2% (10·0 to 12·4)	−7·9 (−10·8 to −5·3)
	Grenada	46·7% (41·5 to 51·8)	27·3 (20·2 to 34·0)	45·1% (40·2 to 50·2)	29·2 (22·9 to 35·3)	76·1% (71·5 to 80·5)	38·8 (30·3 to 46·4)	12·6% (10·2 to 14·9)	−10·6 (−14·3 to −6·8)
	Guyana	25·7% (23·1 to 28·4)	4·5 (−0·5 to 9·1)	24·5% (21·9 to 27·1)	6·0 (1·5 to 10·2)	52·8% (48·8 to 56·7)	16·0 (8·4 to 23·0)	20·6% (18·9 to 22·4)	−8·1 (−11·7 to −4·5)
	Haiti	26·0% (23·0 to 29·2)	15·3 (11·0 to 19·3)	23·3% (20·6 to 26·3)	18·4 (15·2 to 21·5)	45·3% (41·1 to 49·9)	33·3 (27·6 to 38·5)	25·5% (23·3 to 27·7)	−5·2 (−8·4 to −1·9)
	Jamaica	46·5% (42·2 to 50·8)	21·5 (15·9 to 27·2)	44·7% (40·5 to 48·8)	22·0 (16·9 to 27·3)	86·2% (83·4 to 88·5)	37·5 (31·8 to 43·4)	5·3% (4·3 to 6·6)	−16·2 (−18·7 to −13·7)
	Puerto Rico	62·4% (58·6 to 66·2)	23·3 (16·9 to 29·8)	58·2% (54·6 to 61·9)	27·0 (21·8 to 32·8)	88·8% (87·0 to 90·3)	20·9 (16·1 to 26·1)	3·1% (2·5 to 4·0)	−3·7 (−5·4 to −2·1)
	Saint Kitts and Nevis	53·9% (48·6 to 58·9)	34·5 (28·2 to 40·6)	52·5% (47·4 to 57·3)	37·5 (31·9 to 43·0)	84·2% (80·9 to 86·9)	42·8 (36·3 to 49·2)	8·4% (6·9 to 10·1)	−8·3 (−10·8 to −5·5)
	Saint Lucia	47·9% (43·3 to 52·8)	25·3 (18·4 to 32·1)	46·2% (41·8 to 50·9)	29·6 (23·5 to 35·4)	70·6% (65·8 to 75·3)	42·8 (34·9 to 50·2)	17·5% (14·6 to 20·4)	−19·5 (−24·5 to −14·1)
	Saint Vincent and the Grenadines	65·0% (60·5 to 69·8)	18·0 (10·4 to 25·7)	63·3% (58·8 to 67·7)	24·2 (17·4 to 31·3)	86·4% (83·5 to 88·9)	26·1 (20·0 to 32·1)	8·2% (6·5 to 10·1)	−9·4 (−13·1 to −5·6)
	Suriname	33·1% (30·6 to 35·6)	12·8 (7·6 to 17·5)	32·5% (30·1 to 34·9)	13·6 (8·5 to 18·0)	60·7% (57·5 to 64·0)	22·7 (14·9 to 30·3)	20·5% (18·8 to 22·1)	−9·1 (−12·9 to −5·4)
	Trinidad and Tobago	38·5% (33·7 to 43·1)	10·5 (4·1 to 17·1)	35·6% (31·3 to 39·8)	12·2 (6·5 to 18·1)	59·8% (54·3 to 64·6)	13·3 (5·7 to 21·7)	21·1% (18·4 to 23·9)	−1·3 (−5·2 to 2·7)
	Virgin Islands	62·7% (57·8 to 67·3)	20·4 (14·0 to 27·1)	61·9% (57·1 to 66·5)	23·7 (17·5 to 30·3)	89·6% (87·2 to 91·6)	17·3 (12·9 to 22·3)	6·4% (5·2 to 7·8)	−4·1 (−6·8 to −1·8)
Central Latin America		54·1% (52·4 to 55·5)	30·5 (27·4 to 33·0)	50·9% (49·3 to 52·3)	33·2 (30·7 to 35·4)	81·4% (80·1 to 82·5)	42·1 (38·6 to 45·4)	8·4% (7·8 to 9·2)	−12·9 (−14·5 to −11·2)
	Colombia	65·0% (61·6 to 68·1)	38·4 (33·1 to 43·7)	60·9% (57·7 to 63·9)	41·5 (36·8 to 45·8)	86·8% (85·3 to 88·2)	39·2 (33·6 to 45·1)	5·2% (4·4 to 6·0)	−8·9 (−11·3 to −6·7)
	Costa Rica	57·1% (54·6 to 59·7)	19·1 (14·9 to 23·5)	55·1% (52·6 to 57·6)	26·6 (22·6 to 30·4)	82·9% (81·1 to 84·7)	21·4 (17·0 to 25·8)	9·3% (8·2 to 10·5)	1·0 (−0·8 to 2·8)
	El Salvador	50·9% (47·3 to 54·4)	31·4 (26·2 to 36·7)	48·0% (44·6 to 51·4)	31·7 (26·9 to 36·6)	83·1% (80·4 to 85·5)	40·7 (34·1 to 47·6)	7·0% (5·7 to 8·3)	−12·0 (−14·8 to −9·4)
	Guatemala	38·9% (35·2 to 42·7)	24·7 (19·2 to 29·8)	33·5% (30·3 to 36·8)	22·4 (17·9 to 26·6)	69·5% (65·8 to 73·4)	38·0 (30·4 to 44·8)	9·2% (7·7 to 10·8)	−11·6 (−14·4 to −8·7)
	Honduras	47·0% (44·9 to 49·2)	21·0 (15·5 to 26·3)	43·8% (41·8 to 45·9)	24·3 (19·7 to 28·5)	78·1% (76·0 to 80·1)	36·2 (29·1 to 42·7)	9·1% (8·1 to 10·1)	−11·5 (−14·6 to −8·1)
	Mexico	52·6% (49·8 to 55·0)	31·8 (26·5 to 36·6)	50·3% (47·8 to 52·6)	35·0 (30·5 to 38·9)	81·5% (79·3 to 83·5)	48·3 (41·6 to 54·6)	9·1% (8·0 to 10·3)	−16·2 (−19·1 to −12·8)
	Nicaragua	57·7% (54·1 to 61·0)	21·2 (14·8 to 27·9)	55·6% (52·2 to 58·7)	24·5 (18·8 to 30·8)	88·8% (86·9 to 90·4)	29·2 (23·3 to 35·9)	4·9% (4·0 to 5·9)	−10·6 (−13·9 to −7·6)
	Panama	53·0% (49·2 to 56·6)	18·8 (13·4 to 24·3)	51·3% (47·6 to 54·8)	22·0 (17·2 to 27·2)	77·8% (74·6 to 80·6)	26·4 (20·5 to 32·0)	12·9% (11·2 to 14·9)	−9·7 (−13·0 to −6·3)
	Venezuela	53·3% (49·2 to 57·4)	23·0 (17·2 to 28·7)	47·6% (43·7 to 51·4)	25·0 (20·1 to 30·0)	74·3% (70·1 to 77·7)	30·9 (25·1 to 36·8)	10·8% (8·7 to 13·1)	−11·2 (−14·6 to −7·7)
Tropical Latin America		66·4% (62·7 to 69·9)	31·7 (25·5 to 37·6)	61·7% (57·9 to 65·2)	32·6 (26·9 to 37·7)	86·3% (84·0 to 88·3)	27·4 (21·2 to 33·1)	5·0% (4·0 to 6·1)	−9·5 (−12·3 to −6·8)
	Brazil	66·8% (63·0 to 70·4)	31·8 (25·4 to 37·8)	62·0% (58·1 to 65·6)	32·5 (26·5 to 37·6)	86·4% (84·1 to 88·5)	27·1 (20·8 to 32·8)	4·9% (3·9 to 6·0)	−9·7 (−12·5 to −6·9)
	Paraguay	54·1% (50·5 to 57·7)	34·8 (29·2 to 40·1)	50·8% (47·6 to 54·4)	42·5 (38·6 to 46·4)	80·8% (78·4 to 83·0)	54·7 (49·7 to 59·5)	8·8% (7·6 to 10·1)	−4·0 (−6·2 to −1·8)
**North Africa and Middle East**		**36·3% (35·2 to 37·4)**	**15·8 (13·6 to 18·0)**	**29·8% (28·8 to 30·7)**	**17·4 (15·8 to 18·9)**	**66·3% (65·0 to 67·5)**	**35·4 (32·7 to 38·1)**	**8·6% (8·2 to 9·0)**	**−10·8 (−11·9 to −9·8)**
	Afghanistan	16·6% (13·9 to 19·3)	4·0 (−1·0 to 8·5)	14·7% (12·3 to 17·2)	6·5 (2·6 to 10·0)	44·7% (39·5 to 50·3)	21·5 (13·0 to 29·4)	16·2% (14·3 to 18·2)	−6·3 (−9·7 to −3·1)
	Algeria	31·5% (28·6 to 34·4)	10·4 (4·2 to 16·3)	26·6% (24·0 to 29·0)	13·7 (9·1 to 17·9)	67·2% (64·0 to 70·1)	33·5 (25·5 to 41·1)	8·0% (7·0 to 9·1)	−8·9 (−11·9 to −6·0)
	Bahrain	43·6% (39·3 to 47·9)	21·2 (14·4 to 27·7)	30·0% (26·3 to 33·9)	21·4 (17·3 to 25·8)	65·7% (60·2 to 71·1)	34·8 (26·7 to 41·9)	2·0% (1·6 to 2·4)	−3·2 (−4·3 to −2·2)
	Egypt	42·4% (38·2 to 46·4)	26·5 (20·5 to 32·4)	41·4% (37·2 to 45·3)	28·0 (22·7 to 33·3)	85·1% (81·6 to 88·0)	51·1 (43·2 to 59·4)	6·3% (5·1 to 7·7)	−17·2 (−20·6 to −14·0)
	Iran	53·7% (49·8 to 57·6)	18·6 (11·4 to 25·4)	42·1% (38·1 to 46·0)	16·7 (10·5 to 22·7)	73·6% (68·6 to 78·1)	18·9 (10·6 to 26·9)	3·5% (2·7 to 4·4)	−7·8 (−10·2 to −5·4)
	Iraq	35·1% (32·6 to 37·7)	17·6 (12·3 to 22·8)	24·1% (22·1 to 26·1)	17·2 (14·3 to 20·1)	55·1% (52·1 to 58·1)	34·7 (29·2 to 40·3)	8·6% (7·7 to 9·6)	−7·7 (−10·1 to −5·2)
	Jordan	29·3% (26·2 to 32·6)	16·2 (11·3 to 21·2)	20·8% (18·4 to 23·4)	14·5 (11·2 to 17·7)	56·4% (52·9 to 59·9)	39·3 (33·2 to 45·2)	7·5% (6·4 to 8·6)	−16·1 (−19·2 to −13·2)
	Kuwait	45·6% (40·0 to 50·8)	27·2 (19·7 to 34·0)	41·8% (36·8 to 46·8)	30·2 (24·0 to 36·3)	88·5% (86·1 to 90·6)	41·9 (34·1 to 49·4)	1·7% (1·3 to 2·1)	−4·7 (−6·1 to −3·6)
	Lebanon	33·6% (29·4 to 37·9)	11·4 (4·7 to 17·6)	25·4% (22·0 to 29·0)	13·4 (8·8 to 18·0)	63·7% (58·5 to 68·5)	30·2 (21·8 to 38·1)	6·4% (5·1 to 7·8)	−7·4 (−10·3 to −4·5)
	Libya	19·8% (16·2 to 23·7)	6·4 (1·0 to 11·6)	14·2% (11·6 to 17·1)	10·9 (7·8 to 14·0)	48·3% (42·0 to 54·2)	38·3 (31·3 to 44·8)	9·5% (7·9 to 11·1)	−10·0 (−12·9 to −7·1)
	Morocco	37·8% (34·5 to 41·2)	19·0 (12·9 to 24·9)	31·5% (28·5 to 34·4)	20·6 (16·5 to 24·8)	72·2% (69·4 to 74·9)	44·0 (37·1 to 50·9)	5·8% (4·9 to 6·9)	−13·9 (−16·8 to −11·1)
	Oman	24·8% (20·6 to 29·3)	16·6 (11·5 to 21·9)	18·0% (14·9 to 21·5)	15·0 (11·8 to 18·6)	42·9% (37·3 to 48·7)	36·9 (31·2 to 43·0)	17·2% (14·8 to 19·7)	−24·9 (−28·9 to −21·0)
	Palestine	36·4% (34·0 to 38·7)	20·9 (15·6 to 25·6)	27·1% (25·2 to 29·0)	18·6 (14·9 to 21·6)	59·5% (56·8 to 62·0)	37·6 (30·5 to 43·5)	9·1% (8·1 to 10·2)	−13·9 (−17·0 to −10·8)
	Qatar	33·2% (28·0 to 38·4)	21·9 (15·3 to 28·2)	30·2% (25·4 to 35·1)	23·2 (17·8 to 28·7)	61·0% (54·3 to 66·9)	41·6 (32·7 to 49·9)	16·3% (13·7 to 19·1)	−8·5 (−12·3 to −4·5)
	Saudi Arabia	19·9% (16·4 to 23·5)	10·5 (5·4 to 15·2)	18·4% (15·0 to 21·7)	12·8 (8·9 to 16·7)	58·2% (52·0 to 64·3)	40·2 (31·6 to 48·7)	11·7% (10·1 to 13·3)	−10·2 (−13·3 to −7·2)
	Sudan	9·5% (7·3 to 12·0)	6·0 (3·3 to 8·8)	8·8% (6·7 to 11·2)	6·8 (4·4 to 9·2)	31·4% (25·2 to 38·0)	24·9 (18·3 to 31·8)	18·7% (16·8 to 20·6)	−10·2 (−13·4 to −7·0)
	Syria	34·5% (29·9 to 38·6)	21·1 (15·0 to 26·1)	26·2% (22·5 to 29·7)	19·9 (15·7 to 23·8)	62·2% (56·5 to 67·3)	43·9 (36·5 to 51·1)	7·6% (6·1 to 9·4)	−13·4 (−16·4 to −10·4)
	Tunisia	38·3% (35·6 to 40·7)	19·7 (14·3 to 24·6)	33·9% (31·4 to 36·2)	21·7 (17·4 to 25·4)	75·7% (72·9 to 78·4)	42·5 (35·2 to 49·1)	6·5% (5·6 to 7·6)	−11·6 (−14·3 to −8·8)
	Turkey	49·2% (46·5 to 51·8)	20·6 (14·1 to 27·0)	34·8% (32·4 to 37·0)	22·9 (19·1 to 26·5)	62·9% (60·1 to 65·7)	37·3 (31·2 to 43·3)	6·0% (5·1 to 6·9)	−11·4 (−14·4 to −8·5)
	United Arab Emirates	41·1% (33·6 to 47·7)	28·3 (20·4 to 36·2)	38·9% (31·9 to 45·4)	31·8 (24·4 to 38·6)	78·9% (72·5 to 84·4)	58·9 (50·2 to 66·2)	8·1% (6·0 to 10·5)	−14·4 (−18·1 to −10·9)
	Yemen	22·2% (18·5 to 26·1)	18·1 (14·0 to 22·2)	17·9% (14·9 to 21·2)	16·3 (13·2 to 19·8)	44·6% (38·8 to 50·3)	39·7 (33·7 to 46·0)	17·9% (15·7 to 20·1)	−11·4 (−15·0 to −7·9)
**South Asia**		**44·3% (40·8 to 47·6)**	**28·6 (23·7 to 33·0)**	**39·6% (36·4 to 42·6)**	**29·3 (25·6 to 32·9)**	**73·9% (71·1 to 76·5)**	**47·7 (42·5 to 52·6)**	**9·3% (8·0 to 10·6)**	**−14·2 (−16·6 to −11·8)**
	Bangladesh	50·5% (48·1 to 52·7)	34·1 (28·7 to 39·1)	45·1% (42·7 to 47·2)	34·4 (30·5 to 38·0)	74·5% (72·3 to 76·5)	50·0 (43·3 to 56·3)	9·9% (8·9 to 11·0)	−17·2 (−20·5 to −14·0)
	Bhutan	46·6% (42·2 to 50·8)	21·2 (13·8 to 28·4)	46·3% (41·9 to 50·4)	22·7 (15·5 to 29·4)	80·3% (75·2 to 84·8)	37·1 (27·1 to 46·6)	11·1% (8·7 to 13·7)	−18·2 (−23·4 to −13·3)
	India	46·7% (42·2 to 51·0)	30·3 (24·2 to 35·8)	42·3% (38·3 to 46·3)	31·7 (26·8 to 36·5)	76·7% (73·0 to 80·0)	49·2 (43·0 to 55·3)	8·5% (6·8 to 10·2)	−13·7 (−16·7 to −10·7)
	Nepal	37·0% (34·5 to 39·5)	14·8 (8·6 to 20·5)	32·9% (30·5 to 35·2)	14·6 (9·4 to 19·4)	58·1% (55·1 to 60·9)	22·1 (14·2 to 29·6)	19·5% (18·0 to 21·2)	−8·8 (−12·8 to −4·7)
	Pakistan	24·9% (21·9 to 28·0)	18·8 (14·8 to 22·4)	18·9% (16·5 to 21·4)	14·3 (11·1 to 17·1)	50·8% (46·7 to 54·8)	38·4 (32·2 to 44·0)	12·2% (10·8 to 13·8)	−18·7 (−21·9 to −15·3)
**Southeast Asia, east Asia, and Oceania**		**66·9% (65·5 to 68·2)**	**28·7 (25·2 to 32·5)**	**64·7% (63·3 to 65·9)**	**29·6 (26·3 to 33·2)**	**90·4% (89·5 to 91·2)**	**26·7 (22·5 to 31·4)**	**4·6% (4·1 to 5·3)**	**−12·2 (−14·6 to −9·9)**
East Asia		78·5% (76·4 to 80·2)	33·9 (29·1 to 38·8)	77·7% (75·6 to 79·5)	35·3 (30·7 to 40·1)	95·5% (94·4 to 96·4)	24·8 (19·5 to 30·7)	2·9% (2·2 to 3·8)	−12·5 (−15·8 to −9·4)
	China	79·2% (76·9 to 81·0)	34·0 (29·0 to 39·2)	78·4% (76·2 to 80·2)	35·4 (30·7 to 40·4)	95·6% (94·4 to 96·5)	24·7 (19·3 to 30·8)	2·9% (2·1 to 3·8)	−12·6 (−16·1 to −9·4)
	North Korea	51·7% (48·2 to 55·3)	18·0 (10·9 to 25·4)	49·9% (46·4 to 53·3)	19·5 (12·9 to 26·6)	89·8% (87·7 to 91·5)	19·3 (12·7 to 26·6)	3·9% (3·0 to 4·8)	−5·5 (−8·0 to −3·1)
	Taiwan (province of China)	69·1% (66·0 to 71·9)	39·1 (34·1 to 43·8)	68·2% (65·2 to 70·9)	39·8 (35·0 to 44·5)	95·1% (93·9 to 96·1)	34·9 (28·8 to 41·0)	2·6% (1·9 to 3·4)	−14·5 (−17·1 to −11·9)
Oceania		29·9% (28·3 to 31·6)	10·9 (7·5 to 14·1)	25·4% (24·0 to 26·9)	13·1 (10·7 to 15·3)	52·7% (50·5 to 55·1)	26·9 (22·8 to 30·8)	18·4% (17·1 to 19·8)	−10·4 (−13·2 to −7·7)
	American Samoa	41·5% (36·3 to 46·6)	2·3 (−5·4 to 9·4)	40·2% (35·2 to 45·2)	5·8 (−1·5 to 12·4)	79·7% (75·2 to 83·8)	13·2 (6·0 to 20·0)	8·9% (7·2 to 10·7)	−3·6 (−6·6 to −0·8)
	Cook Islands	63·8% (58·3 to 69·2)	32·5 (24·7 to 40·3)	61·2% (55·9 to 66·4)	41·7 (35·0 to 48·5)	86·6% (83·9 to 89·0)	47·7 (40·7 to 54·5)	6·8% (5·5 to 8·5)	−11·9 (−15·1 to −8·4)
	Federated States of Micronesia	27·5% (22·5 to 32·9)	11·4 (4·7 to 18·0)	25·1% (20·4 to 30·1)	14·1 (8·1 to 19·9)	60·2% (52·8 to 67·1)	31·3 (21·2 to 40·4)	14·1% (11·7 to 16·7)	−7·8 (−11·5 to −4·1)
	Fiji	50·8% (45·6 to 55·7)	24·6 (18·2 to 30·6)	47·9% (42·8 to 52·6)	25·8 (19·6 to 31·3)	80·9% (77·1 to 84·4)	29·1 (23·3 to 34·8)	8·4% (6·7 to 10·2)	−8·1 (−10·7 to −5·3)
	Guam	54·0% (48·5 to 59·0)	3·5 (−4·1 to 10·5)	53·2% (47·8 to 58·1)	7·4 (0·3 to 14·2)	87·0% (83·5 to 89·6)	11·4 (6·3 to 16·8)	7·1% (5·7 to 8·7)	−2·9 (−5·5 to −0·3)
	Kiribati	24·8% (21·8 to 27·6)	10·4 (5·3 to 14·9)	20·4% (17·8 to 22·8)	11·9 (8·4 to 15·2)	51·0% (46·6 to 54·9)	27·2 (19·9 to 34·1)	15·2% (13·8 to 16·7)	−6·2 (−9·2 to −3·2)
	Marshall Islands	43·7% (37·8 to 49·6)	19·0 (11·3 to 26·4)	42·3% (36·6 to 48·0)	22·1 (15·2 to 28·9)	88·9% (86·5 to 91·0)	27·0 (20·9 to 33·5)	3·8% (3·0 to 4·7)	−4·1 (−6·1 to −2·2)
	Nauru	35·4% (29·8 to 40·6)	5·9 (−1·7 to 13·3)	27·3% (22·9 to 31·8)	10·8 (4·9 to 16·4)	55·4% (49·9 to 60·6)	21·1 (12·8 to 28·9)	13·8% (11·5 to 16·1)	−5·0 (−8·3 to −1·5)
	Niue	46·1% (40·9 to 51·4)	17·7 (10·4 to 24·8)	44·9% (39·8 to 49·8)	22·3 (15·7 to 28·7)	81·1% (76·9 to 84·7)	32·2 (25·0 to 39·6)	9·2% (7·4 to 11·1)	−8·5 (−11·8 to −5·3)
	Northern Mariana Islands	46·3% (41·1 to 50·9)	7·1 (0·0 to 14·2)	45·4% (40·3 to 49·9)	9·4 (2·5 to 16·1)	84·0% (80·0 to 87·4)	13·2 (7·0 to 19·5)	7·7% (6·1 to 9·4)	−4·0 (−6·6 to −1·2)
	Palau	36·4% (30·6 to 42·5)	17·6 (10·8 to 23·8)	33·4% (28·1 to 38·7)	19·0 (13·0 to 24·7)	71·9% (66·6 to 76·4)	30·1 (21·7 to 38·3)	10·0% (8·2 to 11·8)	−5·5 (−8·2 to −2·8)
	Papua New Guinea	27·9% (26·0 to 29·9)	11·1 (6·0 to 15·6)	23·1% (21·4 to 25·0)	13·9 (10·6 to 16·9)	48·6% (45·7 to 51·7)	30·5 (25·1 to 35·7)	19·6% (18·0 to 21·4)	−14·2 (−18·1 to −10·3)
	Samoa	17·9% (15·2 to 20·7)	7·2 (3·2 to 11·0)	16·3% (13·7 to 18·8)	7·4 (3·8 to 10·8)	41·3% (36·0 to 46·3)	20·8 (13·1 to 27·7)	21·6% (19·7 to 23·8)	−11·3 (−14·8 to −7·9)
	Solomon Islands	23·6% (20·2 to 27·1)	9·5 (4·4 to 14·3)	19·8% (16·9 to 22·8)	10·9 (7·1 to 14·6)	49·8% (44·7 to 54·9)	19·3 (11·6 to 27·2)	16·1% (14·2 to 18·0)	1·2 (−1·4 to 3·8)
	Tokelau	38·4% (33·1 to 43·8)	17·1 (9·7 to 24·1)	37·0% (31·8 to 42·3)	20·8 (14·1 to 27·3)	75·5% (69·7 to 80·0)	36·4 (27·3 to 45·2)	10·5% (8·7 to 12·8)	−9·4 (−12·9 to −5·8)
	Tonga	19·7% (17·4 to 22·4)	−0·3 (−4·4 to 3·5)	16·7% (14·7 to 19·0)	4·0 (0·9 to 7·0)	50·0% (45·9 to 54·6)	16·7 (10·2 to 23·2)	13·6% (12·3 to 14·9)	−4·3 (−7·0 to −2·0)
	Tuvalu	19·5% (16·3 to 22·7)	10·3 (6·0 to 14·4)	17·4% (14·4 to 20·3)	12·7 (9·6 to 16·0)	47·2% (41·5 to 53·1)	33·6 (26·6 to 40·2)	17·3% (15·4 to 19·2)	−7·6 (−10·8 to −4·5)
	Vanuatu	38·7% (33·8 to 43·1)	19·4 (12·6 to 26·0)	33·2% (29·0 to 37·1)	19·3 (14·0 to 24·8)	49·4% (44·4 to 53·6)	21·6 (13·5 to 29·0)	28·4% (25·6 to 31·3)	−2·1 (−6·3 to 2·0)
Southeast Asia		44·4% (42·8 to 45·9)	23·1 (20·3 to 25·6)	39·3% (37·8 to 40·8)	23·6 (21·2 to 25·7)	75·2% (73·5 to 76·5)	37·6 (33·7 to 41·1)	7·9% (7·3 to 8·5)	−12·6 (−14·2 to −11·1)
	Cambodia	36·0% (31·7 to 40·0)	23·4 (17·9 to 29·0)	27·2% (23·6 to 30·5)	19·4 (15·0 to 23·7)	59·8% (54·6 to 64·4)	36·8 (28·9 to 44·3)	9·5% (7·9 to 11·2)	−11·8 (−15·3 to −8·5)
	Indonesia	46·0% (42·7 to 49·2)	25·9 (20·1 to 31·0)	42·9% (39·7 to 45·9)	26·2 (21·1 to 30·7)	78·6% (75·4 to 81·1)	41·1 (33·7 to 48·0)	8·5% (7·3 to 10·0)	−15·7 (−18·8 to −12·4)
	Laos	40·8% (38·2 to 43·4)	23·3 (17·0 to 28·9)	36·9% (34·4 to 39·4)	25·1 (20·5 to 29·5)	68·5% (65·5 to 71·3)	45·7 (38·5 to 52·5)	13·0% (11·6 to 14·5)	−20·9 (−25·1 to −16·8)
	Malaysia	43·3% (38·5 to 47·9)	25·5 (19·7 to 31·0)	40·8% (36·2 to 45·2)	28·2 (23·0 to 33·1)	81·8% (77·5 to 85·7)	50·2 (43·9 to 56·4)	6·5% (4·8 to 8·3)	−15·4 (−18·6 to −12·4)
	Maldives	28·6% (23·2 to 34·3)	13·3 (6·2 to 20·4)	23·2% (18·6 to 27·9)	16·4 (11·2 to 21·7)	45·7% (38·9 to 52·4)	33·9 (26·0 to 41·6)	22·1% (18·9 to 25·2)	−20·3 (−25·5 to −15·1)
	Mauritius	44·5% (41·0 to 47·9)	12·8 (6·3 to 19·5)	26·3% (23·2 to 29·4)	11·0 (6·5 to 15·7)	51·6% (46·9 to 56·7)	18·1 (10·5 to 25·4)	6·4% (5·1 to 7·8)	−7·3 (−10·2 to −4·4)
	Myanmar	29·8% (26·9 to 32·8)	18·0 (13·4 to 22·3)	29·2% (26·2 to 32·1)	20·4 (16·6 to 24·0)	76·0% (71·9 to 79·7)	50·6 (42·7 to 57·8)	8·5% (7·2 to 9·9)	−13·9 (−17·0 to −10·9)
	Philippines	34·2% (31·6 to 36·9)	15·3 (10·2 to 20·3)	25·0% (22·6 to 27·3)	15·6 (12·2 to 18·9)	56·7% (53·0 to 60·4)	32·0 (25·6 to 38·5)	9·9% (8·7 to 11·0)	−9·3 (−12·1 to −6·4)
	Seychelles	44·3% (40·8 to 47·6)	17·3 (10·4 to 23·7)	41·7% (38·3 to 44·9)	22·3 (17·0 to 27·4)	87·3% (84·7 to 89·6)	40·4 (33·3 to 47·7)	3·4% (2·6 to 4·4)	−10·9 (−13·7 to −8·0)
	Sri Lanka	46·6% (42·8 to 49·8)	27·6 (22·2 to 32·5)	38·7% (35·5 to 41·6)	26·8 (22·6 to 30·8)	72·8% (69·4 to 75·8)	40·5 (33·9 to 47·0)	6·5% (5·3 to 7·9)	−11·0 (−13·6 to −8·3)
	Thailand	50·1% (47·4 to 52·5)	26·6 (21·7 to 31·0)	48·5% (46·0 to 50·8)	27·4 (22·9 to 31·6)	85·4% (83·2 to 87·3)	33·2 (26·3 to 40·3)	6·7% (5·7 to 7·8)	−10·2 (−12·8 to −7·8)
	Timor-Leste	15·9% (13·1 to 19·2)	7·2 (3·0 to 11·2)	14·6% (12·0 to 17·5)	10·1 (7·0 to 13·2)	48·9% (42·3 to 55·3)	33·6 (25·9 to 41·4)	14·0% (12·3 to 15·8)	−6·9 (−9·9 to −3·9)
	Vietnam	57·4% (54·1 to 60·3)	22·9 (16·4 to 29·2)	46·8% (43·5 to 50·0)	24·2 (18·7 to 29·1)	76·0% (72·3 to 79·1)	27·4 (20·1 to 34·3)	4·3% (3·3 to 5·3)	−7·9 (−10·8 to −5·2)
**Sub-Saharan Africa**		**27·4% (26·8 to 28·1)**	**16·8 (15·9 to 17·8)**	**23·6% (23·1 to 24·2)**	**17·2 (16·4 to 18·0)**	**52·0% (51·2 to 52·8)**	**34·8 (33·6 to 36·1)**	**18·0% (17·6 to 18·4)**	**−8·7 (−9·5 to −7·9)**
Central sub-Saharan Africa		25·8% (23·7 to 28·0)	15·3 (12·3 to 18·0)	15·9% (14·6 to 17·3)	13·8 (12·5 to 15·2)	32·1% (30·1 to 34·2)	27·1 (24·9 to 29·4)	23·6% (22·4 to 24·9)	−6·9 (−9·1 to −4·6)
	Angola	15·1% (13·0 to 17·5)	8·5 (5·7 to 11·2)	13·9% (11·9 to 16·0)	11·0 (8·9 to 13·3)	33·2% (29·4 to 37·3)	26·4 (22·3 to 30·9)	26·7% (24·7 to 28·7)	−9·1 (−12·7 to −5·6)
	Central African Republic	17·1% (14·7 to 19·6)	5·6 (1·6 to 9·3)	11·0% (9·4 to 12·8)	8·0 (6·1 to 10·0)	23·8% (20·7 to 27·2)	15·4 (11·4 to 19·4)	29·2% (27·0 to 31·3)	5·1 (1·8 to 8·3)
	Congo (Brazzaville)	38·0% (33·6 to 42·4)	23·0 (17·0 to 28·4)	24·1% (21·1 to 27·5)	22·4 (19·4 to 25·6)	43·7% (39·9 to 47·8)	39·6 (35·7 to 43·6)	17·1% (15·3 to 19·1)	−11·0 (−14·3 to −7·9)
	Democratic Republic of the Congo	29·2% (26·2 to 32·3)	18·1 (13·8 to 22·0)	16·1% (14·2 to 18·0)	14·3 (12·3 to 16·2)	30·9% (28·3 to 33·7)	26·5 (23·6 to 29·4)	22·7% (20·9 to 24·5)	−7·1 (−10·2 to −3·7)
	Equatorial Guinea	18·4% (15·1 to 21·9)	15·8 (12·6 to 19·4)	16·1% (13·3 to 19·1)	15·3 (12·5 to 18·4)	38·5% (33·6 to 43·5)	36·7 (31·5 to 41·7)	23·3% (21·4 to 25·5)	−13·7 (−17·4 to −9·9)
	Gabon	37·5% (33·0 to 42·4)	22·0 (16·1 to 28·0)	28·0% (24·7 to 31·7)	25·7 (22·2 to 29·6)	49·2% (45·1 to 53·3)	44·4 (40·2 to 48·8)	19·4% (17·2 to 21·5)	−12·8 (−16·7 to −9·2)
Eastern sub-Saharan Africa		30·8% (29·8 to 31·9)	21·3 (20·0 to 22·7)	28·2% (27·3 to 29·2)	23·1 (22·0 to 24·2)	59·6% (58·4 to 61·0)	45·8 (43·8 to 47·6)	16·5% (15·9 to 17·0)	−11·0 (−12·2 to −9·9)
	Burundi	16·9% (14·4 to 19·7)	10·3 (7·0 to 13·7)	14·0% (11·8 to 16·3)	11·5 (9·2 to 13·9)	39·8% (35·1 to 44·7)	31·1 (25·7 to 36·7)	18·2% (16·7 to 19·8)	−3·5 (−6·3 to −0·8)
	Comoros	19·3% (15·7 to 23·2)	8·6 (3·9 to 13·5)	15·9% (12·8 to 19·0)	12·5 (9·4 to 16·0)	40·7% (34·7 to 46·5)	33·0 (26·7 to 39·2)	19·6% (17·3 to 21·7)	−12·8 (−16·6 to −9·1)
	Djibouti	17·2% (14·2 to 20·7)	6·1 (1·8 to 10·4)	16·4% (13·5 to 19·8)	8·4 (4·7 to 12·3)	59·0% (52·7 to 64·8)	28·1 (18·9 to 36·0)	10·6% (9·2 to 12·0)	−4·2 (−6·5 to −2·0)
	Eritrea	8·2% (6·3 to 10·6)	4·0 (1·6 to 6·7)	6·5% (5·0 to 8·5)	5·3 (3·7 to 7·2)	26·7% (21·3 to 32·9)	22·5 (17·1 to 28·8)	16·3% (14·5 to 18·1)	−9·3 (−12·5 to −6·0)
	Ethiopia	27·8% (25·8 to 30·0)	20·6 (17·4 to 23·5)	26·6% (24·6 to 28·7)	21·7 (19·1 to 24·2)	62·0% (58·8 to 65·3)	48·1 (42·8 to 52·9)	15·0% (13·8 to 16·3)	−12·7 (−15·7 to −9·9)
	Kenya	45·0% (42·3 to 47·7)	34·6 (30·3 to 38·1)	42·9% (40·2 to 45·4)	37·0 (33·7 to 40·0)	74·7% (72·2 to 77·3)	61·1 (56·5 to 65·4)	12·4% (11·1 to 13·7)	−20·4 (−23·4 to −17·3)
	Madagascar	35·1% (32·0 to 38·6)	20·3 (15·1 to 25·6)	31·4% (28·5 to 34·4)	26·4 (23·1 to 29·9)	61·2% (57·6 to 64·7)	48·1 (42·8 to 53·2)	16·1% (14·6 to 17·7)	−6·7 (−9·7 to −3·8)
	Malawi	43·4% (39·2 to 48·0)	27·2 (20·6 to 33·1)	42·2% (38·1 to 46·6)	30·1 (24·5 to 35·2)	72·6% (68·0 to 76·9)	46·6 (38·9 to 53·9)	14·6% (12·5 to 16·9)	−15·6 (−19·4 to −11·7)
	Mozambique	26·0% (22·3 to 29·8)	16·4 (12·0 to 21·0)	24·6% (21·1 to 28·2)	17·8 (14·0 to 21·9)	53·4% (48·1 to 58·3)	33·2 (26·3 to 39·6)	20·0% (18·2 to 21·9)	−3·7 (−6·8 to −0·7)
	Rwanda	34·2% (31·1 to 37·5)	25·6 (21·1 to 29·8)	30·7% (27·9 to 33·7)	28·5 (25·5 to 31·7)	68·1% (64·5 to 71·9)	62·6 (58·6 to 66·6)	10·8% (9·3 to 12·2)	−19·3 (−22·3 to −16·3)
	Somalia	6·0% (4·9 to 7·2)	0·0 (−2·4 to 2·1)	1·9% (1·5 to 2·4)	0·0 (−0·9 to 0·7)	6·9% (5·5 to 8·7)	−0·8 (−3·9 to 1·8)	22·1% (20·6 to 23·7)	2·5 (−0·2 to 5·3)
	South Sudan	3·7% (2·8 to 4·9)	−0·1 (−1·8 to 1·5)	1·9% (1·4 to 2·5)	0·7 (0·1 to 1·4)	4·8% (3·6 to 6·3)	2·3 (0·8 to 4·0)	35·1% (32·6 to 38·0)	−7·0 (−11·4 to −2·2)
	Tanzania	33·9% (29·9 to 37·9)	22·4 (17·4 to 27·4)	28·9% (25·5 to 32·2)	23·4 (19·6 to 27·2)	57·9% (53·8 to 61·6)	43·0 (37·5 to 48·2)	15·9% (14·4 to 17·6)	−9·2 (−12·3 to −6·2)
	Uganda	33·6% (31·2 to 36·1)	24·7 (21·1 to 28·1)	30·1% (27·9 to 32·3)	25·8 (23·4 to 28·4)	55·8% (52·9 to 58·9)	44·5 (40·2 to 48·5)	20·3% (18·7 to 21·8)	−8·1 (−11·2 to −4·9)
	Zambia	35·3% (32·4 to 38·2)	21·1 (16·4 to 25·5)	33·1% (30·4 to 35·8)	25·6 (22·1 to 28·8)	65·2% (62·1 to 68·4)	47·4 (42·1 to 52·4)	15·3% (14·0 to 16·7)	−12·2 (−15·5 to −9·3)
Southern sub-Saharan Africa		50·8% (48·4 to 53·0)	19·0 (13·9 to 23·7)	50·1% (47·7 to 52·4)	21·0 (16·2 to 25·4)	81·9% (80·0 to 83·6)	24·3 (19·5 to 29·2)	10·4% (9·5 to 11·4)	−8·4 (−10·4 to −6·4)
	Botswana	62·6% (58·1 to 67·2)	48·5 (42·9 to 53·8)	62·1% (57·6 to 66·7)	50·8 (45·8 to 55·7)	87·9% (85·4 to 90·3)	56·1 (49·4 to 62·2)	8·0% (6·6 to 9·4)	−13·4 (−16·2 to −10·5)
	eSwatini	52·2% (48·4 to 56·2)	38·3 (32·8 to 43·2)	51·2% (47·5 to 55·1)	41·5 (36·8 to 45·9)	81·3% (78·3 to 84·2)	62·6 (56·7 to 67·8)	10·8% (9·3 to 12·5)	−27·3 (−31·1 to −23·6)
	Lesotho	51·9% (49·1 to 55·0)	43·7 (40·0 to 47·5)	51·1% (48·3 to 54·1)	45·1 (41·8 to 48·4)	80·5% (78·0 to 82·7)	64·9 (60·0 to 69·4)	11·6% (10·3 to 13·0)	−18·7 (−21·6 to −15·5)
	Namibia	52·4% (48·3 to 56·3)	28·4 (22·2 to 34·5)	51·8% (47·7 to 55·7)	31·5 (25·8 to 37·1)	81·7% (78·7 to 84·3)	35·5 (29·3 to 42·2)	11·0% (9·6 to 12·6)	−9·0 (−11·9 to −6·1)
	South Africa	50·6% (47·3 to 53·6)	15·2 (8·5 to 21·3)	50·1% (46·8 to 53·0)	16·9 (10·5 to 22·8)	81·1% (78·6 to 83·3)	18·7 (12·5 to 25·2)	11·1% (10·0 to 12·4)	−6·5 (−9·3 to −3·8)
	Zimbabwe	49·0% (45·2 to 52·6)	23·8 (17·5 to 30·2)	48·0% (44·3 to 51·5)	26·9 (21·2 to 32·6)	84·3% (81·6 to 86·8)	37·1 (30·3 to 45·2)	7·9% (6·6 to 9·2)	−11·5 (−14·6 to −8·8)
Western sub-Saharan Africa		20·3% (19·5 to 21·3)	14·6 (13·5 to 15·7)	16·5% (15·9 to 17·3)	14·0 (13·3 to 14·8)	41·8% (40·4 to 43·2)	34·0 (32·4 to 35·8)	19·3% (18·6 to 20·0)	−7·8 (−9·2 to −6·5)
	Benin	16·4% (14·4 to 19·0)	8·3 (5·0 to 11·4)	12·5% (10·8 to 14·5)	10·7 (8·8 to 12·9)	30·0% (26·4 to 33·6)	25·0 (21·3 to 28·9)	25·3% (23·7 to 27·2)	−3·6 (−7·1 to −0·5)
	Burkina Faso	28·8% (26·3 to 31·2)	18·9 (15·2 to 22·4)	27·2% (24·9 to 29·5)	21·9 (18·9 to 24·7)	54·9% (51·7 to 58·3)	41·6 (36·8 to 46·2)	20·8% (19·2 to 22·3)	−9·3 (−12·5 to −6·2)
	Cameroon	26·0% (23·0 to 29·0)	19·6 (16·1 to 23·1)	18·7% (16·4 to 21·2)	17·6 (15·1 to 20·0)	44·1% (40·7 to 47·6)	41·4 (37·6 to 45·3)	16·4% (15·1 to 17·6)	−19·5 (−22·6 to −16·4)
	Cape Verde	54·5% (49·4 to 59·4)	31·2 (24·7 to 37·8)	52·9% (48·0 to 57·7)	34·3 (28·7 to 40·3)	80·4% (76·4 to 83·8)	41·4 (34·1 to 48·5)	11·2% (9·3 to 13·5)	−12·9 (−16·7 to −9·0)
	Chad	6·6% (5·7 to 7·6)	2·0 (0·1 to 3·6)	4·5% (3·8 to 5·2)	3·4 (2·7 to 4·2)	14·3% (12·3 to 16·4)	10·0 (7·6 to 12·5)	24·8% (22·9 to 26·6)	4·3 (1·3 to 7·3)
	Côte d'Ivoire	24·0% (21·9 to 26·3)	19·4 (17·0 to 22·0)	20·2% (18·5 to 22·2)	18·8 (17·1 to 20·8)	42·2% (39·2 to 45·3)	39·0 (35·7 to 42·2)	23·9% (22·4 to 25·5)	−14·4 (−17·7 to −11·0)
	The Gambia	12·8% (11·7 to 13·9)	4·8 (1·9 to 7·3)	11·7% (10·8 to 12·8)	6·5 (4·3 to 8·3)	38·3% (35·6 to 41·1)	23·1 (17·8 to 28·1)	17·9% (16·7 to 19·1)	−8·8 (−11·7 to −6·1)
	Ghana	26·4% (24·0 to 28·9)	18·0 (14·6 to 21·4)	22·4% (20·4 to 24·6)	18·3 (15·9 to 20·9)	45·9% (42·9 to 49·2)	36·3 (32·2 to 40·6)	22·3% (20·8 to 23·8)	−11·9 (−15·2 to −8·5)
	Guinea	11·9% (10·6 to 13·3)	7·7 (5·9 to 9·4)	8·9% (7·9 to 10·1)	7·5 (6·4 to 8·7)	26·1% (23·6 to 29·1)	21·1 (18·2 to 24·2)	22·2% (20·8 to 23·6)	−1·7 (−4·5 to 1·0)
	Guinea-Bissau	30·4% (28·0 to 32·9)	17·6 (13·6 to 21·6)	26·1% (23·9 to 28·4)	19·0 (16·0 to 21·9)	51·9% (49·2 to 55·0)	34·3 (29·3 to 39·0)	19·9% (18·4 to 21·3)	−7·9 (−11·1 to −4·6)
	Liberia	25·6% (23·5 to 27·5)	15·4 (12·4 to 18·3)	24·4% (22·5 to 26·2)	16·6 (13·9 to 19·1)	45·9% (43·2 to 48·6)	29·2 (24·5 to 33·5)	27·5% (25·8 to 29·3)	−8·7 (−12·0 to −5·3)
	Mali	15·9% (14·4 to 17·6)	11·2 (8·9 to 13·5)	15·1% (13·6 to 16·7)	12·6 (10·8 to 14·4)	38·6% (35·2 to 41·8)	31·4 (27·2 to 35·2)	23·1% (21·4 to 24·9)	−6·8 (−10·4 to −3·7)
	Mauritania	11·3% (9·6 to 13·2)	9·5 (7·7 to 11·6)	9·9% (8·4 to 11·6)	9·0 (7·5 to 10·8)	28·8% (24·7 to 33·1)	25·6 (21·5 to 29·9)	23·2% (21·5 to 24·9)	−3·9 (−7·2 to −0·7)
	Niger	18·1% (15·6 to 21·1)	10·1 (6·1 to 13·7)	16·6% (14·3 to 19·2)	12·7 (10·0 to 15·6)	49·6% (45·0 to 54·3)	34·6 (27·7 to 40·7)	15·3% (14·0 to 16·7)	−2·4 (−5·0 to 0·0)
	Nigeria	19·2% (17·4 to 20·9)	14·7 (12·5 to 16·8)	14·6% (13·2 to 16·0)	12·5 (11·1 to 14·0)	40·0% (37·2 to 42·8)	32·4 (28·8 to 35·8)	17·2% (15·9 to 18·6)	−4·8 (−7·4 to −2·0)
	São Tomé and Príncipe	37·7% (35·0 to 40·5)	22·2 (17·5 to 26·4)	35·6% (33·1 to 38·2)	23·7 (19·8 to 27·4)	59·0% (56·1 to 61·8)	35·6 (29·6 to 41·6)	22·6% (21·0 to 24·3)	−12·7 (−16·1 to −9·2)
	Senegal	19·6% (18·5 to 20·8)	16·3 (14·7 to 17·9)	18·4% (17·3 to 19·5)	16·6 (15·4 to 17·8)	52·8% (50·4 to 55·2)	48·8 (45·9 to 51·4)	15·2% (14·3 to 16·2)	−24·9 (−27·9 to −21·6)
	Sierra Leone	25·9% (23·9 to 28·2)	19·7 (17·0 to 22·3)	24·9% (23·0 to 27·0)	20·5 (18·2 to 22·7)	51·5% (48·6 to 54·4)	39·5 (35·0 to 43·6)	22·5% (21·1 to 24·0)	−8·0 (−11·2 to −4·7)
	Togo	23·4% (20·5 to 26·2)	10·7 (6·2 to 14·9)	21·0% (18·4 to 23·7)	17·7 (14·9 to 20·4)	42·3% (38·4 to 46·1)	35·9 (31·7 to 40·1)	26·2% (24·4 to 28·0)	−11·6 (−15·2 to −8·2)

Numbers in parentheses are 95% uncertainty intervals. CPR=contraceptive prevalence rate. mCPR=modern contraceptive prevalence rate. SDI=Socio-demographic Index.

* Absolute change in percentage points.

Between 1970 and 2019, family planning indicators improved substantially worldwide (table 1). Globally, substantial increases were observed in contraceptive prevalence (18·7 percentage point increase [95% UI 17·1–20·3]), the mCPR (20·1 [18·7–21·6]), and demand satisfied with modern methods (24·3 [22·6–26·1]). Statistically significant increases were observed across all countries between 1970 and 2019, with the exception of Somalia, where the mCPR in 2019 remained effectively unchanged when compared with 1970. The largest increases in the mCPR were observed in Botswana, Lesotho, and Paraguay over this period. Increases in the mCPR were largest in Latin America and the Caribbean (31·2 [28·6–33·5]) followed by southeast Asia, east Asia, and Oceania (29·6 [26·3–33·2]), and south Asia (29·3 [25·6–32·9]).

Despite the substantial improvements in the mCPR and demand satisfied over the past 50 years, in 2019, of 1·176 billion women (95% UI 1·163–1·189) worldwide who had a need for contraception, an estimated 162·9 million women (155·6–170·2) remained with unmet need. Among women with unmet need globally, 29·3% (27·4–30·6) were located in sub-Saharan Africa and 27·2% (24·4–30·3) in south Asia. Unmet need, as a proportion of all women of reproductive age, was largest in South Sudan (35·1% [32·6–38·0]), Central African Republic (29·2% [27·0–31·3]), and Vanuatu (28·4% [25·6–31·3]) in 2019 (table 1). The Family Planning 2020 Initiative (FP2020) set a goal of increasing the number of women using modern contraception by 120 million between 2012 and 2020 in 69 priority countries.^16^ We estimated that the number of women using contraception increased by 69·0 million (51·3–85·9) between 2012 and 2019 in these countries (excluding Western Sahara), leaving the initiative 51·0 million (34·2–68·7) short of reaching its goal if these amounts remained unchanged in 2020. Our estimate of additional users is higher than the estimates of the FP2020 initiative (51·1 million additional users by 2019).^51^ We found that the goals set by the initiative for annual rates of change in mCPR were met in just 5% of country-years in ten countries (including rates of change within 0·2 percentage points). However, compared with the previous 7-year period (2004–2011), mean rates of mCPR growth increased in ten FP2020 countries and were not statistically different from the previous time period in an additional 51 countries (appendix pp 48–50).

### Demand satisfied with modern methods (SDG 3.7.1) by age group and marital status

**Figure 2:**
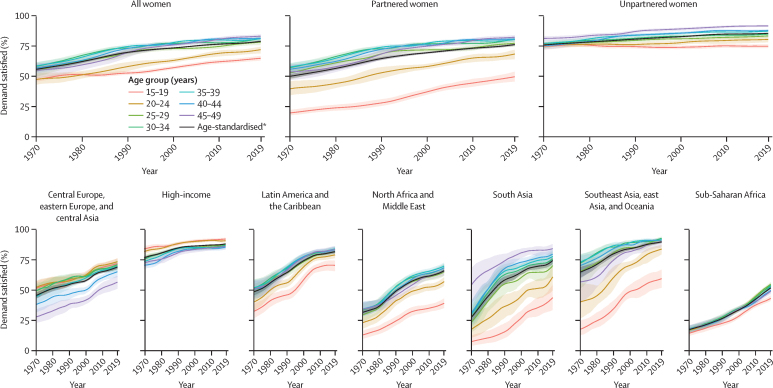
Demand satisfied with modern contraceptive methods, by marital status, age group, and super-region, 1970–2019 *Age-standardised represents the aggregated estimates for ages 15–49 years using a standard age structure for all locations.

Trends in and levels of demand satisfied differed by age and marital status (figure 2). Globally, demand satisfied increased from 54·9% (95% UI 53·2–56·5) in 1970 to 79·1% (78·4–79·8) in 2019 (table 1). Among the 162·9 million women (155·6–170·2) of reproductive age with unmet need, 29·3% (27·9–30·6) resided in sub-Saharan Africa and 27·2% (24·4–30·3) in south Asia. South Asia, southeast Asia, east Asia, and Oceania, and north Africa and the Middle East had the biggest gaps in demand satisfied across ages, but also some of the largest increases in meeting the contraceptive needs of women of reproductive age between 1970 and 2019.

The lowest rates of demand satisfied were among women aged 15–19 years (64·8% [62·9–66·7]) and aged 20–24 years (71·9% [68·9–74·2]) when compared with women of other age groups (figure 2). Women aged 15–24 years accounted for 16·0% of total need but 26·5% of global unmet need, equating to 43 million women worldwide. Furthermore, demand satisfied among women aged 15–24 years remained substantially lower than among women aged 25–49 years (figure 2), with the majority of unmet need concentrated among partnered women in that age group. This difference in unmet need is related to the definition of contraception need: partnered women were considered to have need if they did not desire children, were fecund, and were not breastfeeding or pregnant, whereas unpartnered women were considered to have need if they had been sexually active in the past 4 weeks, in addition to the other criteria.

### Mix of contraceptive methods by age, marital status, super-region, and location

**Figure 3:**
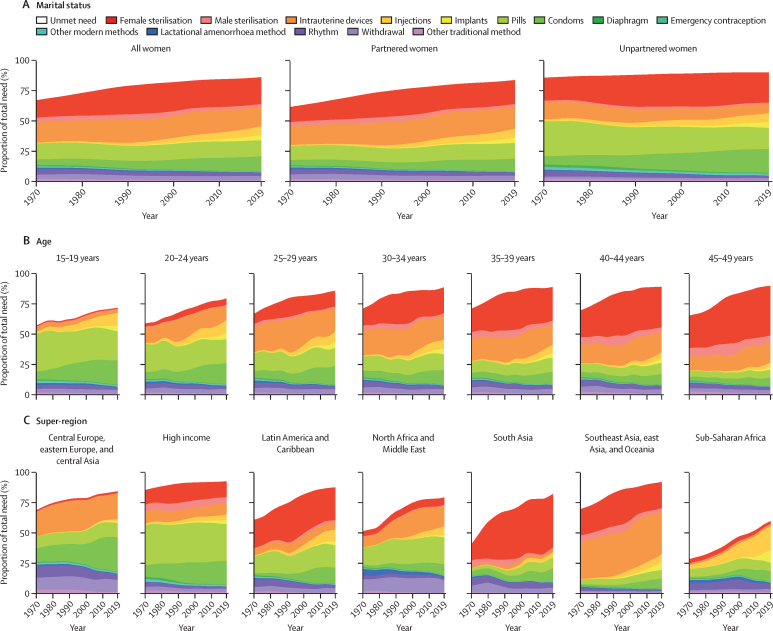
Total need for family planning and mix of contraceptive methods, by marital status (A), age group (B), and super-region (C), 1970–2019

The methods women of reproductive age use to meet their contraception needs differed substantially by marital status, age, and region (figure 3). Globally, between 1970 and 2019, the use of less effective traditional methods such as withdrawal, rhythm, and lactational amenorrhoea method have declined, while the use of condoms, implants, injections, female sterilisation, and other modern methods have increased. Compared with partnered women, unpartnered women more commonly used the oral contraceptive pill and condoms, and less commonly used intrauterine devices. Between 1970 and 2019, condoms and the pill were the most common contraceptive methods among adolescents aged 15–19 years, whereas long-acting reversible methods tended to be most common among women aged 20–49 years. Female sterilisation was more common in older age groups than younger age groups.

The most common method of contraception also differed substantially by super-region (figure 3). South Asia and 28 countries rely on one method for nearly 50% of contraception in use (table 2). Two methods are dominant in Latin America and the Caribbean (female sterilisation and the pill), high-income countries (the pill and condoms), and central Europe, eastern Europe, and central Asia (intrauterine devices and condoms). In 2019, prevalence of female sterilisation was highest in south Asia (53·1% of all methods in use [95% UI 49·6–56·2]), followed by Latin America and the Caribbean (31·6% [29·4–33·6]). The use of contraceptive injections increased substantially in sub-Saharan Africa between 1970 and 2019, and the use of implants increased in both sub-Saharan Africa and south Asia since 2000 (figure 3).

**Table 2:** Contraceptive methods in use as proportion of total contraceptive prevalence, 2019

		**Female sterilisation**	**Male sterilisation**	**Intrauterine devices**	**Injections**	**Implants**	**Pills**	**Condoms**	Other modern methods*	**Lactational amenorrhoea method**	**Rhythm**	**Withdrawal**	**Other traditional methods**
**Global**		**26·6% (24·8–28·2)**	**2·8% (2·4–3·4)**	**19·4% (17·9–20·9)**	**8·2% (8·0–8·5)**	**4·9% (4·3–5·7)**	**14·8% (14·3–15·4)**	**14·0% (13·3–14·9)**	**1·0% (0·9–1·0)**	**0·4% (0·4–0·4)**	**3·0% (2·8–3·2)**	**4·1% (3·9–4·4)**	**0·6% (0·6–0·6)**
	Low SDI	5·2% (4·8–5·5)	1·1% (1·0–1·2)	2·8% (2·6–3·0)	35·8% (34·8–36·8)	17·1% (16·5–17·7)	11·6% (11·1–12·2)	9·9% (9·4–10·4)	1·6% (1·5–1·6)	2·2% (2·0–2·4)	7·1% (6·7–7·6)	4·4% (4·1–4·8)	1·3% (1·2–1·4)
	Low-middle SDI	42·9% (40·0–45·3)	1·1% (0·9–1·4)	6·5% (5·9–7·2)	8·2% (7·6–8·8)	2·8% (2·5–3·0)	13·5% (12·3–14·9)	13·5% (11·8–15·3)	0·9% (0·8–0·9)	0·5% (0·4–0·5)	4·9% (4·3–5·6)	4·9% (4·3–5·6)	0·5% (0·5–0·6)
	Middle SDI	19·1% (18·0–20·2)	1·1% (0·9–1·3)	12·2% (11·5–12·9)	18·4% (17·4–19·5)	4·4% (3·9–4·9)	22·2% (21·0–23·6)	11·6% (10·9–12·4)	0·9% (0·8–0·9)	0·4% (0·4–0·4)	3·6% (3·3–3·9)	5·6% (5·2–6·1)	0·4% (0·4–0·5)
	High-middle SDI	24·3% (20·7–28·2)	3·8% (2·7–5·2)	36·7% (32·7–40·5)	1·6% (1·4–1·9)	5·7% (4·0–7·6)	8·1% (7·4–9·0)	13·9% (12·4–15·7)	0·9% (0·8–0·9)	0·2% (0·2–0·2)	1·2% (1·1–1·4)	3·2% (3·0–3·5)	0·5% (0·4–0·6)
	High SDI	17·4% (16·3–18·6)	6·5% (5·8–7·3)	11·1% (10·4–11·8)	5·2% (4·7–5·7)	3·9% (3·6–4·3)	27·9% (26·8–29·2)	19·8% (18·9–20·9)	1·5% (1·4–1·6)	0·1% (0·1–0·1)	2·2% (2·0–2·5)	3·3% (3·0–3·7)	1·0% (0·9–1·2)
**Central Europe, eastern Europe, and central Asia**		**2·1% (1·9–2·4)**	**0·3% (0·3–0·3)**	**26·5% (24·6–28·7)**	**1·9% (1·6–2·3)**	**1·4% (1·2–1·6)**	**13·7% (12·5–15·1)**	**32·4% (30·5–34·4)**	**1·6% (1·5–1·8)**	**0·5% (0·5–0·6)**	**6·0% (5·3–6·8)**	**12·1% (11·1–13·1)**	**1·3% (1·1–1·7)**
Central Asia		2·7% (2·2–3·3)	0·3% (0·3–0·4)	55·5% (52·9–57·8)	5·4% (3·9–7·2)	1·1% (0·9–1·4)	6·8% (5·9–7·6)	12·5% (11·5–13·5)	1·0% (0·9–1·0)	1·2% (1·0–1·5)	2·5% (2·2–2·9)	10·5% (9·5–11·5)	0·6% (0·5–0·6)
	Armenia	1·2% (0·8–1·8)	0·2% (0·1–0·3)	16·4% (13·2–20·1)	0·6% (0·4–0·9)	1·6% (1·0–2·4)	4·0% (2·9–5·2)	26·1% (21·9–30·9)	0·8% (0·7–0·9)	0·8% (0·6–1·1)	5·8% (4·5–7·5)	41·6% (36·0–47·0)	0·9% (0·7–1·3)
	Azerbaijan	1·3% (0·8–1·9)	0·2% (0·2–0·3)	15·8% (11·7–20·5)	1·0% (0·6–1·6)	1·5% (1·0–2·3)	2·9% (1·9–4·4)	6·4% (4·8–8·6)	1·1% (0·9–1·3)	1·1% (0·8–1·6)	6·2% (4·4–8·2)	61·4% (55·5–66·6)	0·9% (0·6–1·3)
	Georgia	7·0% (5·4–9·0)	1·4% (1·0–1·9)	19·5% (16·2–23·5)	0·5% (0·3–0·8)	0·8% (0·6–1·2)	11·6% (9·3–14·1)	30·1% (26·6–33·9)	2·9% (2·5–3·3)	2·4% (1·8–3·3)	14·4% (11·3–17·8)	8·0% (6·0–10·5)	1·4% (1·1–1·8)
	Kazakhstan	2·7% (2·0–3·6)	0·3% (0·2–0·3)	48·7% (44·8–52·3)	0·6% (0·4–0·9)	0·4% (0·3–0·6)	14·3% (11·9–16·7)	26·2% (23·3–29·3)	1·1% (1·0–1·2)	1·2% (0·9–1·6)	1·6% (1·2–2·0)	1·9% (1·5–2·5)	0·9% (0·7–1·2)
	Kyrgyzstan	3·0% (2·2–4·1)	0·4% (0·3–0·5)	48·1% (43·8–52·7)	1·4% (0·9–2·0)	0·7% (0·5–1·1)	8·1% (6·4–10·3)	31·1% (27·4–34·8)	0·9% (0·8–1·1)	1·4% (1·1–2·0)	1·1% (0·8–1·4)	3·3% (2·5–4·4)	0·4% (0·3–0·6)
	Mongolia	5·5% (4·6–6·7)	0·4% (0·3–0·5)	48·4% (44·6–52·2)	8·5% (6·5–11·1)	4·5% (3·7–5·5)	14·0% (11·7–16·6)	11·1% (9·4–13·0)	1·1% (0·9–1·2)	0·4% (0·3–0·6)	5·1% (4·0–6·5)	0·5% (0·4–0·7)	0·5% (0·4–0·6)
	Tajikistan	2·3% (1·6–3·2)	0·8% (0·5–1·1)	60·1% (55·5–64·5)	6·9% (4·7–9·7)	0·8% (0·5–1·1)	6·9% (5·2–9·2)	11·1% (8·9–13·6)	1·5% (1·3–1·8)	1·8% (1·3–2·6)	0·6% (0·4–0·8)	6·9% (5·3–8·8)	0·3% (0·2–0·5)
	Turkmenistan	0·9% (0·7–1·2)	0·4% (0·3–0·5)	81·6% (79·7–83·5)	1·3% (0·9–1·7)	1·7% (1·2–2·4)	2·1% (1·6–2·6)	3·3% (2·6–4·1)	1·3% (1·1–1·5)	2·2% (1·5–3·0)	1·1% (0·8–1·4)	3·9% (2·9–5·1)	0·4% (0·3–0·5)
	Uzbekistan	2·9% (1·9–4·3)	0·2% (0·2–0·3)	70·6% (65·8–74·7)	9·5% (6·3–13·5)	1·1% (0·7–1·6)	4·0% (2·8–5·6)	5·1% (3·9–6·7)	0·7% (0·6–0·8)	1·1% (0·7–1·6)	1·5% (1·1–2·1)	2·9% (2·1–4·0)	0·3% (0·2–0·4)
Central Europe		3·3% (2·8–3·8)	0·4% (0·3–0·4)	7·6% (6·7–8·4)	1·2% (1·0–1·4)	1·2% (1·1–1·4)	20·5% (18·9–22·4)	36·8% (34·7–38·9)	2·2% (2·1–2·5)	0·3% (0·3–0·4)	8·7% (7·7–9·9)	17·3% (16·2–18·4)	0·5% (0·4–0·6)
	Albania	2·9% (2·0–4·3)	0·3% (0·2–0·4)	0·9% (0·6–1·3)	1·3% (0·8–1·9)	0·4% (0·3–0·5)	3·4% (2·4–4·8)	8·1% (6·1–10·4)	0·8% (0·6–0·9)	1·4% (0·9–2·0)	1·1% (0·8–1·5)	79·0% (75·3–82·2)	0·4% (0·3–0·5)
	Bosnia and Herzegovina	0·5% (0·3–0·7)	0·3% (0·2–0·4)	8·4% (5·8–11·7)	0·8% (0·5–1·3)	0·8% (0·6–1·1)	7·0% (5·3–9·3)	22·1% (17·9–26·8)	1·0% (0·8–1·1)	0·4% (0·3–0·5)	6·0% (4·2–8·1)	52·1% (46·1–58·0)	0·5% (0·4–0·7)
	Bulgaria	1·0% (0·7–1·5)	0·3% (0·2–0·3)	12·0% (8·9–15·9)	0·9% (0·6–1·3)	0·6% (0·5–0·9)	11·6% (8·8–14·7)	38·4% (33·3–43·5)	0·9% (0·8–1·0)	0·3% (0·2–0·4)	4·4% (3·3–6·1)	29·1% (24·3–34·5)	0·4% (0·3–0·5)
	Croatia	1·6% (1·1–2·4)	0·4% (0·3–0·5)	6·1% (4·2–8·6)	2·0% (1·3–3·0)	1·6% (1·2–2·2)	11·4% (8·5–14·7)	39·7% (34·1–45·9)	1·5% (1·3–1·8)	0·4% (0·3–0·5)	4·6% (3·4–6·5)	30·2% (24·3–36·3)	0·5% (0·4–0·7)
	Czechia	7·6% (5·3–10·6)	0·3% (0·2–0·4)	11·4% (8·4–14·8)	2·6% (1·9–3·6)	1·0% (0·7–1·3)	29·1% (24·3–34·0)	34·1% (29·7–39·0)	1·0% (0·8–1·1)	0·2% (0·2–0·3)	1·3% (1·0–1·9)	10·3% (7·9–13·2)	1·2% (0·9–1·8)
	Hungary	6·6% (4·7–9·2)	0·4% (0·3–0·5)	14·8% (11·3–18·7)	1·6% (1·2–2·3)	1·6% (1·2–2·0)	29·2% (24·7–33·9)	31·5% (26·8–36·1)	3·0% (2·6–3·4)	0·3% (0·2–0·4)	3·2% (2·4–4·2)	7·1% (5·6–9·1)	0·7% (0·5–0·8)
	Montenegro	1·1% (0·7–1·6)	0·6% (0·4–0·8)	14·3% (10·7–18·9)	3·0% (2·0–4·5)	1·0% (0·7–1·4)	10·3% (7·8–13·5)	38·3% (32·8–43·8)	3·0% (2·5–3·5)	0·4% (0·3–0·5)	3·7% (2·7–5·1)	22·9% (18·0–28·3)	1·5% (1·1–2·0)
	North Macedonia	1·9% (1·3–2·6)	0·3% (0·2–0·4)	2·3% (1·6–3·1)	0·6% (0·4–0·8)	0·9% (0·6–1·2)	3·3% (2·5–4·2)	26·5% (23·3–29·6)	1·3% (1·1–1·5)	0·4% (0·3–0·6)	2·5% (1·9–3·3)	59·7% (56·1–63·2)	0·4% (0·3–0·5)
	Poland	1·4% (0·9–2·1)	0·3% (0·2–0·4)	3·8% (2·8–5·1)	0·7% (0·5–1·0)	1·6% (1·2–2·2)	25·4% (21·1–29·8)	44·6% (39·2–49·6)	3·7% (3·2–4·3)	0·3% (0·2–0·4)	9·5% (7·4–11·9)	8·5% (6·7–10·7)	0·2% (0·2–0·3)
	Romania	4·4% (3·0–6·4)	0·7% (0·5–1·0)	8·2% (6·2–10·9)	1·1% (0·7–1·6)	0·7% (0·5–0·9)	18·6% (15·1–22·3)	30·7% (26·4–35·5)	2·0% (1·7–2·4)	0·3% (0·2–0·4)	19·9% (16·1–24·1)	12·8% (9·8–16·2)	0·7% (0·5–0·9)
	Serbia	0·7% (0·6–1·0)	0·2% (0·2–0·3)	3·4% (2·6–4·5)	0·4% (0·3–0·6)	1·0% (0·8–1·3)	7·0% (5·7–8·4)	37·1% (34·1–40·2)	0·9% (0·8–1·0)	0·5% (0·4–0·7)	10·2% (8·5–12·0)	38·1% (34·9–41·4)	0·4% (0·3–0·5)
	Slovakia	5·4% (3·8–7·4)	0·2% (0·2–0·3)	12·2% (9·0–16·0)	1·4% (1·0–2·0)	1·3% (1·0–1·8)	20·7% (17·0–24·9)	38·3% (33·6–42·9)	0·9% (0·8–1·0)	0·2% (0·1–0·2)	2·6% (1·9–3·5)	16·5% (12·8–20·6)	0·3% (0·2–0·4)
	Slovenia	1·5% (1·0–2·1)	0·3% (0·2–0·4)	5·4% (3·5–7·7)	2·9% (2·0–4·1)	3·1% (2·3–4·0)	10·7% (8·1–14·1)	45·0% (39·8–50·3)	1·2% (1·0–1·4)	0·2% (0·2–0·3)	3·4% (2·4–4·7)	26·0% (21·0–31·3)	0·4% (0·3–0·5)
Eastern Europe		1·3% (1·1–1·6)	0·3% (0·2–0·3)	25·1% (21·8–28·9)	0·9% (0·7–1·3)	1·6% (1·3–2·0)	12·9% (10·8–15·4)	37·8% (34·5–41·3)	1·6% (1·4–1·8)	0·4% (0·3–0·5)	5·9% (4·7–7·5)	10·0% (8·5–11·7)	2·0% (1·5–2·6)
	Belarus	6·8% (5·7–8·0)	0·2% (0·2–0·3)	15·9% (13·2–18·8)	0·4% (0·3–0·5)	0·4% (0·3–0·5)	14·3% (12·2–16·5)	48·4% (45·8–51·1)	0·9% (0·8–1·0)	0·7% (0·5–1·0)	3·7% (3·0–4·7)	7·9% (6·6–9·5)	0·3% (0·3–0·4)
	Estonia	1·7% (1·2–2·4)	0·3% (0·2–0·3)	25·5% (21·0–29·9)	1·2% (0·9–1·7)	1·4% (1·0–1·8)	29·6% (25·4–34·3)	32·9% (28·9–37·5)	1·3% (1·2–1·5)	0·3% (0·2–0·3)	3·5% (2·5–4·8)	1·8% (1·4–2·4)	0·5% (0·4–0·6)
	Latvia	1·8% (1·2–2·6)	0·3% (0·2–0·3)	23·4% (18·9–28·1)	1·1% (0·8–1·4)	1·2% (0·9–1·6)	12·7% (10·0–16·0)	45·0% (39·9–50·0)	1·1% (1·0–1·2)	0·3% (0·2–0·4)	4·1% (3·0–5·7)	8·3% (6·3–10·8)	0·7% (0·5–0·9)
	Lithuania	1·8% (1·2–2·5)	0·2% (0·2–0·3)	11·0% (8·2–14·3)	0·8% (0·6–1·3)	1·1% (0·8–1·5)	25·0% (20·2–30·0)	44·2% (39·1–49·2)	1·3% (1·1–1·5)	0·3% (0·2–0·4)	7·5% (5·6–10·0)	6·2% (4·6–8·1)	0·7% (0·5–0·9)
	Moldova	6·7% (4·7–9·0)	0·2% (0·2–0·3)	35·4% (30·2–40·6)	0·5% (0·4–0·7)	0·2% (0·1–0·3)	7·6% (5·7–10·0)	21·4% (17·9–25·3)	1·0% (0·9–1·2)	0·7% (0·5–1·0)	4·3% (3·1–5·7)	21·0% (16·8–25·5)	0·9% (0·7–1·2)
	Russia	0·8% (0·5–1·2)	0·3% (0·2–0·4)	26·5% (22·0–31·8)	1·2% (0·8–1·6)	2·1% (1·6–2·7)	13·7% (10·8–17·1)	35·6% (30·9–40·4)	1·8% (1·5–2·1)	0·3% (0·3–0·4)	6·5% (4·6–8·6)	8·7% (6·7–11·0)	2·7% (2·0–3·5)
	Ukraine	1·5% (1·1–2·1)	0·2% (0·2–0·3)	22·1% (17·9–26·7)	0·4% (0·3–0·5)	0·6% (0·4–0·8)	9·6% (7·3–12·4)	44·2% (39·4–48·8)	1·3% (1·1–1·5)	0·4% (0·3–0·6)	4·8% (3·6–6·4)	14·4% (11·3–17·7)	0·5% (0·4–0·6)
**High income**		**16·3% (15·3–17·3)**	**6·1% (5·5–6·8)**	**10·8% (10·1–11·4)**	**4·8% (4·4–5·2)**	**3·1% (2·8–3·4)**	**30·5% (29·4–31·7)**	**20·7% (19·9–21·6)**	**1·5% (1·4–1·6)**	**0·1% (0·1–0·1)**	**2·1% (1·8–2·3)**	**3·0% (2·7–3·3)**	**1·1% (0·9–1·2)**
Australasia		6·0% (4·4–8·1)	6·4% (4·8–8·3)	4·5% (3·8–5·4)	4·3% (3·4–5·4)	5·6% (4·4–7·3)	33·6% (29·6–38·1)	32·7% (29·0–36·4)	1·2% (1·1–1·4)	0·1% (0·1–0·2)	2·5% (2·0–3·2)	1·3% (1·1–1·6)	1·7% (1·1–2·6)
	Australia	6·1% (4·3–8·5)	6·1% (4·2–8·3)	3·4% (2·6–4·4)	3·8% (2·8–5·1)	5·7% (4·3–7·7)	34·4% (29·6–39·7)	33·8% (29·5–38·1)	1·2% (1·0–1·4)	0·2% (0·1–0·2)	2·6% (2·0–3·4)	0·9% (0·7–1·3)	1·9% (1·2–2·9)
	New Zealand	5·4% (4·0–7·1)	8·2% (6·2–10·3)	10·1% (8·3–12·1)	7·0% (5·7–8·6)	5·2% (4·0–6·4)	29·5% (25·9–33·2)	27·0% (23·9–30·4)	1·2% (1·1–1·4)	0·1% (0·1–0·1)	2·2% (1·7–2·9)	3·3% (2·5–4·3)	0·8% (0·6–1·0)
High-income Asia Pacific		12·9% (10·8–15·3)	8·9% (7·2–11·0)	9·0% (7·5–10·6)	11·7% (9·9–13·9)	3·6% (2·9–4·3)	9·6% (7·9–11·5)	31·0% (28·4–33·7)	1·0% (0·9–1·1)	0·1% (0·1–0·1)	5·9% (4·9–7·1)	2·3% (1·8–2·8)	4·0% (3·3–4·8)
	Brunei	13·0% (10·3–16·1)	8·0% (6·0–10·5)	9·7% (7·5–12·2)	7·4% (5·7–9·6)	1·7% (1·3–2·2)	14·4% (11·4–17·8)	29·8% (25·4–34·7)	1·4% (1·2–1·6)	0·1% (0·1–0·2)	6·9% (5·2–8·9)	2·5% (1·9–3·3)	5·0% (3·9–6·4)
	Japan	13·8% (10·4–17·5)	8·1% (5·8–11·0)	8·8% (6·8–11·1)	11·0% (8·6–13·9)	3·4% (2·5–4·4)	11·4% (8·7–14·6)	31·9% (28·0–36·2)	1·0% (0·9–1·1)	0·1% (0·1–0·1)	5·0% (3·6–6·6)	2·0% (1·5–2·7)	3·6% (2·7–4·8)
	Singapore	17·4% (14·5–20·6)	6·1% (4·5–7·9)	4·9% (3·7–6·2)	7·9% (6·3–10·1)	2·0% (1·6–2·6)	15·3% (12·5–18·2)	39·3% (35·5–43·1)	0·8% (0·7–0·9)	0·1% (0·1–0·1)	3·4% (2·5–4·5)	1·8% (1·3–2·4)	1·1% (0·8–1·4)
	South Korea	11·0% (8·1–14·4)	10·6% (7·9–13·8)	9·9% (7·8–12·5)	13·5% (10·2–17·2)	4·1% (3·1–5·4)	6·0% (4·5–7·9)	28·4% (24·5–32·4)	1·0% (0·9–1·1)	0·1% (0·1–0·1)	7·6% (5·8–9·8)	2·8% (2·0–3·8)	5·0% (3·8–6·5)
High-income North America		28·6% (26·2–31·1)	8·3% (6·8–10·0)	9·5% (8·2–11·0)	4·4% (3·6–5·3)	2·6% (2·1–3·3)	22·0% (19·6–24·5)	15·1% (13·3–17·1)	2·2% (2·0–2·5)	0·1% (0·1–0·1)	1·7% (1·3–2·1)	5·2% (4·4–6·2)	0·2% (0·2–0·3)
	Canada	35·1% (30·9–39·1)	9·5% (7·1–12·5)	6·5% (4·9–8·3)	4·5% (3·4–5·8)	1·4% (1·0–1·8)	23·9% (20·4–27·5)	14·4% (11·9–17·6)	2·0% (1·8–2·3)	0·1% (0·1–0·1)	1·3% (1·0–1·7)	1·0% (0·8–1·3)	0·3% (0·2–0·4)
	Greenland	27·9% (24·1–32·1)	8·2% (6·0–10·8)	6·6% (5·0–8·4)	3·9% (2·9–5·3)	0·9% (0·7–1·3)	27·2% (23·0–31·7)	16·2% (13·1–19·5)	2·6% (2·3–3·1)	0·1% (0·1–0·2)	1·8% (1·3–2·4)	4·3% (3·3–5·4)	0·3% (0·2–0·4)
	USA	27·8% (25·2–30·7)	8·2% (6·5–10·0)	9·9% (8·4–11·6)	4·4% (3·5–5·4)	2·8% (2·2–3·5)	21·8% (19·0–24·6)	15·2% (13·1–17·3)	2·3% (2·0–2·6)	0·1% (0·1–0·1)	1·7% (1·3–2·2)	5·7% (4·8–6·8)	0·2% (0·2–0·3)
Southern Latin America		14·4% (13·1–15·8)	0·2% (0·2–0·3)	16·2% (14·8–17·7)	5·3% (4·7–6·1)	6·0% (5·3–6·8)	36·0% (34·0–38·0)	17·5% (16·0–19·1)	1·7% (1·6–1·9)	0·2% (0·2–0·3)	1·0% (0·8–1·1)	0·5% (0·4–0·7)	0·8% (0·7–0·9)
	Argentina	16·3% (14·7–18·0)	0·2% (0·2–0·3)	9·6% (8·5–10·8)	6·5% (5·7–7·4)	5·9% (5·1–6·8)	36·9% (34·6–39·3)	20·2% (18·2–22·3)	1·9% (1·6–2·1)	0·2% (0·2–0·3)	0·7% (0·6–0·9)	0·6% (0·5–0·8)	0·9% (0·8–1·1)
	Chile	10·2% (7·7–13·3)	0·2% (0·2–0·3)	31·8% (27·8–36·3)	2·7% (2·0–3·8)	6·5% (5·0–8·3)	33·6% (29·3–37·6)	11·1% (9·1–13·5)	1·4% (1·2–1·7)	0·2% (0·1–0·2)	1·4% (1·1–1·9)	0·4% (0·3–0·5)	0·3% (0·3–0·5)
	Uruguay	14·0% (10·9–17·4)	0·3% (0·2–0·4)	13·9% (11·3–17·0)	4·9% (3·7–6·5)	4·4% (3·2–6·0)	39·3% (34·0–44·1)	18·8% (15·5–22·4)	1·9% (1·6–2·2)	0·3% (0·2–0·3)	1·0% (0·8–1·3)	0·6% (0·4–0·8)	0·7% (0·6–0·9)
Western Europe		8·3% (7·5–9·2)	4·3% (3·7–4·9)	12·0% (11·1–13·0)	2·7% (2·4–2·9)	2·7% (2·4–3·1)	43·6% (42·0–45·1)	21·5% (20·4–22·6)	1·1% (1·0–1·2)	0·1% (0·1–0·1)	1·2% (1·1–1·3)	1·9% (1·7–2·1)	0·7% (0·7–0·8)
	Andorra	7·2% (4·9–10·0)	3·4% (2·3–5·2)	14·4% (11·1–17·9)	3·2% (2·3–4·4)	1·9% (1·4–2·6)	42·8% (37·5–47·8)	22·6% (19·1–27·0)	1·0% (0·9–1·2)	0·1% (0·1–0·1)	1·1% (0·8–1·5)	1·4% (1·1–1·9)	0·7% (0·5–0·9)
	Austria	1·3% (0·8–1·8)	0·6% (0·4–0·9)	16·2% (13·2–19·5)	4·1% (3·1–5·4)	1·9% (1·4–2·5)	42·6% (38·1–47·0)	28·8% (24·9–32·9)	1·1% (0·9–1·2)	0·1% (0·1–0·2)	1·9% (1·4–2·6)	0·7% (0·6–1·0)	0·5% (0·4–0·7)
	Belgium	14·6% (11·4–17·8)	6·1% (4·4–8·3)	6·5% (5·0–8·4)	2·8% (2·0–3·9)	1·3% (1·0–1·8)	53·0% (48·7–57·1)	11·1% (8·9–13·4)	0·9% (0·8–1·1)	0·1% (0·1–0·1)	1·4% (1·0–1·9)	1·8% (1·3–2·5)	0·3% (0·2–0·4)
	Cyprus	6·5% (4·6–8·9)	3·4% (2·3–4·8)	14·8% (11·5–18·6)	2·3% (1·7–3·1)	1·2% (0·9–1·7)	46·4% (41·3–51·5)	20·5% (16·7–24·7)	1·2% (1·0–1·3)	0·1% (0·1–0·2)	1·3% (0·9–1·7)	1·5% (1·1–2·1)	0·8% (0·6–1·0)
	Denmark	5·6% (3·9–7·8)	2·7% (1·8–3·9)	12·1% (9·5–14·8)	4·4% (3·2–5·8)	2·8% (2·1–3·8)	44·5% (40·0–49·2)	24·1% (20·3–27·9)	0·9% (0·8–1·1)	0·1% (0·1–0·1)	0·8% (0·6–1·2)	1·3% (1·0–1·7)	0·6% (0·5–0·7)
	Finland	8·5% (6·6–10·7)	1·0% (0·7–1·4)	16·1% (13·5–19·1)	2·1% (1·6–2·9)	2·9% (2·2–3·6)	35·2% (31·4–39·0)	32·3% (28·4–36·3)	0·7% (0·6–0·7)	0·1% (0·1–0·1)	0·2% (0·2–0·2)	0·6% (0·5–0·9)	0·3% (0·2–0·4)
	France	7·4% (5·4–9·6)	0·2% (0·2–0·2)	17·9% (14·6–21·4)	2·6% (2·0–3·4)	2·7% (2·0–3·7)	54·0% (49·9–58·2)	10·8% (8·9–13·3)	1·1% (1·0–1·2)	0·1% (0·1–0·1)	1·1% (0·9–1·4)	1·2% (0·9–1·6)	0·8% (0·6–1·0)
	Germany	11·9% (9·4–15·2)	2·7% (1·8–3·9)	9·9% (7·8–12·4)	2·6% (1·9–3·5)	0·8% (0·6–1·1)	51·8% (46·8–57·0)	17·0% (13·9–20·6)	0·9% (0·7–1·0)	0·1% (0·1–0·1)	1·3% (0·9–1·8)	0·6% (0·5–0·8)	0·4% (0·3–0·5)
	Greece	7·7% (5·4–10·7)	4·1% (2·7–5·9)	15·7% (12·2–19·5)	1·7% (1·2–2·4)	0·7% (0·5–1·0)	47·4% (42·2–52·7)	17·0% (13·7–20·9)	1·5% (1·3–1·7)	0·2% (0·1–0·2)	1·6% (1·1–2·1)	1·6% (1·2–2·1)	1·0% (0·8–1·4)
	Iceland	5·5% (3·8–7·6)	2·7% (1·8–3·8)	12·3% (9·6–15·2)	3·8% (2·7–5·1)	2·3% (1·6–3·2)	46·4% (41·3–51·0)	23·1% (19·4–27·0)	1·1% (0·9–1·2)	0·1% (0·1–0·1)	0·9% (0·6–1·2)	1·3% (1·0–1·8)	0·7% (0·5–0·8)
	Ireland	10·9% (8·0–14·1)	2·5% (1·7–3·5)	8·4% (6·5–10·6)	2·8% (2·1–3·8)	1·8% (1·3–2·5)	26·7% (22·3–31·2)	41·4% (36·9–46·0)	0·8% (0·7–1·0)	0·1% (0·1–0·1)	1·9% (1·3–2·8)	1·8% (1·3–2·6)	0·9% (0·7–1·1)
	Israel	6·1% (4·2–8·6)	3·3% (2·3–4·6)	13·7% (10·6–17·2)	2·2% (1·5–3·3)	0·8% (0·6–1·2)	50·9% (45·5–56·0)	17·6% (14·2–21·5)	1·4% (1·2–1·7)	0·2% (0·1–0·2)	1·3% (1·0–1·8)	1·5% (1·2–2·0)	0·9% (0·7–1·2)
	Italy	7·6% (5·3–10·7)	3·9% (2·6–5·7)	15·0% (11·5–18·6)	2·0% (1·4–2·8)	0·8% (0·6–1·1)	47·3% (42·2–52·5)	17·9% (14·2–21·7)	1·4% (1·2–1·7)	0·2% (0·1–0·2)	1·4% (1·1–2·0)	1·5% (1·1–2·0)	1·0% (0·8–1·3)
	Luxembourg	5·8% (4·1–7·8)	2·7% (1·9–3·9)	12·6% (9·8–15·9)	3·8% (2·8–5·1)	2·5% (1·8–3·4)	44·7% (40·2–49·8)	23·9% (20·2–27·9)	1·0% (0·9–1·2)	0·1% (0·1–0·1)	0·9% (0·6–1·2)	1·3% (1·0–1·8)	0·6% (0·5–0·8)
	Malta	6·7% (4·6–9·3)	3·7% (2·5–5·3)	14·7% (11·1–18·6)	1·8% (1·3–2·6)	0·8% (0·6–1·1)	49·1% (43·4–54·4)	17·6% (14·2–21·5)	1·4% (1·2–1·6)	0·2% (0·1–0·2)	1·5% (1·1–2·0)	1·6% (1·1–2·1)	1·0% (0·8–1·3)
	Monaco	6·0% (4·3–8·2)	2·7% (1·8–3·9)	11·8% (9·3–14·9)	5·4% (4·1–7·1)	4·0% (3·0–5·3)	40·3% (35·8–44·9)	26·3% (22·5–30·3)	0·9% (0·8–1·0)	0·1% (0·1–0·1)	0·7% (0·5–1·0)	1·3% (1·0–1·6)	0·5% (0·4–0·7)
	Netherlands	5·9% (4·0–8·3)	8·4% (6·2–11·1)	5·9% (4·5–7·9)	3·6% (2·6–4·9)	2·4% (1·7–3·2)	54·0% (49·2–58·5)	12·7% (10·2–15·8)	0·9% (0·8–1·1)	0·1% (0·1–0·1)	1·8% (1·3–2·5)	2·4% (1·8–3·2)	1·9% (1·5–2·5)
	Norway	6·6% (4·7–8·7)	3·0% (2·0–4·2)	23·8% (20·1–27·6)	6·2% (4·5–8·1)	1·8% (1·4–2·5)	41·9% (37·8–45·9)	14·0% (11·6–16·7)	1·0% (0·9–1·2)	0·1% (0·1–0·1)	0·2% (0·2–0·3)	0·5% (0·4–0·7)	0·9% (0·7–1·1)
	Portugal	2·1% (1·5–3·0)	0·3% (0·2–0·4)	6·6% (5·1–8·4)	11·6% (8·8–14·9)	0·2% (0·2–0·3)	34·9% (30·5–40·0)	18·9% (15·4–22·7)	2·9% (2·5–3·4)	0·1% (0·1–0·2)	2·5% (1·9–3·4)	18·7% (14·8–23·1)	1·1% (0·9–1·4)
	San Marino	6·6% (4·5–9·0)	3·1% (2·1–4·7)	13·0% (10·1–16·5)	4·1% (3·0–5·5)	2·6% (1·9–3·5)	42·8% (38·3–48·0)	23·9% (20·1–28·1)	1·0% (0·9–1·2)	0·1% (0·1–0·1)	0·9% (0·6–1·2)	1·4% (1·0–1·8)	0·6% (0·5–0·8)
	Spain	2·9% (2·1–4·0)	5·6% (4·4–7·0)	10·6% (8·3–13·3)	0·7% (0·5–0·9)	0·4% (0·3–0·6)	23·0% (19·4–26·6)	52·1% (48·6–55·4)	0·8% (0·7–0·9)	0·2% (0·1–0·2)	0·6% (0·4–0·7)	1·8% (1·4–2·2)	1·5% (1·2–1·8)
	Sweden	6·2% (4·6–8·0)	2·1% (1·5–3·0)	19·3% (16·4–22·5)	1·4% (1·1–1·8)	3·4% (2·5–4·5)	32·0% (27·8–36·2)	26·1% (22·3–30·2)	3·5% (3·1–4·0)	0·1% (0·1–0·1)	2·4% (1·8–3·2)	3·0% (2·3–3·9)	0·4% (0·3–0·5)
	Switzerland	5·5% (3·8–7·4)	2·5% (1·6–3·5)	11·8% (9·1–14·8)	5·4% (4·0–7·2)	4·7% (3·5–6·3)	40·1% (35·5–44·5)	26·8% (22·9–30·8)	0·8% (0·7–0·9)	0·1% (0·1–0·1)	0·7% (0·5–1·0)	1·3% (1·0–1·7)	0·4% (0·4–0·6)
	UK	11·6% (9·2–14·6)	10·6% (7·7–13·6)	6·0% (4·7–7·6)	2·3% (1·6–3·1)	8·2% (6·4–10·3)	33·0% (28·9–36·9)	24·0% (20·3–28·1)	0·8% (0·7–0·9)	0·1% (0·1–0·1)	0·7% (0·5–0·9)	2·5% (1·9–3·5)	0·3% (0·2–0·4)
**Latin America and Caribbean**		**31·6% (29·4–33·6)**	**1·9% (1·6–2·4)**	**6·9% (6·5–7·5)**	**11·3% (10·6–12·2)**	**3·3% (3·0–3·5)**	**20·8% (18·8–22·8)**	**15·2% (14·0–16·5)**	**1·1% (1·0–1·2)**	**0·3% (0·3–0·3)**	**2·8% (2·6–3·0)**	**4·2% (3·6–4·9)**	**0·6% (0·5–0·7)**
Andean Latin America		18·0% (16·7–19·3)	1·5% (1·3–1·7)	6·3% (5·5–7·1)	20·2% (18·9–21·5)	5·5% (4·9–6·2)	10·3% (9·3–11·5)	15·6% (14·8–16·5)	1·3% (1·2–1·4)	0·6% (0·5–0·7)	12·6% (11·8–13·4)	7·7% (7·1–8·3)	0·5% (0·5–0·6)
	Bolivia	12·9% (10·8–15·1)	6·9% (6·0–8·0)	10·5% (8·5–12·7)	17·9% (15·1–20·9)	6·8% (5·5–8·3)	5·6% (4·5–6·7)	9·6% (7·8–11·7)	1·0% (0·9–1·2)	1·8% (1·4–2·2)	22·3% (19·7–25·1)	4·4% (3·5–5·6)	0·3% (0·3–0·4)
	Ecuador	33·0% (29·4–37·0)	0·3% (0·3–0·4)	8·8% (6·8–11·0)	15·1% (12·0–18·6)	7·0% (5·4–8·9)	13·8% (11·1–17·0)	9·2% (7·5–11·3)	1·5% (1·3–1·8)	0·6% (0·5–0·8)	5·7% (4·5–7·1)	4·6% (3·6–5·9)	0·2% (0·2–0·3)
	Peru	10·9% (10·1–11·7)	0·6% (0·5–0·7)	3·6% (3·0–4·4)	23·8% (22·4–25·2)	4·3% (3·9–4·7)	9·7% (8·9–10·4)	21·1% (20·0–22·1)	1·2% (1·1–1·3)	0·2% (0·2–0·2)	13·6% (12·6–14·6)	10·3% (9·5–11·2)	0·8% (0·7–0·9)
Caribbean		27·4% (26·3–28·5)	0·5% (0·4–0·6)	12·2% (11·3–13·1)	15·2% (14·0–16·5)	3·2% (2·9–3·7)	16·8% (16·0–17·6)	19·6% (18·6–20·6)	0·9% (0·9–1·0)	0·4% (0·3–0·5)	1·4% (1·3–1·6)	1·7% (1·5–1·9)	0·7% (0·6–0·7)
	Antigua and Barbuda	26·8% (22·9–30·8)	0·2% (0·2–0·3)	2·7% (2·1–3·3)	14·7% (11·5–18·1)	8·1% (6·4–10·1)	20·0% (16·5–23·5)	23·2% (19·4–27·1)	0·8% (0·7–0·9)	0·1% (0·1–0·2)	0·5% (0·4–0·6)	0·8% (0·7–1·0)	2·2% (1·8–2·8)
	The Bahamas	23·1% (19·2–26·8)	0·2% (0·2–0·3)	3·9% (3·0–5·0)	11·3% (9·1–13·9)	18·0% (14·8–21·4)	22·3% (18·6–26·3)	17·1% (14·1–20·3)	1·8% (1·5–2·0)	0·1% (0·1–0·1)	0·4% (0·3–0·5)	1·5% (1·2–1·9)	0·3% (0·2–0·3)
	Barbados	8·3% (6·1–11·0)	0·3% (0·2–0·4)	5·6% (4·2–7·2)	10·9% (8·0–14·1)	1·2% (0·8–1·8)	32·8% (27·7–37·7)	33·4% (28·9–38·2)	1·2% (1·0–1·4)	0·2% (0·2–0·3)	1·8% (1·4–2·2)	2·7% (2·0–3·6)	1·8% (1·4–2·2)
	Belize	30·9% (27·0–35·2)	0·3% (0·3–0·5)	2·6% (2·0–3·4)	20·5% (16·6–25·0)	4·2% (3·1–5·5)	21·9% (18·5–25·8)	12·8% (10·6–15·4)	1·1% (0·9–1·2)	0·4% (0·3–0·5)	2·7% (2·1–3·5)	1·9% (1·4–2·5)	0·6% (0·5–0·8)
	Bermuda	15·4% (12·3–18·9)	0·2% (0·1–0·2)	2·1% (1·6–2·7)	26·5% (22·7–30·4)	17·3% (14·1–20·8)	10·5% (8·5–12·9)	26·3% (23·1–29·7)	0·5% (0·4–0·6)	0·1% (0·1–0·1)	0·3% (0·2–0·4)	0·7% (0·5–0·9)	0·2% (0·1–0·2)
	Cuba	22·4% (20·4–24·4)	0·2% (0·1–0·2)	31·7% (29·0–34·5)	1·6% (1·2–2·0)	0·9% (0·7–1·1)	14·9% (13·5–16·3)	26·2% (24·1–28·2)	0·6% (0·5–0·6)	0·2% (0·1–0·2)	0·4% (0·3–0·5)	0·3% (0·2–0·4)	0·7% (0·6–0·9)
	Dominica	22·0% (18·5–25·6)	0·2% (0·2–0·3)	2·6% (1·9–3·4)	33·3% (28·8–38·0)	6·3% (4·9–8·2)	13·3% (10·8–16·2)	19·9% (16·6–23·5)	0·6% (0·5–0·7)	0·1% (0·1–0·1)	0·4% (0·3–0·5)	0·7% (0·5–0·9)	0·6% (0·5–0·8)
	Dominican Republic	46·2% (44·0–48·4)	0·3% (0·2–0·4)	3·9% (3·4–4·5)	13·6% (12·6–14·7)	3·4% (3·0–4·0)	24·3% (22·7–26·0)	4·3% (3·7–5·1)	1·1% (1·0–1·2)	0·3% (0·2–0·4)	1·0% (0·8–1·2)	1·0% (0·8–1·2)	0·5% (0·4–0·6)
	Grenada	24·1% (19·7–28·7)	0·4% (0·3–0·5)	4·3% (3·2–5·8)	17·4% (13·2–22·1)	4·0% (2·9–5·5)	21·9% (17·5–26·7)	23·4% (19·3–27·5)	1·2% (1·0–1·4)	0·3% (0·2–0·4)	1·1% (0·8–1·4)	1·4% (1·0–1·8)	0·6% (0·5–0·8)
	Guyana	8·4% (6·7–10·2)	0·4% (0·4–0·6)	13·3% (11·3–15·6)	13·7% (11·6–16·0)	6·9% (5·5–8·4)	18·1% (15·3–21·1)	32·7% (28·8–36·6)	1·6% (1·4–1·8)	0·7% (0·6–0·9)	1·0% (0·8–1·3)	1·4% (1·1–1·9)	1·8% (1·4–2·2)
	Haiti	3·8% (2·7–5·1)	0·6% (0·5–0·8)	0·7% (0·5–0·9)	48·2% (43·3–52·7)	7·1% (5·3–9·2)	6·7% (5·2–8·7)	21·4% (18·4–24·9)	1·3% (1·2–1·5)	1·3% (1·0–1·8)	3·7% (2·8–4·8)	4·3% (3·2–5·5)	0·9% (0·7–1·1)
	Jamaica	18·3% (15·0–22·0)	0·3% (0·2–0·4)	1·4% (1·0–1·8)	24·0% (19·9–27·9)	1·3% (1·0–1·7)	18·5% (15·1–22·2)	31·3% (27·2–35·5)	1·0% (0·9–1·2)	0·2% (0·2–0·3)	0·5% (0·4–0·6)	2·9% (2·2–3·7)	0·3% (0·2–0·4)
	Puerto Rico	49·9% (45·7–54·0)	2·3% (1·6–3·2)	1·3% (0·9–1·8)	5·6% (4·3–7·3)	3·2% (2·4–4·3)	9·2% (7·1–11·6)	21·2% (18·0–24·5)	0·7% (0·6–0·8)	0·1% (0·1–0·1)	3·1% (2·4–4·0)	3·3% (2·6–4·1)	0·2% (0·2–0·3)
	Saint Kitts and Nevis	12·1% (9·5–15·3)	0·3% (0·2–0·3)	4·7% (3·7–6·0)	20·1% (16·5–24·1)	8·7% (6·7–11·0)	18·2% (15·1–21·9)	31·3% (27·4–35·7)	2·0% (1·7–2·3)	0·1% (0·1–0·2)	0·5% (0·4–0·6)	0·9% (0·7–1·1)	1·2% (0·9–1·5)
	Saint Lucia	10·2% (7·4–13·3)	0·4% (0·3–0·5)	5·4% (4·0–7·0)	12·6% (9·9–15·9)	1·0% (0·7–1·7)	35·5% (30·6–40·8)	30·5% (26·2–34·7)	0·9% (0·8–1·1)	0·3% (0·3–0·5)	1·7% (1·2–2·4)	0·8% (0·6–1·2)	0·6% (0·5–0·9)
	Saint Vincent and the Grenadines	27·3% (23·5–31·3)	0·2% (0·2–0·3)	3·8% (2·9–5·0)	23·0% (18·9–27·1)	1·2% (0·9–1·6)	20·8% (17·4–24·4)	20·3% (17·1–23·8)	0·7% (0·6–0·8)	0·2% (0·1–0·2)	0·6% (0·5–0·8)	0·7% (0·5–0·9)	1·2% (0·9–1·5)
	Suriname	11·6% (9·8–13·7)	0·4% (0·3–0·5)	4·6% (3·6–5·6)	13·1% (11·2–15·3)	0·6% (0·4–0·8)	56·2% (53·2–59·6)	10·6% (8·9–12·5)	1·2% (1·0–1·3)	0·3% (0·2–0·3)	0·5% (0·4–0·6)	0·4% (0·3–0·5)	0·6% (0·4–0·7)
	Trinidad and Tobago	14·0% (10·3–18·0)	0·4% (0·3–0·5)	4·2% (3·1–5·6)	5·9% (4·1–8·5)	1·5% (1·0–2·1)	22·7% (18·4–27·6)	42·2% (37·1–47·4)	1·7% (1·4–1·9)	0·3% (0·2–0·3)	2·7% (2·1–3·4)	2·5% (1·9–3·3)	2·0% (1·6–2·6)
	Virgin Islands	15·1% (12·1–18·2)	0·2% (0·1–0·2)	2·1% (1·6–2·7)	25·8% (22·2–29·4)	15·7% (12·9–18·7)	10·3% (8·3–12·6)	29·1% (25·5–32·9)	0·5% (0·5–0·6)	0·1% (0·1–0·1)	0·3% (0·2–0·4)	0·7% (0·5–0·8)	0·2% (0·2–0·2)
Central Latin America		40·7% (39·3–42·0)	1·4% (1·2–1·5)	11·6% (10·7–12·6)	12·1% (11·3–12·9)	5·6% (5·1–6·1)	9·3% (8·6–10·1)	12·2% (11·4–13·0)	1·3% (1·2–1·4)	0·2% (0·2–0·2)	2·4% (2·2–2·7)	3·0% (2·7–3·3)	0·3% (0·3–0·4)
	Colombia	34·8% (32·1–37·6)	2·3% (1·8–2·9)	7·5% (6·2–9·1)	18·6% (16·3–21·0)	7·5% (6·3–8·9)	10·3% (8·6–12·0)	11·5% (9·9–13·2)	1·3% (1·2–1·5)	0·2% (0·1–0·2)	2·2% (1·7–2·7)	3·6% (2·9–4·4)	0·3% (0·2–0·4)
	Costa Rica	25·9% (23·9–28·0)	4·4% (3·6–5·4)	4·0% (3·3–4·9)	14·8% (13·2–16·5)	0·8% (0·7–1·1)	33·6% (31·3–36·0)	12·0% (10·2–14·0)	0·8% (0·7–0·9)	1·0% (0·8–1·4)	1·3% (1·0–1·6)	0·4% (0·3–0·5)	0·9% (0·7–1·1)
	El Salvador	49·8% (46·0–53·6)	0·5% (0·4–0·6)	2·4% (1·8–3·2)	26·7% (22·6–30·6)	0·8% (0·6–1·0)	6·2% (4·8–7·8)	7·4% (6·0–8·9)	0·7% (0·6–0·8)	0·2% (0·2–0·3)	2·7% (2·2–3·4)	2·4% (1·8–3·0)	0·2% (0·2–0·3)
	Guatemala	33·6% (29·4–38·1)	1·1% (0·8–1·4)	2·6% (1·9–3·4)	29·0% (24·6–33·9)	4·2% (3·1–5·7)	6·1% (4·8–8·0)	8·7% (7·0–10·5)	0·7% (0·6–0·9)	0·3% (0·2–0·3)	7·1% (5·5–9·0)	6·3% (4·9–8·1)	0·2% (0·2–0·3)
	Honduras	31·4% (29·8–33·1)	0·5% (0·4–0·5)	8·7% (7·6–10·0)	26·2% (24·7–27·9)	4·5% (4·0–5·1)	14·5% (13·2–15·9)	6·7% (5·9–7·7)	0·7% (0·7–0·8)	0·2% (0·1–0·2)	2·4% (1·9–2·9)	3·9% (3·0–5·0)	0·3% (0·3–0·4)
	Mexico	46·1% (43·8–48·2)	1·2% (1·0–1·4)	15·6% (14·0–17·3)	5·8% (4·9–6·7)	6·5% (5·8–7·2)	4·5% (3·8–5·3)	14·7% (13·3–16·0)	1·3% (1·2–1·5)	0·2% (0·1–0·2)	1·9% (1·6–2·3)	2·0% (1·6–2·4)	0·3% (0·3–0·4)
	Nicaragua	37·4% (33·1–41·8)	0·5% (0·3–0·6)	5·5% (4·1–7·0)	30·1% (25·4–35·0)	0·5% (0·3–0·6)	13·5% (10·6–17·0)	8·1% (6·5–10·0)	0·7% (0·6–0·9)	0·6% (0·5–0·8)	1·4% (1·1–1·9)	1·5% (1·1–1·9)	0·2% (0·2–0·3)
	Panama	31·8% (27·9–36·0)	0·6% (0·4–0·8)	3·3% (2·6–4·4)	32·2% (28·0–36·6)	0·9% (0·7–1·3)	19·4% (16·2–23·0)	7·7% (6·1–9·5)	1·0% (0·8–1·1)	0·1% (0·1–0·2)	1·0% (0·8–1·3)	1·5% (1·1–2·0)	0·5% (0·4–0·7)
	Venezuela	36·5% (31·4–41·2)	0·5% (0·3–0·7)	13·1% (10·3–16·2)	4·1% (3·0–5·6)	1·9% (1·4–2·6)	23·8% (19·7–28·2)	7·6% (5·9–9·7)	1·8% (1·5–2·1)	0·2% (0·2–0·3)	4·0% (3·0–5·2)	5·9% (4·6–7·8)	0·6% (0·4–0·7)
Tropical Latin America		26·9% (22·5–31·4)	2·8% (2·0–3·7)	2·1% (1·7–2·8)	8·1% (6·7–10·0)	0·6% (0·5–0·8)	34·0% (29·8–38·3)	17·3% (14·6–20·1)	0·9% (0·8–1·1)	0·3% (0·3–0·4)	1·2% (0·9–1·6)	4·8% (3·6–6·4)	0·8% (0·6–1·0)
	Brazil	27·4% (22·8–32·0)	2·8% (2·0–3·8)	2·0% (1·5–2·6)	7·5% (6·0–9·4)	0·6% (0·5–0·8)	34·3% (30·0–38·7)	17·3% (14·5–20·1)	0·9% (0·8–1·1)	0·3% (0·3–0·4)	1·2% (0·9–1·6)	4·9% (3·6–6·5)	0·8% (0·6–1·0)
	Paraguay	10·8% (8·9–12·6)	0·2% (0·2–0·3)	8·8% (7·2–10·8)	32·0% (28·7–35·4)	0·3% (0·2–0·3)	23·1% (20·4–26·1)	18·1% (15·6–20·8)	0·6% (0·6–0·7)	0·5% (0·4–0·7)	2·6% (2·0–3·4)	1·8% (1·3–2·4)	1·1% (0·8–1·5)
**North Africa and Middle East**		**8·0% (7·0–9·0)**	**0·9% (0·7–1·1)**	**21·1% (19·4–22·6)**	**8·9% (7·8–10·1)**	**2·6% (2·2–3·0)**	**28·1% (26·7–29·8)**	**11·7% (10·7–12·9)**	**0·7% (0·7–0·8)**	**1·3% (1·2–1·5)**	**2·6% (2·4–2·8)**	**13·7% (12·6–15·1)**	**0·3% (0·3–0·3)**
	Afghanistan	6·2% (4·4–8·7)	0·5% (0·4–0·7)	5·2% (3·8–6·9)	35·1% (27·6–43·1)	2·7% (1·7–3·9)	25·2% (19·8–31·0)	12·6% (9·7–16·1)	1·1% (0·9–1·3)	4·5% (3·2–6·3)	0·7% (0·5–1·0)	5·9% (4·4–7·8)	0·5% (0·4–0·7)
	Algeria	0·8% (0·6–1·2)	0·3% (0·2–0·3)	4·8% (3·8–5·9)	0·4% (0·2–0·5)	0·7% (0·5–0·9)	72·0% (69·3–74·4)	4·2% (3·4–5·1)	1·1% (0·9–1·3)	1·8% (1·4–2·4)	8·3% (6·9–9·7)	5·5% (4·3–6·8)	0·3% (0·2–0·4)
	Bahrain	9·5% (6·4–13·4)	0·3% (0·2–0·4)	6·3% (4·4–9·0)	1·3% (0·8–2·1)	7·9% (5·4–10·8)	20·6% (15·6–26·0)	21·4% (16·5–26·6)	1·4% (1·1–1·7)	0·8% (0·6–1·2)	3·1% (2·2–4·3)	27·0% (21·7–32·8)	0·4% (0·3–0·5)
	Egypt	2·0% (1·4–2·8)	0·2% (0·2–0·3)	46·3% (40·3–51·9)	19·9% (15·5–25·2)	2·6% (1·8–3·8)	24·0% (19·5–29·0)	2·1% (1·5–2·9)	0·5% (0·4–0·6)	1·1% (0·8–1·4)	0·6% (0·4–0·8)	0·5% (0·4–0·7)	0·2% (0·1–0·3)
	Iran	15·9% (12·0–20·2)	2·8% (2·1–3·9)	10·7% (7·9–14·1)	7·6% (5·0–11·0)	0·3% (0·2–0·5)	21·6% (16·8–26·9)	18·9% (14·9–23·9)	0·4% (0·4–0·5)	0·2% (0·2–0·3)	0·4% (0·3–0·5)	20·9% (16·5–25·9)	0·1% (0·1–0·2)
	Iraq	5·8% (4·9–6·9)	0·3% (0·2–0·4)	17·2% (14·9–19·6)	8·5% (7·0–10·2)	0·7% (0·5–1·1)	29·3% (26·5–32·3)	6·2% (5·3–7·1)	0·7% (0·6–0·8)	2·0% (1·5–2·5)	2·5% (2·0–3·1)	26·6% (23·4–29·6)	0·3% (0·2–0·4)
	Jordan	3·1% (2·3–4·2)	0·2% (0·2–0·3)	37·9% (33·9–42·1)	2·2% (1·5–3·0)	1·0% (0·7–1·4)	15·0% (12·7–17·6)	10·7% (8·9–12·5)	0·8% (0·7–1·0)	1·9% (1·5–2·4)	4·1% (3·1–5·3)	22·5% (19·6–25·5)	0·6% (0·4–0·8)
	Kuwait	2·7% (1·7–4·2)	0·5% (0·3–0·7)	13·4% (9·7–17·9)	5·1% (3·2–7·6)	6·4% (4·3–9·2)	53·6% (47·2–59·8)	9·5% (6·8–12·9)	0·7% (0·5–0·8)	0·8% (0·5–1·1)	3·2% (2·2–4·4)	4·0% (2·8–5·7)	0·2% (0·2–0·3)
	Lebanon	5·1% (3·1–7·8)	0·3% (0·2–0·4)	24·0% (19·0–29·8)	1·3% (0·8–2·0)	6·8% (4·5–9·9)	22·5% (16·7–28·6)	15·4% (11·7–20·0)	0·5% (0·4–0·6)	0·3% (0·2–0·4)	8·7% (6·4–11·6)	14·6% (10·9–19·2)	0·6% (0·5–0·9)
	Libya	9·0% (5·7–13·5)	0·4% (0·3–0·6)	12·3% (9·1–16·0)	2·7% (1·7–4·2)	9·1% (6·0–12·9)	31·2% (24·4–38·2)	6·2% (4·7–8·1)	0·7% (0·6–0·8)	2·4% (1·8–3·4)	18·3% (14·7–22·7)	5·1% (3·5–7·1)	2·6% (1·7–3·9)
	Morocco	1·9% (1·4–2·5)	0·2% (0·2–0·3)	7·0% (5·9–8·4)	2·4% (1·7–3·4)	0·7% (0·4–1·0)	66·6% (63·5–69·4)	4·0% (3·3–4·8)	0·5% (0·5–0·6)	1·5% (1·2–1·9)	7·9% (6·5–9·3)	7·0% (5·9–8·4)	0·2% (0·2–0·3)
	Oman	10·6% (7·6–14·6)	0·5% (0·4–0·8)	8·6% (6·2–11·7)	24·7% (18·1–31·8)	0·6% (0·4–0·9)	17·0% (12·6–22·1)	9·9% (7·3–13·1)	0·9% (0·8–1·1)	1·9% (1·3–2·6)	3·9% (2·8–5·4)	20·4% (15·8–25·6)	1·0% (0·7–1·4)
	Palestine	3·1% (2·5–3·8)	0·3% (0·2–0·4)	44·4% (41·8–47·2)	2·3% (1·7–3·0)	0·5% (0·3–0·7)	12·7% (11·1–14·4)	10·4% (9·0–11·9)	0·6% (0·5–0·7)	2·3% (1·9–2·8)	6·6% (5·5–7·7)	16·5% (14·7–18·5)	0·3% (0·3–0·4)
	Qatar	2·8% (1·8–4·4)	2·2% (1·5–3·2)	24·6% (18·5–30·8)	16·2% (10·9–22·3)	1·3% (0·9–2·0)	32·8% (25·6–40·1)	9·8% (7·2–12·9)	1·3% (1·0–1·5)	1·0% (0·7–1·4)	3·0% (2·0–4·2)	4·3% (2·9–6·2)	0·6% (0·5–0·9)
	Saudi Arabia	1·0% (0·6–1·7)	0·4% (0·3–0·6)	12·7% (9·0–17·4)	1·7% (1·0–2·9)	33·9% (26·1–42·9)	39·3% (32·0–46·7)	2·7% (1·8–4·0)	0·7% (0·6–0·9)	4·0% (2·9–5·7)	0·9% (0·6–1·4)	2·3% (1·6–3·4)	0·3% (0·2–0·4)
	Sudan	3·9% (2·4–6·0)	0·8% (0·6–1·2)	3·8% (2·6–5·5)	17·9% (12·3–24·8)	4·4% (2·8–6·6)	59·2% (51·1–66·9)	1·6% (1·1–2·2)	1·8% (1·4–2·2)	2·1% (1·4–3·0)	1·9% (1·3–2·7)	0·9% (0·6–1·4)	1·7% (1·2–2·4)
	Syria	5·1% (3·2–7·5)	0·3% (0·2–0·4)	40·1% (33·0–46·7)	2·8% (1·7–4·4)	0·6% (0·4–0·9)	22·2% (16·9–28·4)	4·2% (3·0–5·8)	0·7% (0·6–0·8)	2·6% (1·8–3·6)	14·8% (11·2–19·0)	6·0% (4·2–8·2)	0·6% (0·4–0·8)
	Tunisia	3·6% (2·5–4·9)	0·3% (0·2–0·4)	40·3% (36·4–44·3)	2·7% (1·9–3·6)	1·9% (1·4–2·6)	36·9% (33·3–40·2)	2·3% (1·8–3·0)	0·6% (0·5–0·7)	0·6% (0·5–0·8)	9·7% (8·0–11·6)	0·6% (0·4–0·9)	0·5% (0·4–0·7)
	Turkey	13·4% (11·9–15·1)	0·2% (0·2–0·3)	20·4% (17·5–23·5)	1·7% (1·3–2·3)	0·7% (0·5–0·9)	6·9% (5·7–8·2)	26·2% (23·9–28·6)	1·0% (0·9–1·2)	0·2% (0·1–0·2)	0·6% (0·5–0·8)	28·4% (25·6–31·2)	0·3% (0·2–0·3)
	United Arab Emirates	12·7% (7·9–18·3)	0·4% (0·3–0·6)	11·8% (7·8–17·2)	20·2% (13·2–28·6)	5·5% (3·3–8·5)	33·1% (25·2–41·9)	10·1% (6·7–14·3)	0·9% (0·7–1·1)	0·4% (0·3–0·6)	1·6% (1·0–2·3)	2·8% (1·8–4·2)	0·7% (0·5–1·0)
	Yemen	7·4% (5·1–10·4)	0·5% (0·3–0·7)	16·6% (12·4–21·4)	18·3% (13·0–24·2)	2·7% (1·8–3·8)	32·6% (26·1–39·9)	1·8% (1·3–2·6)	0·9% (0·8–1·1)	8·0% (5·8–10·8)	4·5% (3·3–6·1)	6·1% (4·5–8·2)	0·6% (0·4–0·8)
**South Asia**		**53·1% (49·6–56·2)**	**1·3% (1·0–1·7)**	**3·3% (2·7–4·2)**	**4·3% (3·8–4·8)**	**1·1% (1·0–1·3)**	**11·4% (10·0–13·2)**	**14·2% (12·1–16·6)**	**0·7% (0·6–0·7)**	**0·3% (0·2–0·3)**	**4·8% (4·0–5·7)**	**5·1% (4·4–6·0)**	**0·4% (0·3–0·5)**
	Bangladesh	6·2% (5·4–7·0)	1·2% (1·0–1·5)	1·1% (0·9–1·3)	22·3% (19·2–25·4)	4·4% (3·5–5·4)	43·4% (40·7–46·2)	10·1% (8·8–11·5)	0·5% (0·4–0·5)	0·2% (0·1–0·2)	7·4% (6·3–8·6)	2·9% (2·4–3·6)	0·3% (0·3–0·4)
	Bhutan	9·8% (6·9–13·3)	15·8% (11·9–20·1)	5·1% (3·6–7·1)	50·0% (43·4–56·3)	0·5% (0·4–0·8)	9·8% (7·1–13·4)	7·8% (5·9–10·3)	0·5% (0·5–0·6)	0·1% (0·1–0·2)	0·2% (0·1–0·2)	0·2% (0·1–0·2)	0·1% (0·1–0·2)
	India	62·2% (58·0–66·0)	1·3% (0·9–1·7)	3·3% (2·6–4·4)	1·0% (0·7–1·3)	0·5% (0·4–0·7)	7·7% (6·0–9·8)	13·9% (11·3–16·7)	0·7% (0·6–0·8)	0·2% (0·2–0·3)	4·7% (3·8–5·9)	4·1% (3·2–5·1)	0·4% (0·3–0·6)
	Nepal	25·3% (23·3–27·3)	8·6% (7·5–9·8)	3·6% (3·1–4·2)	26·0% (23·7–28·3)	8·5% (7·5–9·5)	9·4% (8·3–10·6)	6·9% (5·9–8·0)	0·6% (0·6–0·7)	0·2% (0·2–0·3)	1·2% (1·0–1·5)	9·5% (8·1–11·1)	0·2% (0·2–0·3)
	Pakistan	24·8% (21·5–28·1)	0·5% (0·4–0·7)	6·3% (4·9–7·9)	9·9% (7·7–12·6)	1·6% (1·2–2·1)	5·6% (4·3–7·1)	26·2% (22·7–29·7)	0·9% (0·8–1·0)	1·2% (0·9–1·6)	2·5% (1·9–3·2)	20·1% (17·3–23·5)	0·5% (0·4–0·6)
**Southeast Asia, east Asia, and Oceania**		**25·5% (21·7–29·5)**	**3·6% (2·5–5·1)**	**37·6% (33·5–41·6)**	**6·9% (6·4–7·5)**	**6·8% (5·2–8·9)**	**7·2% (6·4–8·1)**	**8·4% (6·8–10·2)**	**0·7% (0·6–0·8)**	**0·1% (0·1–0·2)**	**1·3% (1·2–1·5)**	**1·5% (1·4–1·7)**	**0·3% (0·2–0·4)**
East Asia		29·8% (25·0–35·1)	4·5% (3·1–6·5)	44·8% (39·4–49·9)	0·7% (0·5–1·1)	6·8% (4·6–9·5)	2·8% (1·9–3·9)	8·9% (6·9–11·2)	0·7% (0·6–0·8)	0·1% (0·1–0·1)	0·4% (0·3–0·5)	0·2% (0·2–0·3)	0·3% (0·2–0·4)
	China	30·5% (25·5–35·9)	4·7% (3·2–6·6)	44·2% (38·7–49·5)	0·6% (0·4–1·0)	6·6% (4·3–9·3)	2·8% (1·9–4·0)	9·0% (6·9–11·4)	0·7% (0·6–0·8)	0·1% (0·1–0·1)	0·3% (0·2–0·5)	0·2% (0·2–0·3)	0·3% (0·2–0·4)
	North Korea	2·4% (1·8–3·1)	0·2% (0·1–0·3)	90·0% (88·8–91·2)	0·3% (0·2–0·5)	0·2% (0·1–0·3)	0·6% (0·5–0·9)	1·0% (0·8–1·3)	1·7% (1·5–1·9)	0·3% (0·2–0·4)	2·6% (2·0–3·3)	0·5% (0·3–0·6)	0·2% (0·1–0·2)
	Taiwan (province of China)	6·9% (5·0–9·4)	0·4% (0·3–0·6)	45·7% (41·2–50·2)	5·7% (4·0–7·8)	27·8% (23·3–32·2)	1·2% (0·9–1·6)	9·6% (7·6–11·8)	1·2% (1·0–1·5)	0·1% (0·1–0·1)	0·6% (0·5–0·9)	0·5% (0·4–0·6)	0·1% (0·1–0·2)
Oceania		21·1% (19·0–23·5)	1·5% (1·2–1·9)	2·1% (1·8–2·5)	22·6% (20·2–25·3)	23·9% (21·5–26·4)	7·6% (6·3–8·8)	5·2% (4·5–5·9)	1·2% (1·1–1·4)	1·1% (0·8–1·4)	6·8% (5·8–8·1)	3·6% (3·0–4·2)	3·4% (2·7–4·2)
	American Samoa	9·9% (6·8–13·7)	0·3% (0·2–0·5)	1·3% (1·0–1·8)	41·5% (35·8–47·0)	34·8% (29·6–40·3)	4·0% (2·8–5·5)	4·4% (3·3–5·9)	0·7% (0·6–0·9)	0·1% (0·1–0·2)	1·1% (0·8–1·5)	1·4% (1·0–1·9)	0·4% (0·3–0·5)
	Cook Islands	4·3% (3·0–5·9)	0·3% (0·2–0·5)	1·3% (0·8–2·2)	34·6% (30·6–38·3)	41·5% (37·7–45·4)	11·8% (9·5–14·3)	1·4% (1·0–2·0)	0·7% (0·6–0·8)	0·1% (0·1–0·1)	0·1% (0·1–0·2)	1·5% (1·1–2·2)	2·3% (1·8–2·8)
	Federated States of Micronesia	19·1% (13·5–25·8)	0·8% (0·6–1·2)	2·7% (1·9–3·8)	39·7% (31·6–47·6)	15·6% (11·3–20·9)	7·9% (5·4–11·4)	3·8% (2·7–5·1)	1·6% (1·3–2·0)	0·5% (0·4–0·7)	3·7% (2·6–5·0)	3·1% (2·2–4·4)	1·3% (1·0–1·8)
	Fiji	23·7% (18·8–29·4)	0·3% (0·2–0·4)	5·5% (3·9–7·7)	7·4% (5·2–10·2)	26·9% (22·0–32·5)	9·9% (7·3–13·0)	19·6% (16·1–23·3)	1·0% (0·9–1·2)	0·2% (0·2–0·2)	1·7% (1·3–2·4)	3·3% (2·4–4·4)	0·4% (0·3–0·5)
	Guam	6·1% (4·4–8·2)	0·2% (0·1–0·3)	0·8% (0·6–1·1)	38·9% (35·4–42·1)	44·2% (40·9–47·6)	2·7% (2·0–3·7)	5·0% (3·8–6·5)	0·5% (0·4–0·5)	0·1% (0·0–0·1)	0·5% (0·4–0·6)	0·9% (0·7–1·1)	0·2% (0·1–0·2)
	Kiribati	17·4% (14·2–21·2)	1·1% (0·8–1·5)	1·9% (1·4–2·6)	25·6% (21·1–30·7)	28·8% (24·5–33·3)	4·0% (2·9–5·6)	2·0% (1·5–2·6)	1·6% (1·4–1·8)	0·9% (0·7–1·3)	8·1% (6·3–10·2)	4·7% (3·6–6·2)	3·9% (3·0–4·8)
	Marshall Islands	46·0% (39·3–52·3)	0·5% (0·4–0·7)	1·6% (1·1–2·4)	24·8% (19·1–31·1)	13·7% (9·9–18·4)	4·2% (2·9–6·0)	5·0% (3·4–7·3)	0·8% (0·7–1·0)	0·4% (0·3–0·5)	1·9% (1·4–2·6)	0·8% (0·5–1·1)	0·3% (0·2–0·3)
	Nauru	25·7% (19·8–32·3)	0·5% (0·4–0·6)	7·0% (4·8–9·9)	8·3% (5·6–12·2)	22·1% (16·7–27·9)	3·2% (2·2–4·5)	8·8% (6·5–11·8)	1·5% (1·2–1·7)	2·6% (2·0–3·4)	11·0% (8·7–13·8)	8·0% (6·0–10·6)	1·3% (1·0–1·8)
	Niue	10·0% (7·0–13·7)	0·3% (0·2–0·5)	1·2% (0·9–1·7)	41·5% (36·1–46·9)	35·8% (30·7–41·1)	3·7% (2·7–5·0)	4·1% (3·1–5·4)	0·7% (0·6–0·8)	0·1% (0·1–0·1)	1·0% (0·7–1·3)	1·3% (1·0–1·7)	0·3% (0·3–0·5)
	Northern Mariana Islands	7·5% (5·1–10·4)	0·2% (0·2–0·3)	0·9% (0·7–1·3)	39·3% (34·6–43·7)	41·8% (37·5–46·0)	2·9% (2·1–4·1)	4·8% (3·5–6·3)	0·5% (0·4–0·7)	0·1% (0·1–0·1)	0·6% (0·4–0·9)	1·0% (0·8–1·4)	0·2% (0·2–0·3)
	Palau	7·3% (4·6–11·1)	0·4% (0·3–0·6)	2·7% (1·9–3·7)	18·1% (13·8–23·3)	26·6% (21·3–32·9)	32·4% (26·6–38·4)	2·5% (1·9–3·3)	1·7% (1·4–2·0)	0·2% (0·2–0·3)	1·7% (1·1–2·5)	0·5% (0·4–0·6)	5·9% (4·5–7·4)
	Papua New Guinea	20·8% (17·9–23·7)	1·9% (1·4–2·4)	1·2% (0·9–1·5)	23·6% (20·3–27·1)	24·7% (21·7–28·0)	6·8% (5·3–8·5)	2·7% (2·1–3·4)	1·2% (1·1–1·4)	1·3% (1·0–1·7)	7·9% (6·5–9·7)	3·5% (2·8–4·5)	4·4% (3·4–5·4)
	Samoa	19·7% (14·9–25·2)	0·7% (0·5–0·9)	1·6% (1·2–2·1)	42·8% (35·3–50·1)	1·4% (1·1–1·8)	19·5% (15·1–24·6)	1·7% (1·3–2·3)	3·4% (2·7–4·2)	0·8% (0·7–1·0)	3·7% (2·7–4·9)	3·1% (2·3–4·2)	1·4% (1·1–1·9)
	Solomon Islands	28·3% (23·3–33·5)	1·5% (1·1–2·1)	6·0% (4·5–7·8)	27·9% (22·4–33·7)	8·6% (6·2–11·5)	3·4% (2·4–4·6)	6·0% (4·8–7·6)	2·0% (1·7–2·3)	0·4% (0·3–0·5)	9·0% (7·1–11·3)	5·7% (4·4–7·3)	1·1% (0·9–1·5)
	Tokelau	11·8% (8·1–16·4)	0·4% (0·3–0·6)	1·5% (1·1–2·2)	42·3% (35·7–48·6)	30·9% (25·1–37·1)	4·6% (3·1–6·6)	3·9% (2·9–5·2)	0·8% (0·7–1·0)	0·2% (0·1–0·2)	1·4% (1·0–1·9)	1·6% (1·2–2·2)	0·5% (0·4–0·7)
	Tonga	30·3% (25·8–35·0)	0·7% (0·5–0·9)	6·7% (5·0–8·9)	20·8% (16·5–25·9)	13·3% (10·6–16·9)	6·3% (4·6–8·3)	3·0% (2·2–4·1)	3·5% (3·0–4·2)	1·2% (0·9–1·5)	4·4% (3·2–5·7)	7·7% (5·9–9·9)	2·1% (1·6–2·9)
	Tuvalu	8·2% (5·3–12·1)	1·0% (0·7–1·5)	4·8% (3·1–6·8)	48·5% (40·7–55·6)	19·2% (14·0–25·5)	3·3% (2·1–5·1)	2·7% (1·8–4·0)	1·7% (1·4–2·1)	0·7% (0·5–1·1)	3·5% (2·2–5·3)	2·7% (1·9–4·0)	3·6% (2·7–4·8)
	Vanuatu	21·2% (16·8–25·9)	1·0% (0·7–1·4)	5·9% (4·3–8·0)	21·2% (16·1–26·9)	1·3% (0·8–2·0)	24·3% (19·1–29·8)	9·4% (7·6–11·5)	1·4% (1·2–1·7)	0·9% (0·7–1·3)	7·6% (5·8–9·6)	4·8% (3·5–6·3)	1·0% (0·7–1·4)
Southeast Asia		10·1% (9·4–10·8)	0·4% (0·4–0·5)	12·6% (11·4–13·8)	28·8% (27·0–30·6)	6·6% (5·6–7·7)	22·9% (21·2–24·5)	6·7% (6·0–7·5)	0·6% (0·6–0·7)	0·2% (0·2–0·3)	4·7% (4·2–5·3)	6·1% (5·5–6·8)	0·3% (0·3–0·4)
	Cambodia	5·2% (3·7–6·9)	0·3% (0·2–0·4)	6·5% (4·8–8·6)	24·2% (18·7–29·4)	4·7% (3·2–6·5)	28·9% (23·7–34·4)	5·1% (3·8–6·6)	0·8% (0·6–0·9)	0·2% (0·1–0·2)	5·2% (3·9–6·8)	18·9% (15·2–23·6)	0·2% (0·2–0·3)
	Indonesia	5·5% (4·6–6·6)	0·3% (0·2–0·4)	7·3% (6·0–8·8)	46·7% (43·0–50·3)	10·3% (8·2–12·8)	19·0% (16·0–22·2)	3·4% (2·8–4·1)	0·5% (0·4–0·6)	0·2% (0·1–0·2)	2·0% (1·6–2·5)	4·4% (3·6–5·3)	0·4% (0·3–0·5)
	Laos	7·7% (6·5–9·1)	0·2% (0·2–0·3)	3·6% (2·9–4·5)	25·6% (22·1–29·7)	2·7% (2·1–3·3)	47·0% (43·4–50·7)	2·8% (2·3–3·3)	0·7% (0·6–0·7)	0·8% (0·6–1·1)	6·3% (5·2–7·5)	2·1% (1·7–2·6)	0·4% (0·3–0·5)
	Malaysia	7·8% (5·3–10·8)	0·5% (0·3–0·7)	2·8% (1·9–4·0)	22·8% (16·6–29·4)	22·5% (17·5–28·0)	23·8% (18·8–29·4)	13·1% (10·1–16·6)	0·7% (0·6–1·0)	0·1% (0·1–0·1)	1·9% (1·3–2·6)	3·5% (2·4–4·8)	0·4% (0·3–0·6)
	Maldives	18·8% (14·1–23·7)	1·3% (0·9–1·9)	2·7% (1·7–4·0)	8·9% (5·9–12·8)	2·9% (1·9–4·3)	16·9% (12·2–22·0)	28·6% (23·2–34·6)	1·1% (1·0–1·3)	0·4% (0·3–0·6)	4·1% (3·0–5·6)	11·3% (8·7–14·6)	3·0% (2·2–4·1)
	Mauritius	11·5% (8·4–15·2)	0·3% (0·2–0·4)	3·0% (2·2–4·1)	5·8% (3·9–8·2)	2·6% (1·7–3·6)	16·8% (13·0–20·9)	18·4% (14·8–22·4)	0·8% (0·7–0·9)	0·1% (0·1–0·2)	5·1% (3·7–6·7)	34·3% (28·9–39·6)	1·5% (1·1–2·0)
	Myanmar	8·7% (6·4–11·5)	1·0% (0·7–1·5)	4·6% (3·5–6·0)	55·2% (49·7–60·4)	2·1% (1·4–2·9)	23·7% (19·4–28·4)	1·7% (1·3–2·3)	0·6% (0·5–0·7)	0·2% (0·1–0·2)	0·8% (0·6–1·1)	0·9% (0·6–1·2)	0·4% (0·3–0·5)
	Philippines	14·5% (11·9–17·2)	0·3% (0·2–0·4)	6·6% (5·0–8·5)	10·5% (8·5–12·8)	2·2% (1·7–2·8)	33·4% (29·0–37·6)	4·8% (3·8–5·9)	0·9% (0·8–1·1)	0·6% (0·5–0·8)	8·8% (6·8–11·0)	17·0% (13·9–20·3)	0·5% (0·4–0·7)
	Seychelles	10·2% (7·5–13·8)	0·5% (0·4–0·7)	5·1% (3·7–6·9)	36·1% (30·4–42·1)	17·8% (13·4–22·8)	15·8% (12·0–20·3)	7·4% (5·7–9·5)	1·1% (0·9–1·5)	0·2% (0·1–0·2)	1·9% (1·4–2·7)	3·5% (2·5–4·8)	0·3% (0·2–0·3)
	Sri Lanka	21·5% (18·3–24·7)	0·6% (0·4–0·8)	13·8% (11·3–16·3)	15·8% (12·8–19·1)	7·5% (6·1–9·3)	12·1% (10·0–14·5)	11·1% (9·4–13·1)	0·6% (0·5–0·7)	0·1% (0·1–0·2)	10·6% (8·4–13·1)	6·1% (4·8–7·7)	0·1% (0·1–0·2)
	Thailand	31·4% (29·0–33·9)	0·9% (0·6–1·2)	1·0% (0·7–1·4)	15·8% (13·9–17·9)	2·8% (2·3–3·4)	39·9% (37·6–42·2)	4·1% (3·3–5·0)	0·9% (0·8–1·0)	0·2% (0·1–0·2)	1·0% (0·8–1·2)	1·8% (1·4–2·2)	0·2% (0·2–0·2)
	Timor-Leste	3·5% (2·3–5·1)	0·6% (0·4–0·8)	4·4% (3·2–6·0)	55·2% (48·0–62·4)	18·2% (13·8–22·8)	7·3% (5·2–9·8)	0·7% (0·5–1·0)	2·2% (1·8–2·7)	0·8% (0·6–1·1)	3·2% (2·3–4·4)	3·0% (2·1–4·1)	0·8% (0·6–1·1)
	Vietnam	4·3% (3·0–6·0)	0·3% (0·2–0·4)	41·9% (36·8–47·2)	3·0% (2·0–4·2)	0·7% (0·4–1·0)	14·2% (11·3–17·7)	16·8% (13·4–20·3)	0·5% (0·4–0·6)	0·3% (0·2–0·3)	11·5% (9·0–14·4)	6·5% (4·8–8·6)	0·1% (0·1–0·2)
**Sub-Saharan Africa**		**3·3% (3·1–3·6)**	**0·5% (0·4–0·5)**	**2·3% (2·1–2·4)**	**34·1% (33·3–34·9)**	**17·0% (16·5–17·6)**	**12·0% (11·6–12·4)**	**15·2% (14·6–15·8)**	**1·8% (1·7–1·9)**	**1·6% (1·5–1·8)**	**6·8% (6·4–7·2)**	**4·0% (3·7–4·4)**	**1·4% (1·3–1·5)**
Central sub-Saharan Africa		1·2% (0·9–1·5)	0·4% (0·3–0·5)	1·2% (1·0–1·4)	11·5% (10·2–13·0)	8·6% (7·4–9·9)	10·6% (9·5–11·7)	26·0% (24·0–28·0)	2·2% (2·0–2·3)	3·5% (2·9–4·2)	22·0% (20·1–24·2)	9·9% (8·5–11·5)	3·1% (2·7–3·6)
	Angola	0·6% (0·5–0·9)	0·6% (0·5–0·8)	1·7% (1·3–2·2)	27·6% (22·5–33·2)	5·2% (3·7–7·4)	16·9% (13·6–21·0)	37·1% (31·9–42·6)	1·9% (1·6–2·2)	0·7% (0·5–0·9)	4·7% (3·6–6·1)	2·1% (1·6–2·7)	0·9% (0·7–1·2)
	Central African Republic	1·4% (1·0–1·8)	0·7% (0·5–1·0)	0·8% (0·6–1·0)	10·5% (8·3–13·3)	8·6% (6·9–10·5)	26·3% (22·4–30·7)	13·2% (10·8–16·2)	2·9% (2·6–3·3)	12·5% (10·1–15·4)	19·9% (16·1–23·9)	2·0% (1·5–2·6)	1·3% (1·0–1·6)
	Congo (Brazzaville)	0·6% (0·4–0·8)	0·3% (0·2–0·4)	0·4% (0·3–0·5)	8·2% (6·1–10·8)	1·6% (1·2–2·1)	10·4% (8·3–12·8)	40·0% (35·5–44·5)	2·0% (1·7–2·2)	1·2% (0·9–1·7)	26·1% (22·4–30·1)	5·7% (4·3–7·6)	3·6% (3·0–4·3)
	Democratic Republic of the Congo	1·3% (1·0–1·7)	0·3% (0·3–0·4)	1·2% (0·9–1·5)	9·0% (7·6–10·6)	10·1% (8·5–11·7)	8·7% (7·5–10·2)	22·2% (19·9–24·7)	2·2% (2·0–2·4)	3·9% (3·1–4·8)	25·2% (22·6–28·0)	12·2% (10·4–14·2)	3·6% (3·0–4·3)
	Equatorial Guinea	3·1% (2·0–4·9)	0·6% (0·4–0·8)	2·7% (1·9–3·7)	25·6% (20·1–32·1)	4·2% (2·8–6·3)	14·9% (11·7–18·8)	33·3% (27·5–39·6)	3·1% (2·6–3·6)	1·6% (1·1–2·3)	5·5% (4·2–7·2)	4·4% (3·2–5·9)	0·9% (0·6–1·2)
	Gabon	1·4% (0·8–2·1)	0·3% (0·2–0·3)	0·4% (0·3–0·5)	1·8% (1·3–2·7)	0·8% (0·6–1·0)	12·1% (9·4–15·3)	55·8% (50·8–60·5)	2·2% (1·9–2·5)	1·6% (1·2–2·1)	18·3% (15·0–21·8)	4·5% (3·5–5·8)	1·0% (0·8–1·2)
Eastern sub-Saharan Africa		4·0% (3·6–4·3)	0·4% (0·4–0·4)	2·5% (2·3–2·7)	46·0% (44·9–47·2)	21·5% (20·6–22·3)	8·7% (8·1–9·3)	7·2% (6·7–7·8)	1·3% (1·2–1·4)	1·1% (1·0–1·2)	4·2% (3·9–4·5)	2·4% (2·2–2·7)	0·7% (0·7–0·8)
	Burundi	1·9% (1·3–2·6)	0·9% (0·7–1·2)	3·9% (2·8–5·2)	43·8% (37·8–49·9)	17·8% (14·1–22·1)	7·1% (5·4–9·4)	6·0% (4·6–7·4)	1·6% (1·4–2·0)	0·9% (0·7–1·1)	8·4% (6·4–10·6)	7·1% (5·3–9·1)	0·7% (0·5–1·0)
	Comoros	3·7% (2·3–5·7)	0·5% (0·4–0·7)	1·0% (0·7–1·3)	39·0% (30·9–47·4)	12·8% (8·9–17·3)	13·4% (9·2–18·3)	10·1% (7·6–13·0)	1·8% (1·4–2·2)	2·0% (1·5–2·8)	8·1% (6·0–10·8)	6·8% (5·1–8·8)	0·7% (0·6–1·0)
	Djibouti	1·6% (1·1–2·4)	0·9% (0·7–1·1)	1·8% (1·3–2·5)	41·9% (34·7–49·4)	4·6% (3·3–6·2)	37·7% (30·3–44·8)	3·9% (2·7–5·6)	2·9% (2·4–3·6)	2·3% (1·7–3·1)	1·0% (0·7–1·4)	0·8% (0·6–1·1)	0·6% (0·4–0·7)
	Eritrea	2·6% (1·7–4·0)	1·2% (0·9–1·6)	4·0% (2·8–5·7)	31·9% (23·4–41·5)	3·4% (2·4–4·9)	20·3% (14·8–27·3)	12·5% (9·5–16·3)	3·8% (3·1–4·6)	10·2% (7·3–14·0)	7·2% (5·2–9·8)	1·7% (1·2–2·4)	1·0% (0·8–1·4)
	Ethiopia	1·2% (0·9–1·5)	0·4% (0·3–0·5)	3·0% (2·5–3·5)	60·4% (58·1–62·6)	22·6% (20·9–24·5)	5·5% (4·6–6·6)	1·3% (1·1–1·6)	1·2% (1·1–1·3)	0·8% (0·7–1·0)	2·5% (2·1–2·9)	0·7% (0·6–0·9)	0·5% (0·4–0·6)
	Kenya	3·9% (3·2–4·6)	0·3% (0·2–0·3)	4·4% (3·6–5·2)	38·4% (36·0–41·0)	31·9% (29·6–34·1)	8·7% (7·2–10·4)	6·6% (5·7–7·7)	1·1% (1·0–1·3)	0·6% (0·4–0·7)	2·8% (2·3–3·4)	0·7% (0·5–0·8)	0·7% (0·6–0·9)
	Madagascar	1·2% (0·9–1·7)	0·3% (0·3–0·4)	1·6% (1·3–2·0)	58·6% (55·3–61·6)	14·9% (13·1–16·8)	10·0% (8·3–11·7)	1·7% (1·3–2·1)	1·0% (0·9–1·1)	0·8% (0·6–1·0)	8·6% (7·0–10·3)	0·9% (0·7–1·2)	0·4% (0·3–0·5)
	Malawi	13·6% (11·1–16·3)	0·3% (0·3–0·4)	1·1% (0·8–1·4)	52·0% (47·4–56·6)	19·3% (15·8–22·9)	3·5% (2·6–4·6)	5·9% (4·6–7·2)	1·5% (1·3–1·8)	0·3% (0·2–0·3)	0·7% (0·5–0·9)	1·1% (0·8–1·5)	0·8% (0·6–1·0)
	Mozambique	1·0% (0·7–1·5)	0·4% (0·3–0·5)	1·2% (0·9–1·6)	46·8% (41·5–52·2)	4·5% (3·4–5·9)	21·2% (17·4–25·4)	18·3% (14·9–22·0)	1·3% (1·1–1·4)	2·0% (1·5–2·7)	1·5% (1·2–1·9)	0·5% (0·4–0·7)	1·3% (1·0–1·7)
	Rwanda	2·6% (2·1–3·1)	0·5% (0·4–0·6)	2·3% (1·9–2·8)	29·7% (26·0–33·4)	37·2% (34·2–40·1)	9·8% (8·3–11·3)	6·5% (5·6–7·6)	1·3% (1·1–1·5)	0·5% (0·4–0·6)	5·5% (4·7–6·4)	4·0% (3·4–4·8)	0·4% (0·3–0·5)
	Somalia	2·3% (1·5–3·4)	2·0% (1·4–2·8)	1·8% (1·3–2·4)	2·5% (1·7–3·8)	3·1% (2·2–4·2)	5·7% (4·3–7·5)	1·5% (1·2–2·0)	13·4% (11·5–15·7)	20·4% (16·1–25·6)	43·8% (38·4–49·4)	1·8% (1·3–2·4)	1·7% (1·3–2·4)
	South Sudan	3·4% (2·2–5·2)	3·0% (2·2–4·2)	2·5% (1·8–3·5)	15·3% (10·6–21·5)	2·8% (1·9–4·1)	5·8% (4·0–8·2)	9·5% (7·1–12·7)	8·1% (6·9–9·5)	13·0% (9·6–17·4)	28·5% (22·3–35·4)	3·9% (2·8–5·4)	4·0% (2·9–5·3)
	Tanzania	6·9% (5·3–8·7)	0·4% (0·3–0·5)	1·6% (1·2–2·0)	34·5% (30·6–38·6)	19·5% (16·9–23·0)	9·3% (7·4–11·6)	11·9% (9·8–14·3)	1·1% (0·9–1·2)	1·1% (0·8–1·4)	6·6% (5·5–7·9)	6·3% (5·1–7·7)	0·9% (0·7–1·1)
	Uganda	5·4% (4·5–6·5)	0·4% (0·3–0·5)	2·2% (1·8–2·7)	42·3% (39·3–45·4)	20·3% (17·8–22·7)	5·7% (4·7–6·9)	12·1% (10·4–14·0)	1·1% (0·9–1·2)	0·9% (0·7–1·1)	3·9% (3·3–4·8)	4·8% (4·1–5·7)	0·8% (0·6–1·0)
	Zambia	2·7% (2·1–3·4)	0·3% (0·2–0·3)	1·3% (1·0–1·6)	45·2% (42·2–48·4)	16·2% (14·4–18·1)	16·1% (13·6–19·0)	11·1% (9·4–13·1)	0·8% (0·7–0·9)	1·2% (0·9–1·5)	1·2% (0·9–1·5)	3·4% (2·6–4·5)	0·6% (0·5–0·8)
Southern sub-Saharan Africa		6·7% (5·5–8·0)	0·5% (0·4–0·6)	1·4% (1·2–1·7)	40·8% (38·2–43·6)	8·5% (7·4–9·6)	18·7% (16·9–20·4)	21·4% (19·7–23·3)	0·7% (0·7–0·8)	0·2% (0·2–0·2)	0·2% (0·2–0·3)	0·5% (0·5–0·7)	0·3% (0·2–0·3)
	Botswana	3·3% (2·3–4·5)	0·2% (0·2–0·2)	2·6% (1·9–3·4)	25·8% (22·1–29·7)	1·5% (1·1–2·2)	15·9% (13·1–18·9)	49·4% (45·3–53·6)	0·5% (0·5–0·6)	0·1% (0·1–0·1)	0·3% (0·2–0·4)	0·3% (0·2–0·3)	0·2% (0·1–0·2)
	eSwatini	4·0% (3·0–5·3)	0·3% (0·2–0·4)	0·9% (0·7–1·3)	35·3% (31·5–39·3)	4·5% (3·6–5·8)	11·7% (9·3–14·0)	40·6% (37·2–44·1)	0·7% (0·6–0·8)	0·3% (0·3–0·4)	0·3% (0·2–0·3)	0·8% (0·6–1·1)	0·5% (0·4–0·6)
	Lesotho	2·2% (1·7–2·7)	0·3% (0·2–0·4)	2·4% (1·9–2·9)	37·8% (35·5–40·2)	6·7% (5·9–7·7)	19·5% (17·7–21·4)	28·8% (26·4–31·3)	0·7% (0·7–0·8)	0·2% (0·1–0·2)	0·4% (0·3–0·5)	0·5% (0·4–0·7)	0·5% (0·4–0·6)
	Namibia	5·8% (4·5–7·5)	0·3% (0·2–0·4)	1·1% (0·8–1·5)	46·5% (42·4–50·4)	0·6% (0·5–0·8)	9·1% (7·6–11·1)	34·1% (30·3–38·1)	1·3% (1·2–1·5)	0·2% (0·1–0·2)	0·3% (0·2–0·3)	0·3% (0·2–0·4)	0·4% (0·3–0·5)
	South Africa	8·5% (6·8–10·3)	0·6% (0·4–0·8)	1·5% (1·2–1·9)	47·6% (44·0–51·3)	7·3% (6·0–8·7)	11·1% (9·2–13·1)	21·7% (19·4–24·3)	0·7% (0·6–0·7)	0·2% (0·1–0·2)	0·2% (0·2–0·3)	0·4% (0·3–0·6)	0·2% (0·2–0·3)
	Zimbabwe	1·5% (1·1–2·1)	0·2% (0·2–0·3)	0·7% (0·6–1·0)	16·9% (13·9–20·1)	16·5% (13·8–19·7)	52·2% (47·7–56·2)	9·1% (7·8–10·6)	0·8% (0·7–0·9)	0·4% (0·3–0·5)	0·3% (0·2–0·3)	1·1% (0·8–1·4)	0·3% (0·3–0·4)
Western sub-Saharan Africa		1·6% (1·4–1·8)	0·6% (0·6–0·7)	2·8% (2·5–3·1)	22·4% (21·4–23·6)	18·0% (17·1–18·9)	13·8% (13·0–14·5)	19·3% (18·1–20·8)	2·9% (2·7–3·0)	2·4% (2·2–2·7)	8·1% (7·5–8·8)	5·9% (5·3–6·6)	2·2% (2·0–2·5)
	Benin	1·1% (0·8–1·5)	0·6% (0·5–0·8)	4·7% (3·6–6·1)	15·8% (12·6–19·3)	25·6% (21·9–29·6)	8·7% (6·8–10·8)	17·4% (13·9–21·5)	2·3% (2·0–2·7)	1·5% (1·2–2·0)	13·8% (11·2–17·1)	6·5% (5·0–8·5)	1·8% (1·4–2·3)
	Burkina Faso	0·6% (0·4–0·7)	0·4% (0·3–0·5)	2·3% (1·8–3·0)	26·7% (23·2–30·4)	42·6% (38·8–46·6)	10·0% (8·2–12·1)	10·8% (9·1–13·0)	1·2% (1·0–1·3)	0·7% (0·6–0·9)	3·6% (2·9–4·5)	0·6% (0·4–0·7)	0·6% (0·4–0·7)
	Cameroon	1·1% (0·8–1·6)	0·4% (0·3–0·5)	1·4% (1·1–1·8)	12·9% (10·8–15·3)	7·4% (6·1–8·9)	5·9% (4·9–7·3)	40·9% (36·9–45·0)	1·9% (1·7–2·1)	3·3% (2·6–4·2)	19·4% (16·5–22·5)	3·2% (2·5–4·0)	2·1% (1·7–2·6)
	Cape Verde	9·8% (7·6–12·5)	0·2% (0·1–0·2)	2·4% (1·8–3·3)	34·6% (29·6–39·3)	4·6% (3·3–6·3)	23·2% (19·4–27·2)	21·3% (17·8–25·2)	0·9% (0·8–1·0)	0·1% (0·1–0·2)	1·3% (1·0–1·8)	0·9% (0·7–1·1)	0·7% (0·5–0·8)
	Chad	2·7% (2·0–3·6)	1·4% (1·1–1·8)	1·7% (1·2–2·2)	31·9% (27·1–37·0)	12·6% (9·7–15·9)	6·2% (4·6–8·2)	5·5% (4·0–7·3)	5·5% (4·8–6·1)	19·0% (15·2–23·4)	10·2% (8·3–12·7)	1·7% (1·3–2·2)	1·7% (1·3–2·2)
	Côte d'Ivoire	0·8% (0·6–1·0)	0·9% (0·7–1·2)	0·7% (0·6–1·0)	24·0% (20·8–27·4)	7·2% (5·7–8·9)	24·7% (21·4–28·2)	21·6% (18·7–24·9)	4·4% (3·9–5·0)	1·6% (1·2–2·0)	10·8% (9·0–12·8)	1·1% (0·8–1·4)	2·2% (1·8–2·8)
	The Gambia	2·9% (2·1–3·7)	0·9% (0·7–1·2)	3·3% (2·5–4·3)	41·3% (37·8–45·1)	25·0% (22·1–28·1)	12·4% (9·4–15·9)	2·9% (2·3–3·6)	3·0% (2·6–3·4)	1·3% (1·0–1·7)	2·0% (1·6–2·5)	1·8% (1·4–2·4)	3·2% (2·5–4·0)
	Ghana	3·7% (2·9–4·5)	0·4% (0·3–0·5)	2·0% (1·6–2·5)	27·7% (24·9–30·9)	21·8% (19·3–24·5)	14·8% (12·8–17·0)	9·7% (8·1–11·6)	4·6% (4·1–5·1)	1·0% (0·8–1·3)	9·8% (8·2–11·4)	3·2% (2·7–3·9)	1·1% (0·9–1·4)
	Guinea	1·4% (1·0–1·8)	1·3% (1·0–1·6)	2·2% (1·7–2·9)	23·3% (19·2–27·7)	12·8% (10·3–15·6)	16·7% (13·9–20·1)	14·4% (11·8–17·4)	2·8% (2·5–3·1)	16·5% (13·3–19·8)	4·9% (3·9–6·1)	1·6% (1·2–2·1)	2·1% (1·7–2·7)
	Guinea-Bissau	0·6% (0·4–0·8)	0·3% (0·3–0·4)	16·5% (14·5–18·7)	4·5% (3·6–5·7)	40·7% (37·8–43·8)	4·2% (3·4–5·1)	17·2% (14·3–20·5)	1·8% (1·7–2·0)	9·2% (7·7–10·7)	3·8% (3·0–4·7)	0·4% (0·3–0·5)	0·8% (0·7–1·0)
	Liberia	1·0% (0·7–1·3)	0·5% (0·4–0·6)	0·6% (0·4–0·8)	54·6% (51·3–57·8)	16·9% (14·6–19·3)	13·7% (11·6–16·1)	6·5% (5·1–8·2)	1·6% (1·5–1·8)	0·5% (0·4–0·7)	2·7% (2·2–3·3)	1·0% (0·8–1·3)	0·4% (0·3–0·5)
	Mali	1·6% (1·2–2·1)	0·7% (0·5–0·9)	2·5% (1·9–3·2)	34·4% (30·1–39·2)	36·0% (31·7–40·3)	15·5% (12·7–18·8)	1·7% (1·2–2·3)	2·3% (2·1–2·7)	1·3% (1·0–1·6)	1·2% (0·9–1·5)	1·0% (0·8–1·3)	1·8% (1·4–2·3)
	Mauritania	1·4% (1·0–2·0)	0·8% (0·6–1·1)	3·3% (2·3–4·7)	22·4% (16·8–28·7)	6·5% (4·5–9·2)	44·3% (37·2–51·0)	5·8% (4·5–7·3)	3·2% (2·8–3·7)	0·7% (0·5–1·0)	1·1% (0·8–1·5)	9·5% (7·1–12·8)	0·9% (0·7–1·2)
	Niger	1·1% (0·7–1·6)	0·5% (0·3–0·7)	2·5% (1·9–3·4)	20·1% (16·4–24·7)	21·4% (17·3–25·8)	43·0% (37·7–48·0)	1·2% (0·8–1·7)	1·7% (1·4–1·9)	3·4% (2·6–4·5)	1·2% (0·9–1·5)	0·6% (0·4–0·8)	3·3% (2·5–4·4)
	Nigeria	1·5% (1·2–1·8)	0·7% (0·6–0·9)	3·2% (2·7–3·9)	18·6% (16·5–20·9)	13·4% (12·1–14·9)	11·1% (9·7–12·6)	24·3% (21·8–27·1)	3·1% (2·8–3·5)	2·3% (1·9–2·9)	8·2% (7·1–9·4)	10·7% (9·4–12·1)	2·9% (2·5–3·4)
	São Tomé and Príncipe	1·2% (0·9–1·6)	0·5% (0·4–0·6)	2·7% (2·2–3·5)	37·0% (33·7–40·6)	6·0% (4·8–7·4)	30·7% (27·5–33·7)	14·7% (12·2–17·7)	1·6% (1·4–1·8)	0·5% (0·4–0·6)	2·8% (2·2–3·5)	0·7% (0·5–0·8)	1·6% (1·3–2·0)
	Senegal	2·2% (1·8–2·8)	0·6% (0·4–0·7)	6·6% (5·6–7·8)	30·3% (27·7–33·0)	32·6% (30·3–35·1)	15·1% (13·1–17·5)	4·9% (4·1–5·8)	1·4% (1·2–1·5)	0·5% (0·4–0·6)	2·5% (2·1–3·1)	0·9% (0·7–1·1)	2·3% (1·9–2·8)
	Sierra Leone	0·8% (0·6–1·0)	0·4% (0·3–0·5)	1·1% (0·9–1·3)	42·5% (39·6–45·5)	27·3% (24·7–29·9)	20·9% (18·5–23·4)	1·6% (1·3–2·1)	1·5% (1·4–1·7)	1·1% (0·9–1·4)	0·7% (0·5–0·8)	0·6% (0·4–0·7)	1·5% (1·2–1·8)
	Togo	1·8% (1·3–2·4)	0·6% (0·4–0·8)	2·1% (1·6–2·9)	27·3% (23·2–31·9)	19·4% (15·9–23·5)	8·8% (7·1–10·9)	27·8% (23·6–32·5)	2·0% (1·8–2·3)	1·4% (1·0–1·8)	7·0% (5·5–8·7)	1·0% (0·8–1·3)	0·8% (0·6–1·1)

SDI= Socio-demographic Index.

* Diaphragm, emergency contraception, and other modern methods that were estimated separately were combined into a single column (other modern methods) since these prevalence estimates tend to be small (prevalence <5%).

### Demand satisfied and mCPR by age and SDI

**Figure 4:**
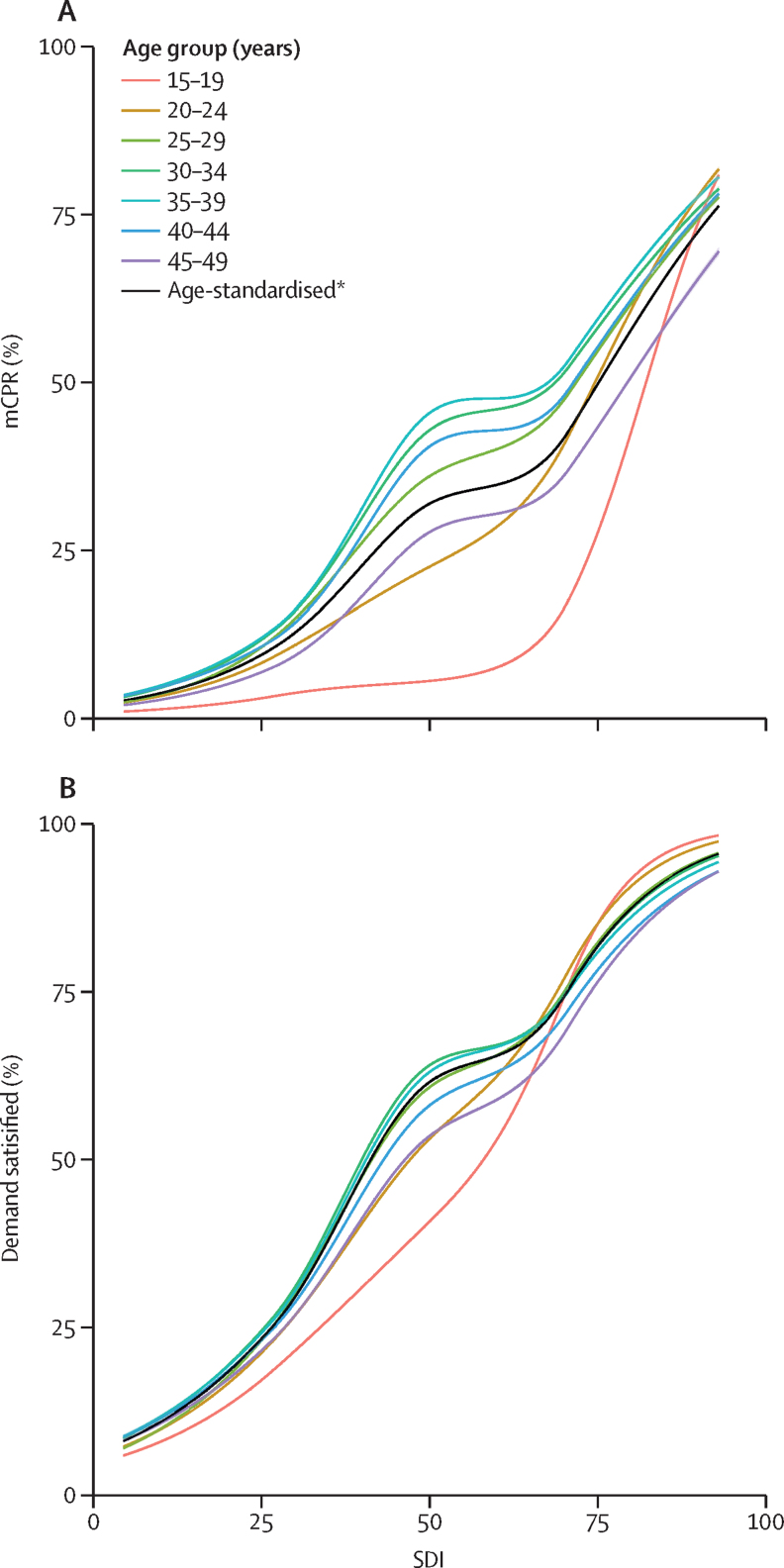
Expected mCPR (A) and demand satisfied (B) by SDI for different age groups, 1970–2019 mCPR=modern contraceptive prevalence rate. SDI=Socio-demographic Index. *Age-standardised represents the aggregated estimates for ages 15–49 years using a standard age structure for all locations.

Relative to SDI levels, patterns in the mCPR and demand satisfied varied substantially by age group between 1970 and 2019 (figure 4). For the older age groups (25–49 years), the largest increases in the mCPR corresponded with an increase in SDI of 25 to 50, with a second acceleration in the 75 to 100 SDI range. However, for women aged 15–19 years, increases occurred at the highest levels of social and economic development, as countries moved from approximately 60 to 100 SDI. A similar gap in rates of demand satisfied was observed between the 15–19 age group and other age groups in the middle SDI range, with 15–19 age group rates reaching other age group rates only at higher levels of SDI.

## Discussion

### Main findings

Since 1970, the use of contraception has increased considerably worldwide, underpinned by a global transition from traditional methods to more effective modern methods. However, global family planning goals have yet to be fully realised. In 2019, 1·176 billion women (1·163–1·189) had need for contraception, of whom 162·9 million (95% UI 155·6–170·2) had unmet need. Between 2012 and 2019, there were 69·0 million (51·3–85·9) additional users of modern contraception in the 69 FP2020 countries, 51·0 million (34·2–68·7) short of meeting the FP2020 goal of 120 million additional users over this period.^16^ Globally, nearly 60% of women with unmet need resided in sub-Saharan Africa and south Asia, and 43 million women with unmet need were aged 15–24 years (26·5% of global unmet need). This is notable because, in south Asia, more than 50% of users rely on female sterilisation, which is unlikely to appeal to younger women. In 28 individual countries, one method was similarly dominant. Country-by-country analysis of the specific groups with unmet need and the methods most suited to their life stage can support the development of family planning policies.

The FP2020 shortfall was evident despite major investments in family planning made by donors such as the Bill & Melinda Gates Foundation and the US and UK Governments. $3·7 billion in development assistance for health was invested in FP2020 countries between 2012 and 2020. Although substantial increases in the mCPR were observed in many of the 69 countries, increases largely fell short of the targets set by the initiative. Analysis of the best performing countries and the years in which targets were met, accounting for age, marital status, and contraceptive method mix, could support future investments aimed at increasing contraceptive coverage.

A large proportion of women aged 15–24 years had unmet need, consistent with other analyses that focused solely on adolescents.^27^ The level of unmet need in this age group is concerning because unintended pregnancies before the age of 25 years can hinder or eliminate education and employment opportunities, which promote social and economic empowerment later in life.^5^, ^6^, ^7^, ^8^, ^9^, ^10^, ^11^, ^12^ Globally, the largest gaps were among young, partnered women. Most pregnancies in the 15–19 year age group occurred among partnered women, and around half of pregnancies in this age group were unintended.^52^ Absence of contraceptive supplies tailored to the needs of younger age groups might explain unmet need in this group. Female sterilisation, which is common in south Asia, is unlikely to appeal to young women who have yet to have children. The broader social and economic context is also relevant. In some communities, marriage and childbearing are viewed as means of attaining social and economic security,^53^, ^54^ and thus young, partnered women might not be empowered to access contraception, particularly if their personal choices, empowerment, and autonomy are restricted and preferences for contraceptive use and desire for children differ from that of their partner.

Differences in contraceptive method mix across super-regions, age groups, and marital status were significant and associated with family planning policies and programmes. For example, in sub-Saharan Africa, organisations such as Marie Stopes International have promoted implants, explaining in part the larger proportion of women using those methods in the region when compared with other areas.^55^ Condom use has also been promoted strongly as part of international initiatives to address the HIV/AIDS epidemic, particularly in sub-Saharan Africa.^56^, ^57^ In India, the larger proportion of women using female sterilisation is likely to be associated with incentives for sterilisation provided to some groups by the Indian Government.^58^, ^59^, ^60^

Our results show that one method comprised nearly 50% of contraceptive use in south Asia and 28 countries. Dependence on a single method is theorised to restrict access and constrain the mCPR, because some women might prefer a method that is not widely available.^40^, ^61^ In fact, existing evidence shows that the mCPR is higher in areas where more methods are available.^41^ Unmet need might be due to suitable methods not being available, including for past users of contraception, who have been shown to account for a substantial proportion of unmet need in some settings.^62^ Availability of existing contraceptive methods relies on existing infrastructure and knowledge, thus, launching a new contraceptive method is not a trivial undertaking, requiring additional training of providers, supply chain changes, and other activities. The higher prevalence of specific methods in some geographies thus raises the question of whether family planning programmes should focus on expanding method mix or, alternatively, making accepted methods more widely available in these settings.

Family planning programmes should consider barriers to access specific to the method preferred by the groups with the most unmet need. From our analysis, younger women were more likely than older women to use short-term methods that require less contact with providers, such as the pill and condoms. In many settings, the pill is of higher cost than other methods, potentially posing an important barrier to satisfying demand among younger women.^63^ Older women are more likely to use contraceptives that are long-acting and require a medical procedure, such as female sterilisation. These methods cost less in many settings because they are provided through government health facilities and thereby subsidised. Beyond costs, different methods are usually provided by different sources (eg, the public *vs* private sectors, in pharmacies), which has implications for travel time, privacy, and other access barriers.^42^, ^64^ It might be more socially acceptable for married women to access medical care required for long-acting contraceptive methods. Scarcity of information might also be an important factor for users identifying methods that address cultural barriers and placate concerns about fertility. Further research into preferences by age and marital status could elucidate whether expanding method mix would best reduce unmet need or whether financial and physical barriers that result in inequitable access are more important. Demand-side programmes that improve knowledge of contraceptive methods, side-effects and efficacy, and the risk of pregnancy, are also fundamental to reducing unmet need for contraception.

We identified an association between both the mCPR and demand satisfied with SDI levels across countries, with the mCPR and demand satisfied among women aged 15–19 years increasing to the same levels as other age groups when SDI values increased above 60. This finding suggests that investments in access, quality, and other family planning programme efforts during the phase of rapid increase in the mCPR have not reached younger women to the same degree as older women. The inability to generate increases in contraception use and demand satisfied in younger age groups might translate into delays in countries realising the social and economic benefits of education and work experience possible when younger women postpone childbearing through use of contraception.

A key future direction is estimating the impact of the COVID-19 pandemic on family planning indicators. Reports from administrative bodies and health systems and early modelling efforts indicated large disruptions in family planning services occurred in 2020.^34^, ^36^, ^37^, ^65^, ^66^ However, women of reproductive age in phone interviews in four sub-Saharan African countries and surveys of smartphone users around the world did not report declines in demand satisfied.^35^, ^67^, ^68^, ^69^ It is possible that initial reports of disruptions prompted action that ultimately ensured women had continued access to their contraceptive method of choice. Alternatively, the reduction of physical contact during social distancing mandates might have reduced need. The COVID-19 pandemic could prompt longer-term changes in need and contraceptive use. An analysis has shown that the COVID-19 crisis tended to exacerbate existing gender disparities.^70^ The number of women who left the workforce and school was higher than that for men, partly due to increased care-taking duties amid school and childcare closures. If these changes are sustained, lack of employment and educational opportunities might change long-term fertility intentions and thus the need and use of contraception.^71^ Greater data granularity, information from a more representative set of contraceptive users, and detailed analysis should be immediate priorities for better understanding the impact of the pandemic on family planning.

### Limitations

Our analysis had a number of limitations. Major shortcomings exist with regard to the current definition of need used in our analysis, which was consistent with the SDG definition of demand satisfied.^17^ Definitions of need differ for partnered women versus unpartnered women, making demand satisfied potentially incomparable between these two groups. The algorithm does not entail asking women if they need or want contraception, but rather defines need based on whether partnered women do not want children and are not infecund. Only unpartnered women who report being sexually active and have these other characteristics are considered to have need. Women might be hesitant to report sexual activity to survey interviewers, and thus a reporting bias could have underestimated need among unpartnered women. This approach also does not account for the possibility that unpartnered women are not sexually active (within the past 4 weeks) because they do not have access to contraception. More generally, the unmet need metric falls short of fully capturing need by not taking into consideration what women intend to do. Some women classified as not having need for contraception report intent to use a method in the future;^72^ similarly, women with no intent to use a method might have been classified as needing contraception. The large proportion of women who were dissatisfied with their current method could be considered as having need.^73^, ^74^ The inconsistency between need and intent to use is also likely to differ depending on the age group, which affects our unmet need by age estimates. We did not disaggregate unmet need depending on whether a women had pregnancy spacing or limiting needs, which could be pertinent to the types of methods available.

More data were available for partnered women than unpartnered women, which resulted in more uncertainty in our family planning estimates for the unpartnered group. Third, some surveys were missing information on elements of need and methods of contraception. We used GBD crosswalking methods to adjust these biases with average differences, but this introduced more uncertainty into our estimates. Fourth, we were unable to estimate family planning indicators for some important groups. This includes women who are postpartum and the 10–14 year age group, both constituting potential users of contraception with special needs. Although data were available for the 15–19 year age group in many countries, there could be under-reporting of sexual activity or over-reporting of desire for children if a parent or partner were present during interviews. Data were more sparse in the earlier time periods (1970–1980), increasing uncertainty of estimates for that time period. Our uncertainty intervals did not capture sources of bias such as measurement bias, selection bias, or model specification bias. Although the ST-GPR approach closely follows data where they exist, some large and unexpected changes might be smoothed over if there are not other data nearby that follow a similar pattern.

## Conclusion

Despite the major improvements in family planning indicators observed since 1970, more than 160 million women remain with unmet need for contraception worldwide. More than 40 million women aged 15–24 years had unmet need, which is of crucial importance since the economic and social benefits of contraceptive access are likely to be most substantial in this age group. As family planning programmes assess their goals beyond 2020, they must consider how unmet need by age and marital status connect to the mix of methods available. By concentrating on the needs and barriers to use faced by the groups with the most unmet need, programmes can identify targeted strategies that bolster use of contraception and unlock its social and economic benefits.

## Data sharing

For detailed information regarding input data sources and to download the data used in these analyses, please visit the Global Health Data Exchange GBD 2019 website.

For the **Global Health Data Exchange GBD 2019 website** see http://ghdx.healthdata.org/gbd-2019

## Declaration of interests

NF reports other funding support from WHO as a consultant from June to September 2019 and Gates Ventures since 2020, all outside the submitted work. NJH reports grants or contracts from the Bill & Melinda Gates Foundation, outside the submitted work. All other authors declare no competing interests.
