# Supplementary material for: Measuring contraceptive method mix, prevalence, and demand satisfied by age and marital status in 204 countries and territories, 1970–2019: a systematic analysis for the Global Burden of Disease Study 2019
Source: Lancet. 2022 Jul 23;400(10348):295–327. doi: 10.1016/S0140-6736(22)00936-9 (PMC9304984; doi:10.1016/S0140-6736(22)00936-9)
Supplement: Supplementary appendix [file mmc1.pdf]

THE LANCET

Supplementary appendix

This appendix formed part of the original submission and has been peer reviewed. We post it as supplied by the authors.

Supplement to: Haakenstad A, Angelino O, Irvine C M S, et al. Measuring contraceptive method mix, prevalence, and demand satisfied by age and marital status in 204 countries and territories, 1970–2019: a systematic analysis for the Global Burden of Disease Study 2019. *Lancet* 2022; **400:** 295–327.

Appendix to Measuring contraceptive method mix, prevalence, and demand satisfied by age and marital status in 204 countries and territories, 1970-2019: a systematic analysis for the Global Burden of Disease Study 2019

This appendix provides further methodological detail and supplemental figures and tables.

Table of Contents

Author Contributions 3
Preamble 4
List of supplementary figures and tables 5
Figures 5
Tables 5
GATHER Statement 7
GATHER Checklist 8
Part 1. Estimation of indicators 10
Section 1. Overview 10
History of contraception in the GBD 10
Definitions of indicators 10
Section 2. Input data 12
Sources 12
Exclusion criteria 13
Processing of input data 18
Section 3. Modelling strategy 19
Flowchart 19
Overview 19
Marital-splitting 20
Crosswalking 20
Age-splitting 32
Modelling 32
Section 4. Post-modelling processing 45
Method mix squeezing 45
Age-standardisation 45
Global, super-regional and SDI quintile aggregation 45
Uncertainty calculation 46
Part 2. Additional analyses 46
Section 1. Evaluation of FP2020 Goal 46
Section 2. Expected values of mCPR and demand satisfied given SDI 50
Section 3. Comparison of IHME and UN-DESA Estimates 51
Overview 51
2019 Estimates 51
Definitions and Data Processing 52
Methods 53
Part 3. Online tools and glossary of terms 54
Section 1. Online tools 54
Section 2. List of abbreviations 54
Section 3. List of ISO3 code and location names 54
Part 4. Additional Supplementary Results 60
References 72

Author Contributions

Managing the estimation process

Annie Haakenstad, Rafael Lozano, Stephen Lim

Writing the first draft of the manuscript

Annie Haakenstad, Olivia Angelino

Providing data or critical feedback on data sources

Olivia Angelino, Kate Causey, Nancy Fullman, Caleb Irvine, Ira Martopullo, Everett Mumford

Extracting, cleaning, or cataloging data; designing or coding figures and tables

Olivia Angelino, Corinne Bintz, Caleb Irvine, Ira Martopullo, Thomas Glucksman

Drafting the work or revising is critically for important intellectual content

Zulfiqar Butta, Kelly Bienhoff, Kate Causey, Maegan Dirac, Nancy Fullman, Simon Hay, Nathaniel Henry, Stephen Lim, Ali H Mokdad, Everett Mumford, Christopher J L Murray

Managing the overall research enterprise

Christopher J L Murray, Rafael Lozano, Stephen Lim, Annie Haakenstad, Kelly Bienhoff

Preamble

This appendix provides methodological detail for estimating contraception coverage indicators as well as supplementary results. The appendix is organised into broad sections following the structure of the main paper. This study complies with the Guidelines for Accurate and Transparent Health Estimates Reporting (GATHER) recommendations. It includes detailed indicator modelling write-ups and flowcharts, and information on data sourcing to maximise transparency in our estimation processes and provides a comprehensive account of analytical steps. We intend this to be a living document, to be updated with each annual iteration of the Global Burden of Diseases, Injuries, and Risk Factors Study (GBD).

List of supplementary figures and tables

Figures

Supplementary Figure 1. Map of input data for marital status
Supplementary Figure 2. Map of input data for any contraceptive use, partnered women
Supplementary Figure 3. Map of input data for any contraceptive use, unpartnered women
Supplementary Figure 4. Map of input data for method breakdown, partnered women
Supplementary Figure 5. Map of input data for method breakdown, unpartnered women
Supplementary Figure 6. Map of input data for proportion of unmet need among partnered non-users
Supplementary Figure 7. Map of input data for proportion of unmet need among unpartnered non-users
Supplementary Figure 8. Map of contraceptive prevalence, 2019
Supplementary Figure 9. Map of the absolute change in contraceptive prevalence, 1970-2019

Supplementary Figure 10. Map of the absolute change in modern contraceptive prevalence, 1970-2019

Supplementary Figure 11. Map of the absolute change in demand satisfied with modern methods, 1970-2019

Supplementary Figure 12. Map of the absolute change in unmet need for any method, 1970-2019

Supplementary Figure 13. Demand satisfied by five-year age cohort and super-region, 1970-2019

Supplementary Figure 14. Input estimates for the expected change in modern contraceptive prevalence given SDI between 1970 and 2019

Supplementary Figure 15. Input estimates for the expected change in demand satisfied with modern methods given SDI between 1970 and 2019

Tables

Supplementary Table 1. GATHER checklist
Supplementary Table 2. Number of contraceptive use sources included by type
Supplementary Table 3. Number of surveys by missing component(s) of need algorithm
Supplementary Table 4. Reference and counterfactual scenarios for determining need
Supplementary Table 5. Crosswalk adjustment factors for proportion unmet need among partnered non-users
Supplementary Table 6. Crosswalk adjustment factors for proportion unmet need among unpartnered non-users
Supplementary Table 7. ST-GPR model coefficients for marital status
Supplementary Table 8. ST-GPR model coefficients for any contraceptive use among partnered women
Supplementary Table 9. ST-GPR model coefficients for any contraceptive use among unpartnered women
Supplementary Table 10. ST-GPR model coefficients for by-method use among partnered women
Supplementary Table 11. ST-GPR model coefficients for by-method use among unpartnered women
Supplementary Table 12. ST-GPR model coefficients for proportion unmet need among partnered non-users
Supplementary Table 13. ST-GPR model coefficients for proportion unmet need among unpartnered non-users

Supplementary Table 14. Age group weights for age-standardisation
Supplementary Table 15. SDI quintile cutoffs for GBD 2019
Supplementary Table 16. Projected and estimated annual percentage point changes in mCPR in 68 FP2020 priority countries
Supplementary Table 17. Average mCPR percentage point change between 2004-2011 and 2012-2019 in 68 FP2020 priority countries
Supplementary Table 18. Contraceptive methods in use as a share of contraceptive prevalence, 1970

GATHER Statement

This study complies with the Guidelines for Accurate and Transparent Health Estimates Reporting (GATHER) recommendations.[1] We have documented the steps involved in our analytical procedures and detailed the data sources used in compliance with the GATHER. For additional GATHER reporting, please refer to Appendix Table 1 on pages 7-8.

GATHER Checklist

Supplementary Table 1. ***GATHER checklist of information that should be included in reports of global health estimates, with description of compliance and location of information***

#	GATHER checklist item	Description of compliance	Reference
**Objectives and funding**			
**1**	Define the indicator(s), populations (including age, sex, and geographic entities), and time period(s) for which estimates were made.	Description of indicators, definitions, relevant time periods, and populations in paper and appendix.	Main Text Methods; Main Text, Table 1; Appendix, Part 1, Section 1
**2**	List the funding sources for the work.	Funding sources listed in paper.	Main Text Summary
**Data inputs**			
For all data inputs from multiple sources that are synthesised as part of the study:			
**3**	Describe how the data were identified and how the data were accessed.	Narrative description of data seeking methodology provided.	Appendix, Part 1, Section 2
**4**	Specify the inclusion and exclusion criteria. Identify all ad-hoc exclusions.	Narrative about inclusion and exclusion criteria by data type provided in linked materials.	Appendix, Part 1, Section 2; Linked to appendix with ad-hoc exclusions in cause-specific write-ups.
**5**	Provide information on all included data sources and their main characteristics. For each data source used, report reference information or contact name/institution, population represented, data collection method, year(s) of data collection, sex and age range, diagnostic criteria or measurement method, and sample size, as relevant.	An interactive, online data source tool that provides metadata for data sources by component, geography, cause, risk, or impairment.	Available pending publication at http://ghdx.healthdata.org/gbd-2019
**6**	Identify and describe any categories of input data that have potentially important biases (eg, based on characteristics listed in item 5).	Summary of known biases included in paper and appendix.	Main Text Methods; Appendix, Part 1, Section 3
For data inputs that contribute to the analysis but were not synthesised as part of the study:			
**7**	Describe and give sources for any other data inputs.	An interactive, online data source tool that provides metadata for data sources by component, geography, cause, risk, or impairment.	Appendix, Part 1, Section 2; Appendix Methods, Part 3; http://ghdx.healthdata.org/gbd-2019
For all data inputs:			
**8**	Provide all data inputs in a file format from which data can be efficiently extracted (eg, a spreadsheet as opposed to a PDF), including all relevant meta-data listed in item 5. For any data inputs that cannot be shared due to ethical or legal reasons, such as third-party ownership, provide a contact name or the name of the institution that retains the right to the data.	Downloads of input data are available through online tools, including data visualisation tools and data query tools. Input data is not available in tools but can be made available upon request.	Appendix Methods, Part 3; http://ghdx.healthdata.org/gbd-2019

**Data analysis**			
**9**	Provide a conceptual overview of the data analysis method. A diagram may be helpful.	Flow diagrams of the overall methodological processes, as well as cause-specific modelling processes have been provided.	Appendix, Part 1, Section 3
**10**	Provide a detailed description of all steps of the analysis, including mathematical formulae. This description should cover, as relevant, data cleaning, data pre-processing, data adjustments and weighting of data sources, and mathematical or statistical model(s).		Appendix, Part 1, Sections 2 and 3
**11**	Describe how candidate models were evaluated and how the final model(s) were selected.	Provided in the methodological write-ups.	Appendix Part 1, Section 3
**12**	Provide the results of an evaluation of model performance, if done, as well as the results of any relevant sensitivity analysis.	Provided in the methodological write-ups.	Appendix Part 1, Section 3
**13**	Describe methods for calculating uncertainty of the estimates. State which sources of uncertainty were, and were not, accounted for in the uncertainty analysis.	Provided in the methodological write-ups.	Appendix Methods, Part 1, Section 4
**14**	State how analytic or statistical source code used to generate estimates can be accessed.	Access statement provided.	Links to code will be updated at time of publication and can be found here: http://ghdx.healthdata.org/gbd-2019
**Results and Discussion**			
**15**	Provide published estimates in a file format from which data can be efficiently extracted.	GBD 2019 results are available through online data visualisation tools, the Global Health Data Exchange, and the online data query tool.	Online data tools will be updated at time of publication and findable at this link: http://ghdx.healthdata.org/gbd-2019
**16**	Report a quantitative measure of the uncertainty of the estimates (e.g., uncertainty intervals).	Uncertainty intervals are provided with all results.	Main text results, Main text tables 1 and 2.
**17**	Interpret results in light of existing evidence. If updating a previous set of estimates, describe the reasons for changes in estimates.		Main Text, Discussion
**18**	Discuss limitations of the estimates. Include a discussion of any modelling assumptions or data limitations that affect interpretation of the estimates.	Discussion of limitations provided in the main text.	Main Text, Methods and Discussion

Part 1. Estimation of indicators

Section 1. Overview

History of contraception in the GBD

Estimates of modern contraceptive prevalence and demand satisfied with modern methods for all women were first modelled for GBD 2010. In previous iterations of the modelling pipeline these estimates were modelled directly with no marital status breakdown nor a nested proportions modelling approach. This allowed inconsistencies to arise in final indicator estimates where modern contraceptive prevalence could surpass demand satisfied, which by definition is impossible, plus limited the range of indicators we could output. Additionally, surveys restricted to currently or ever-partnered women had to be crosswalked to all women increasing uncertainty. This is the first time GBD contraception estimates have been produced with a nested proportions modelling approach, by marital status, and with method-specific breakdowns.

Definitions of indicators

The population of interest for this study were women aged 15 to 49 years in 204 GBD countries and territories. Indicators were produced from 1970 to 2019 for all women 15-49, split into partnered and unpartnered women, and for each of the following 5-year age groups: 15-19, 20-24, 25-29, 30-34, 35-39, 40-44, and 45-49 in addition to age-standardised 15-49 estimates.

Marital status

The proportion of women currently partnered include women who self-identify as legally or formally married, in-union, living with a partner, or otherwise partnered. Unpartnered women include women who have never been married, or are divorced, separated, or widowed.

Any contraceptive prevalence

Any contraceptive prevalence is the proportion of women who are currently using, or whose partner is currently using, at least one method of contraception. Complete abstinence (different from periodic abstinence) is not considered a method of contraception but does play a role in determining need among un-partnered women.

Modern contraceptive prevalence

Modern contraceptive prevalence is the proportion of women who are currently using, or whose partner is currently using, at least one method of modern contraception. Modern methods include female or male sterilisation, oral contraceptive pills, male or female condoms, diaphragms, spermicides and sponges, hormonal or non-hormonal intrauterine devices (IUDs), implants, injections, contraceptive patches and rings, as well as emergency contraceptives. All other family planning methods were considered traditional, including lactational amenorrhoea method (LAM), withdrawal, calendar methods (rhythm or standard days), douches, periodic abstinence, herbs, and other methods. Our categorisation of LAM as traditional varies from UN-DESA, which considers the method to be modern.

Demand satisfied for family planning with modern methods

Demand satisfied with modern methods is the proportion of women with a need for family planning who are currently using, or whose partner is currently using, at least one method of modern contraception. This indicator is also known as SDG Indicator 3.7.1, a component of SDG Goal 3[1]:

> SDG Goal 3: *Ensure healthy lives and promote well-being for all at all ages*
>
> SDG Target 3.7: *By 2030, ensure universal access to sexual and reproductive health-care services, including for family planning, information and education, and the integration of reproductive health into national strategies and programmes*
>
> SDG Indicator 3.7.1: *The proportion of women of reproductive age (15 to 49 years) who are sexually active and have their need for family planning satisfied with modern methods*

Women were defined as having a need for family planning if they were using any method of contraception, or if they were fecund, sexually active, and did not wish to become pregnant within the next two years. Sexual activity was assumed for all currently partnered women. Women were assumed to be fecund unless they met one or more of the following criteria:

(1) they were pregnant;
(2) they were postpartum amenorrhoeic from a birth that occurred 5 or more years ago;
(3) they had not menstruated within the last 6 months (unless postpartum amenorrhoeic);
(4) they had been continuously married/in a union for 5 or more years without having a child and without ever having used any method of contraceptive (modern or traditional); or
(5) they otherwise indicated that they were infertile (ex. mentioned having had a hysterectomy).

Women who were pregnant or postpartum amenorrhoeic from a birth within the last 2 years were considered separately and were determined to have a need for contraception if they indicated a desire to have delayed or avoided their current or most recent pregnancy. For additional details on the algorithm for need see the DHS 2012 Revising Unmet Need for Family Planning publication[2].

Unmet need for family planning

Unmet need is the proportion of women who are not currently using, or whose partner is not currently using, at least one method of contraception and have a need for family planning. The denominator for unmet need is all women regardless of need status, not to be confused with demand satisfied where the denominator is only women with need for family planning. Additionally, in the indicator demand satisfied, traditional methods are considered unmet need, but they count as meeting need in the unmet need indicator.

Method mix

Method-specific prevalence is the proportion of women who are currently using, or whose partner is currently using, a particular method, and are not simultaneously using a method approximately more effective (see order of priority below). Method mix is the proportion of any contraceptive use each method comprises such that the sum of all methods is 100%. In instances where women or their partner were using more than one method, we followed the skip pattern used for Question 304 in the Phase 7 DHS Model Questionnaire for women to select a single method[3]. We also considered earlier phases of DHS in order to rank diaphragm which did not appear in the Phase 7 Questionnaire[4]. The ranking of methods is primarily based on method effectiveness with a few exceptions. Implants have higher effectiveness than IUDs and injections, however, we would expect and observed little to no instances in which a woman was using both of these methods at once[5]. Condoms are less effective than the diaphragm, but condoms are one of the most consistently methods asked about in surveys over time so they are prioritised over the diaphragm[5]. Methods were grouped and modelled in the following categories, in order of priority:

(1) Female sterilisation

(2) Male sterilisation

(3) IUDs

(4) Injections

(5) Implants

(6) Pill

(7) Condom (including male and female condoms)

(8) Diaphragm

(9) Emergency contraception

(10) Other modern (including contraceptive rings and patches, foams, jellies, sponges)

(11) Lactational amenorrhoea method (LAM)

(12) Rhythm (including calendar methods such as billings and standard days, periodic abstinence)

(13) Withdrawal

(14) Other traditional

Section 2. Input data

Sources

The present study used two primary types of input data:

(1) individual-level survey microdata and

(2) tabulated data from survey reports.

Our primary data sources included multi-country survey series such as Demographic and Health Surveys (DHS), Multiple Indicator Cluster Surveys (MICS), Centers for Disease Control and Prevention Reproductive Health Surveys (CDC RHS), and Performance Monitoring and Accountability 2020 (PMA2020) surveys. The main inclusion criteria for sources were that they contained self-reported use of contraception among women aged 15-49 and were nationally- or GBD subnationally-representative. The most comprehensive data sources reported contraceptive use for both partnered and unpartnered by age group, however, surveys with no marital status breakdown or age-aggregated data were still included and underwent additional processing to marital- and/or age-split estimates. Surveys conducted in-person, over the telephone or on the internet were all accepted. We currently do not incorporate administrative data.

In order to strengthen our marital status estimates, we sought additional sources that did not report on contraceptive use including censuses and other population-representative surveys. We downloaded the World Marriage Data 2019 dataset from https://www.un.org/development/desa/pd/data/world-marriage-data on 1/13/2021 and incorporated all census data for women aged 15-49 which was flagged as including consensual unions.

Exclusion criteria

The exclusion of surveys was considered on a case-by-case basis, following this list of criteria:

(1) Did not report on current use of contraception; survey did not contain information about contraceptive use, or only recorded knowledge of contraception or ever used contraception

(2) Not representative: survey was not representative of the entire location or population

(3) Biased sampling: survey only sampled girls in schools or women within a hospital/mall/urban area/etc.

(4) Excluded sub-groups of women: survey did not ask women who have never had sex or never menstruated about contraceptive use, etc.

(4) Not self-reported data

(5) Only interviewed males about contraceptive use

Supplementary Table 2. Number of contraceptive use sources included by type

Survey Series	Microdata	Reports	Total
DHS	314	10	324
MICS	210	31	234
PMA2020	61	0	58
Generations and Gender Survey	25	0	21
CDC RHS	39	28	67
World Fertility Survey	41	7	46
PAPFAM/PAPCHILD	21	1	22
Other/Country-specific surveys	103	271	357
**Total**	814	348	1162

Data for unpartnered women was more sparse as surveys on reproductive health and contraceptive use sometimes chose to only interview currently or ever-partnered women, which was common prior to 1990. Proportion of unmet need among non-users had considerably less input data than the any contraceptive prevalence and method-specific prevalence models, particularly for unpartnered women. Not all surveys which ask about contraceptive use also ask the subsequent questions necessary to determine need for family planning, hence the reduction in data. In the figures below, grey reflects locations in which we had no input data.

Supplementary Figure 1. Map of input data for marital status

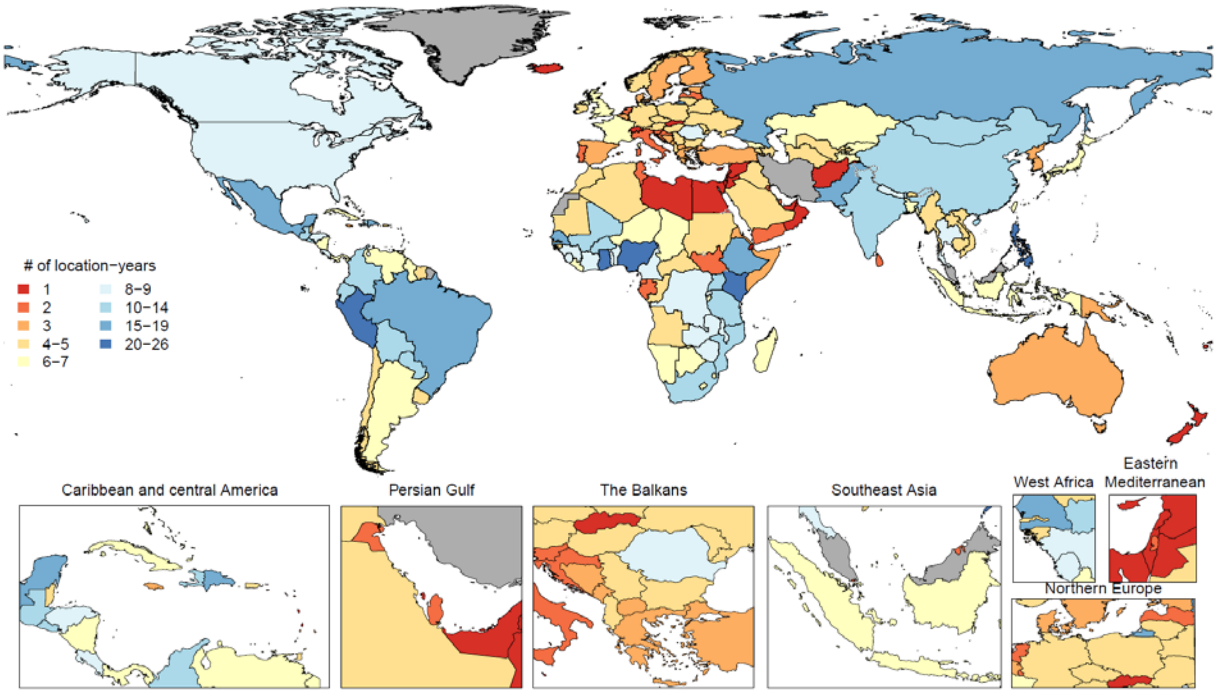

Supplementary Figure 2. Map of input data for any contraceptive use, partnered women

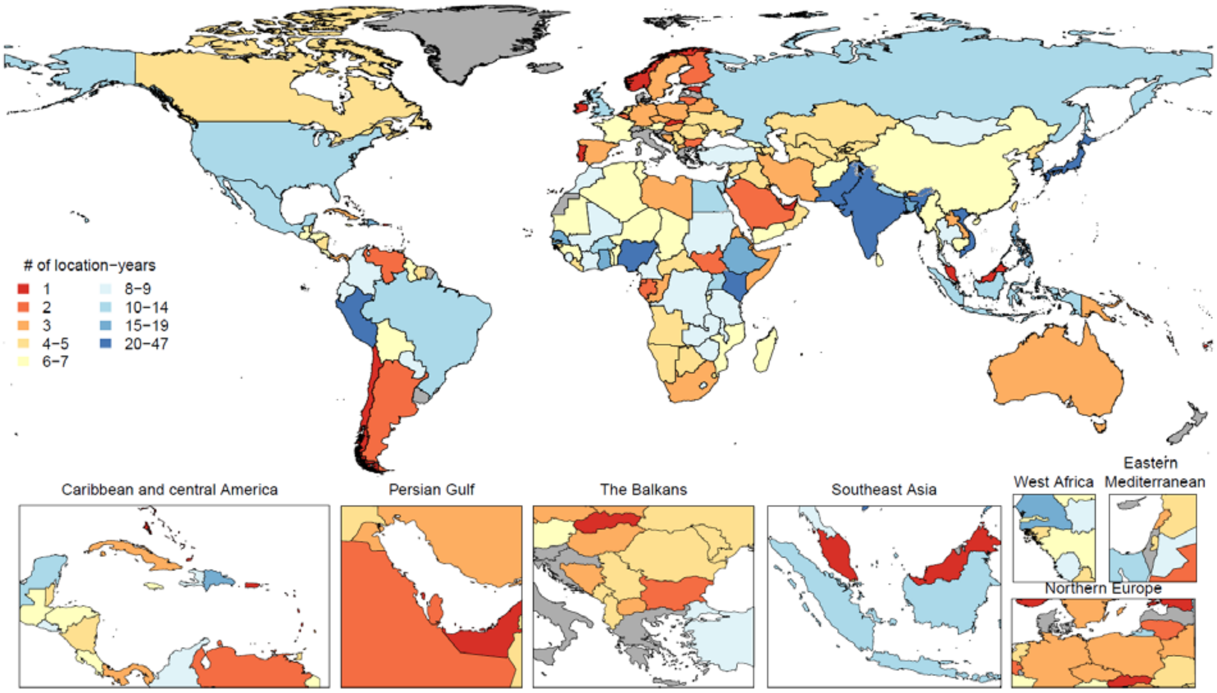

Supplementary Figure 3. Map of input data for any contraceptive use, unpartnered women

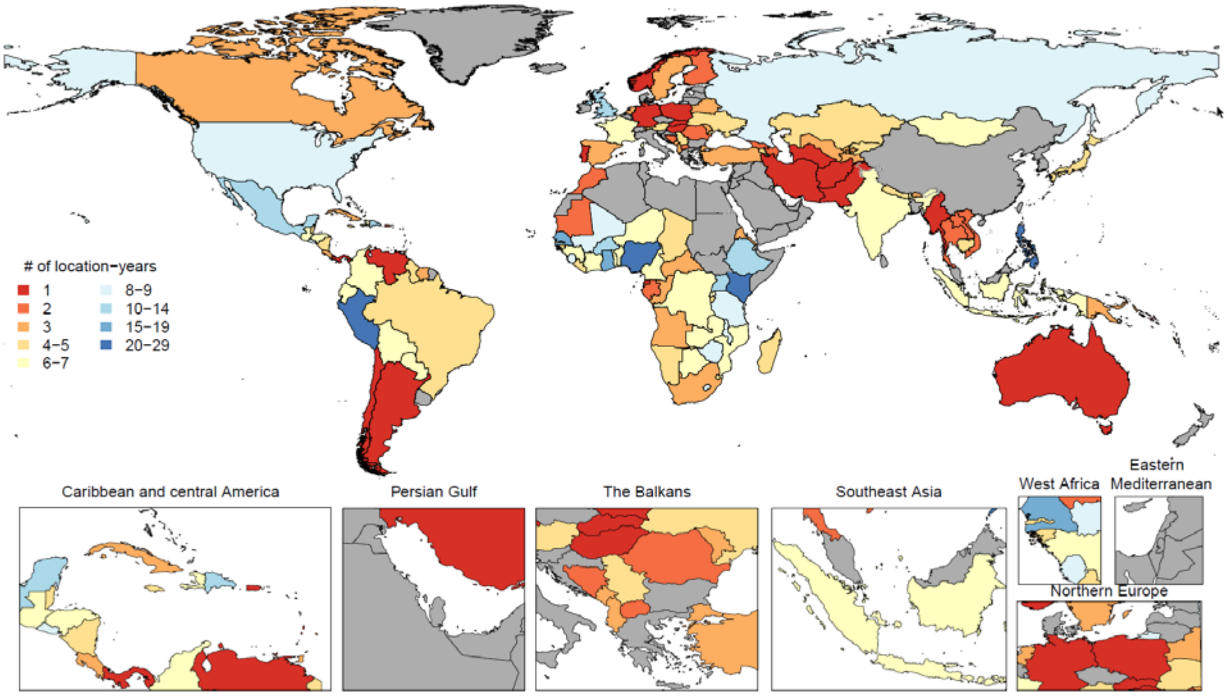

Supplementary Figure 4. Map of input data for method breakdown, partnered women

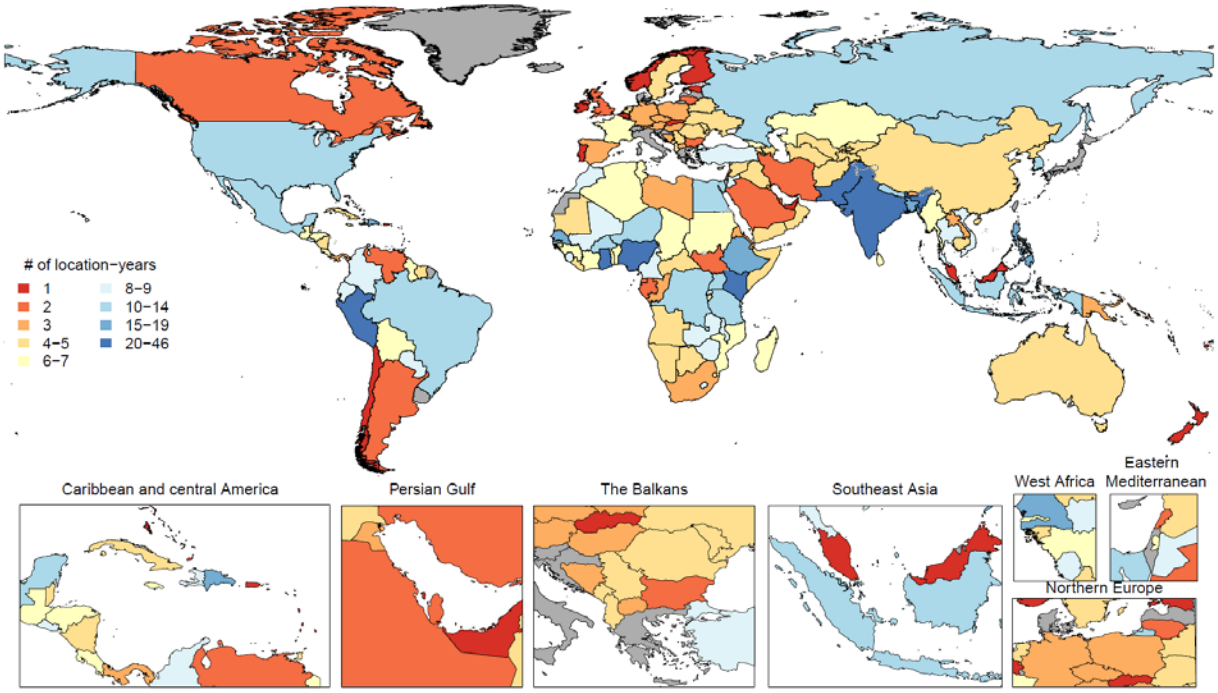

Supplementary Figure 5. Map of input data for method breakdown, unpartnered women

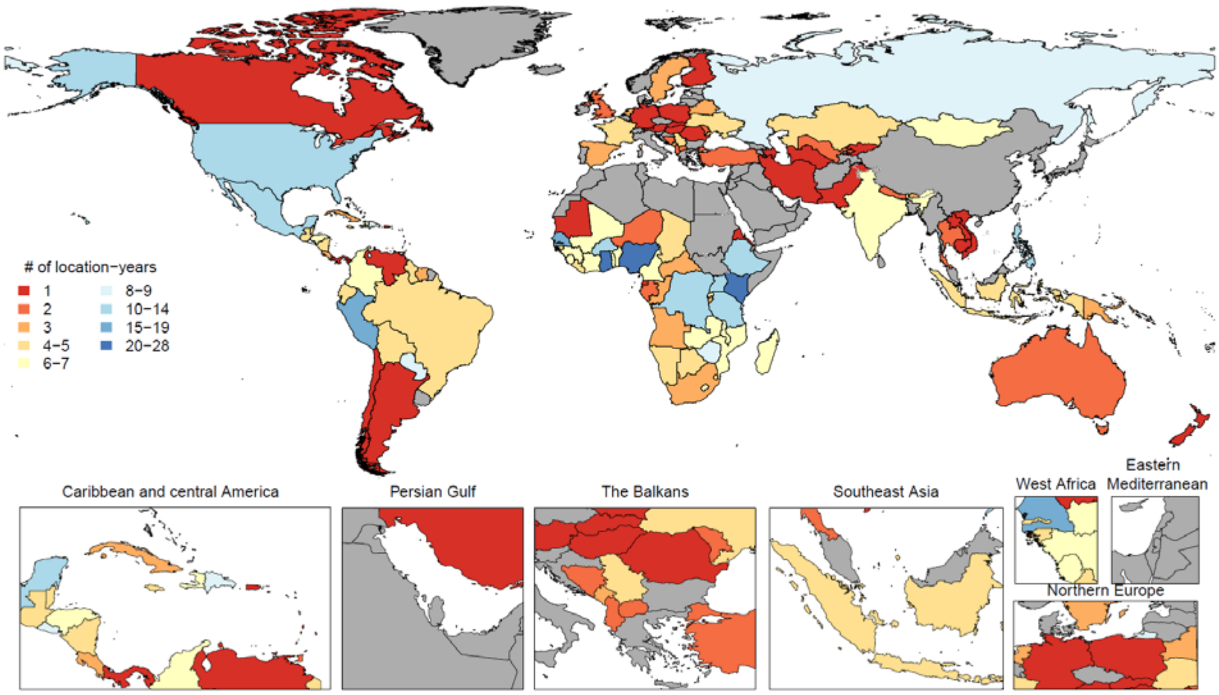

Supplementary Figure 6. Map of input data for proportion of unmet need among partnered non-users

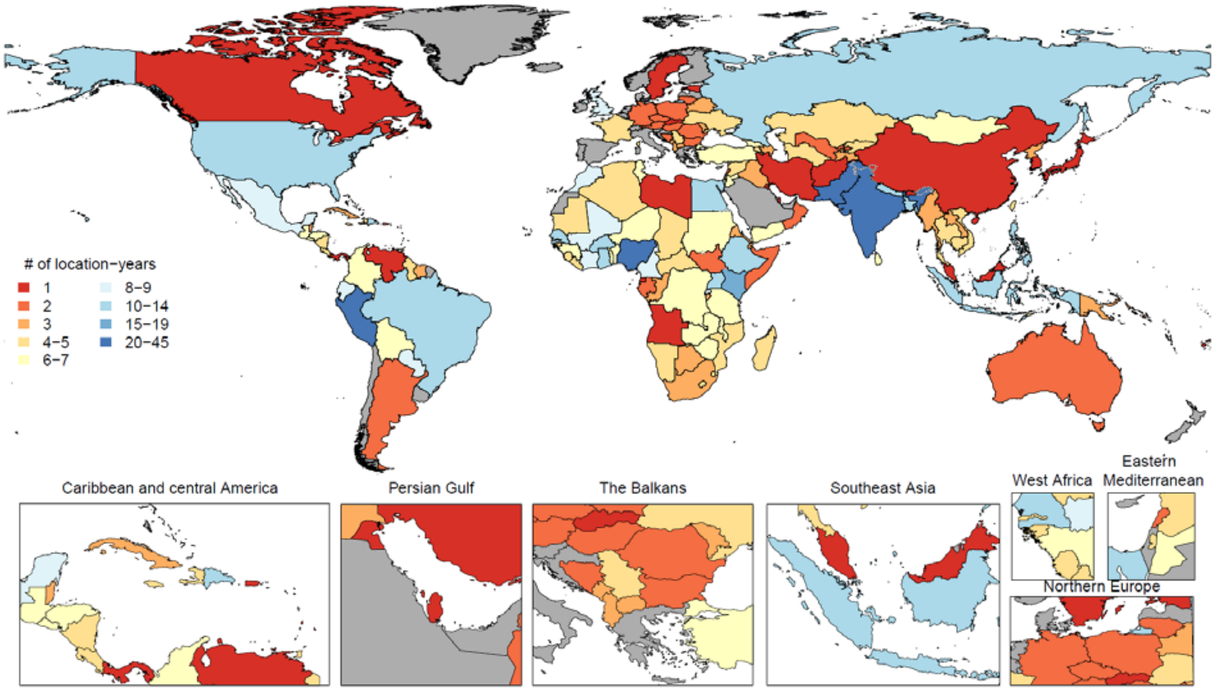

Supplementary Figure 7. Map of input data for proportion of unmet need among unpartnered non-users

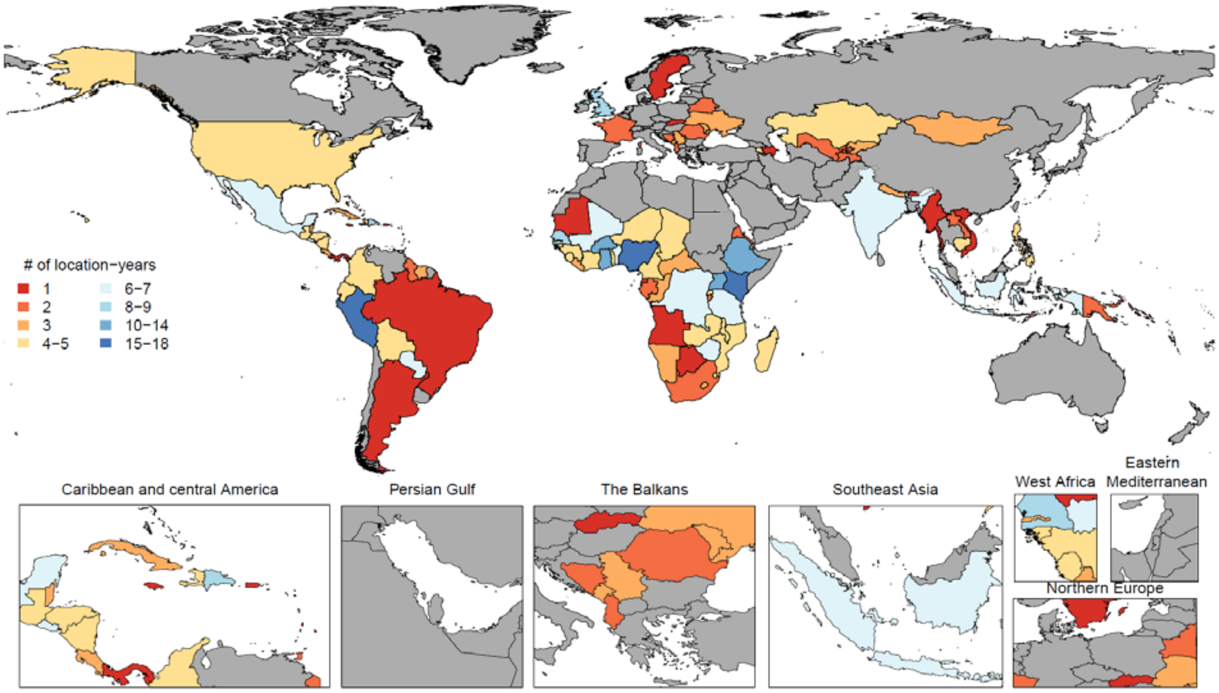

Processing of input data

Data points that covered 2 or fewer ages within a 5-year age group were dropped. This was most common for the 15-19 age group where surveys only interviewed women aged 18-19, which are likely to have differential contraceptive use rates relative to younger women.

In instances where tabulated reports did not provide sample sizes or only provided total population numbers, sample size was imputed using the 5th percentile of available sample sizes for each age group. For example, to impute missing sample sizes for any contraceptive use among partnered women aged 15-19, we took the 5th percentile of sample sizes that were available for that indicator and age group. We imputed sample size by age group because sample sizes vary by age and partner status; we would expect larger sample sizes in the older partnered age groups than the younger age groups and vice versa for unpartnered women. We used the 5th percentile to be conservative – potentially inflating the errors. When tabulated reports included data for all women and partnered women for the same overlapping age groups, we back-calculated quantities of interest for unpartnered women.

Among the surveys for which we had access to microdata, we applied survey weights based on survey sampling frames made available by data collection agencies to generate weighted national or subnational estimates accompanied by estimates of the standard error (SE). In instances where we had microdata, we took into account the effect of clustering by multiplying our standard errors by the square root of the design effect.

In the absence of microdata or survey sampling information, we used survey sample sizes as a mechanism for informing uncertainty estimation via the formula for standard error of the binomial mean:

$$Standard\ error = \sqrt{\frac{p(1-p)}{n}}$$

where p is the indicator proportion and n is sample size.

We predicted any contraceptive use when surveys only reported modern contraceptive prevalence by running a linear regression on all surveys which reported both. Regressions had a fixed effect on age group and a random effect on GBD super-region. Regressions were run separately for all, partnered and unpartnered women data. This allowed us to retain the unmet need data from those surveys as we modelled unmet need as a proportion of non-users which required any contraceptive use to calculate.

Section 3. Modelling strategy

Flowchart

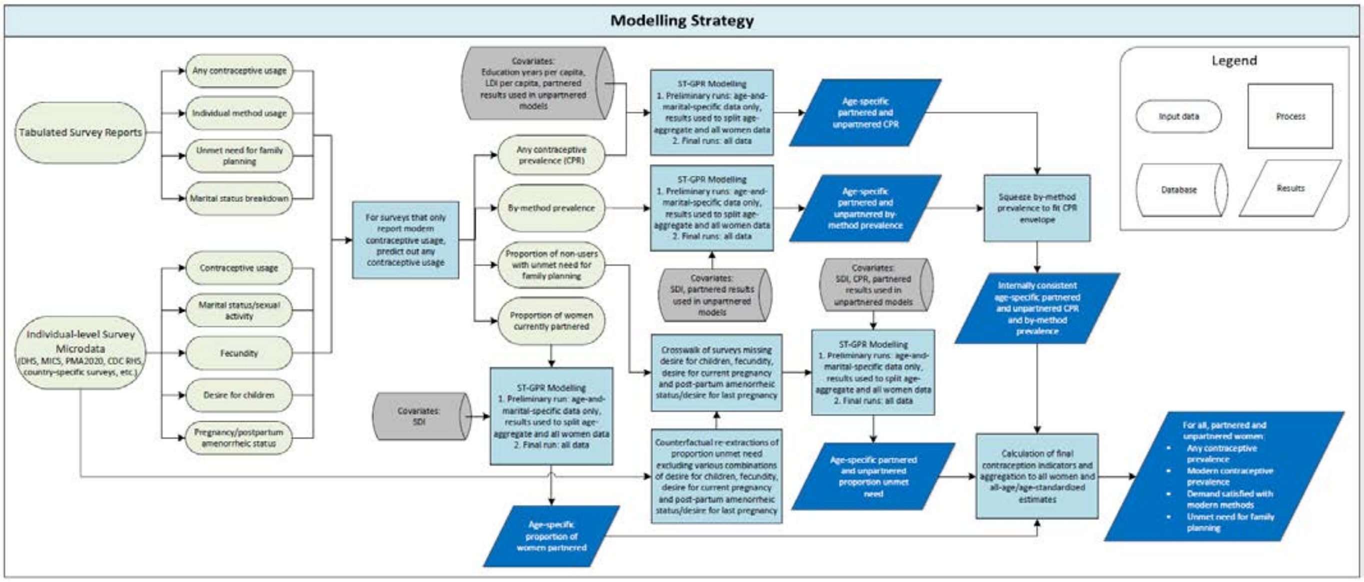

CPR = any contraceptive prevalence
LDI = Lag-distributed Income
SDI = Socio-demographic Index
ST-GPR = spatiotemporal Gaussian process regression

Overview

Modelled indicators included the proportion of women currently partnered (marital status), any contraceptive use, method-specific prevalence, and the proportion of non-users of contraception that have an unmet need for family planning. All contraception estimates were modelled separately for partnered and unpartnered women. Additional indicators of interest were calculated from the modelled estimates using the following equations:

$$\begin{aligned} Contra_{mod} = {} & Contra_{female\ sterilisation} + Contra_{male\ sterilisation} + Contra_{iud} + Contra_{injections} \\ & + Contra_{implants} + Contra_{condom} + Contra_{pill} + Contra_{diaphragm} \\ & + Contra_{emergency} + Contra_{other\ modern} \end{aligned}$$

$$Demand\ Satisfied = \frac{Contra_{mod}}{Contra_{any} + Prop_{unmet} * (1 - Contra_{any})}$$

$$Unmet\ Need = Prop_{unmet} * (1 - Contra_{any})$$

where $Contra_{mod}$ = modern contraceptive prevalence, $Contra_{method}$ = method-specific contraceptive prevalence, $Demand\ Satisfied$ = the proportion of women in need of family planning that are using a modern method, $Contra_{any}$ = any contraceptive prevalence, $Prop_{unmet}$ = the proportion of non-users who have an unmet need for family planning, and $Unmet\ Need$ = the proportion of women who have a need for family planning and are not using any method of contraception.

All women estimates of indicators were calculated by taking the average of the partnered and unpartnered estimates weighted by marital status as follows:

$$Estimate_{all} = Estimate_{partnered} * Marital\ Status + Estimate_{unpartnered} * (1 - Marital\ Status)$$

where $Marital\ Status =$ the proportion of women currently partnered.

Marital-splitting

In some tabulated reports, there was no information on the breakdown of family planning indicators by marital status. We thus conducted "marital-splitting" to disaggregate all women estimates from the tabulated data into partnered and unpartnered estimates. First, we ran preliminary models for each indicator using only the partnered and unpartnered data (so excluding the all women tabulated data). Second, based on the preliminary models, we computed 1000 draws of an all women estimate of each family planning indicator by multiplying the share partnered and unpartnered by the corresponding indicator estimate. Third, we calculated 100 draws of the ratio of each of the partnered/unpartnered estimates to the all women indicator estimate. Ratios were calculated for every location-year-age group or age-aggregate group. Fourth, we generated 1000 normally distributed draws for each tabulated all women data point using the standard error reported in the tabulated data. Each generated draw was then multiplied by the ratio of partnered/unpartnered women to all women from the preliminary models to produce 1000 draws of partnered and unpartnered estimates. The mean and standard error were calculated for each group and then used in our final models. All calculations were performed in logit-space and sample sizes were split according to the marital-age-sex-population distribution from the results of our marital status model and GBD population estimates.

Crosswalking

The algorithm for determining need for family planning has changed over time and requires a multitude of survey questions to be asked for its calculation. As a result, 235 surveys were missing one or more questions needed to accurately assign need to women which we grouped into following components:

(1) Missing all information on spacing and limiting for future children
(2) Missing information on limiting for future children, but has partial information on spacing (only know a women's desire for a child right now)
(3) Missing information on spacing for future children, but has information on limiting
(4) Missing information needed to determine fecundity
(5) Missing information from pregnant women on the desire for their current pregnancy
(6) Missing information needed to identify post-partum amenorrhoeic women from a birth within the last 2 years, and/or the desire for their most recent pregnancy

Surveys could only be flagged for one of (1), (2), or (3) because these components refer to the same set of survey questions. If a survey was flagged for (4) then it was also flagged for (6) since last menstruation is a required question to determine fecundity and post-partum amenorrhoeic status. The number of surveys which were flagged for each combination of missing components by survey series are displayed below.

Supplementary Table 3. Number of surveys by missing component(s) of need algorithm

Component(s)	DHS	MICS	Generations and Gender Survey	RHS	WFS	PAPFAM/ PAPCHILD	Other
(1), (4), (5)				1			
(1), (4), (6)	2	1	2				3
(1), (5), (6)	1	1			26		1
(1), (6)							2
(2)				6			
(2), (4)				1			2
(2), (4), (5), (6)			1				1
(2), (4), (6)			5			2	1
(2), (5)				1			
(2), (5), (6)				1			10
(2), (6)			13	9			13
(3)	1			2			
(3), (4), (5), (6)							1
(3), (5), (6)						10	
(4)				2			6
(4), (5), (6)	1	2					5
(4), (6)	1	24					2
(5)	25			1			
(5), (6)	5			1			12
(6)	3	1		3	1	9	11

In order to ensure all input data for the proportion of women with unmet need was comparable, we adjusted, or “crosswalked,” for item non-response by performing a series of counterfactual extractions on surveys with all the information required (“gold standard”) and estimating the average difference by age group between the reference and counterfactual estimates. All surveys missing information (“non-gold standard”) were extracted under the counterfactual scenarios.

Supplementary Table 4. Reference and counterfactual scenarios for determining need

Missing Component	Reference Scenario	Counterfactual Scenario
(1) Missing desire for spacing and limiting	All partnered and sexually active unpartnered women who do not want a child within the next 2 years or do not want any (more) children have a need	Assume all partnered and sexually active unpartnered women have a need
(2) Missing desire for limiting, partial information on spacing	All partnered and sexually active unpartnered women who do not want a child within the next 2 years or do not want any (more) children have a need	Assume all partnered and sexually active unpartnered women who do not want a child right now have a need

(3) Missing desire for spacing	All partnered and sexually active unpartnered women who do not want a child within the next 2 years or do not want any (more) children have a need	All partnered and sexually active unpartnered women who do not want any more children in the future have a need, otherwise assume all other women do not have a need
(4) Missing fecundity	All women who meet the criteria for infecundity do not have a need	Assume all women are fecund, no change to need
(5) Missing desire for current pregnancy	All pregnant women do not have a need unless they expressed desire to delay/prevent their current pregnant	Assume all pregnant women do not have a need (no consideration of desire for current pregnancy)
(6) Missing identification of post-partum amenorrhoeic women and/or desire for most recent pregnancy	All post-partum amenorrhoeic women from a birth in the last two years do not have a need unless they expressed desire to delay/prevent their last pregnancy	Skip all processing of post-partum amenorrhoeic women, no change to need

Adjustment factors for non-gold standard data were produced using the CrossWalk package written by the IHME Mathematical Sciences team (https://github.com/ihmeuw-msca/CrossWalk). We applied the crosswalk model to the difference between matched reference and counterfactual estimates from gold standard surveys transformed into logit space using the delta method. For every age group and combination of counterfactual scenarios we obtained the beta coefficient, beta standard error and gamma (between-study variance) from the model which were used to adjust the non-gold standard data with the following equations:

$$logit(proportion, adj) = logit(proportion, unadj) - \hat{\beta}$$

$$standard\ error(logit(proportion)) = \sqrt{logit(standard\ error)^2 + se(\hat{\beta})^2 + \widehat{\gamma^2}}$$

where β is the beta coefficient and γ is gamma.

Supplementary Table 5. Crosswalk adjustment factors for proportion unmet need among partnered non-users

Notes: Adjustment factor is the ratio of adjusted and unadjusted values in linear space. The median is provided as some ratios are quite large due to small unadjusted estimates. Missing values for median adjustment ratio reflect age group-counterfactual combinations that had no data to be adjusted.

Counterfactual(s)	Age Group	Gamma	Beta (95% UI)	Median adjustment factor
(1) Missing desire for spacing and limiting &<br>(4) Missing fecundity &<br>(5) Missing desire for current pregnancy	15-19	0.59	0.84 (0.76, 0.91)	0.7
	20-24	0.29	0.7 (0.64, 0.75)	0.78

	25-29	0.25	0.84 (0.79, 0.89)	0.73
	30-34	0.3	1.09 (1.04, 1.14)	0.67
	35-39	0.52	1.62 (1.55, 1.69)	0.61
	40-44	0.93	2.82 (2.72, 2.91)	0.7
	45-49	1.68	4.91 (4.77, 5.05)	0.31
(1) Missing desire for spacing and limiting & (4) Missing fecundity & (6) Missing identification of post-partum amenorrhoeic women and/or desire for most recent pregnancy	15-19	0.28	1.93 (1.88, 1.99)	0.74
	20-24	0.15	1.89 (1.85, 1.93)	0.64
	25-29	0.12	2.03 (1.99, 2.06)	0.5
	30-34	0.1	2.29 (2.25, 2.32)	0.47
	35-39	0.18	2.81 (2.76, 2.85)	0.45
	40-44	0.43	4.01 (3.94, 4.09)	0.49
	45-49	1.12	5.93 (5.79, 6.08)	0.21
(1) Missing desire for spacing and limiting & (5) Missing desire for current pregnant & (6) Missing identification of post-partum amenorrhoeic women and/or desire for most recent pregnancy	15-19	0.77	1.25 (1.16, 1.34)	0.58
	20-24	0.42	1.16 (1.1, 1.22)	0.61
	25-29	0.28	1.13 (1.08, 1.18)	0.63
	30-34	0.24	1.06 (1.01, 1.11)	0.66
	35-39	0.22	0.89 (0.84, 0.94)	0.74
	40-44	0.13	0.59 (0.55, 0.63)	0.78
	45-49	0.07	0.29 (0.26, 0.32)	0.83
(1) Missing desire for spacing and limiting & (6) Missing identification of post-partum amenorrhoeic women and/or desire for most recent pregnancy	15-19	0.31	1.72 (1.66, 1.78)	0.79
	20-24	0.21	1.58 (1.54, 1.63)	0.72
	25-29	0.17	1.49 (1.45, 1.53)	0.36
	30-34	0.17	1.39 (1.35, 1.43)	0.4
	35-39	0.19	1.14 (1.09, 1.18)	0.47
	40-44	0.13	0.7 (0.66, 0.74)	0.61
	45-49	0.07	0.32 (0.29, 0.35)	0.78
(2) Missing desire for limiting, partial information on spacing	15-19	0.03	0.33 (0.3, 0.36)	0.79
	20-24	0.05	0.26 (0.23, 0.28)	0.85
	25-29	0.06	0.22 (0.2, 0.25)	0.87
	30-34	0.05	0.2 (0.18, 0.23)	0.89
	35-39	0.05	0.17 (0.14, 0.19)	0.91
	40-44	0.04	0.11 (0.08, 0.13)	0.95
	45-49	0.07	0.04 (0.01, 0.08)	0.97

(2) Missing desire for limiting, partial information on spacing &<br>(4) Missing fecundity	15-19	0.07	0.45 (0.41, 0.48)	0.82
	20-24	0.09	0.41 (0.38, 0.44)	0.84
	25-29	0.11	0.45 (0.42, 0.48)	0.78
	30-34	0.15	0.58 (0.54, 0.62)	0.76
	35-39	0.32	0.92 (0.87, 0.98)	0.76
	40-44	0.68	1.78 (1.7, 1.86)	0.69
	45-49	1.24	3.53 (3.42, 3.64)	0.49
(2) Missing desire for limiting, partial information on spacing &<br>(4) Missing fecundity &<br>(5) Missing desire for current pregnant &<br>(6) Missing identification of post-partum amenorrhoeic women and/or desire for most recent pregnancy	15-19	0.33	0.62 (0.56, 0.68)	--
	20-24	0.23	0.71 (0.66, 0.76)	0.67
	25-29	0.18	0.76 (0.72, 0.81)	0.62
	30-34	0.18	0.85 (0.8, 0.89)	0.67
	35-39	0.26	1.11 (1.06, 1.16)	0.73
	40-44	0.54	1.91 (1.84, 1.98)	0.71
	45-49	1	3.63 (3.53, 3.73)	0.65
(2) Missing desire for limiting, partial information on spacing &<br>(4) Missing fecundity &<br>(6) Missing identification of post-partum amenorrhoeic women and/or desire for most recent pregnancy	15-19	0.08	1.03 (0.99, 1.07)	0.77
	20-24	0.09	1.08 (1.05, 1.12)	0.52
	25-29	0.09	1.09 (1.06, 1.13)	0.49
	30-34	0.1	1.16 (1.12, 1.19)	0.55
	35-39	0.2	1.37 (1.32, 1.41)	0.65
	40-44	0.55	2.08 (2.01, 2.16)	0.51
	45-49	1.01	3.7 (3.6, 3.8)	0.73
(2) Missing desire for limiting, partial information on spacing &<br>(5) Missing desire for current pregnant	15-19	0.2	-0.06 (-0.11, -0.01)	1.05
	20-24	0.13	-0.09 (-0.13, -0.05)	1.06
	25-29	0.12	-0.08 (-0.12, -0.05)	1.05
	30-34	0.1	-0.07 (-0.1, -0.04)	1.04
	35-39	0.08	-0.04 (-0.08, -0.01)	1.02
	40-44	0.05	0.01 (-0.02, 0.03)	1
	45-49	0.07	0.02 (-0.01, 0.05)	--
(2) Missing desire for limiting, partial information on spacing &	15-19	0.33	0.49 (0.43, 0.55)	0.76

(5) Missing desire for current pregnant &<br>(6) Missing identification of post-partum amenorrhoeic women and/or desire for most recent pregnancy				
	20-24	0.26	0.54 (0.49, 0.59)	0.76
	25-29	0.21	0.52 (0.47, 0.56)	0.72
	30-34	0.16	0.44 (0.4, 0.48)	0.75
	35-39	0.12	0.32 (0.28, 0.36)	0.84
	40-44	0.05	0.19 (0.16, 0.21)	0.89
	45-49	0.05	0.08 (0.06, 0.11)	0.95
(2) Missing desire for limiting, partial information on spacing &<br>(6) Missing identification of post-partum amenorrhoeic women and/or desire for most recent pregnancy	15-19	0.09	0.9 (0.86, 0.93)	0.51
	20-24	0.12	0.91 (0.87, 0.94)	0.51
	25-29	0.12	0.83 (0.79, 0.86)	0.54
	30-34	0.11	0.73 (0.69, 0.76)	0.59
	35-39	0.09	0.53 (0.5, 0.57)	0.71
	40-44	0.04	0.29 (0.26, 0.31)	0.83
	45-49	0.05	0.11 (0.09, 0.14)	0.92
(3) Missing desire for spacing	15-19	0.14	-1.07 (-1.12, -1.03)	2.15
	20-24	0.13	-1.04 (-1.07, -1)	1.78
	25-29	0.11	-0.84 (-0.87, -0.8)	1.54
	30-34	0.08	-0.6 (-0.63, -0.57)	1.33
	35-39	0.06	-0.38 (-0.41, -0.35)	1.18
	40-44	0.05	-0.22 (-0.24, -0.19)	1.09
	45-49	0.02	-0.13 (-0.15, -0.1)	1.08
(3) Missing desire for spacing &<br>(4) Missing fecundity &<br>(5) Missing desire for current pregnant &<br>(6) Missing identification of post-partum amenorrhoeic women and/or desire for most recent pregnancy	15-19	0.97	-1.99 (-2.1, -1.88)	--
	20-24	0.81	-1.61 (-1.71, -1.52)	2.75
	25-29	0.52	-1.03 (-1.11, -0.95)	1.9
	30-34	0.31	-0.48 (-0.54, -0.42)	1.35
	35-39	0.24	0.06 (0, 0.11)	0.98
	40-44	0.29	0.81 (0.75, 0.87)	0.74
	45-49	0.44	1.77 (1.7, 1.84)	0.76
(3) Missing desire for spacing &<br>(5) Missing desire for current pregnancy	15-19	0.16	-1.8 (-1.86, -1.75)	3.2
	20-24	0.11	-1.6 (-1.64, -1.56)	3.08
	25-29	0.13	-1.28 (-1.32, -1.24)	2.27
	30-34	0.11	-0.95 (-0.99, -0.92)	1.8

	35-39	0.1	-0.62 (-0.66, -0.59)	1.36
	40-44	0.09	-0.33 (-0.37, -0.3)	1.23
	45-49	0.03	-0.16 (-0.18, -0.13)	1.13
(3) Missing desire for spacing &<br>(5) Missing desire for current pregnant &<br>(6) Missing identification of post-partum amenorrhoeic women and/or desire for most recent pregnancy	15-19	0.98	-2.05 (-2.16, -1.94)	5.65
	20-24	0.83	-1.69 (-1.79, -1.59)	4.01
	25-29	0.51	-1.14 (-1.22, -1.07)	2.41
	30-34	0.25	-0.69 (-0.74, -0.63)	1.61
	35-39	0.09	-0.36 (-0.39, -0.32)	1.23
	40-44	0.02	-0.14 (-0.17, -0.12)	1.07
	45-49	0.01	-0.06 (-0.08, -0.04)	1.04
(4) Missing fecundity	15-19	0.01	0.07 (0.05, 0.09)	0.82
	20-24	0.04	0.07 (0.04, 0.09)	0.83
	25-29	0.05	0.1 (0.08, 0.13)	0.83
	30-34	0.06	0.2 (0.17, 0.23)	0.83
	35-39	0.15	0.43 (0.39, 0.47)	0.82
	40-44	0.3	0.95 (0.89, 1)	0.82
	45-49	0.49	1.8 (1.74, 1.87)	0.6
(4) Missing fecundity &<br>(5) Missing desire for current pregnant &<br>(6) Missing identification of post-partum amenorrhoeic women and/or desire for most recent pregnancy	15-19	0.24	0.21 (0.16, 0.27)	0.84
	20-24	0.18	0.33 (0.29, 0.37)	0.79
	25-29	0.14	0.38 (0.34, 0.41)	0.78
	30-34	0.13	0.43 (0.39, 0.46)	0.8
	35-39	0.14	0.58 (0.54, 0.62)	0.78
	40-44	0.23	1.04 (0.99, 1.09)	0.6
	45-49	0.39	1.86 (1.8, 1.92)	0.34
(4) Missing fecundity &<br>(6) Missing identification of post-partum amenorrhoeic women and/or desire for most recent pregnancy	15-19	0.06	0.62 (0.58, 0.65)	0.71
	20-24	0.08	0.68 (0.65, 0.71)	0.69
	25-29	0.08	0.68 (0.65, 0.71)	0.67
	30-34	0.07	0.71 (0.68, 0.74)	0.68
	35-39	0.1	0.8 (0.77, 0.83)	0.66
	40-44	0.22	1.15 (1.1, 1.19)	0.63
	45-49	0.39	1.88 (1.82, 1.94)	0.44
(5) Missing desire for current pregnancy	15-19	0	-0.45 (-0.48, -0.41)	1.33
	20-24	0	-0.41 (-0.44, -0.38)	1.28
	25-29	0	-0.37 (-0.4, -0.34)	1.24

	30-34	0	-0.32 (-0.35, -0.3)	1.2
	35-39	0	-0.25 (-0.27, -0.22)	1.14
	40-44	0	-0.12 (-0.13, -0.1)	1.07
	45-49	0	-0.06 (-0.08, -0.04)	1.04
(5) Missing desire for current pregnant & (6) Missing identification of post-partum amenorrhoeic women and/or desire for most recent pregnancy	15-19	0	0.12 (0.06, 0.17)	0.92
	20-24	0	0.21 (0.16, 0.26)	0.87
	25-29	0	0.22 (0.17, 0.26)	0.88
	30-34	0	0.18 (0.14, 0.22)	0.9
	35-39	0	0.11 (0.08, 0.14)	0.94
	40-44	0	0.06 (0.04, 0.08)	0.97
	45-49	0	0.02 (0, 0.04)	0.99
(6) Missing identification of post-partum amenorrhoeic women and/or desire for most recent pregnancy	15-19	0	0.52 (0.49, 0.56)	0.75
	20-24	0	0.56 (0.53, 0.6)	0.74
	25-29	0	0.52 (0.48, 0.55)	0.74
	30-34	0	0.45 (0.42, 0.48)	0.77
	35-39	0	0.32 (0.29, 0.35)	0.85
	40-44	0	0.16 (0.14, 0.18)	0.93
	45-49	0	0.05 (0.03, 0.07)	0.97

Supplementary Table 6. Crosswalk adjustment factors for proportion unmet need among unpartnered non-users

Notes: Adjustment factor is the ratio of adjusted and unadjusted values in linear space. The median is provided as some ratios are quite large due to small unadjusted estimates. Missing values for median adjustment ratio reflect age group-counterfactual combinations that had no data to be adjusted.

Counterfactual(s)	Age Group	Gamma	Beta (95% UI)	Median adjustment factor
(1) Missing desire for spacing and limiting & (4) Missing fecundity & (5) Missing desire for current pregnancy	15-19	0.59	0.84 (0.76, 0.91)	1.12
	20-24	0.29	0.7 (0.64, 0.75)	--
	25-29	0.25	0.84 (0.79, 0.89)	--
	30-34	0.3	1.09 (1.04, 1.14)	--
	35-39	0.52	1.62 (1.55, 1.69)	--
	40-44	0.93	2.82 (2.72, 2.91)	--
	45-49	1.68	4.91 (4.77, 5.05)	--
(1) Missing desire for spacing and limiting & (4) Missing fecundity &	15-19	0.28	1.93 (1.88, 1.99)	0.99

(6) Missing identification of post-partum amenorrhoeic women and/or desire for most recent pregnancy				
	20-24	0.15	1.89 (1.85, 1.93)	--
	25-29	0.12	2.03 (1.99, 2.06)	--
	30-34	0.1	2.29 (2.25, 2.32)	--
	35-39	0.18	2.81 (2.76, 2.85)	--
	40-44	0.43	4.01 (3.94, 4.09)	--
	45-49	1.12	5.93 (5.79, 6.08)	0.82
(1) Missing desire for spacing and limiting & (5) Missing desire for current pregnant & (6) Missing identification of post-partum amenorrhoeic women and/or desire for most recent pregnancy	15-19	0.77	1.25 (1.16, 1.34)	--
	20-24	0.42	1.16 (1.1, 1.22)	--
	25-29	0.28	1.13 (1.08, 1.18)	--
	30-34	0.24	1.06 (1.01, 1.11)	--
	35-39	0.22	0.89 (0.84, 0.94)	0.72
	40-44	0.13	0.59 (0.55, 0.63)	--
	45-49	0.07	0.29 (0.26, 0.32)	--
(1) Missing desire for spacing and limiting & (6) Missing identification of post-partum amenorrhoeic women and/or desire for most recent pregnancy	15-19	0.31	1.72 (1.66, 1.78)	--
	20-24	0.21	1.58 (1.54, 1.63)	0.66
	25-29	0.17	1.49 (1.45, 1.53)	--
	30-34	0.17	1.39 (1.35, 1.43)	--
	35-39	0.19	1.14 (1.09, 1.18)	0.57
	40-44	0.13	0.7 (0.66, 0.74)	--
	45-49	0.07	0.32 (0.29, 0.35)	0.43
(2) Missing desire for limiting, partial information on spacing	15-19	0.03	0.33 (0.3, 0.36)	--
	20-24	0.05	0.26 (0.23, 0.28)	--
	25-29	0.06	0.22 (0.2, 0.25)	--
	30-34	0.05	0.2 (0.18, 0.23)	--
	35-39	0.05	0.17 (0.14, 0.19)	--
	40-44	0.04	0.11 (0.08, 0.13)	--
	45-49	0.07	0.04 (0.01, 0.08)	--
(2) Missing desire for limiting, partial information on spacing & (4) Missing fecundity	15-19	0.07	0.45 (0.41, 0.48)	--
	20-24	0.09	0.41 (0.38, 0.44)	--
	25-29	0.11	0.45 (0.42, 0.48)	--
	30-34	0.15	0.58 (0.54, 0.62)	--
	35-39	0.32	0.92 (0.87, 0.98)	--

	40-44	0.68	1.78 (1.7, 1.86)	--
	45-49	1.24	3.53 (3.42, 3.64)	--
(2) Missing desire for limiting, partial information on spacing &<br>(4) Missing fecundity &<br>(5) Missing desire for current pregnant &<br>(6) Missing identification of post-partum amenorrhoeic women and/or desire for most recent pregnancy	15-19	0.33	0.62 (0.56, 0.68)	--
	20-24	0.23	0.71 (0.66, 0.76)	--
	25-29	0.18	0.76 (0.72, 0.81)	--
	30-34	0.18	0.85 (0.8, 0.89)	--
	35-39	0.26	1.11 (1.06, 1.16)	--
	40-44	0.54	1.91 (1.84, 1.98)	--
	45-49	1	3.63 (3.53, 3.73)	--
(2) Missing desire for limiting, partial information on spacing &<br>(4) Missing fecundity &<br>(6) Missing identification of post-partum amenorrhoeic women and/or desire for most recent pregnancy	15-19	0.08	1.03 (0.99, 1.07)	--
	20-24	0.09	1.08 (1.05, 1.12)	--
	25-29	0.09	1.09 (1.06, 1.13)	--
	30-34	0.1	1.16 (1.12, 1.19)	--
	35-39	0.2	1.37 (1.32, 1.41)	--
	40-44	0.55	2.08 (2.01, 2.16)	--
	45-49	1.01	3.7 (3.6, 3.8)	--
(2) Missing desire for limiting, partial information on spacing &<br>(5) Missing desire for current pregnancy	15-19	0.2	-0.06 (-0.11, -0.01)	1.08
	20-24	0.13	-0.09 (-0.13, -0.05)	--
	25-29	0.12	-0.08 (-0.12, -0.05)	--
	30-34	0.1	-0.07 (-0.1, -0.04)	--
	35-39	0.08	-0.04 (-0.08, -0.01)	--
	40-44	0.05	0.01 (-0.02, 0.03)	--
	45-49	0.07	0.02 (-0.01, 0.05)	--
(2) Missing desire for limiting, partial information on spacing &<br>(5) Missing desire for current pregnant &<br>(6) Missing identification of post-partum amenorrhoeic women and/or desire for most recent pregnancy	15-19	0.33	0.49 (0.43, 0.55)	1.03
	20-24	0.26	0.54 (0.49, 0.59)	--
	25-29	0.21	0.52 (0.47, 0.56)	--
	30-34	0.16	0.44 (0.4, 0.48)	--

	35-39	0.12	0.32 (0.28, 0.36)	--
	40-44	0.05	0.19 (0.16, 0.21)	--
	45-49	0.05	0.08 (0.06, 0.11)	0.85
(2) Missing desire for limiting, partial information on spacing & (6) Missing identification of post-partum amenorrhoeic women and/or desire for most recent pregnancy	15-19	0.09	0.9 (0.86, 0.93)	--
	20-24	0.12	0.91 (0.87, 0.94)	--
	25-29	0.12	0.83 (0.79, 0.86)	--
	30-34	0.11	0.73 (0.69, 0.76)	--
	35-39	0.09	0.53 (0.5, 0.57)	0.78
	40-44	0.04	0.29 (0.26, 0.31)	--
	45-49	0.05	0.11 (0.09, 0.14)	--
(3) Missing desire for spacing	15-19	0.14	-1.07 (-1.12, -1.03)	--
	20-24	0.13	-1.04 (-1.07, -1)	0.73
	25-29	0.11	-0.84 (-0.87, -0.8)	--
	30-34	0.08	-0.6 (-0.63, -0.57)	--
	35-39	0.06	-0.38 (-0.41, -0.35)	0.62
	40-44	0.05	-0.22 (-0.24, -0.19)	--
	45-49	0.02	-0.13 (-0.15, -0.1)	0.44
(3) Missing desire for spacing & (4) Missing fecundity & (5) Missing desire for current pregnant & (6) Missing identification of post-partum amenorrhoeic women and/or desire for most recent pregnancy	15-19	0.97	-1.99 (-2.1, -1.88)	1.5
	20-24	0.81	-1.61 (-1.71, -1.52)	--
	25-29	0.52	-1.03 (-1.11, -0.95)	--
	30-34	0.31	-0.48 (-0.54, -0.42)	--
	35-39	0.24	0.06 (0, 0.11)	--
	40-44	0.29	0.81 (0.75, 0.87)	--
	45-49	0.44	1.77 (1.7, 1.84)	--
(3) Missing desire for spacing & (5) Missing desire for current pregnancy	15-19	0.16	-1.8 (-1.86, -1.75)	1.32
	20-24	0.11	-1.6 (-1.64, -1.56)	--
	25-29	0.13	-1.28 (-1.32, -1.24)	--
	30-34	0.11	-0.95 (-0.99, -0.92)	--
	35-39	0.1	-0.62 (-0.66, -0.59)	--
	40-44	0.09	-0.33 (-0.37, -0.3)	--
	45-49	0.03	-0.16 (-0.18, -0.13)	1.09
(3) Missing desire for spacing & (5) Missing desire for current pregnant &	15-19	0.98	-2.05 (-2.16, -1.94)	--

(6) Missing identification of post-partum amenorrhoeic women and/or desire for most recent pregnancy				
	20-24	0.83	-1.69 (-1.79, -1.59)	--
	25-29	0.51	-1.14 (-1.22, -1.07)	--
	30-34	0.25	-0.69 (-0.74, -0.63)	--
	35-39	0.09	-0.36 (-0.39, -0.32)	1.01
	40-44	0.02	-0.14 (-0.17, -0.12)	--
	45-49	0.01	-0.06 (-0.08, -0.04)	--
(4) Missing fecundity	15-19	0.01	0.07 (0.05, 0.09)	--
	20-24	0.04	0.07 (0.04, 0.09)	0.96
	25-29	0.05	0.1 (0.08, 0.13)	--
	30-34	0.06	0.2 (0.17, 0.23)	--
	35-39	0.15	0.43 (0.39, 0.47)	0.87
	40-44	0.3	0.95 (0.89, 1)	--
	45-49	0.49	1.8 (1.74, 1.87)	0.82
(4) Missing fecundity &<br>(5) Missing desire for current pregnant &<br>(6) Missing identification of post-partum amenorrhoeic women and/or desire for most recent pregnancy	15-19	0.24	0.21 (0.16, 0.27)	0.94
	20-24	0.18	0.33 (0.29, 0.37)	--
	25-29	0.14	0.38 (0.34, 0.41)	--
	30-34	0.13	0.43 (0.39, 0.46)	--
	35-39	0.14	0.58 (0.54, 0.62)	--
	40-44	0.23	1.04 (0.99, 1.09)	--
	45-49	0.39	1.86 (1.8, 1.92)	--
(4) Missing fecundity &<br>(6) Missing identification of post-partum amenorrhoeic women and/or desire for most recent pregnancy	15-19	0.06	0.62 (0.58, 0.65)	0.91
	20-24	0.08	0.68 (0.65, 0.71)	--
	25-29	0.08	0.68 (0.65, 0.71)	--
	30-34	0.07	0.71 (0.68, 0.74)	--
	35-39	0.1	0.8 (0.77, 0.83)	--
	40-44	0.22	1.15 (1.1, 1.19)	--
	45-49	0.39	1.88 (1.82, 1.94)	0.85
(5) Missing desire for current pregnancy	15-19	0	-0.45 (-0.48, -0.41)	--
	20-24	0	-0.41 (-0.44, -0.38)	--
	25-29	0	-0.37 (-0.4, -0.34)	--
	30-34	0	-0.32 (-0.35, -0.3)	--
	35-39	0	-0.25 (-0.27, -0.22)	0.83
	40-44	0	-0.12 (-0.13, -0.1)	--
	45-49	0	-0.06 (-0.08, -0.04)	--

(5) Missing desire for current pregnant & (6) Missing identification of post-partum amenorrhoeic women and/or desire for most recent pregnancy	15-19	0	0.12 (0.06, 0.17)	--
	20-24	0	0.21 (0.16, 0.26)	0.85
	25-29	0	0.22 (0.17, 0.26)	--
	30-34	0	0.18 (0.14, 0.22)	--
	35-39	0	0.11 (0.08, 0.14)	0.86
	40-44	0	0.06 (0.04, 0.08)	--
	45-49	0	0.02 (0, 0.04)	0.89
(6) Missing identification of post-partum amenorrhoeic women and/or desire for most recent pregnancy	15-19	0	0.52 (0.49, 0.56)	0.89
	20-24	0	0.56 (0.53, 0.6)	--
	25-29	0	0.52 (0.48, 0.55)	--
	30-34	0	0.45 (0.42, 0.48)	--
	35-39	0	0.32 (0.29, 0.35)	--
	40-44	0	0.16 (0.14, 0.18)	--
	45-49	0	0.05 (0.03, 0.07)	--

Age-splitting

Following the same methods described above for marital-splitting, age-aggregated data from tabulated reports which spanned multiple 5-year age bins underwent "age-splitting" to disaggregate estimates into single 5-year age bins. We ran preliminary models ran with data that were already age bin-specific and computed 1000 draws of age-aggregated estimates using GBD population counts covering the same age bins as the age-aggregated tabulated data. Next, we calculated 1000 draws the ratios of the 5-year age bin estimates to the age-aggregated estimate for every location-year-age group. Ratios were applied to 1000 generated normally distributed draws of the age-aggregated tabulated data from which we took the mean and standard error of to produce the final age-specific estimates. All calculations were performed in logit-space and sample sizes were split according to the marital-age-sex-population distribution from the results of our marital status model and GBD population estimates.

Modelling

Prepared data were modelled in logit-space using spatiotemporal Gaussian process regression (ST-GPR), a flexible modelling tool that draws strength across locations, time, and age used widely within the GBD study to produce full time series estimates of an indicator with uncertainty intervals from multiple sources of data. The model is composed of three stages. In the first stage, a linear regression predicts indicator values for every location, year, and 5-year age group. In the second stage, residuals between the data and corresponding linear predictions are smoothed over space, time, and age. In the third stage, the smoothed residuals are used as the mean model in a Gaussian process regression, which leverages data point variances to produce 1000 draws of the indicator for every location, year, and 5-year age group, from which mean values and quantile-based uncertainty intervals were calculated.

ST-GPR was ran twice for each modelled indicator – a preliminary run with only marital- and age bin-specific data, then a final run with all data including all women and age-aggregated data that has been

split into partnered and unpartnered estimates and the desired 5-year age bins using the splitting methods described above. All stage one linear models included a fixed effect on age group and nested random effects on GBD super-region, region and location. Due to the data sparsity of the unpartnered models relative to the partnered models, all modelled partnered estimates were incorporated in the unpartnered models as logit-transformed covariates to assist in the stage one model fit. Additional suites of GBD covariates included were (1) education years per capita and lag-distributed income (LDI) per capita, or (2) the Socio-demographic Index (SDI). Covariate selection was determined based on model fit. Lastly, any contraceptive use was added as a covariate to the stage one linear model for proportion unmet need among non-users as these models are particularly data sparse and any contraceptive was used to stabilize the stage one fit.

To evaluate model fit, we sought to minimize the root mean square error (RMSE) of the first stage model. We also extensively vetted modelled estimates by visually inspecting the modelled estimates alongside the data extracted from reports and microdata. Each modelled estimate was reviewed several times by at least two modellers and was flagged and investigated for validity when they deviated substantially from other extracted data. We also considered the plausibility of time trends in selection of covariates and refining model specifications, excluding models that did not follow the extracted data closely, imposed implausible trends (e.g. a strong U shape) in between extracted data points, or otherwise did not align with priors of modellers and experts in family planning.

Additional details on the methodology for ST-GPR can be found in Appendix 1, Section 2.3.3 of the GBD 2019 Risk Factors Capstone[6]. Additional details on the SDI covariate can be found in Appendix 1, Section 6 of the GBD 2019 Demographics Capstone[7]. Details on the LDI per capita covariate are found in Appendix 1, Section 7.3 of the GBD 2019 Demographics Capstone[7].

First-stage generalized linear mixed models and coefficients

$$logit(Proportion\ Partnered) \sim \hat{\beta}SDI + \hat{\delta}as.factor(age\ group) + \gamma_l + \gamma_r + \gamma_{sr}$$

Where γ represents random effects for location, GBD region and GBD super-region, respectively.

Supplementary Table 7. ST-GPR model coefficients for marital status (proportion of women currently married/in-union, or otherwise partnered)

Preliminary RMSE = 0.1074; Final RMSE = 0.1074

Variable	Preliminary Model Coefficients (95% UI)	Final Model Coefficients (95% UI)
Intercept	-0.586 (-0.801, -0.37)	-0.564 (-0.79, -0.339)
SDI	-2.583 (-2.739, -2.428)	-2.65 (-2.807, -2.493)
Age group 20-24	1.971 (1.936, 2.007)	1.981 (1.945, 2.017)
Age group 25-29	3.008 (2.972, 3.043)	3.026 (2.99, 3.061)
Age group 30-34	3.455 (3.42, 3.491)	3.478 (3.442, 3.514)
Age group 35-39	3.536 (3.501, 3.572)	3.561 (3.526, 3.597)
Age group 40-44	3.417 (3.382, 3.453)	3.443 (3.408, 3.479)
Age group 45-49	3.217 (3.181, 3.253)	3.242 (3.205, 3.278)

$$logit\left(Contra_{any_{partnered}}\right) \sim \hat{\beta}education_{yrs_{pc}} + \hat{\beta}\log\left(LDI_{pc}\right) + \hat{\delta}as.factor(age\ group) + \gamma_l + \gamma_r + \gamma_{sr}$$

Where γ represents random effects for location, GBD region and GBD super-region, respectively.

Supplementary Table 8. ST-GPR model coefficients for any contraceptive use among partnered women

Preliminary RMSE = 0.2097; Final RMSE = 0.2075

Variable	Preliminary Model Coefficients (95% UI)	Final Model Coefficients (95% UI)
Intercept	-4.803 (-5.328, -4.279)	-4.688 (-5.181, -4.195)
Education years per capita	0.193 (0.182, 0.204)	0.187 (0.177, 0.198)
LDI per capita, log	0.258 (0.206, 0.309)	0.254 (0.205, 0.302)
Age group 20-24	0.598 (0.555, 0.641)	0.594 (0.553, 0.635)
Age group 25-29	0.91 (0.867, 0.953)	0.921 (0.88, 0.962)
Age group 30-34	1.233 (1.19, 1.275)	1.24 (1.199, 1.28)
Age group 35-39	1.405 (1.362, 1.449)	1.415 (1.374, 1.457)
Age group 40-44	1.317 (1.272, 1.362)	1.326 (1.284, 1.369)
Age group 45-49	0.791 (0.744, 0.839)	0.81 (0.765, 0.855)

$$logit\left(Contra_{any_{unpartnered}}\right) \sim \hat{\beta} education_{yrs_{pc}} + \hat{\beta}\log(LDI_{pc}) + \hat{\beta} logit\left(Contra_{any_{partnered}}\right) + \hat{\delta} as.factor(age\ group) + \gamma_l + \gamma_r + \gamma_{sr}$$

Where γ represents random effects for location, GBD region and GBD super-region, respectively.

Supplementary Table 9. ST-GPR model coefficients for any contraceptive use among unpartnered women

Preliminary RMSE = 0.1583; Final RMSE = 0.1638

Variable	Preliminary Model Coefficients (95% UI)	Final Model Coefficients (95% UI)
Intercept	-4.958 (-6.472, -3.445)	-5.76 (-7.186, -4.333)
Education years per capita	-0.083 (-0.111, -0.055)	-0.095 (-0.121, -0.07)
LDI per capita, log	0.318 (0.202, 0.434)	0.422 (0.314, 0.53)
Any contraceptive use among partnered women, logit	1.032 (0.965, 1.099)	0.986 (0.924, 1.047)
Age group 20-24	0.844 (0.755, 0.932)	0.857 (0.774, 0.94)
Age group 25-29	1.036 (0.94, 1.132)	1.043 (0.953, 1.132)
Age group 30-34	0.85 (0.746, 0.955)	0.845 (0.748, 0.941)
Age group 35-39	0.668 (0.557, 0.778)	0.655 (0.553, 0.757)
Age group 40-44	0.485 (0.376, 0.594)	0.465 (0.365, 0.565)
Age group 45-49	0.532 (0.435, 0.629)	0.486 (0.396, 0.575)

$$logit\left(Contra_{method_{partnered}}\right) \sim \hat{\beta} SDI + \hat{\delta} as.factor(age\ group) + \gamma_l + \gamma_r + \gamma_{sr}$$

Where γ represents random effects for location, GBD region and GBD super-region, respectively.

Supplementary Table 10. ST-GPR model coefficients for by-method use among partnered women

Method	Variable	Preliminary Model Coefficients (95% UI)	Final Model Coefficients (95% UI)
Female Sterilisation<br>Preliminary RMSE = 0.1938<br>Final RMSE = 0.2016	Intercept	-7.126 (-7.973, -6.279)	-7.042 (-7.887, -6.198)
	SDI	1.333 (1.026, 1.64)	1.272 (0.993, 1.551)
	Age group 20-24	0.906 (0.837, 0.975)	0.887 (0.822, 0.952)
	Age group 25-29	1.817 (1.748, 1.886)	1.831 (1.766, 1.896)
	Age group 30-34	2.756 (2.687, 2.825)	2.768 (2.703, 2.833)
	Age group 35-39	3.412 (3.343, 3.481)	3.434 (3.369, 3.499)
	Age group 40-44	3.759 (3.69, 3.828)	3.782 (3.717, 3.847)
	Age group 45-49	3.785 (3.714, 3.856)	3.794 (3.726, 3.861)

Male Sterilisation<br>Preliminary RMSE = 0.0257<br>Final RMSE = 0.0264	Intercept	-6.464 (-7.211, -5.716)	-6.546 (-7.279, -5.812)
	SDI	-0.217 (-0.519, 0.086)	-0.051 (-0.336, 0.235)
	Age group 20-24	0.233 (0.165, 0.301)	0.207 (0.14, 0.274)
	Age group 25-29	0.513 (0.444, 0.581)	0.524 (0.457, 0.591)
	Age group 30-34	0.844 (0.776, 0.912)	0.869 (0.802, 0.936)
	Age group 35-39	1.114 (1.046, 1.182)	1.15 (1.083, 1.217)
	Age group 40-44	1.232 (1.164, 1.301)	1.271 (1.204, 1.338)
	Age group 45-49	1.171 (1.101, 1.241)	1.183 (1.114, 1.252)
IUD<br>Preliminary RMSE = 0.0733<br>Final RMSE = 0.0737	Intercept	-5.648 (-6.412, -4.885)	-5.663 (-6.421, -4.905)
	SDI	1.747 (1.464, 2.03)	1.803 (1.541, 2.065)
	Age group 20-24	0.778 (0.715, 0.842)	0.782 (0.721, 0.843)
	Age group 25-29	1.251 (1.188, 1.314)	1.256 (1.195, 1.317)
	Age group 30-34	1.511 (1.447, 1.574)	1.517 (1.456, 1.577)
	Age group 35-39	1.595 (1.531, 1.658)	1.606 (1.546, 1.667)
	Age group 40-44	1.423 (1.359, 1.487)	1.44 (1.379, 1.501)
	Age group 45-49	0.936 (0.871, 1.002)	0.942 (0.879, 1.005)
Injections<br>Preliminary RMSE = 0.1306<br>Final RMSE = 0.1284	Intercept	-10.986 (-12.84, -9.132)	-10.932 (-12.755, -9.109)
	SDI	11.773 (11.421, 12.125)	11.715 (11.37, 12.06)
	Age group 20-24	0.447 (0.371, 0.523)	0.438 (0.361, 0.515)
	Age group 25-29	0.62 (0.544, 0.696)	0.614 (0.537, 0.69)
	Age group 30-34	0.655 (0.579, 0.73)	0.646 (0.569, 0.722)
	Age group 35-39	0.551 (0.475, 0.627)	0.54 (0.463, 0.616)
	Age group 40-44	0.284 (0.208, 0.361)	0.271 (0.194, 0.348)
	Age group 45-49	-0.359 (-0.437, -0.281)	-0.358 (-0.437, -0.28)
Implants<br>Preliminary RMSE = 0.0668<br>Final RMSE = 0.0627	Intercept	-16.804 (-18.937, -14.67)	-16.387 (-18.431, -14.344)
	SDI	18.889 (18.222, 19.556)	18.135 (17.484, 18.787)
	Age group 20-24	0.469 (0.36, 0.578)	0.459 (0.35, 0.567)
	Age group 25-29	0.661 (0.552, 0.769)	0.651 (0.543, 0.759)
	Age group 30-34	0.706 (0.597, 0.814)	0.688 (0.58, 0.796)
	Age group 35-39	0.574 (0.465, 0.682)	0.562 (0.454, 0.67)
	Age group 40-44	0.369 (0.26, 0.478)	0.359 (0.251, 0.468)
	Age group 45-49	-0.099 (-0.209, 0.012)	-0.095 (-0.206, 0.015)

Pill<br>Preliminary RMSE = 0.0949<br>Final RMSE = 0.0937	Intercept	-4.752 (-5.21, -4.295)	-4.478 (-4.923, -4.032)
	SDI	2.968 (2.731, 3.205)	2.635 (2.411, 2.859)
	Age group 20-24	0.584 (0.531, 0.637)	0.572 (0.52, 0.624)
	Age group 25-29	0.76 (0.707, 0.813)	0.732 (0.68, 0.783)
	Age group 30-34	0.716 (0.663, 0.769)	0.687 (0.635, 0.738)
	Age group 35-39	0.52 (0.467, 0.573)	0.482 (0.43, 0.534)
	Age group 40-44	0.143 (0.09, 0.196)	0.105 (0.053, 0.157)
	Age group 45-49	-0.568 (-0.622, -0.513)	-0.594 (-0.648, -0.54)
Condom<br>Preliminary RMSE = 0.0510<br>Final RMSE = 0.0523	Intercept	-7.263 (-7.731, -6.794)	-7.279 (-7.735, -6.824)
	SDI	6.482 (6.213, 6.75)	6.471 (6.222, 6.721)
	Age group 20-24	0.402 (0.341, 0.463)	0.412 (0.354, 0.47)
	Age group 25-29	0.514 (0.453, 0.574)	0.537 (0.479, 0.595)
	Age group 30-34	0.498 (0.437, 0.558)	0.528 (0.47, 0.586)
	Age group 35-39	0.325 (0.264, 0.385)	0.354 (0.296, 0.412)
	Age group 40-44	0.102 (0.041, 0.163)	0.135 (0.077, 0.193)
	Age group 45-49	-0.444 (-0.506, -0.381)	-0.411 (-0.471, -0.351)
Diaphragm<br>Preliminary RMSE = 0.0090<br>Final RMSE = 0.0091	Intercept	-4.844 (-5.418, -4.27)	-4.705 (-5.33, -4.08)
	SDI	-3.283 (-3.667, -2.898)	-3.498 (-3.884, -3.111)
	Age group 20-24	0.025 (-0.05, 0.1)	0.021 (-0.056, 0.097)
	Age group 25-29	0.173 (0.097, 0.248)	0.18 (0.104, 0.257)
	Age group 30-34	0.27 (0.195, 0.345)	0.271 (0.195, 0.347)
	Age group 35-39	0.31 (0.235, 0.385)	0.315 (0.239, 0.391)
	Age group 40-44	0.324 (0.249, 0.4)	0.332 (0.256, 0.409)
	Age group 45-49	0.212 (0.135, 0.289)	0.219 (0.14, 0.297)
Emergency Contraception<br>Preliminary RMSE = 0.0059<br>Final RMSE = 0.0055	Intercept	-7.121 (-7.683, -6.558)	-6.872 (-7.327, -6.418)
	SDI	0.991 (0.056, 1.925)	0.544 (-0.23, 1.318)
	Age group 20-24	0.174 (0.015, 0.334)	0.174 (0.042, 0.306)
	Age group 25-29	0.143 (-0.016, 0.301)	0.143 (0.012, 0.275)
	Age group 30-34	0.109 (-0.05, 0.269)	0.122 (-0.009, 0.254)
	Age group 35-39	0.054 (-0.106, 0.214)	0.066 (-0.066, 0.198)
	Age group 40-44	-0.058 (-0.218, 0.102)	-0.037 (-0.168, 0.095)
	Age group 45-49	-0.133 (-0.295, 0.029)	-0.11 (-0.244, 0.023)

Other Modern<br>Preliminary RMSE = 0.0073<br>Final RMSE = 0.0073	Intercept	-5.056 (-5.663, -4.448)	-4.988 (-5.632, -4.344)
	SDI	-2.686 (-3.063, -2.309)	-2.733 (-3.101, -2.366)
	Age group 20-24	0.237 (0.153, 0.322)	0.233 (0.149, 0.317)
	Age group 25-29	0.279 (0.195, 0.364)	0.28 (0.196, 0.363)
	Age group 30-34	0.321 (0.237, 0.406)	0.315 (0.231, 0.398)
	Age group 35-39	0.304 (0.219, 0.388)	0.299 (0.216, 0.383)
	Age group 40-44	0.319 (0.234, 0.404)	0.315 (0.231, 0.399)
	Age group 45-49	0.125 (0.038, 0.212)	0.123 (0.037, 0.209)
Lactational Amenorrhoea Method<br>Preliminary RMSE = 0.0259<br>Final RMSE = 0.0261	Intercept	-2.741 (-3.505, -1.977)	-2.381 (-3.141, -1.621)
	SDI	-5.189 (-5.81, -4.567)	-5.793 (-6.381, -5.205)
	Age group 20-24	0.262 (0.152, 0.372)	0.262 (0.157, 0.367)
	Age group 25-29	0.257 (0.147, 0.367)	0.259 (0.154, 0.364)
	Age group 30-34	0.06 (-0.05, 0.17)	0.062 (-0.043, 0.167)
	Age group 35-39	-0.169 (-0.279, -0.059)	-0.166 (-0.271, -0.061)
	Age group 40-44	-0.704 (-0.814, -0.594)	-0.703 (-0.808, -0.598)
	Age group 45-49	-1.225 (-1.337, -1.113)	-1.224 (-1.33, -1.117)
Rhythm<br>Preliminary RMSE = 0.0543<br>Final RMSE = 0.0541	Intercept	-3.843 (-4.315, -3.372)	-3.871 (-4.342, -3.4)
	SDI	-2.218 (-2.52, -1.916)	-2.139 (-2.431, -1.847)
	Age group 20-24	0.638 (0.571, 0.706)	0.639 (0.572, 0.705)
	Age group 25-29	0.914 (0.847, 0.982)	0.923 (0.857, 0.99)
	Age group 30-34	1.106 (1.039, 1.173)	1.116 (1.049, 1.182)
	Age group 35-39	1.223 (1.155, 1.29)	1.228 (1.162, 1.294)
	Age group 40-44	1.203 (1.135, 1.271)	1.209 (1.142, 1.275)
	Age group 45-49	0.923 (0.854, 0.993)	0.92 (0.851, 0.988)
Withdrawal<br>Preliminary RMSE = 0.0594<br>Final RMSE = 0.0604	Intercept	-5.274 (-5.907, -4.641)	-5.182 (-5.809, -4.556)
	SDI	1.077 (0.773, 1.382)	0.987 (0.7, 1.275)
	Age group 20-24	0.393 (0.326, 0.461)	0.383 (0.318, 0.449)
	Age group 25-29	0.533 (0.466, 0.6)	0.533 (0.469, 0.598)
	Age group 30-34	0.593 (0.526, 0.66)	0.592 (0.527, 0.656)
	Age group 35-39	0.552 (0.485, 0.62)	0.553 (0.488, 0.618)
	Age group 40-44	0.508 (0.44, 0.575)	0.509 (0.444, 0.574)

	Age group 45-49	0.197 (0.128, 0.266)	0.205 (0.138, 0.272)
Other Traditional<br>Preliminary RMSE = 0.0181<br>Final RMSE = 0.0195	Intercept	-4.417 (-4.91, -3.924)	-4.252 (-4.796, -3.709)
	SDI	-3.173 (-3.494, -2.852)	-3.316 (-3.633, -2.999)
	Age group 20-24	0.189 (0.117, 0.26)	0.179 (0.106, 0.252)
	Age group 25-29	0.341 (0.27, 0.412)	0.348 (0.276, 0.421)
	Age group 30-34	0.461 (0.39, 0.533)	0.47 (0.398, 0.543)
	Age group 35-39	0.592 (0.52, 0.663)	0.607 (0.534, 0.679)
	Age group 40-44	0.718 (0.646, 0.789)	0.723 (0.651, 0.796)
	Age group 45-49	0.615 (0.541, 0.688)	0.619 (0.544, 0.694)

$$logit\left(Contra_{method_{unpartnered}}\right) \sim \hat{\beta}SDI + \hat{\beta}logit\left(Contra_{method_{partnered}}\right) + \hat{\delta}as.factor(age\ group) + \gamma_l + \gamma_r + \gamma_{sr}$$

Where γ represents random effects for location, GBD region and GBD super-region, respectively.

Supplementary Table 11. ST-GPR model coefficients for by-method use among unpartnered women

Method	Variable	Preliminary Model Coefficients (95% UI)	Final Model Coefficients (95% UI)
Female Sterilisation<br>Preliminary RMSE = 0.0598<br>Final RMSE = 0.0575	Intercept	-0.509 (-0.973, -0.045)	-0.18 (-0.669, 0.309)
	SDI	-1.304 (-1.773, -0.836)	-1.545 (-2, -1.091)
	Female sterilisation use among partnered women, logit	0.902 (0.865, 0.938)	0.906 (0.87, 0.942)
	Age group 20-24	-0.28 (-0.376, -0.184)	-0.309 (-0.402, -0.216)
	Age group 25-29	-0.297 (-0.407, -0.187)	-0.337 (-0.445, -0.23)
	Age group 30-34	-0.213 (-0.348, -0.079)	-0.257 (-0.39, -0.125)
	Age group 35-39	-0.08 (-0.236, 0.075)	-0.115 (-0.269, 0.038)
	Age group 40-44	0.002 (-0.167, 0.172)	-0.04 (-0.207, 0.126)
	Age group 45-49	0.161 (-0.015, 0.336)	0.103 (-0.069, 0.276)
Male Sterilisation<br>Preliminary RMSE = 0.0089<br>Final RMSE = 0.0088	Intercept	-3.35 (-3.805, -2.895)	-3.174 (-3.586, -2.762)
	SDI	-0.07 (-0.618, 0.479)	-0.118 (-0.598, 0.362)

	Male sterilisation use among partnered women, logit	0.514 (0.472, 0.555)	0.525 (0.487, 0.562)
	Age group 20-24	0.021 (-0.078, 0.121)	-0.006 (-0.099, 0.088)
	Age group 25-29	-0.107 (-0.209, -0.005)	-0.129 (-0.225, -0.032)
	Age group 30-34	-0.081 (-0.188, 0.027)	-0.1 (-0.202, 0.002)
	Age group 35-39	-0.204 (-0.318, -0.091)	-0.231 (-0.338, -0.123)
	Age group 40-44	-0.137 (-0.255, -0.019)	-0.16 (-0.272, -0.048)
	Age group 45-49	-0.223 (-0.347, -0.099)	-0.244 (-0.361, -0.127)
IUD<br>Preliminary RMSE = 0.0297<br>Final RMSE = 0.0299	Intercept	-2.753 (-3.517, -1.988)	-2.33 (-3.104, -1.556)
	SDI	-1.417 (-2.045, -0.789)	-1.759 (-2.334, -1.184)
	IUD use among partnered women, partnered	0.588 (0.536, 0.641)	0.61 (0.56, 0.66)
	Age group 20-24	0.349 (0.237, 0.46)	0.281 (0.173, 0.389)
	Age group 25-29	0.645 (0.522, 0.768)	0.599 (0.479, 0.718)
	Age group 30-34	0.807 (0.676, 0.939)	0.763 (0.635, 0.891)
	Age group 35-39	0.848 (0.712, 0.984)	0.805 (0.673, 0.938)
	Age group 40-44	0.738 (0.603, 0.874)	0.699 (0.568, 0.83)
	Age group 45-49	0.586 (0.453, 0.719)	0.54 (0.412, 0.669)
Injections<br>Preliminary RMSE = 0.0718<br>Final RMSE = 0.0700	Intercept	-5.296 (-6.131, -4.462)	-5.045 (-5.813, -4.277)
	SDI	3.31 (2.586, 4.033)	3.066 (2.395, 3.736)
	Injections use among partnered women, logit	0.749 (0.706, 0.792)	0.748 (0.708, 0.789)
	Age group 20-24	0.889 (0.796, 0.981)	0.869 (0.779, 0.959)
	Age group 25-29	1.344 (1.251, 1.438)	1.322 (1.23, 1.413)
	Age group 30-34	1.457 (1.361, 1.553)	1.439 (1.345, 1.532)
	Age group 35-39	1.269 (1.171, 1.367)	1.255 (1.159, 1.351)
	Age group 40-44	1.056 (0.955, 1.157)	1.041 (0.942, 1.139)
	Age group 45-49	0.857 (0.743, 0.971)	0.84 (0.73, 0.951)
Implants<br>Preliminary RMSE = 0.0326<br>Final RMSE = 0.0326	Intercept	-2.932 (-3.752, -2.111)	-2.927 (-3.742, -2.113)
	SDI	1.327 (0.349, 2.304)	1.329 (0.369, 2.289)
	Implants use among partnered women, logit	0.813 (0.769, 0.856)	0.811 (0.768, 0.855)

	Age group 20-24	0.38 (0.245, 0.515)	0.376 (0.241, 0.511)
	Age group 25-29	0.582 (0.445, 0.72)	0.568 (0.431, 0.705)
	Age group 30-34	0.619 (0.478, 0.76)	0.603 (0.463, 0.744)
	Age group 35-39	0.409 (0.264, 0.555)	0.397 (0.253, 0.542)
	Age group 40-44	0.315 (0.165, 0.465)	0.309 (0.16, 0.458)
	Age group 45-49	0.352 (0.192, 0.513)	0.359 (0.2, 0.519)
Pill<br>Preliminary RMSE = 0.0582<br>Final RMSE = 0.0591	Intercept	-3.995 (-4.947, -3.042)	-3.801 (-4.651, -2.951)
	SDI	0.476 (-0.12, 1.072)	0.552 (0.008, 1.096)
	Pill use among partnered women, logit	0.525 (0.469, 0.582)	0.547 (0.496, 0.599)
	Age group 20-24	1.081 (0.982, 1.181)	1.045 (0.951, 1.139)
	Age group 25-29	1.377 (1.274, 1.48)	1.332 (1.235, 1.429)
	Age group 30-34	1.297 (1.192, 1.401)	1.255 (1.156, 1.354)
	Age group 35-39	1.071 (0.967, 1.175)	1.03 (0.931, 1.128)
	Age group 40-44	0.61 (0.505, 0.715)	0.575 (0.475, 0.675)
	Age group 45-49	0.242 (0.124, 0.361)	0.214 (0.1, 0.328)
Condom<br>Preliminary RMSE = 0.0724<br>Final RMSE = 0.0715	Intercept	-3.497 (-4.61, -2.385)	-3.719 (-4.746, -2.692)
	SDI	3.218 (2.489, 3.947)	3.717 (3.037, 4.396)
	Condom use among partnered women, logit	0.89 (0.821, 0.958)	0.876 (0.816, 0.937)
	Age group 20-24	0.665 (0.567, 0.762)	0.608 (0.514, 0.702)
	Age group 25-29	0.625 (0.527, 0.723)	0.588 (0.493, 0.683)
	Age group 30-34	0.33 (0.23, 0.431)	0.298 (0.201, 0.396)
	Age group 35-39	-0.013 (-0.116, 0.091)	-0.038 (-0.139, 0.062)
	Age group 40-44	-0.222 (-0.331, -0.113)	-0.251 (-0.356, -0.147)
	Age group 45-49	-0.416 (-0.539, -0.292)	-0.426 (-0.544, -0.307)
Diaphragm<br>Preliminary RMSE = 0.0125<br>Final RMSE = 0.0124	Intercept	-2.525 (-3.069, -1.982)	-2.213 (-2.767, -1.658)
	SDI	-0.235 (-0.677, 0.208)	0.033 (-0.54, 0.605)
	Diaphragm use among partnered women, logit	0.634 (0.557, 0.712)	0.703 (0.63, 0.776)
	Age group 20-24	0.302 (0.154, 0.451)	0.284 (0.145, 0.423)
	Age group 25-29	0.361 (0.211, 0.511)	0.333 (0.193, 0.474)
	Age group 30-34	0.268 (0.113, 0.422)	0.24 (0.096, 0.385)
	Age group 35-39	0.198 (0.039, 0.357)	0.18 (0.031, 0.329)

	Age group 40-44	0.149 (-0.016, 0.314)	0.124 (-0.03, 0.279)
	Age group 45-49	0.037 (-0.143, 0.217)	-0.007 (-0.176, 0.162)
Emergency Contraception<br>Preliminary RMSE = 0.0132<br>Final RMSE = 0.0131	Intercept	-5.077 (-6.825, -3.329)	-4.896 (-6.522, -3.27)
	SDI	1.484 (0.03, 2.938)	1.257 (-0.066, 2.579)
	Emergency contraception use among partnered women, logit	0.377 (0.171, 0.583)	0.384 (0.186, 0.581)
	Age group 20-24	0.811 (0.589, 1.033)	0.821 (0.605, 1.037)
	Age group 25-29	0.882 (0.659, 1.105)	0.894 (0.677, 1.111)
	Age group 30-34	0.407 (0.181, 0.634)	0.394 (0.175, 0.614)
	Age group 35-39	-0.03 (-0.27, 0.21)	-0.011 (-0.243, 0.221)
	Age group 40-44	0.12 (-0.132, 0.371)	0.106 (-0.135, 0.347)
	Age group 45-49	-0.224 (-0.481, 0.034)	-0.277 (-0.524, -0.03)
Other Modern<br>Preliminary RMSE = 0.0075<br>Final RMSE = 0.0073	Intercept	-3.239 (-3.894, -2.584)	-3.112 (-3.73, -2.495)
	SDI	0.211 (-0.369, 0.791)	0.425 (-0.155, 1.006)
	Other modern use among partnered women, logit	0.557 (0.478, 0.636)	0.589 (0.515, 0.662)
	Age group 20-24	0.221 (0.082, 0.361)	0.192 (0.058, 0.325)
	Age group 25-29	0.399 (0.258, 0.541)	0.39 (0.255, 0.524)
	Age group 30-34	0.165 (0.021, 0.309)	0.143 (0.005, 0.28)
	Age group 35-39	0.195 (0.048, 0.343)	0.186 (0.045, 0.326)
	Age group 40-44	0.056 (-0.093, 0.205)	0.042 (-0.1, 0.185)
	Age group 45-49	0.062 (-0.1, 0.224)	0.047 (-0.107, 0.202)
Lactational Amenorrhoea Method<br>Preliminary RMSE = 0.0143<br>Final RMSE = 0.0142	Intercept	-4.406 (-4.776, -4.036)	-4.001 (-4.355, -3.647)
	SDI	-1.283 (-1.882, -0.684)	-1.548 (-2.157, -0.938)
	LAM use among partnered women, logit	0.33 (0.271, 0.389)	0.377 (0.32, 0.434)
	Age group 20-24	0.285 (0.171, 0.399)	0.261 (0.147, 0.376)
	Age group 25-29	0.312 (0.197, 0.427)	0.287 (0.172, 0.403)
	Age group 30-34	0.388 (0.271, 0.504)	0.373 (0.257, 0.489)
	Age group 35-39	0.432 (0.311, 0.552)	0.437 (0.316, 0.558)
	Age group 40-44	0.499 (0.365, 0.633)	0.549 (0.415, 0.682)

	Age group 45-49	0.474 (0.315, 0.633)	0.561 (0.404, 0.719)
Rhythm<br>Preliminary RMSE = 0.0411<br>Final RMSE = 0.0410	Intercept	-1.486 (-2.149, -0.823)	-1.417 (-2.106, -0.728)
	SDI	-2.944 (-3.655, -2.232)	-3.034 (-3.701, -2.368)
	Rhythm use among partnered women, logit	0.585 (0.517, 0.652)	0.582 (0.518, 0.645)
	Age group 20-24	0.354 (0.238, 0.469)	0.337 (0.225, 0.449)
	Age group 25-29	0.393 (0.27, 0.517)	0.388 (0.268, 0.507)
	Age group 30-34	0.27 (0.139, 0.402)	0.265 (0.138, 0.392)
	Age group 35-39	0.032 (-0.108, 0.171)	0.026 (-0.109, 0.16)
	Age group 40-44	-0.305 (-0.45, -0.16)	-0.304 (-0.443, -0.164)
	Age group 45-49	-0.477 (-0.622, -0.332)	-0.458 (-0.598, -0.318)
Withdrawal<br>Preliminary RMSE = 0.0158<br>Final RMSE = 0.0156	Intercept	-5.319 (-5.846, -4.793)	-5.085 (-5.58, -4.589)
	SDI	1.826 (1.215, 2.437)	1.732 (1.169, 2.294)
	Withdrawal use among partnered women, logit	0.416 (0.36, 0.472)	0.447 (0.394, 0.499)
	Age group 20-24	0.394 (0.286, 0.503)	0.361 (0.257, 0.465)
	Age group 25-29	0.359 (0.249, 0.469)	0.334 (0.228, 0.439)
	Age group 30-34	0.154 (0.041, 0.268)	0.138 (0.029, 0.247)
	Age group 35-39	-0.065 (-0.181, 0.051)	-0.081 (-0.193, 0.03)
	Age group 40-44	-0.191 (-0.311, -0.071)	-0.195 (-0.309, -0.08)
	Age group 45-49	-0.272 (-0.399, -0.146)	-0.264 (-0.386, -0.142)
Other Traditional<br>Preliminary RMSE = 0.0158<br>Final RMSE = 0.0160	Intercept	-3.016 (-3.532, -2.5)	-2.825 (-3.298, -2.353)
	SDI	-0.583 (-1.216, 0.05)	-0.647 (-1.262, -0.032)
	Other traditional use among partnered women, logit	0.524 (0.457, 0.591)	0.545 (0.484, 0.607)
	Age group 20-24	0.172 (0.057, 0.287)	0.154 (0.042, 0.267)
	Age group 25-29	0.175 (0.057, 0.294)	0.136 (0.021, 0.252)
	Age group 30-34	0.113 (-0.011, 0.238)	0.061 (-0.06, 0.182)
	Age group 35-39	0.095 (-0.035, 0.225)	0.048 (-0.079, 0.176)
	Age group 40-44	0.101 (-0.039, 0.24)	0.054 (-0.082, 0.19)
	Age group 45-49	-0.046 (-0.192, 0.1)	-0.108 (-0.25, 0.035)

$$logit\left(Prop_{unmet_{partnered}}\right) \sim \hat{\beta}SDI + \hat{\beta}logit\left(Contra_{any_{partnered}}\right) + \hat{\delta}as.factor(age\ group) + \gamma_l + \gamma_r + \gamma_{sr}$$

Where γ represents random effects for location, GBD region and GBD super-region, respectively.

Supplementary Table 12. ST-GPR model coefficients for proportion unmet need among partnered non-users

Preliminary RMSE = 0.1479; Final RMSE = 0.1494

Variable	Preliminary Model Coefficients (95% UI)	Final Model Coefficients (95% UI)
Intercept	0.037 (-0.196, 0.269)	0.09 (-0.152, 0.331)
SDI	-1.165 (-1.422, -0.908)	-1.273 (-1.524, -1.022)
Any contraceptive use among partnered women, logit	0.075 (0.044, 0.106)	0.086 (0.056, 0.116)
Age group 20-24	0.062 (0.017, 0.108)	0.055 (0.01, 0.099)
Age group 25-29	0.096 (0.046, 0.147)	0.085 (0.036, 0.134)
Age group 30-34	0.186 (0.132, 0.24)	0.17 (0.117, 0.223)
Age group 35-39	0.314 (0.259, 0.369)	0.304 (0.25, 0.357)
Age group 40-44	0.235 (0.183, 0.287)	0.228 (0.177, 0.278)
Age group 45-49	-0.386 (-0.43, -0.342)	-0.378 (-0.421, -0.334)

$$logit\left(Prop_{unmet_{unpartnered}}\right) \sim \hat{\beta}SDI + \hat{\beta}logit\left(Contra_{any_{unpartnered}}\right) + \hat{\beta}logit\left(Prop_{unmet_{partnered}}\right) + \hat{\delta}as.factor(age\ group) + \gamma_l + \gamma_r + \gamma_{sr}$$

Where γ represents random effects for location, GBD region and GBD super-region, respectively.

Supplementary Table 13. ST-GPR model coefficients for proportion unmet need among unpartnered non-users

Preliminary RMSE = 0.0691; Final RMSE = 0.0710

Variable	Preliminary Model Coefficients (95% UI)	Final Model Coefficients (95% UI)
Intercept	-3.229 (-4.288, -2.169)	-3.293 (-4.329, -2.256)
SDI	0.739 (0.049, 1.429)	0.837 (0.132, 1.541)
Any contraceptive use among partnered women, logit	0.275 (0.227, 0.322)	0.241 (0.193, 0.29)
Proportion unmet need among partnered non-users, logit	0.559 (0.465, 0.653)	0.631 (0.538, 0.725)
Age group 20-24	0.469 (0.35, 0.589)	0.516 (0.394, 0.638)
Age group 25-29	0.41 (0.279, 0.542)	0.511 (0.377, 0.645)
Age group 30-34	0.259 (0.127, 0.391)	0.369 (0.235, 0.503)
Age group 35-39	-0.075 (-0.205, 0.054)	0.022 (-0.11, 0.153)

Age group 40-44	-0.365 (-0.487, -0.244)	-0.286 (-0.41, -0.162)
Age group 45-49	-0.73 (-0.853, -0.608)	-0.618 (-0.741, -0.495)

Section 4. Post-modelling processing

Method mix squeezing

To ensure the estimates from the 14 individual method models for partnered and unpartnered women were consistent with our any contraceptive use estimates, we calculated and applied an adjustment factor to the by-method estimates to ensure their sum equals any contraceptive use. Adjustments were made at the 1000 draw level before taking the mean to retain uncertainty with the following equations:

$$Adjustment\ factor = 1 + \frac{Contra_{any} - \sum Contra_{method}}{\sum Contra_{method}}$$

$$Contra_{method} = Contra_{method} * Adjustment\ factor$$

where $Contra_{any}$ is any contraceptive prevalence and $Contra_{method}$ is method-specific prevalence.

Age-standardisation

Age-standardised results for women 15-49 were calculated at the 1000 draw level by multiplying each draw in an age group by a set age weight. Shown in Table 13, the age weight for each age group is normalised to the population 15-49 so that the weights add up to 1 using the GBD 2019 world population age standard. Additional details on the GBD world population standard can be found in Appendix 1, Section 5.4.5 of the GBD 2019 Demographics Capstone.

Supplementary Table 14. Age group weights for age-standardisation

Age Group	Weight
15-19	0.1678325
20-24	0.1585875
25-29	0.1538938
30-34	0.1477765
35-39	0.1372288
40-44	0.1236737
45-49	0.1110073

Global, super-regional and SDI quintile aggregation

Aggregation to the global, super-regional and SDI quintile levels was done at the draw level in count space using age-sex-specific GBD population estimates in combination with our marital status estimates to ensure population counts matched the denominator of the indicator. As such, in order to aggregate demand satisfied with modern methods, population counts were also multiplied by the proportion of women with a need for family planning. The cutoffs for each SDI quintile are displayed below.

Supplementary Table 15. SDI quintile cutoffs for GBD 2019

SDI Quintile	Lower Bound	Upper Bound
Low	0	0.4547428
Low-middle	0.4547428	0.6076789

Middle	0.6076789	0.6895037
Middle-high	0.6895037	0.8051292
High	0.8051292	1

* Lower bounds are inclusive.

Information on which countries and territories are contained in each super-region can be found in the Appendix, Page 6 of GBD 2010[8]. The methodology for determining the SDI quintiles can be found in Appendix 1, Section 6.2 of the GBD 2019 Demographics Capstone[7].

Uncertainty calculation

ST-GPR outputs 1000 draws for every location, year and 5-year age group which are used to calculate 95% uncertainty intervals using the 0.025 and 0.975 quantiles. All marital- and age-splitting was done at the draw level to propagate uncertainty throughout the process. Modern contraceptive prevalence, demand satisfied, and unmet need, plus all women estimates, were calculated by combining the corresponding ordinal draw from each model to preserve uncertainty.

Part 2. Additional analyses

Section 1. Evaluation of FP2020 Goal

We evaluated the progress of 68 of the 69 FP2020 priority countries (https://fp2030.org/countries) between 2012 and 2019, excluding Western Sahara which is not a location included in the GBD. We calculated the baseline 2012 and final 2019 counts of women of reproductive age using modern contraception by multiplying our all-age 15-49 mCPR estimates by the GBD population estimates for women aged 15-49 in each year at the draw level. We then summed the counts of modern contraceptive users in the 68 countries for each draw, and took the mean, 0.025 quantile and 0.975 quantile across all 1000 draws to produce the final point estimates and uncertainty intervals.

In order to calculate the additional number of modern contraceptive users between 2012 and 2019, we found the difference between the total users in each year at the draw level before taking the mean, 0.025 quantile and 0.975 quantile of the differences across all 1000 draws to produce the final point estimate and uncertainty interval.

Following the groupings of countries into low, medium and high historical mCPR growth set by the FP2020 Family Planning Metrics Team, we investigated if annual percentage point changes in mCPR met the projected rates of growth used to set the goal of 120 million additional modern contraceptive users[9]. We included countries that were within 0.2 percentage points of the projected rates of growth as meeting the targets. The table below shows the countries in each growth bin, the FP 2020 projected rates of change, and our estimated annual percentage point changes in mCPR for the years 2012-2019.

Supplementary Table 16. Projected and estimated annual percentage point changes in mCPR in 68 FP2020 priority countries

		2012 - 2015					2016 - 2019				
Growth Bin	Country	Projected	2012	2013	2014	2015	Projected	2016	2017	2018	2019
Low	Afghanistan	1.3	0.19	0.08	0.03	0.01	1.8	0.09	0.17	0.22	0.28
	Bangladesh	1.3	0.39	0.18	0.1	0.14	1.8	0.19	0.34	0.74	0.97

	Benin	1.3	0.43	0.53	0.55	0.42	1.8	0.37	0.39	0.47	0.57
	Bolivia	1.3	0.99	1.13*	1.21*	1.23*	1.8	1.17	0.74	0.58	0.48
	Burkina Faso	1.3	0.73	1.01	1.36*	1.86*	1.8	2.27*	2.29*	1.9*	1.27*
	Cameroon	1.3	0.6	0.61	0.42	0	1.8	-0.27	-0.37	-0.24	0.27
	Central African Republic	1.3	0.14	-0.15	-0.04	0.1	1.8	0.21	0.29	0.37	0.17
	Chad	1.3	0.16	0.17	0.19	0.17	1.8	0.15	0.13	0.14	0.15
	Côte d'Ivoire	1.3	0.09	0.15	0.3	0.55	1.8	0.88	1.3	1.39	1.27
	DR Congo	1.3	0.78	1.03	1.3*	1.36*	1.8	1.15	1.01	1.07	1.1
	Egypt	1.3	0.33	0.48	0.68	1.22*	1.8	1.41	1.5	1.51	1.47
	Eritrea	1.3	0.25	0.22	0.36	0.26	1.8	0.29	0.25	0.3	0.3
	Guinea	1.3	0.11	0.16	0.2	0.24	1.8	0.31	0.46	0.53	0.52
	Guinea-Bissau	1.3	0.79	0.8	0.79	0.75	1.8	0.84	0.92	0.92	0.76
	Haiti	1.3	0.43	0.32	0.3	0.27	1.8	0.25	0.2	0.24	0.23
	India	1.3	-0.03	-0.05	-0.03	0.1	1.8	0.61	0.81	0.99	1.09
	Indonesia	1.3	-0.21	-0.22	-0.21	-0.13	1.8	0.04	0.33	0.62	0.72
	Kyrgyzstan	1.3	-0.15	0.14	0.22	0.36	1.8	0.44	0.49	0.53	0.65
	Liberia	1.3	1.2*	1.29*	1	0.88	1.8	0.73	0.66	0.57	0.46
	Mali	1.3	0.73	0.87	1	1.03	1.8	0.81	0.68	0.54	0.29
	Mauritania	1.3	0.56	0.63	0.67	0.67	1.8	0.44	0.36	0.28	0.22
	Nepal	1.3	-0.47	-0.48	-0.44	-0.41	1.8	-0.34	-0.03	0.16	0.39
	Niger	1.3	0.83	1.1	1.26*	1.35*	1.8	1.36	1.17	0.6	0.24
	Nigeria	1.3	0.57	0.73	0.77	0.48	1.8	0.05	0.11	0.18	0.34
	North Korea	1.3	0.61	0.58	0.54	0.33	1.8	0.33	0.36	0.45	0.5
	Pakistan	1.3	0.38	0.62	0.22	0.27	1.8	0.33	0.41	0.76	0.85
	Philippines	1.3	0.32	0.27	0.22	0.21	1.8	0.23	0.32	0.45	0.55
	Senegal	1.3	1.15*	1.3*	1.36*	1.3*	1.8	1.11	0.83	0.49	0.26
	Somalia	1.3	0.02	-0.02	-0.05	-0.09	1.8	-0.12	-0.13	-0.11	0.02
	South Sudan	1.3	0	0.02	0.02	0	1.8	-0.02	-0.01	-0.01	0
	Sri Lanka	1.3	0.13	0.16	0.21	0.25	1.8	0.28	0.4	0.39	0.38
	Sudan	1.3	0.3	0.35	0.39	0.42	1.8	0.45	0.47	0.46	0.44
	Tajikistan	1.3	-0.09	0	0.06	0.12	1.8	0.22	0.3	0.45	0.51
	Togo	1.3	0.59	0.74	0.83	0.89	1.8	0.91	0.9	0.78	0.75
	Uzbekistan	1.3	0.74	0.8	0.84	0.85	1.8	0.84	0.79	0.73	0.66
	Zimbabwe	1.3	1.15*	1.23*	1.14*	0.79	1.8	0.47	0.33	0.23	0.02
Medium	Bhutan	1.8	0.62	0.66	0.63	0.62	2.3	0.62	0.61	0.62	0.61
	Congo (Brazzaville)	1.8	0.7	0.54	0.6	0.56	2.3	0.49	0.59	0.54	0.53
	Malawi	1.8	1.94*	1.78*	1.23	0.91	2.3	0.27	0.02	-0.1	-0.11
	Myanmar	1.8	0.58	0.52	0.48	0.45	2.3	0.29	0.27	0.24	0.22
	Nicaragua	1.8	0.61	0.55	0.5	0.48	2.3	0.46	0.43	0.3	0.2
	Palestine	1.8	0.24	0.21	0.18	0.26	2.3	0.31	0.32	0.37	0.44

	Papua New Guinea	1.8	0.3	0.3	0.41	0.47	2.3	0.49	0.49	0.44	0.46
	Sierra Leone	1.8	1.94*	2.19*	1.82*	1.25	2.3	1.07	0.81	0.26	0.12
	Solomon Islands	1.8	-0.04	-0.02	-0.02	0.02	2.3	0.26	0.34	0.4	0.42
	The Gambia	1.8	0.08	0.16	0.29	0.39	2.3	0.46	0.53	0.59	0.59
	Vietnam	1.8	0.08	0.17	0.5	0.68	2.3	0.88	0.96	0.96	0.92
	Zambia	1.8	1.58	1.43	1.03	0.8	2.3	0.65	0.56	0.48	0.29
High	Burundi	2.3	0.56	0.58	0.55	0.41	2.8	0.36	0.57	0.52	0.49
	Cambodia	2.3	0.82	0.82	0.81	0.72	2.8	0.69	0.64	0.61	0.57
	Comoros	2.3	0.12	0.48	0.56	0.61	2.8	0.66	0.69	0.71	0.7
	Djibouti	2.3	0.35	0.3	0.3	0.34	2.8	0.39	0.33	0.34	0.34
	Ethiopia	2.3	0.81	0.75	0.7	0.56	2.8	0.51	0.49	0.55	0.56
	Ghana	2.3	0.13	0.08	0.42	0.82	2.8	0.88	0.94	1.04	1.1
	Honduras	2.3	0.34	0.21	0.15	0.13	2.8	0.15	0.21	0.27	0.33
	Iraq	2.3	0.22	0.28	0.23	0.22	2.8	0.3	0.31	0.36	0.6
	Kenya	2.3	1.69	0.99	0.7	0.73	2.8	0.59	0.57	0.62	0.64
	Laos	2.3	1.04	1.06	1.05	1.03	2.8	1.05	1.01	0.81	0.73
	Lesotho	2.3	1.85	1.42	1.13	0.82	2.8	0.71	0.56	0.46	0.14
	Madagascar	2.3	1.15	0.92	0.95	0.96	2.8	0.98	1	0.98	0.61
	Mongolia	2.3	0.07	0.04	0.22	0.18	2.8	0.16	0.13	0.22	0.67
	Mozambique	2.3	1.19	1.56	1.75	1.72	2.8	1.11	0.78	0.52	0.33
	Rwanda	2.3	1.35	1.2	1.17	0.98	2.8	0.94	0.86	0.81	0.81
	São Tomé and Príncipe	2.3	0.89	0.95	1	0.96	2.8	1.05	1.05	1.01	0.94
	Tanzania	2.3	0.57	0.54	0.59	0.63	2.8	0.59	0.77	0.83	0.85
	Timor-Leste	2.3	0.31	0.26	0.25	0.23	2.8	0.25	0.23	0.23	0.29
	Uganda	2.3	0.83	0.96	1.23	1.65	2.8	1.75	1.66	1.51	1.3
	Yemen	2.3	0.6	0.58	0.47	0.27	2.8	0.18	0.13	0.12	0.12

* country-years in which the target was met within 0.2 percentage points

Additionally, we compared the average rates of change in percentage points in mCPR between 2004-2011 and 2012-2019 to examine if FP2020 countries outperformed historical rates. 10 countries experienced a statistically significant increase in mCPR growth rates, 51 countries with overlapping uncertainty intervals were considered to have sustained growth, and 7 countries experienced a statistically significant decrease. Countries grouped into these three categories and the mCPR growth rates for each 7-year time period are included in the table below.

Supplementary Table 17. Average mCPR percentage point change between 2004-2011 and 2012-2019 in 68 FP2020 priority countries

Growth	Country	2004-2011 Average Growth Rate (95% UI)	2012-2019 Average Growth Rate (95% UI)
Increased	Benin	0.21 (0.15, 0.3)	0.47 (0.37, 0.57)
	Burkina Faso	0.53 (0.33, 0.72)	1.59 (0.73, 2.29)

	Comoros	-0.01 (-0.08, 0.05)	0.57 (0.12, 0.71)
	DR Congo	0.17 (0.05, 0.55)	1.1 (0.78, 1.36)
	Eritrea	0.04 (-0.04, 0.21)	0.28 (0.22, 0.36)
	Kyrgyzstan	-0.53 (-0.76, -0.24)	0.34 (-0.15, 0.65)
	Sudan	0.2 (0.14, 0.28)	0.41 (0.3, 0.47)
	The Gambia	-0.05 (-0.11, 0.04)	0.38 (0.08, 0.59)
	Togo	0.07 (-0.26, 0.5)	0.8 (0.59, 0.91)
	Uzbekistan	0.2 (-0.16, 0.65)	0.78 (0.66, 0.85)
Sustained	Afghanistan	0.56 (0.23, 0.75)	0.13 (0.01, 0.28)
	Bangladesh	0.44 (0.18, 0.89)	0.38 (0.1, 0.97)
	Bhutan	0.84 (0.6, 0.94)	0.62 (0.61, 0.66)
	Bolivia	0.54 (0.4, 0.84)	0.94 (0.48, 1.23)
	Burundi	0.09 (-0.62, 0.54)	0.51 (0.36, 0.58)
	Cameroon	0.5 (0.29, 0.95)	0.13 (-0.37, 0.61)
	Central African Republic	0.26 (0.02, 0.65)	0.14 (-0.15, 0.37)
	Chad	0.12 (0.07, 0.17)	0.16 (0.13, 0.19)
	Congo (Brazzaville)	0.77 (0.43, 0.9)	0.57 (0.49, 0.7)
	Côte d'Ivoire	0.16 (0.08, 0.26)	0.74 (0.09, 1.39)
	Djibouti	0.52 (0.39, 0.69)	0.34 (0.3, 0.39)
	Egypt	0.24 (0.15, 0.39)	1.08 (0.33, 1.51)
	Ghana	0.38 (0.07, 0.65)	0.67 (0.08, 1.1)
	Guinea	0.12 (0.02, 0.3)	0.32 (0.11, 0.53)
	Guinea-Bissau	0.8 (0.6, 0.93)	0.82 (0.75, 0.92)
	India	0.31 (0.05, 0.77)	0.43 (-0.05, 1.09)
	Indonesia	0.13 (-0.13, 0.36)	0.12 (-0.22, 0.72)
	Iraq	0.47 (0.14, 0.81)	0.32 (0.22, 0.6)
	Kenya	1.67 (0.48, 2.59)	0.82 (0.57, 1.69)
	Laos	0.92 (0.77, 1.04)	0.97 (0.73, 1.06)
	Lesotho	1.93 (0.94, 2.72)	0.89 (0.14, 1.85)
	Liberia	0.83 (0.56, 1.11)	0.85 (0.46, 1.29)
	Madagascar	1.3 (1.13, 1.49)	0.94 (0.61, 1.15)
	Malawi	1.65 (1.23, 1.99)	0.74 (-0.11, 1.94)
	Mali	0.33 (0.16, 0.6)	0.74 (0.29, 1.03)
	Mauritania	0.28 (0.19, 0.4)	0.48 (0.22, 0.67)
	Mongolia	-0.2 (-0.4, 0.23)	0.21 (0.04, 0.67)
	Mozambique	0.18 (-0.12, 0.69)	1.12 (0.33, 1.75)
	Nepal	0.47 (-0.42, 1.14)	-0.2 (-0.48, 0.39)
	Niger	0.35 (0.14, 0.64)	0.99 (0.24, 1.36)
	Nigeria	0.37 (0.29, 0.5)	0.4 (0.05, 0.77)
	North Korea	0.53 (0.36, 0.64)	0.46 (0.33, 0.61)
	Pakistan	0.35 (0.1, 0.6)	0.48 (0.22, 0.85)
	Palestine	0.27 (0.2, 0.36)	0.29 (0.18, 0.44)
	Papua New Guinea	0.32 (0.27, 0.38)	0.42 (0.3, 0.49)

	Philippines	0.27 (0.22, 0.34)	0.32 (0.21, 0.55)
	Rwanda	1.72 (1.01, 2.16)	1.02 (0.81, 1.35)
	Senegal	0.4 (0.17, 0.92)	0.97 (0.26, 1.36)
	Sierra Leone	1.02 (0.43, 1.67)	1.18 (0.12, 2.19)
	Solomon Islands	0.1 (-0.03, 0.26)	0.17 (-0.04, 0.42)
	Somalia	0.05 (0.02, 0.07)	-0.06 (-0.13, 0.02)
	South Sudan	0.05 (0.02, 0.08)	0 (-0.02, 0.02)
	Sri Lanka	0.08 (-0.03, 0.23)	0.28 (0.13, 0.4)
	São Tomé and Príncipe	0.8 (0.56, 0.92)	0.98 (0.89, 1.05)
	Tajikistan	-0.13 (-0.22, 0.01)	0.2 (-0.09, 0.51)
	Tanzania	0.74 (0.55, 0.88)	0.67 (0.54, 0.85)
	Timor-Leste	0.43 (0.24, 0.54)	0.26 (0.23, 0.31)
	Uganda	0.72 (0.38, 0.87)	1.36 (0.83, 1.75)
	Vietnam	0.49 (0.18, 0.74)	0.64 (0.08, 0.96)
	Zambia	1.05 (0.71, 1.58)	0.85 (0.29, 1.58)
	Zimbabwe	0.61 (0.35, 0.84)	0.67 (0.02, 1.23)
Decreased	Cambodia	0.96 (0.83, 1.03)	0.71 (0.57, 0.82)
	Ethiopia	1.33 (0.91, 1.75)	0.62 (0.49, 0.81)
	Haiti	0.54 (0.45, 0.6)	0.28 (0.2, 0.43)
	Honduras	0.8 (0.65, 0.87)	0.23 (0.13, 0.34)
	Myanmar	0.85 (0.65, 0.96)	0.38 (0.22, 0.58)
	Nicaragua	0.92 (0.84, 0.97)	0.44 (0.2, 0.61)
	Yemen	0.77 (0.65, 0.89)	0.31 (0.12, 0.6)

Section 2. Expected values of mCPR and demand satisfied given SDI

We modelled the expected relationship between SDI and modern contraceptive prevalence and demand satisfied with modern methods depicted in Figure 5 using meta-regression – Bayesian, regularised, trimmed (MR-BRT). We first logit-transformed the CPR and demand satisfied estimates and standard errors, then ran a separate MR-BRT regression for each age group. The MR-BRT model settings used for each regression are displayed in the table below. After running the regression, we took 1000 samples of the betas and gammas, and generated 1000 draws from which we predicted out expected estimates of CPR and demand satisfied for each observed value of SDI. Lastly, we transformed the predictions and their standard errors back into linear space.

Details on the statistical models underlying MR-BRT and the fitting procedure used to obtain estimates can be found in Appendix 1, Section 4.4 of the GBD 2019 risk factors capstone[6]. Further details on models and algorithms can be found in the technical reports[10].

Supplementary Table 18. MR-BRT spline constraints and priors

Constraint/Prior	Setting	Interpretation
**Degrees**	Cubic	Fit a cubic spline
**Monotonicity**	Increasing	Force spline to be increasing
**Knots**	0, .25, .5, .75, 1	Knots placed at the minimum, maximum, and quartiles of SDI
**Knot type**	Domain	Knots placed proportionally between the minimum and maximum values of SDI
**Right tail**	Linear	Linear right tail on spline
**Left tail**	Linear	Linear left tail on spline
**Trimming**	5%	Trim 5% of data points out; removes outliers
**Random effects**	None	No random effects on the intercept nor SDI

Section 3. Comparison of IHME and UN-DESA Estimates

Overview

IHME and UN-DESA both produce complete time series of key family planning indicators for all, partnered and unpartnered women of reproductive age aged 15-49. Family planning indicators estimated include any contraceptive prevalence, modern contraceptive prevalence, traditional contraceptive prevalence, unmet need for any method, unmet need for a modern method, total need/demand for family planning and demand satisfied for family planning with modern methods. All IHME results are available for the time range 1970-2019 and by 5-year age bin in addition to all ages 15-49 and age-standardized 15-49 results. UN-DESA results are available for 1970-2030 for married women and 1990-2030 for all and unmarried women; the only publicly available age group is all ages 15-49.

2019 Estimates

(grey)	Reference
(red)	Lower, statistically significant
(green)	Similar (overlapping 95% UI's)
(blue)	Higher, statistically significant

Supplementary Table 19. Comparison of IHME and UN-DESA family planning estimates for all women

	IHME		UN-DESA	
	%	# (millions)	%	# (millions)
Any contraceptive prevalence	51.9<br>(51, 52.8)	1013.2<br>(995, 1030.7)	48.6<br>(46.5, 51.4)	924.4<br>(883.7, 978.1)
Modern contraceptive prevalence	47.7<br>(46.9, 48.6)	931.1<br>(914.6, 947.7)	44.1<br>(42.1, 46.7)	838.9<br>(800.7, 889.3)
Demand satisfied with modern methods	79.2<br>(78.5, 79.8)	931.1<br>(914.6, 947.7)	76.7<br>(74.3, 78.6)	838.9<br>(800.7, 889.3)
Unmet need for any contraceptive method	8.3<br>(8, 8.7)	162.9<br>(155.6, 170.2)	8.9<br>(8.1, 10.3)	169.8<br>(154, 196.7)

Total need for family planning	60.3 (59.6, 60.9)	1176.1 (1162.5, 1188.8)	57.5 (55.6, 60.2)	1094.2 (1057.1, 1145.4)

Supplementary Table 19. Comparison of IHME and UN-DESA family planning estimates for partnered women

	IHME		UN-DESA	
	%	# (millions)	%	# (millions)
Any contraceptive prevalence	67.3 (65.8, 68.6)	867.1 (848.7, 884.6)	62.4 (60.2, 64.7)	779.4 (750.9, 807.5)
Modern contraceptive prevalence	61.5 (60.2, 62.7)	792.7 (776.5, 808.7)	56.6 (54.4, 58.7)	705.9 (678.4, 733)
Demand satisfied with modern methods	78.5 (77.7, 79.3)	792.7 (776.5, 808.7)	76.7 (74.7, 78.6)	705.9 (678.4, 733)
Unmet need for any contraceptive method	11.0 (10.5, 11.6)	142.3 (135.2, 149.4)	11.3 (10.3, 12.4)	141 (128.3, 155.3)
Total need for family planning	78.3 (77.3, 79.2)	1009.3 (996.4, 1021.1)	73.7 (72.1, 75.4)	920.3 (899.6, 920.3)

Supplementary Table 20. Comparison of IHME and UN-DESA family planning estimates for unpartnered women

	IHME		UN-DESA	
	%	# (millions)	%	# (millions)
Any contraceptive prevalence	22.1 (21.4, 22.8)	146.2 (141.5, 151.2)	22.2 (18.1, 29.1)	145.1 (118.1, 190.3)
Modern contraceptive prevalence	20.9 (20.2, 21.6)	138.4 (133.7, 143.2)	20.3 (16.6, 26.9)	133 (108.5, 175.8)
Demand satisfied with modern methods	82.9 (82.4, 83.5)	138.4 (133.7, 143.2)	76.5 (66.2, 83.2)	133 (108.5, 175.8)
Unmet need for any contraceptive method	3.1 (3, 3.2)	20.6 (19.8, 21.4)	4.4 (3.1, 8.1)	28.8 (20.3, 53.3)
Total need for family planning	25.2 (24.5, 25.9)	166.7 (162, 171.6)	26.6 (22.1, 33.6)	173.9 (144.6, 219.7)

Definitions and Data Processing

The main definitional difference between IHME and UN-DESA family planning indicators pertains to the classification of LAM as modern or traditional. We consider LAM to be a traditional method, while UN-DESA classifies it as a modern method. We employ the same calculation of need for family planning.

Methods

There are numerous methodological differences between how IHME and UN-DESA arrive at estimates for the same set of family planning indicators.[11-13]

- Country specific asymptotes
- Pace parameters unique to countries, sub region and regions

IHME and UN-DESA employ similar functional forms.

Similarities:

- Functional form: any contraceptive use, proportion unmet need among non-users

Differences:

- Functional form: Individual method prevalence rather than proportion modern among users
- UN-DESA modeled using logistic curve of asymptote, pace of transition and timing of transition; did not assume countries progress through the entire curve; auto-correlated error processes allowed the model to deviate from the smooth logistic curve when data was available; married model drew strength from super region-region hierarchies, unmarried model drew strength from super region-region-sexual activity level hierarchies; applied misclassification parameters and multipliers for bias due to sampling of non-baseline groups and misclassification; added normally distributed random error to estimates by survey type;

Part 3. Online tools and glossary of terms

Section 1. Online tools

Data for all family planning indicators can be located and downloaded here: http://ghdx.healthdata.org/gbd-2019.

Section 2. List of abbreviations

CPR – Contraceptive Prevalence Rate
GATHER – Guidelines for Accurate and Transparent Health Estimates Reporting
GBD – Global Burden of Diseases, Injuries, and Risk Factors Study
GHDx – Global Health Data Exchange
LDI – Lag-distributed Income
mCPR – Modern Contraceptive Prevalence Rate
SDI – Socio-demographic Index
ST-GPR – spatiotemporal Gaussian process regression
UI – Uncertainty Interval
UN-DESA – Department of Economic and Social Affairs of the United Nations

Section 3. List of ISO3 code and location names

ABW Aruba
AFG Afghanistan
AGO Angola
AIA Anguilla
ALA Åland Islands
ALB Albania
AND Andorra
ANT Netherlands Antilles
ARE United Arab Emirates
ARG Argentina
ARM Armenia
ASM American Samoa
ATA Antarctica
ATF French Southern Territories
ATG Antigua and Barbuda
AUS Australia
AUT Austria
AZE Azerbaijan
BDI Burundi
BEL Belgium
BEN Benin
BFA Burkina Faso
BGD Bangladesh
BGR Bulgaria
BHR Bahrain
BHS Bahamas
BIH Bosnia and Herzegovina
BLM Saint Barthélemy

BLR Belarus
BLZ Belize
BMU Bermuda
BOL Bolivia, Plurinational State of
BRA Brazil
BRB Barbados
BRN Brunei Darussalam
BTN Bhutan
BVT Bouvet Island
BWA Botswana
CAF Central African Republic
CAN Canada
CCK Cocos (Keeling) Islands
CHE Switzerland
CHL Chile
CHN China
CIV Côte d'Ivoire
CMR Cameroon
COD Congo, the Democratic Republic of the
COG Congo
COK Cook Islands
COL Colombia
COM Comoros
CPV Cape Verde
CRI Costa Rica
CUB Cuba
CXR Christmas Island
CYM Cayman Islands
CYP Cyprus
CZE Czech Republic
DEU Germany
DJI Djibouti
DMA Dominica
DNK Denmark
DOM Dominican Republic
DZA Algeria
ECU Ecuador
EGY Egypt
ERI Eritrea
ESH Western Sahara
ESP Spain
EST Estonia
ETH Ethiopia
FIN Finland
FJI Fiji
FLK Falkland Islands (Malvinas)
FRA France
FRO Faroe Islands

FSM Micronesia, Federated States of
GAB Gabon
GBR United Kingdom
GEO Georgia
GGY Guernsey
GHA Ghana
GIB Gibraltar
GIN Guinea
GLP Guadeloupe
GMB Gambia
GNB Guinea-Bissau
GNQ Equatorial Guinea
GRC Greece
GRD Grenada
GRL Greenland
GTM Guatemala
GUF French Guiana
GUM Guam
GUY Guyana
HKG Hong Kong
HMD Heard Island and McDonald Islands
HND Honduras
HRV Croatia
HTI Haiti
HUN Hungary
IDN Indonesia
IMN Isle of Man
IND India
IOT British Indian Ocean Territory
IRL Ireland
IRN Iran, Islamic Republic of
IRQ Iraq
ISL Iceland
ISR Israel
ITA Italy
JAM Jamaica
JEY Jersey
JOR Jordan
JPN Japan
KAZ Kazakhstan
KEN Kenya
KGZ Kyrgyzstan
KHM Cambodia
KIR Kiribati
KNA Saint Kitts and Nevis
KOR Korea, Republic of
KWT Kuwait
LAO Lao People's Democratic Republic

LBN Lebanon
LBR Liberia
LBY Libyan Arab Jamahiriya
LCA Saint Lucia
LIE Liechtenstein
LKA Sri Lanka
LSO Lesotho
LTU Lithuania
LUX Luxembourg
LVA Latvia
MAC Macao
MAF Saint Martin (French part)
MAR Morocco
MCO Monaco
MDA Moldova, Republic of
MDG Madagascar
MDV Maldives
MEX Mexico
MHL Marshall Islands
MKD Macedonia, the former Yugoslav Republic of
MLI Mali
MLT Malta
MMR Myanmar
MNE Montenegro
MNG Mongolia
MNP Northern Mariana Islands
MOZ Mozambique
MRT Mauritania
MSR Montserrat
MTQ Martinique
MUS Mauritius
MWI Malawi
MYS Malaysia
MYT Mayotte
NAM Namibia
NCL New Caledonia
NER Niger
NFK Norfolk Island
NGA Nigeria
NIC Nicaragua
NIU Niue
NLD Netherlands
NOR Norway
NPL Nepal
NRU Nauru
NZL New Zealand
OMN Oman
PAK Pakistan

PAN Panama
PCN Pitcairn
PER Peru
PHL Philippines
PLW Palau
PNG Papua New Guinea
POL Poland
PRI Puerto Rico
PRK Korea, Democratic People's Republic of
PRT Portugal
PRY Paraguay
PSE Palestinian Territory, Occupied
PYF French Polynesia
QAT Qatar
REU Réunion
ROU Romania
RUS Russian Federation
RWA Rwanda
SAU Saudi Arabia
SDN Sudan
SEN Senegal
SGP Singapore
SGS South Georgia and the South Sandwich Islands
SHN Saint Helena, Ascension and Tristan da Cunha
SJM Svalbard and Jan Mayen
SLB Solomon Islands
SLE Sierra Leone
SLV El Salvador
SMR San Marino
SOM Somalia
SPM Saint Pierre and Miquelon
SRB Serbia
STP Sao Tome and Principe
SUR Suriname
SVK Slovakia
SVN Slovenia
SWE Sweden
SWZ Swaziland
SYC Seychelles
SYR Syrian Arab Republic
TCA Turks and Caicos Islands
TCD Chad
TGO Togo
THA Thailand
TJK Tajikistan
TKL Tokelau
TKM Turkmenistan
TLS Timor-Leste

TON Tonga
TTO Trinidad and Tobago
TUN Tunisia
TUR Turkey
TUV Tuvalu
TWN Taiwan, Province of China
TZA Tanzania, United Republic of
UGA Uganda
UKR Ukraine
UMI United States Minor Outlying Islands
URY Uruguay
USA United States
UZB Uzbekistan
VAT Holy See (Vatican City State)
VCT Saint Vincent and the Grenadines
VEN Venezuela, Bolivarian Republic of
VGB Virgin Islands, British
VIR Virgin Islands, U.S.
VNM Viet Nam
VUT Vanuatu
WLF Wallis and Futuna
WSM Samoa
YEM Yemen
ZAF South Africa
ZMB Zambia
ZWE Zimbabwe

Part 4. Additional Supplementary Results

Supplementary Figure 8. Map of contraceptive prevalence, 2019

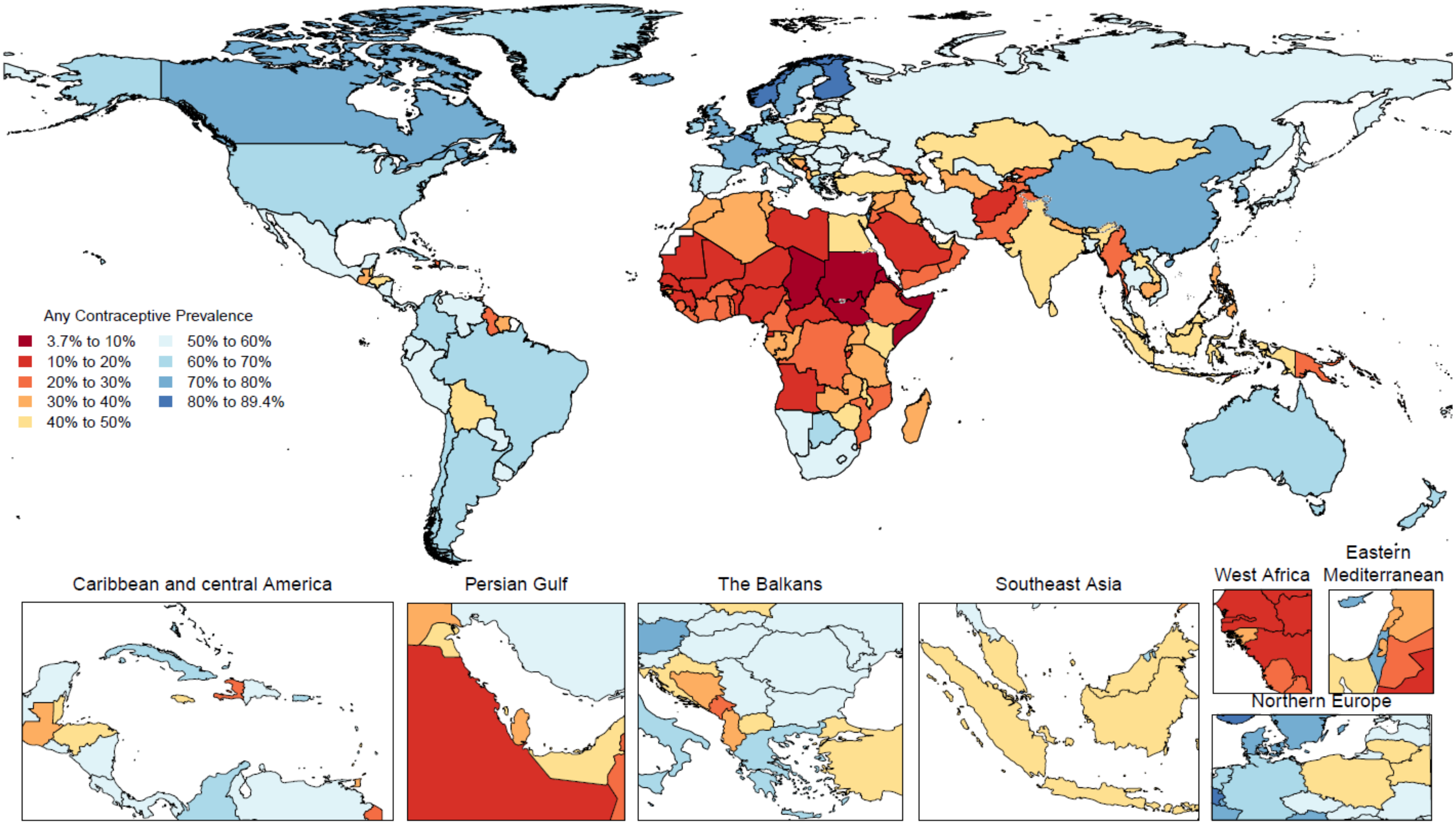

Supplementary Figure 9. Map of the absolute change in contraceptive prevalence, 1970-2019

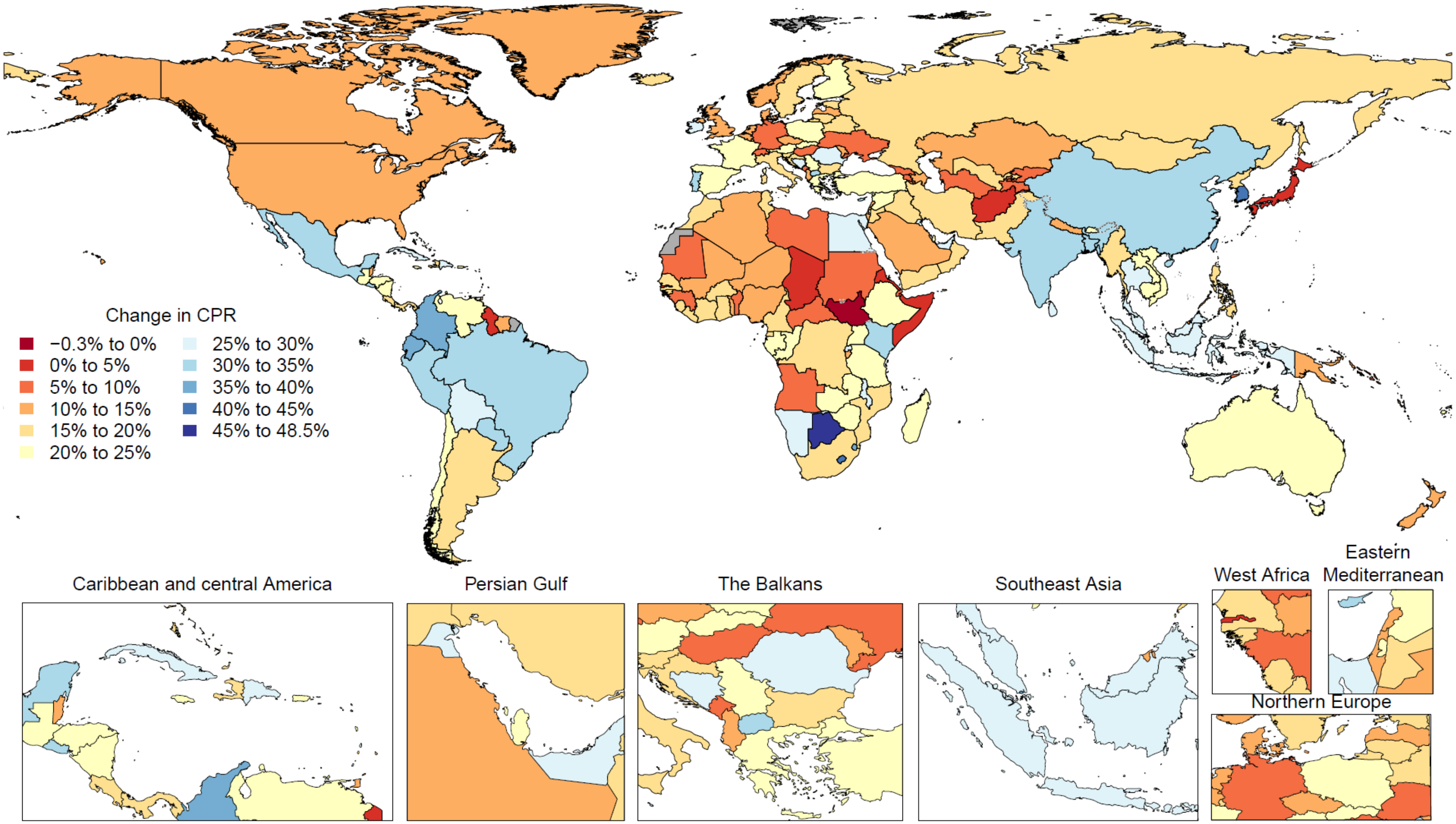

Supplementary Figure 10. Map of the absolute change in modern contraceptive prevalence, 1970-2019

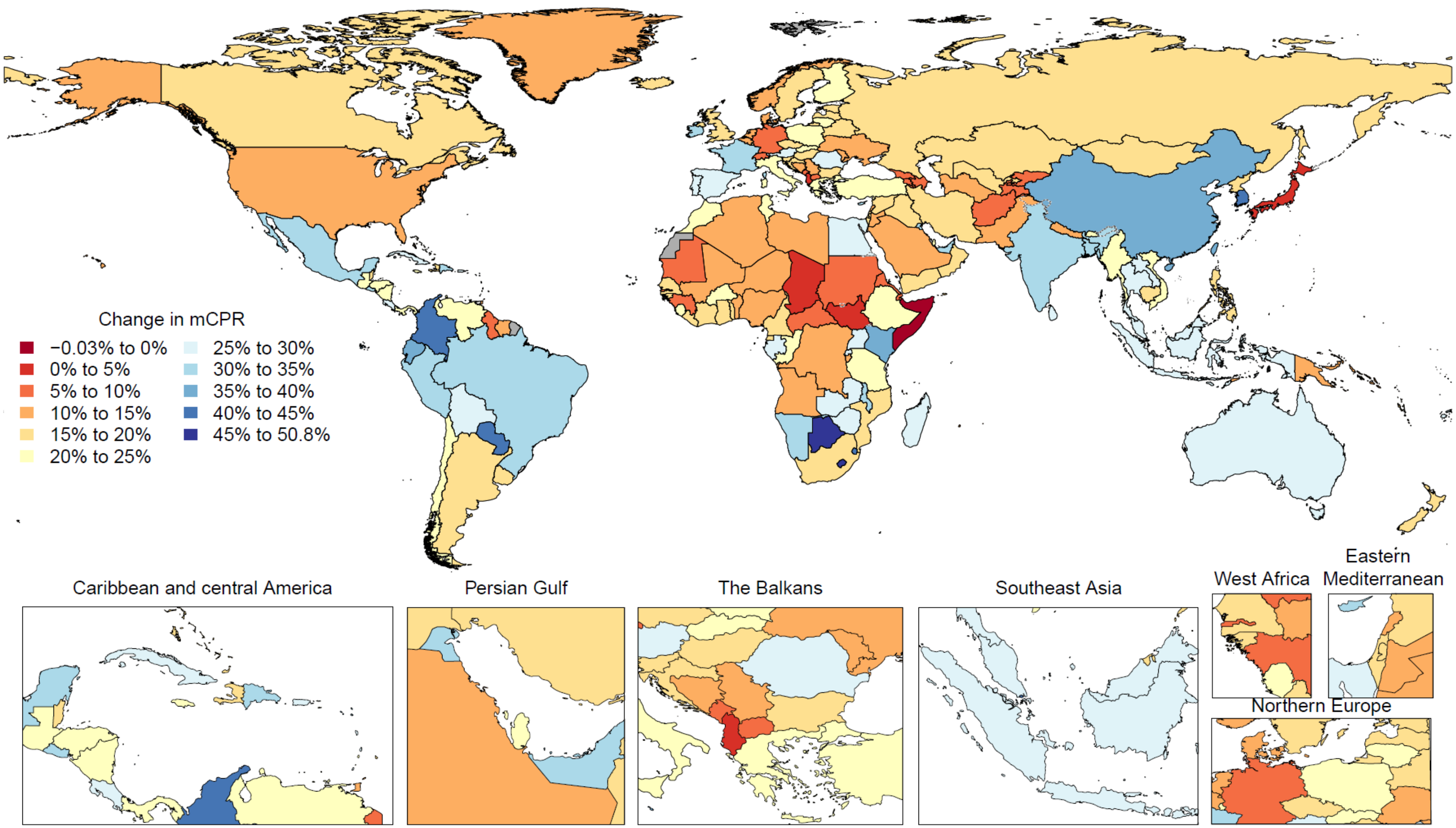

Supplementary Figure 11. Map of the absolute change in demand satisfied with modern methods, 1970-2019

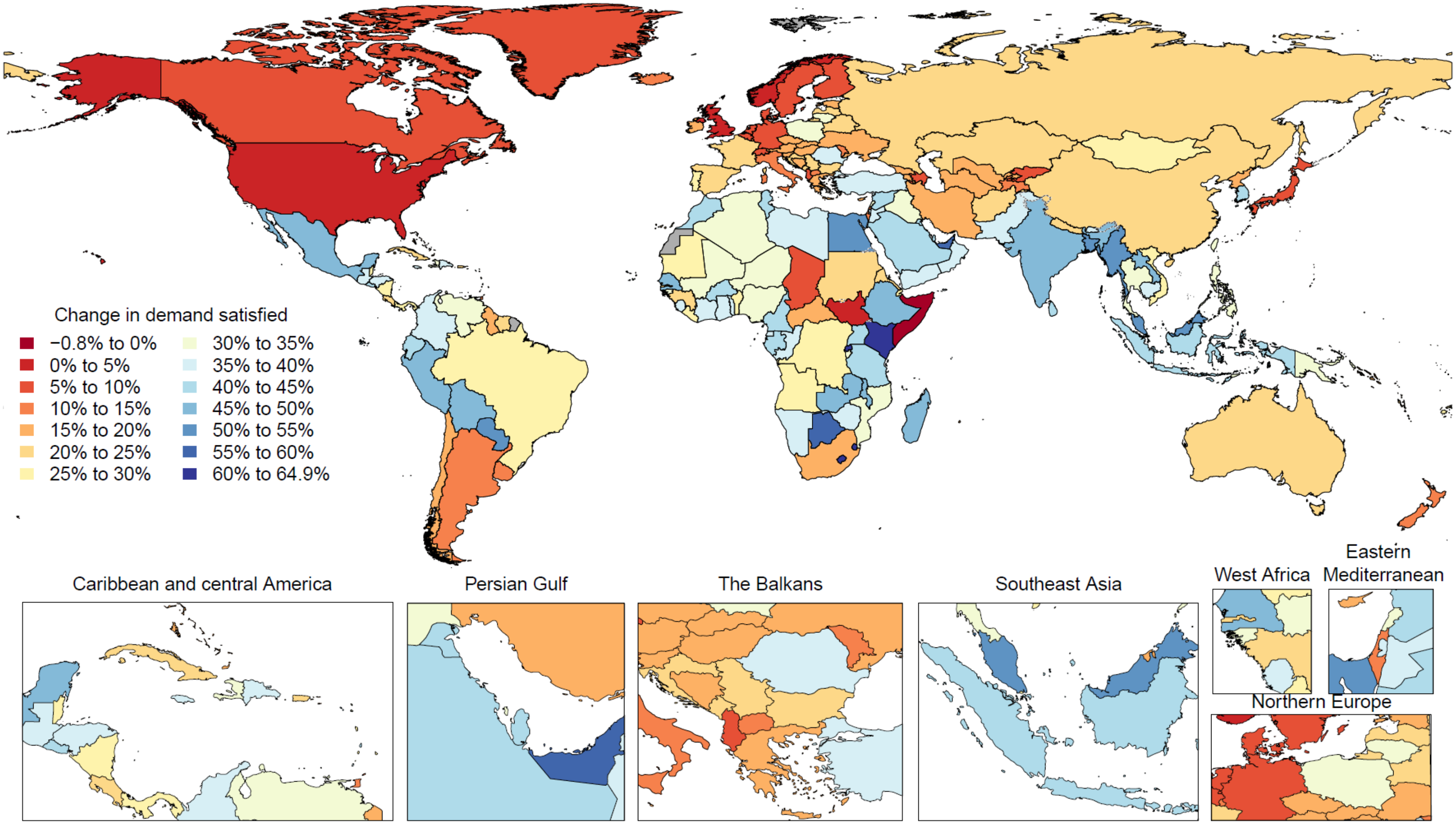

Supplementary Figure 12. Map of the absolute change in unmet need for any method, 1970-2019

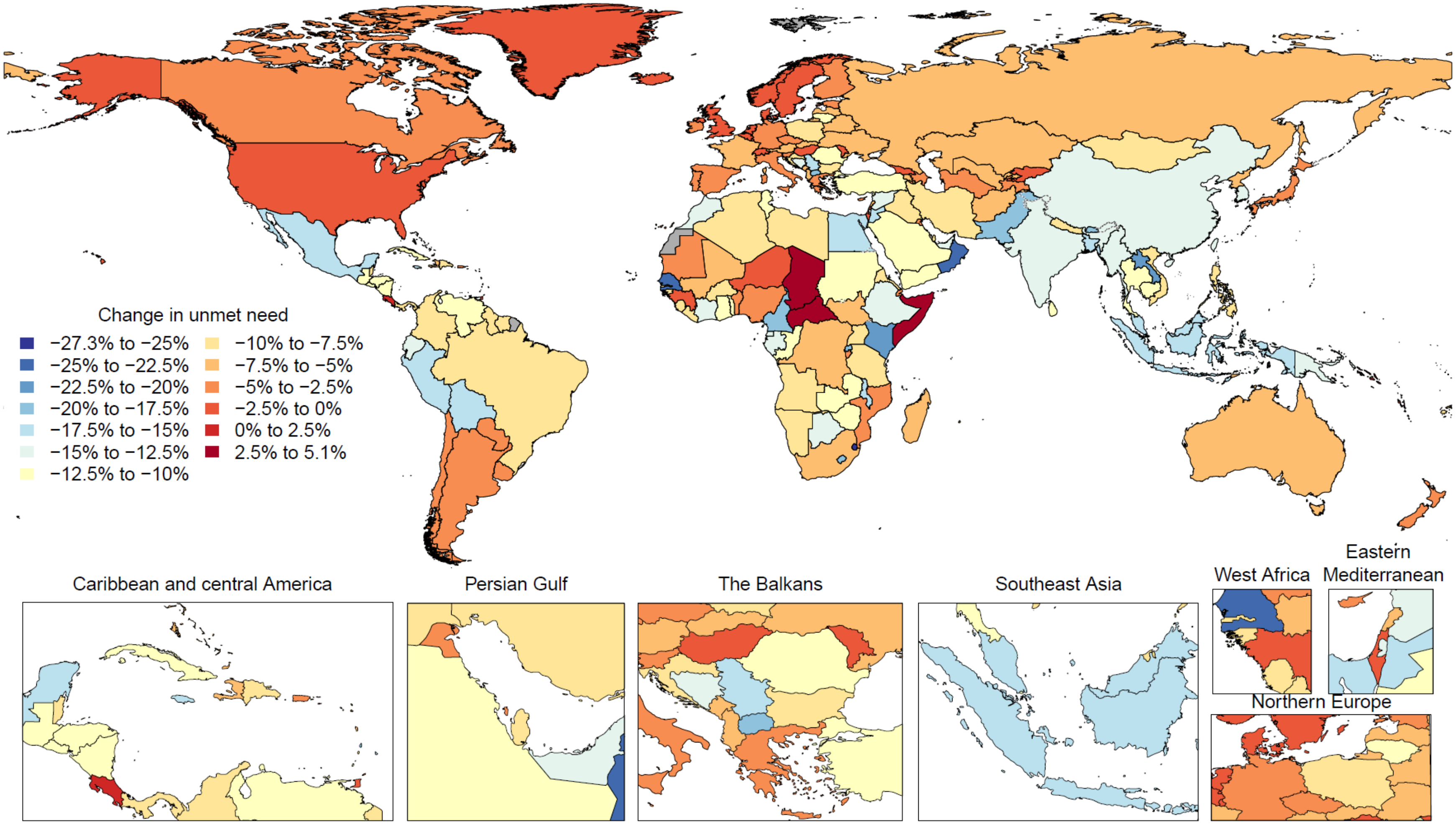

Supplementary Figure 13. Demand satisfied by five-year age cohort and super-region, 1970-2019

Notes: Cohorts are defined as women who were aged 15-19 in a given start year and followed by age over time.

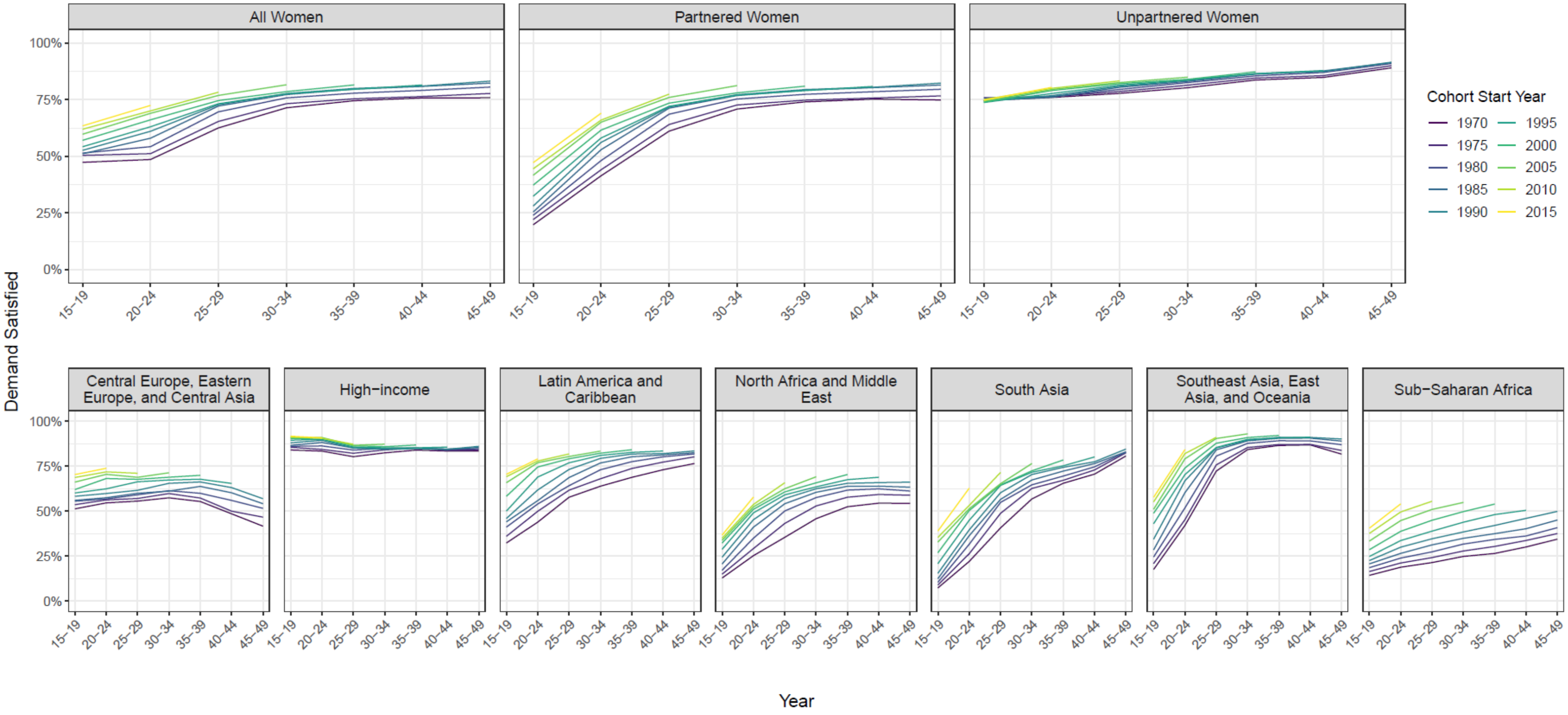

Supplementary Figure 14. Input estimates for the expected change in modern contraceptive prevalence given SDI between 1970 and 2019
Notes: The grey points by age group represent every location-year estimate between 1970 and 2019 which were used to inform the expected values of mCPR and demand satisfied.

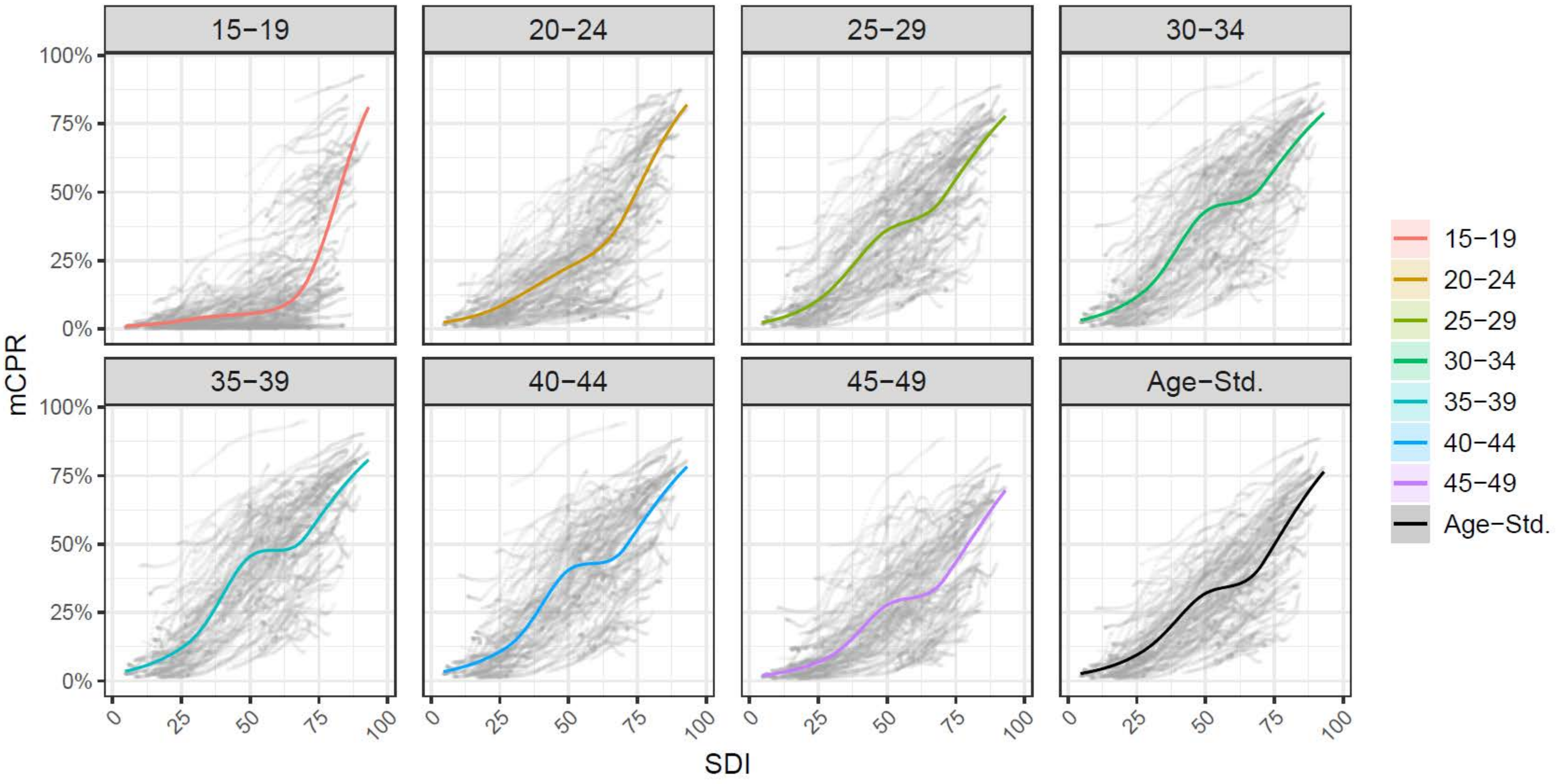

Supplementary Figure 15. Input estimates for the expected change in demand satisfied with modern methods given SDI between 1970 and 2019

Notes: The grey points by age group represent every location-year estimate between 1970 and 2019 which were used to inform the expected values of mCPR and demand satisfied.

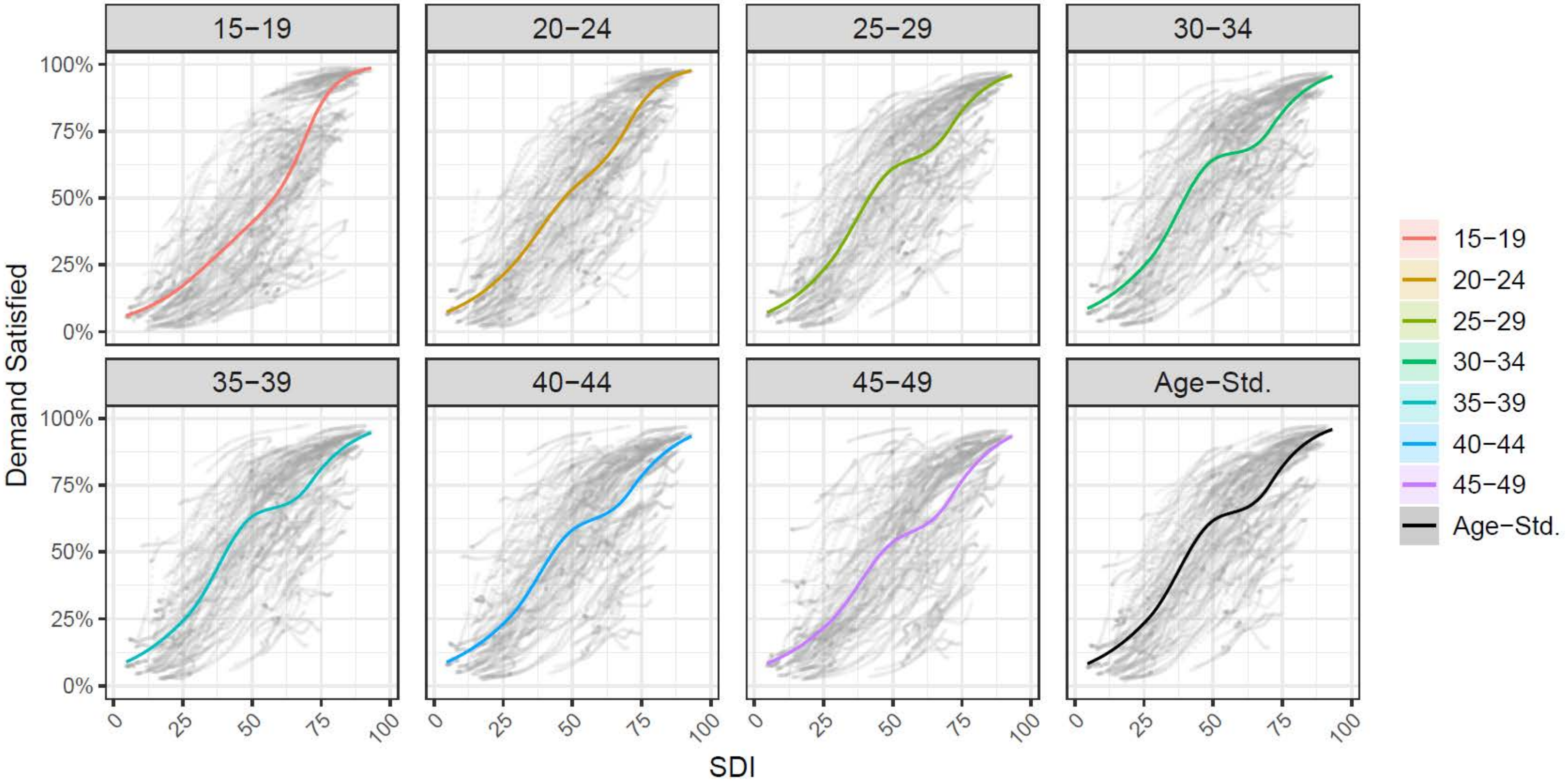

Location	Female sterilisation	Male sterilisation	Intrauterine device	Injections	Implants	Pill	Condom	Other modern method	Lactational amenorrhoea method	Rhythm	Withdrawal	Other traditional method
**Global**	**20·9 (18·8 – 23·2)**	**6·1 (5·3 – 7·1)**	**24·2 (21·9 – 26·4)**	**1·0 (0·9 – 1·1)**	**0·1 (0·1 – 0·1)**	**20·2 (19·3 – 21·2)**	**7·2 (6·7 – 7·7)**	**3·3 (3·1 – 3·5)**	**2·0 (1·8 – 2·2)**	**6·8 (6·3 – 7·2)**	**5·5 (5·1 – 5·9)**	**2·7 (2·5 – 3·0)**
Low SDI	9·5 (8·2 – 11·0)	6·0 (4·5 – 7·8)	3·2 (2·8 – 3·6)	7·9 (6·9 – 9·0)	0·6 (0·6 – 0·7)	15·7 (14·2 – 17·4)	4·1 (3·7 – 4·6)	4·8 (4·5 – 5·1)	13·6 (12·6 – 14·8)	23·3 (21·6 – 25·1)	5·9 (5·2 – 6·7)	5·4 (4·9 – 5·9)
Low-middle SDI	26·4 (22·5 – 30·8)	9·0 (7·4 – 10·8)	9·5 (8·2 – 10·9)	1·0 (0·8 – 1·2)	0·0 (0·0 – 0·0)	10·8 (9·3 – 12·6)	7·7 (6·4 – 9·6)	2·5 (2·3 – 2·7)	4·2 (3·6 – 5·0)	15·1 (12·6 – 17·8)	11·3 (9·9 – 13·0)	2·4 (2·0 – 2·9)
Middle SDI	21·6 (19·5 – 23·8)	1·3 (1·1 – 1·6)	18·0 (16·4 – 19·9)	2·7 (2·3 – 3·4)	0·1 (0·1 – 0·1)	27·9 (25·5 – 30·6)	2·6 (2·3 – 3·0)	2·8 (2·6 – 3·0)	3·8 (3·4 – 4·2)	10·1 (9·1 – 11·1)	6·4 (5·7 – 7·3)	2·7 (2·3 – 3·1)
High-middle SDI	23·4 (19·2 – 28·0)	5·7 (4·1 – 7·5)	38·0 (33·4 – 42·4)	0·1 (0·1 – 0·2)	0·1 (0·1 – 0·1)	10·0 (9·0 – 11·0)	5·7 (5·1 – 6·4)	2·5 (2·3 – 2·8)	1·7 (1·4 – 2·2)	4·9 (4·3 – 5·5)	5·0 (4·5 – 5·6)	2·9 (2·5 – 3·4)
High SDI	15·5 (14·1 – 16·9)	7·6 (6·7 – 8·5)	11·5 (10·6 – 12·3)	1·2 (1·0 – 1·5)	0·2 (0·2 – 0·2)	36·9 (35·1 – 38·6)	11·1 (10·2 – 12·1)	4·9 (4·6 – 5·3)	0·4 (0·3 – 0·4)	4·6 (4·1 – 5·0)	3·7 (3·4 – 4·1)	2·5 (2·2 – 2·9)
**Central Europe, eastern Europe, and central Asia**	**2·1 (1·9 – 2·4)**	**0·4 (0·4 – 0·5)**	**30·1 (27·3 – 33·1)**	**0·2 (0·2 – 0·3)**	**0·2 (0·1 – 0·2)**	**13·0 (11·6 – 14·5)**	**15·7 (14·1 – 17·5)**	**3·3 (3·0 – 3·6)**	**1·8 (1·6 – 2·1)**	**14·2 (12·5 – 15·9)**	**14·3 (12·9 – 15·7)**	**4·7 (3·8 – 5·8)**
Central Asia	2·3 (1·9 – 2·8)	0·3 (0·3 – 0·4)	57·7 (55·0 – 60·4)	0·6 (0·4 – 0·8)	0·1 (0·1 – 0·1)	4·6 (3·9 – 5·4)	5·5 (4·7 – 6·3)	2·1 (1·9 – 2·3)	4·3 (3·7 – 5·1)	7·4 (6·4 – 8·5)	12·6 (11·0 – 14·3)	2·5 (2·1 – 3·1)
Armenia	1·6 (1·0 – 2·5)	0·2 (0·2 – 0·3)	17·3 (12·8 – 23·1)	0·1 (0·1 – 0·2)	0·1 (0·1 – 0·1)	2·2 (1·4 – 3·3)	11·3 (7·9 – 15·6)	1·7 (1·4 – 2·1)	3·1 (2·0 – 4·6)	9·4 (6·7 – 12·6)	50·3 (42·7 – 57·3)	2·7 (1·9 – 3·8)
Azerbaijan	1·1 (0·7 – 1·9)	0·3 (0·2 – 0·4)	14·4 (10·3 – 19·3)	0·2 (0·1 – 0·3)	0·1 (0·0 – 0·1)	2·6 (1·6 – 3·9)	2·9 (1·9 – 4·1)	1·9 (1·5 – 2·2)	3·5 (2·4 – 5·0)	9·1 (6·4 – 12·5)	61·0 (54·0 – 67·1)	3·1 (2·1 – 4·4)
Georgia	4·9 (3·1 – 7·7)	0·6 (0·4 – 0·8)	23·7 (17·7 – 30·3)	0·2 (0·1 – 0·4)	0·2 (0·1 – 0·3)	7·9 (5·4 – 11·1)	15·3 (11·3 – 20·6)	3·9 (3·3 – 4·7)	5·5 (3·8 – 7·6)	28·0 (21·6 – 34·6)	9·2 (6·3 – 13·0)	0·6 (0·4 – 0·8)
Kazakhstan	1·9 (1·2 – 2·9)	0·3 (0·2 – 0·4)	64·3 (58·7 – 69·0)	0·2 (0·1 – 0·3)	0·1 (0·1 – 0·2)	6·2 (4·4 – 8·4)	6·0 (4·5 – 8·0)	2·3 (1·9 – 2·7)	4·1 (2·9 – 6·0)	6·4 (4·8 – 8·4)	3·2 (2·3 – 4·6)	5·0 (3·7 – 6·6)
Kyrgyzstan	2·8 (1·8 – 4·5)	0·4 (0·3 – 0·5)	62·9 (56·6 – 68·9)	1·1 (0·7 – 1·7)	0·2 (0·1 – 0·3)	5·9 (4·1 – 8·4)	12·7 (9·3 – 16·5)	1·2 (1·0 – 1·5)	3·4 (2·3 – 5·0)	2·9 (2·0 – 3·9)	4·9 (3·4 – 7·1)	1·6 (1·1 – 2·2)
Mongolia	4·5 (2·9 – 6·9)	0·5 (0·3 – 0·6)	49·3 (42·5 – 56·6)	1·2 (0·7 – 2·0)	0·0 (0·0 – 0·1)	12·3 (8·6 – 16·9)	3·6 (2·6 – 5·0)	2·7 (2·3 – 3·2)	8·0 (5·6 – 10·7)	15·9 (12·0 – 20·2)	0·6 (0·4 – 0·8)	1·3 (0·9 – 1·7)
Tajikistan	1·3 (0·8 – 2·0)	0·8 (0·5 – 1·2)	71·8 (65·2 – 77·4)	1·9 (1·1 – 3·2)	0·1 (0·1 – 0·1)	3·7 (2·2 – 5·8)	2·1 (1·4 – 3·3)	3·1 (2·4 – 3·8)	4·8 (3·0 – 7·4)	2·6 (1·8 – 3·8)	7·2 (4·8 – 10·3)	0·7 (0·5 – 1·0)
Turkmenistan	2·2 (1·4 – 3·4)	0·4 (0·3 – 0·5)	73·7 (68·7 – 78·1)	0·4 (0·3 – 0·7)	0·1 (0·1 – 0·1)	2·0 (1·3 – 3·1)	1·6 (1·1 – 2·3)	1·9 (1·6 – 2·3)	9·4 (6·4 – 13·1)	2·2 (1·5 – 3·1)	4·8 (3·3 – 6·7)	1·3 (1·0 – 1·9)
Uzbekistan	2·3 (1·4 – 3·7)	0·3 (0·2 – 0·4)	79·2 (75·2 – 82·5)	0·9 (0·6 – 1·6)	0·0 (0·0 – 0·0)	2·8 (1·8 – 4·1)	1·4 (1·0 – 2·0)	1·5 (1·3 – 1·8)	3·9 (2·6 – 5·7)	3·0 (2·1 – 4·2)	3·5 (2·5 – 4·9)	1·0 (0·7 – 1·4)
Central Europe	4·4 (3·8 – 5·2)	0·6 (0·5 – 0·7)	9·5 (8·4 – 10·6)	0·2 (0·1 – 0·2)	0·2 (0·1 – 0·2)	21·3 (19·3 – 23·2)	13·9 (12·6 – 15·4)	5·6 (5·0 – 6·3)	2·5 (2·1 – 2·9)	16·4 (14·7 – 18·3)	23·2 (21·3 – 25·3)	2·3 (2·0 – 2·6)
Albania	2·4 (1·5 – 3·8)	0·4 (0·3 – 0·5)	0·8 (0·6 – 1·3)	0·2 (0·1 – 0·3)	0·0 (0·0 – 0·1)	3·8 (2·4 – 5·9)	3·6 (2·5 – 5·2)	1·3 (1·1 – 1·6)	9·8 (6·7 – 13·5)	5·2 (3·7 – 7·1)	71·5 (65·7 – 76·6)	0·9 (0·7 – 1·3)
Bosnia and Herzegovina	0·5 (0·4 – 0·8)	0·5 (0·3 – 0·6)	9·7 (6·8 – 13·7)	0·1 (0·0 – 0·1)	0·1 (0·1 – 0·2)	6·9 (4·7 – 9·9)	3·9 (2·8 – 5·3)	2·2 (1·8 – 2·8)	3·5 (2·3 – 5·2)	14·3 (10·3 – 19·4)	56·6 (48·8 – 63·0)	1·7 (1·2 – 2·3)
Bulgaria	1·8 (1·2 – 2·7)	0·4 (0·3 – 0·6)	14·0 (10·2 – 18·8)	0·1 (0·1 – 0·2)	0·1 (0·1 – 0·2)	12·1 (9·0 – 15·9)	16·8 (12·9 – 21·2)	2·3 (1·9 – 2·7)	2·6 (1·7 – 3·8)	10·2 (7·6 – 13·2)	38·1 (31·9 – 44·4)	1·6 (1·2 – 2·1)
Croatia	2·7 (1·8 – 3·9)	0·7 (0·5 – 0·9)	9·7 (6·9 – 13·0)	0·3 (0·2 – 0·4)	0·3 (0·2 – 0·4)	16·6 (12·5 – 21·5)	15·8 (12·3 – 19·9)	3·9 (3·2 – 4·6)	2·3 (1·7 – 3·2)	11·5 (8·6 – 15·1)	34·0 (27·7 – 40·8)	2·4 (1·7 – 3·2)
Czechia	8·6 (5·9 – 12·0)	0·5 (0·3 – 0·6)	18·1 (14·1 – 22·8)	0·4 (0·3 – 0·7)	0·2 (0·1 – 0·3)	24·3 (19·4 – 29·5)	18·2 (14·3 – 22·5)	1·7 (1·5 – 2·0)	1·4 (1·0 – 2·1)	3·6 (2·6 – 4·8)	18·3 (14·1 – 22·9)	4·7 (3·5 – 6·1)
Hungary	8·4 (6·2 – 11·2)	0·4 (0·3 – 0·5)	12·9 (10·0 – 16·4)	0·1 (0·1 – 0·2)	0·2 (0·1 – 0·3)	40·0 (35·0 – 45·2)	6·2 (4·8 – 8·1)	4·5 (3·9 – 5·3)	1·3 (1·0 – 1·8)	6·3 (4·9 – 7·7)	15·7 (12·5 – 19·5)	3·9 (3·1 – 4·8)
Montenegro	0·9 (0·6 – 1·4)	0·8 (0·6 – 1·0)	14·4 (10·4 – 19·5)	0·4 (0·2 – 0·6)	0·6 (0·3 – 1·1)	12·3 (8·9 – 16·3)	17·3 (13·3 – 21·9)	6·4 (5·3 – 7·6)	2·0 (1·4 – 2·7)	13·2 (9·8 – 17·3)	27·0 (20·7 – 34·0)	4·7 (3·1 – 6·9)
North Macedonia	3·6 (2·3 – 5·5)	1·3 (1·0 – 1·9)	3·7 (2·6 – 5·6)	0·2 (0·1 – 0·3)	0·3 (0·2 – 0·5)	10·6 (7·8 – 14·4)	20·9 (16·1 – 26·6)	11·8 (10·0 – 14·1)	5·4 (3·7 – 7·6)	8·6 (6·5 – 11·6)	28·5 (22·1 – 35·4)	5·0 (3·7 – 6·9)
Poland	2·6 (1·7 – 3·9)	0·6 (0·4 – 0·8)	2·1 (1·5 – 2·9)	0·1 (0·1 – 0·2)	0·2 (0·1 – 0·3)	27·3 (21·8 – 33·3)	19·3 (15·0 – 24·4)	12·6 (10·6 – 15·0)	2·8 (1·9 – 4·1)	22·8 (17·9 – 28·1)	8·7 (6·4 – 11·4)	0·9 (0·7 – 1·2)
Romania	3·6 (2·3 – 5·7)	1·0 (0·7 – 1·4)	7·5 (5·3 – 10·3)	0·1 (0·0 – 0·1)	0·1 (0·0 – 0·1)	9·2 (6·6 – 12·3)	5·8 (4·3 – 7·7)	5·2 (4·2 – 6·3)	3·2 (2·3 – 4·5)	30·2 (24·7 – 36·0)	32·1 (25·7 – 38·9)	2·2 (1·5 – 2·9)
Serbia	0·7 (0·5 – 1·0)	0·4 (0·3 – 0·5)	6·1 (4·3 – 8·8)	0·1 (0·1 – 0·2)	0·2 (0·1 – 0·3)	9·4 (7·1 – 12·2)	22·4 (18·0 – 27·7)	2·9 (2·4 – 3·5)	3·2 (2·3 – 4·4)	24·9 (19·9 – 30·3)	28·2 (22·6 – 34·4)	1·4 (1·0 – 1·8)
Slovakia	7·9 (5·6 – 10·6)	0·4 (0·3 – 0·6)	16·9 (12·8 – 21·2)	0·2 (0·2 – 0·3)	0·2 (0·1 – 0·3)	25·5 (20·7 – 30·7)	17·6 (14·2 – 21·4)	2·3 (1·9 – 2·7)	1·1 (0·8 – 1·5)	6·7 (5·0 – 8·8)	19·9 (15·4 – 24·6)	1·4 (1·0 – 1·8)
Slovenia	2·7 (1·8 – 3·9)	0·6 (0·5 – 0·8)	9·3 (6·7 – 12·5)	0·5 (0·3 – 0·7)	0·3 (0·2 – 0·5)	17·3 (13·6 – 21·5)	23·0 (18·7 – 27·9)	3·2 (2·6 – 3·8)	1·2 (0·9 – 1·6)	9·1 (6·9 – 12·0)	31·0 (25·3 – 36·8)	1·8 (1·4 – 2·4)
Eastern Europe	1·1 (0·9 – 1·4)	0·3 (0·3 – 0·4)	34·7 (30·5 – 39·2)	0·2 (0·1 – 0·3)	0·2 (0·1 – 0·2)	10·8 (8·9 – 13·0)	18·0 (15·5 – 20·7)	2·5 (2·2 – 2·8)	1·2 (1·0 – 1·6)	14·2 (11·6 – 16·9)	10·8 (9·0 – 12·7)	6·0 (4·6 – 7·7)
Belarus	5·0 (3·3 – 7·4)	0·4 (0·3 – 0·5)	26·5 (20·9 – 32·5)	0·1 (0·1 – 0·2)	0·1 (0·1 – 0·1)	17·3 (13·4 – 22·4)	22·0 (17·8 – 26·8)	2·2 (1·9 – 2·6)	3·4 (2·3 – 4·6)	10·0 (7·5 – 12·9)	11·8 (8·6 – 15·5)	1·2 (0·9 – 1·6)
Estonia	1·2 (0·8 – 1·8)	0·4 (0·3 – 0·5)	32·9 (27·3 – 39·1)	0·2 (0·2 – 0·3)	0·1 (0·1 – 0·2)	34·3 (28·2 – 40·7)	12·9 (10·1 – 16·3)	3·2 (2·7 – 3·8)	1·2 (0·9 – 1·7)	9·6 (7·1 – 12·4)	2·5 (1·9 – 3·4)	1·5 (1·1 – 1·9)
Latvia	1·2 (0·8 – 1·7)	0·4 (0·3 – 0·5)	36·5 (30·1 – 42·4)	0·2 (0·2 – 0·3)	0·1 (0·1 – 0·2)	12·0 (9·0 – 15·7)	18·9 (15·1 – 23·0)	2·3 (2·0 – 2·7)	1·2 (0·9 – 1·7)	12·4 (9·5 – 15·9)	11·1 (8·4 – 14·4)	3·6 (2·6 – 4·7)
Lithuania	1·2 (0·8 – 1·7)	0·4 (0·3 – 0·5)	13·6 (10·2 – 17·8)	0·1 (0·1 – 0·2)	0·1 (0·1 – 0·1)	28·9 (23·0 – 36·1)	16·6 (13·0 – 21·0)	3·7 (3·1 – 4·4)	1·8 (1·3 – 2·6)	20·4 (16·0 – 25·9)	9·0 (6·7 – 12·0)	4·2 (3·1 – 5·6)
Moldova	4·2 (2·9 – 6·1)	0·3 (0·2 – 0·4)	45·2 (39·1 – 51·1)	0·2 (0·1 – 0·2)	0·1 (0·1 – 0·1)	4·3 (3·1 – 5·9)	8·1 (6·1 – 10·5)	1·5 (1·3 – 1·8)	2·2 (1·5 – 3·2)	7·3 (5·4 – 9·6)	25·2 (20·2 – 31·0)	1·2 (0·9 – 1·6)
Russia	0·4 (0·3 – 0·6)	0·4 (0·3 – 0·5)	37·8 (31·7 – 43·9)	0·2 (0·1 – 0·3)	0·2 (0·2 – 0·3)	10·9 (8·2 – 14·1)	15·1 (12·0 – 18·9)	2·4 (2·1 – 2·8)	1·3 (0·9 – 1·7)	15·5 (11·8 – 19·4)	7·6 (5·6 – 9·8)	8·3 (6·2 – 10·8)
Ukraine	2·0 (1·4 – 2·9)	0·3 (0·3 – 0·4)	29·1 (23·8 – 35·0)	0·2 (0·1 – 0·3)	0·1 (0·1 – 0·2)	8·7 (6·4 – 11·5)	24·7 (20·3 – 29·5)	2·6 (2·2 – 3·0)	0·7 (0·6 – 1·0)	12·3 (9·4 – 15·7)	17·1 (12·9 – 21·6)	2·1 (1·6 – 2·8)
**High income**	**14·4 (13·2 – 15·8)**	**7·1 (6·4 – 8·0)**	**11·3 (10·6 – 12·1)**	**1·2 (1·0 – 1·4)**	**0·2 (0·2 – 0·2)**	**38·6 (37·0 – 40·1)**	**11·0 (10·1 – 11·9)**	**4·9 (4·6 – 5·2)**	**0·4 (0·4 – 0·4)**	**4·4 (4·0 – 4·8)**	**3·6 (3·3 – 4·0)**	**2·9 (2·6 – 3·2)**
Australasia	9·0 (6·5 – 12·1)	10·6 (8·2 – 13·6)	6·3 (5·2 – 7·8)	0·5 (0·4 – 0·7)	0·3 (0·2 – 0·4)	42·4 (37·6 – 46·9)	12·6 (10·2 – 15·6)	2·9 (2·5 – 3·4)	0·7 (0·6 – 0·9)	7·6 (6·1 – 9·3)	1·9 (1·5 – 2·4)	5·2 (3·9 – 7·2)
Australia	9·8 (6·8 – 13·4)	10·5 (7·5 – 14·0)	4·5 (3·3 – 6·2)	0·3 (0·2 – 0·5)	0·2 (0·2 – 0·3)	43·0 (37·2 – 48·6)	12·6 (9·4 – 16·3)	3·0 (2·5 – 3·6)	0·8 (0·6 – 1·0)	8·1 (6·2 – 10·2)	1·2 (0·9 – 1·7)	5·9 (4·3 – 8·4)
New Zealand	6·1 (4·2 – 8·5)	10·8 (7·8 – 14·4)	13·2 (10·3 – 16·5)	1·3 (0·9 – 1·8)	0·4 (0·3 – 0·6)	40·2 (35·0 – 45·4)	12·6 (9·8 – 15·8)	2·4 (2·1 – 2·9)	0·5 (0·4 – 0·7)	5·7 (4·4 – 7·3)	4·4 (3·2 – 6·1)	2·3 (1·8 – 3·0)
High-income Asia Pacific	16·1 (12·8 – 19·5)	7·7 (5·7 – 10·1)	10·5 (8·3 – 13·2)	2·3 (1·6 – 3·2)	0·2 (0·2 – 0·3)	22·3 (18·4 – 26·9)	20·6 (16·8 – 24·5)	2·7 (2·4 – 3·1)	0·5 (0·4 – 0·6)	6·6 (5·2 – 8·3)	3·8 (2·8 – 4·9)	6·7 (5·4 – 8·3)
Brunei	13·2 (10·4 – 16·6)	5·9 (4·2 – 8·1)	8·4 (6·5 – 10·4)	0·3 (0·2 – 0·4)	0·1 (0·1 – 0·2)	34·8 (28·8 – 41·4)	8·2 (6·0 – 11·1)	5·1 (4·2 – 6·2)	1·2 (0·9 – 1·6)	9·7 (7·5 – 12·3)	3·1 (2·3 – 4·3)	9·8 (7·6 – 12·4)
Japan	16·5 (12·9 – 20·5)	7·6 (5·3 – 10·2)	10·0 (7·6 – 13·0)	2·6 (1·7 – 3·7)	0·2 (0·2 – 0·3)	22·8 (18·3 – 27·9)	22·6 (18·3 – 27·0)	2·3 (2·0 – 2·7)	0·3 (0·2 – 0·4)	5·8 (4·3 – 7·7)	3·7 (2·6 – 5·0)	5·6 (4·2 – 7·3)
South Korea	12·6 (9·2 – 16·6)	9·3 (6·7 – 12·3)	15·0 (11·8 – 18·7)	0·3 (0·2 – 0·4)	0·1 (0·0 – 0·1)	16·9 (13·2 – 21·1)	6·1 (4·6 – 8·0)	5·8 (4·9 – 6·9)	1·7 (1·2 – 2·2)	12·7 (10·1 – 15·9)	4·7 (3·5 – 6·3)	14·8 (11·5 – 18·8)
Singapore	15·0 (12·2 – 18·3)	5·6 (4·0 – 7·5)	4·4 (3·5 – 5·6)	0·2 (0·2 – 0·3)	0·1 (0·1 – 0·2)	37·5 (31·9 – 43·3)	12·8 (10·5 – 15·8)	4·2 (3·5 – 5·0)	1·0 (0·7 – 1·3)	7·3 (5·6 – 9·3)	2·7 (1·9 – 3·6)	9·2 (7·4 – 11·3)
High-income North America	20·5 (17·7 – 23·5)	11·0 (9·0 – 13·3)	8·6 (7·4 – 10·0)	1·1 (0·8 – 1·5)	0·1 (0·1 – 0·2)	34·4 (31·0 – 37·7)	8·2 (6·9 – 9·7)	8·6 (7·6 – 9·6)	0·3 (0·2 – 0·3)	3·7 (3·0 – 4·6)	2·0 (1·5 – 2·5)	1·6 (1·4 – 2·0)
Canada	32·7 (28·0 – 37·6)	12·8 (9·4 – 16·4)	7·4 (5·5 – 9·6)	1·0 (0·7 – 1·5)	0·1 (0·1 – 0·1)	30·9 (26·3 – 35·3)	6·4 (4·8 – 8·2)	3·7 (3·1 – 4·3)	0·3 (0·2 – 0·3)	2·8 (2·0 – 3·7)	1·2 (0·9 – 1·6)	0·8 (0·6 – 1·0)
Greenland	25·9 (21·3 – 30·8)	12·6 (9·5 – 16·3)	6·3 (4·8 – 8·2)	0·7 (0·5 – 1·0)	0·1 (0·1 – 0·1)	31·5 (26·7 – 36·7)	6·5 (4·7 – 8·7)	7·9 (6·7 – 9·3)	0·4 (0·3 – 0·6)	4·2 (3·1 – 5·5)	2·2 (1·6 – 2·9)	1·7 (1·3 – 2·1)
USA	19·0 (16·0 – 22·4)	10·7 (8·7 – 13·3)	8·8 (7·4 – 10·3)	1·1 (0·8 – 1·6)	0·1 (0·1 – 0·2)	34·8 (31·0 – 38·4)	8·4 (7·0 – 10·1)	9·1 (8·1 – 10·3)	0·3 (0·2 – 0·3)	3·8 (3·1 – 4·8)	2·1 (1·6 – 2·7)	1·8 (1·4 – 2·1)
Southern Latin America	13·3 (10·7 – 16·2)	0·4 (0·3 – 0·5)	21·2 (18·7 – 24·1)	1·0 (0·7 – 1·4)	0·5 (0·3 – 0·7)	43·7 (39·3 – 48·0)	9·6 (7·7 – 12·1)	4·0 (3·5 – 4·6)	1·0 (0·8 – 1·1)	3·1 (2·5 – 3·7)	0·9 (0·7 – 1·2)	1·4 (1·2 – 1·8)
Argentina	14·2 (10·5 – 18·4)	0·4 (0·3 – 0·5)	13·0 (10·3 – 16·4)	1·4 (1·0 – 2·0)	0·6 (0·4 – 0·9)	47·9 (41·8 – 54·2)	12·6 (9·7 – 16·2)	4·2 (3·4 – 5·1)	0·8 (0·6 – 1·1)	2·3 (1·7 – 3·1)	1·1 (0·7 – 1·5)	1·6 (1·2 – 2·1)
Chile	10·4 (7·4 – 14·2)	0·4 (0·3 – 0·5)	41·3 (35·5 – 47·0)	0·2 (0·1 – 0·3)	0·2 (0·2 – 0·4)	33·7 (28·3 – 40·0)	2·6 (1·9 – 3·4)	3·5 (2·9 – 4·3)	1·2 (0·9 – 1·7)	4·7 (3·5 – 6·4)	0·5 (0·4 – 0·8)	1·0 (0·7 – 1·4)
Uruguay	14·6 (10·3 – 19·2)	0·5 (0·4 – 0·7)	25·0 (19·7 – 30·7)	0·6 (0·4 – 0·9)	0·3 (0·2 – 0·5)	40·5 (33·8 – 46·4)	6·5 (4·8 – 8·5)	4·4 (3·5 – 5·5)	1·1 (0·8 – 1·6)	3·9 (3·0 – 5·2)	0·8 (0·5 – 1·1)	1·7 (1·3 – 2·3)
Western Europe	10·3 (9·2 – 11·6)	4·9 (4·2 – 5·7)	12·7 (11·6 – 13·8)	0·8 (0·7 – 1·0)	0·2 (0·2 – 0·2)	46·8 (44·9 – 48·8)	9·1 (8·3 – 9·9)	3·6 (3·4 – 3·9)	0·4 (0·3 – 0·4)	3·9 (3·5 – 4·4)	4·9 (4·3 – 5·5)	2·3 (2·0 – 2·6)
Andorra	5·9 (4·3 – 8·1)	1·9 (1·4 – 2·8)	12·2 (9·5 – 15·6)	1·8 (1·2 – 2·8)	0·3 (0·2 – 0·4)	53·6 (48·1 – 58·5)	11·8 (9·1 – 15·0)	3·3 (2·7 – 4·0)	0·3 (0·2 – 0·3)	2·9 (2·1 – 3·8)	4·3 (3·2 – 5·7)	1·7 (1·3 – 2·3)
Austria	2·3 (1·5 – 3·4)	1·0 (0·7 – 1·5)	15·4 (12·0 – 19·4)	0·4 (0·3 – 0·6)	0·3 (0·2 – 0·4)	59·1 (53·5 – 64·5)	8·1 (6·2 – 10·6)	4·6 (3·8 – 5·6)	0·5 (0·3 – 0·6)	5·1 (3·8 – 6·9)	1·3 (0·9 – 1·8)	1·9 (1·4 – 2·5)
Belgium	16·4 (12·4 – 20·8)	8·7 (6·3 – 11·9)	5·9 (4·4 – 8·0)	0·8 (0·5 – 1·3)	0·1 (0·1 – 0·1)	54·6 (49·2 – 59·5)	3·0 (2·2 – 4·1)	2·9 (2·3 – 3·8)	0·4 (0·3 – 0·5)	3·8 (2·8 – 5·0)	2·6 (1·9 – 3·6)	0·7 (0·5 – 1·0)
Cyprus	6·3 (4·4 – 8·7)	2·5 (1·7 – 3·7)	12·4 (9·3 – 15·7)	0·5 (0·3 – 0·9)	0·1 (0·1 – 0·2)	52·8 (46·4 – 58·6)	5·6 (4·1 – 7·4)	5·9 (4·8 – 7·5)	0·7 (0·5 – 1·0)	5·2 (3·9 – 6·8)	4·8 (3·4 – 6·5)	3·2 (2·4 – 4·2)
Denmark	6·9 (5·0 – 9·3)	2·1 (1·4 – 3·0)	12·8 (10·0 – 16·1)	2·0 (1·3 – 3·0)	0·2 (0·2 – 0·3)	50·7 (45·2 – 56·0)	12·7 (10·0 – 15·9)	3·2 (2·6 – 3·9)	0·3 (0·2 – 0·3)	2·9 (2·2 – 3·9)	4·6 (3·3 – 6·1)	1·6 (1·2 – 2·1)
Finland	9·4 (6·9 – 12·3)	1·5 (1·0 – 2·1)	19·8 (16·1 – 23·9)	1·0 (0·6 – 1·5)	0·2 (0·1 – 0·2)	48·2 (43·2 – 53·0)	14·8 (11·8 – 18·3)	2·3 (2·0 – 2·8)	0·3 (0·2 – 0·4)	0·6 (0·5 – 0·8)	1·2 (0·9 – 1·7)	0·7 (0·5 – 0·9)
France	5·4 (3·6 – 7·8)	0·3 (0·2 – 0·4)	15·7 (12·3 – 19·4)	0·4 (0·3 – 0·6)	0·1 (0·1 – 0·2)	47·0 (42·0 – 52·3)	4·5 (3·3 – 5·8)	5·6 (4·8 – 6·5)	0·4 (0·3 – 0·5)	7·6 (5·9 – 9·5)	11·9 (9·3 – 14·8)	1·2 (0·9 – 1·6)
Germany	12·9 (9·6 – 16·7)	2·4 (1·6 – 3·6)	13·7 (10·6 – 17·3)	1·0 (0·6 – 1·6)	0·1 (0·1 – 0·2)	54·8 (49·1 – 60·4)	7·1 (5·3 – 9·5)	2·2 (1·7 – 2·8)	0·3 (0·2 – 0·3)	3·5 (2·6 – 4·6)	1·1 (0·8 – 1·5)	0·9 (0·7 – 1·2)
Greece	7·7 (5·4 – 10·7)	2·8 (1·9 – 4·2)	14·0 (10·6 – 17·9)	0·5 (0·3 – 0·8)	0·1 (0·1 – 0·1)	49·0 (42·9 – 55·3)	6·5 (4·8 – 8·8)	5·4 (4·5 – 6·6)	0·6 (0·5 – 0·8)	5·3 (3·8 – 7·0)	5·1 (3·7 – 6·8)	2·9 (2·2 – 3·8)
Iceland	6·9 (4·8 – 9·4)	2·3 (1·6 – 3·3)	12·6 (9·7 – 16·2)	1·1 (0·7 – 1·8)	0·2 (0·1 – 0·2)	53·0 (47·6 – 58·7)	8·3 (6·4 – 10·5)	4·6 (3·8 – 5·7)	0·4 (0·3 – 0·5)	3·9 (2·9 – 4·9)	4·4 (3·3 – 5·9)	2·3 (1·8 – 3·1)
Ireland	12·8 (9·4 – 17·3)	2·5 (1·7 – 3·6)	10·7 (8·2 – 13·9)	0·6 (0·4 – 0·9)	0·2 (0·1 – 0·3)	41·1 (35·0 – 47·9)	14·7 (11·3 – 18·6)	4·3 (3·4 – 5·5)	0·5 (0·4 – 0·7)	5·9 (4·1 – 7·9)	2·9 (2·1 – 3·9)	3·9 (2·7 – 5·5)
Israel	6·8 (4·6 – 9·3)	2·4 (1·7 – 3·5)	12·3 (9·4 – 15·8)	0·9 (0·6 – 1·5)	0·1 (0·1 – 0·2)	53·1 (47·6 – 58·4)	7·6 (5·8 – 10·1)	4·8 (3·9 – 6·0)	0·5 (0·4 – 0·7)	4·2 (3·1 – 5·5)	4·8 (3·5 – 6·4)	2·5 (1·9 – 3·3)
Italy	8·0 (5·6 – 11·1)	2·6 (1·7 – 3·7)	14·1 (11·0 – 17·6)	0·7 (0·4 – 1·1)	0·2 (0·1 – 0·2)	49·8 (44·0 – 55·4)	6·8 (5·0 – 8·9)	5·1 (4·3 – 6·2)	0·5 (0·4 – 0·7)	4·9 (3·7 – 6·4)	4·5 (3·2 – 6·1)	2·9 (2·2 – 3·9)
Luxembourg	7·9 (5·6 – 11·0)	2·3 (1·6 – 3·3)	13·8 (10·7 – 17·3)	1·6 (1·0 – 2·5)	0·2 (0·1 – 0·3)	49·2 (43·5 – 54·4)	11·2 (8·7 – 13·9)	3·6 (3·0 – 4·4)	0·3 (0·2 – 0·4)	3·4 (2·5 – 4·5)	4·6 (3·3 – 6·2)	1·9 (1·4 – 2·4)

Malta	6·8 (4·6 – 9·7)	2·8 (1·8 – 4·1)	12·8 (9·6 – 16·7)	0·4 (0·2 – 0·7)	0·1 (0·1 – 0·2)	50·7 (44·4 – 57·3)	5·3 (3·8 – 7·3)	6·1 (5·0 – 7·3)	0·8 (0·6 – 1·0)	5·8 (4·4 – 7·6)	4·8 (3·5 – 6·6)	3·6 (2·7 – 4·8)
Monaco	7·4 (5·3 – 10·2)	1·7 (1·2 – 2·4)	12·2 (9·7 – 15·2)	4·4 (3·2 – 6·0)	1·3 (0·9 – 1·7)	44·1 (39·4 – 48·9)	20·2 (16·6 – 24·1)	1·9 (1·7 – 2·2)	0·1 (0·1 – 0·2)	1·8 (1·3 – 2·5)	3·9 (2·9 – 5·2)	1·0 (0·7 – 1·3)
Netherlands	6·3 (4·6 – 8·4)	12·1 (8·8 – 15·6)	6·2 (4·7 – 8·1)	1·4 (0·9 – 2·1)	0·2 (0·1 – 0·2)	54·5 (49·7 – 59·4)	8·1 (6·3 – 10·3)	2·3 (1·8 – 2·8)	0·2 (0·2 – 0·3)	3·9 (2·9 – 5·2)	2·9 (2·2 – 3·8)	2·0 (1·5 – 2·6)
Norway	8·5 (6·0 – 11·5)	2·7 (1·9 – 3·8)	25·8 (21·3 – 30·7)	1·8 (1·1 – 2·8)	0·1 (0·1 – 0·2)	48·8 (44·1 – 54·0)	4·4 (3·2 – 5·8)	3·4 (2·8 – 4·2)	0·4 (0·3 – 0·5)	0·9 (0·6 – 1·1)	0·7 (0·5 – 1·0)	2·6 (1·9 – 3·4)
Portugal	2·0 (1·4 – 2·7)	0·3 (0·2 – 0·4)	6·9 (5·3 – 8·7)	1·5 (1·0 – 2·2)	0·1 (0·1 – 0·1)	34·7 (29·5 – 40·6)	5·9 (4·4 – 7·9)	8·7 (7·4 – 10·0)	0·7 (0·5 – 0·9)	7·0 (5·4 – 8·9)	29·3 (24·0 – 34·6)	3·0 (2·3 – 3·9)
San Marino	7·4 (5·3 – 9·8)	2·2 (1·5 – 3·1)	13·3 (10·5 – 16·6)	1·8 (1·2 – 2·8)	0·2 (0·2 – 0·3)	50·3 (45·0 – 55·4)	11·9 (9·2 – 14·9)	3·4 (2·8 – 4·0)	0·3 (0·2 – 0·3)	3·1 (2·3 – 4·1)	4·5 (3·3 – 6·0)	1·7 (1·3 – 2·3)
Spain	5·4 (3·7 – 8·0)	10·0 (6·9 – 13·8)	13·7 (10·6 – 17·3)	0·1 (0·1 – 0·1)	0·1 (0·1 – 0·2)	25·9 (20·8 – 31·4)	22·1 (17·9 – 26·7)	1·8 (1·5 – 2·3)	0·8 (0·6 – 1·0)	1·5 (1·1 – 1·9)	4·4 (3·2 – 6·0)	14·2 (11·0 – 17·9)
Sweden	4·6 (3·2 – 6·3)	2·1 (1·4 – 3·0)	20·6 (16·9 – 24·6)	0·8 (0·5 – 1·1)	0·5 (0·3 – 0·7)	34·3 (29·6 – 38·8)	18·3 (14·9 – 22·0)	8·1 (7·1 – 9·3)	0·2 (0·2 – 0·3)	3·6 (2·5 – 4·8)	6·3 (4·8 – 8·2)	0·7 (0·5 – 0·9)
Switzerland	6·5 (4·8 – 8·9)	1·8 (1·3 – 2·5)	12·1 (9·4 – 15·2)	3·8 (2·7 – 5·5)	0·8 (0·6 – 1·2)	47·7 (42·8 – 52·5)	17·6 (14·3 – 21·2)	2·3 (1·9 – 2·7)	0·1 (0·1 – 0·2)	2·1 (1·5 – 2·7)	4·0 (3·0 – 5·3)	1·1 (0·9 – 1·4)
UK	18·9 (15·0 – 22·8)	13·1 (9·7 – 16·6)	7·3 (5·7 – 9·3)	0·8 (0·5 – 1·1)	0·3 (0·2 – 0·4)	38·8 (34·3 – 43·6)	11·9 (9·3 – 14·8)	2·4 (2·0 – 2·9)	0·3 (0·2 – 0·3)	1·6 (1·2 – 2·1)	4·0 (2·9 – 5·3)	0·6 (0·5 – 0·8)
**Latin America and Caribbean**	**35·2 (31·8 – 38·8)**	**1·1 (0·9 – 1·4)**	**9·5 (8·5 – 10·6)**	**1·0 (0·8 – 1·3)**	**0·1 (0·1 – 0·2)**	**25·0 (21·8 – 28·4)**	**2·8 (2·5 – 3·2)**	**3·8 (3·5 – 4·1)**	**2·5 (2·2 – 2·9)**	**10·3 (9·1 – 11·5)**	**6·3 (5·3 – 7·3)**	**2·5 (2·2 – 2·9)**
Andean Latin America	14·2 (11·7 – 16·9)	0·5 (0·4 – 0·7)	10·6 (8·7 – 12·6)	1·4 (1·0 – 2·2)	0·0 (0·0 – 0·1)	11·3 (9·1 – 13·8)	2·3 (1·8 – 2·9)	5·3 (4·7 – 6·1)	5·4 (4·3 – 6·6)	36·9 (33·3 – 40·7)	7·0 (5·5 – 8·8)	5·1 (4·0 – 6·5)
Bolivia	9·7 (6·1 – 13·8)	0·4 (0·3 – 0·5)	12·7 (9·1 – 17·3)	0·6 (0·4 – 1·0)	0·1 (0·0 – 0·1)	4·1 (2·8 – 6·0)	1·1 (0·8 – 1·5)	1·6 (1·3 – 1·9)	13·4 (9·9 – 17·5)	50·6 (44·2 – 56·8)	2·8 (2·0 – 3·8)	3·0 (2·1 – 4·0)
Ecuador	26·4 (20·0 – 33·7)	0·5 (0·3 – 0·6)	15·0 (11·1 – 20·1)	0·6 (0·3 – 0·9)	0·0 (0·0 – 0·1)	17·3 (12·4 – 22·7)	1·5 (1·1 – 2·2)	7·7 (6·4 – 9·2)	5·7 (3·9 – 8·3)	18·5 (14·5 – 23·1)	5·7 (4·1 – 7·9)	1·1 (0·8 – 1·5)
Peru	10·0 (6·9 – 13·4)	0·6 (0·4 – 0·8)	7·8 (5·6 – 10·4)	2·1 (1·3 – 3·4)	0·0 (0·0 – 0·1)	10·8 (7·8 – 14·6)	3·0 (2·1 – 4·1)	5·5 (4·5 – 6·6)	2·5 (1·8 – 3·4)	41·0 (35·4 – 47·2)	9·0 (6·5 – 11·9)	7·7 (5·8 – 9·9)
Caribbean	30·2 (27·4 – 33·2)	1·0 (0·8 – 1·3)	28·6 (25·2 – 32·1)	1·3 (1·0 – 1·6)	0·1 (0·1 – 0·2)	15·9 (14·1 – 17·9)	7·2 (6·2 – 8·3)	2·9 (2·7 – 3·1)	2·2 (1·8 – 2·7)	5·5 (4·8 – 6·3)	3·5 (3·0 – 4·1)	1·5 (1·4 – 1·8)
Antigua and Barbuda	35·2 (29·4 – 41·3)	0·5 (0·4 – 0·6)	4·0 (3·0 – 5·1)	1·4 (1·0 – 1·9)	0·2 (0·2 – 0·3)	28·9 (23·9 – 34·1)	5·7 (4·2 – 7·6)	2·7 (2·3 – 3·2)	0·8 (0·7 – 1·0)	2·6 (2·0 – 3·3)	2·2 (1·7 – 2·9)	15·8 (12·7 – 19·4)
The Bahamas	31·0 (26·3 – 36·4)	0·5 (0·4 – 0·6)	6·5 (4·9 – 8·6)	2·0 (1·4 – 2·9)	1·6 (1·2 – 2·3)	40·3 (34·6 – 45·7)	4·1 (3·1 – 5·5)	7·9 (6·6 – 9·3)	0·5 (0·4 – 0·7)	1·8 (1·4 – 2·4)	3·0 (2·2 – 4·0)	0·9 (0·7 – 1·1)
Barbados	26·7 (21·1 – 33·1)	0·6 (0·5 – 0·9)	6·6 (4·9 – 8·8)	3·1 (1·9 – 4·8)	0·1 (0·1 – 0·1)	30·1 (23·7 – 36·8)	13·2 (9·6 – 17·8)	5·9 (4·9 – 7·1)	1·1 (0·8 – 1·5)	3·7 (2·9 – 4·9)	4·8 (3·3 – 6·7)	4·1 (2·8 – 6·2)
Belize	40·4 (32·9 – 47·8)	0·7 (0·5 – 0·9)	3·4 (2·3 – 4·6)	2·1 (1·3 – 3·4)	0·0 (0·0 – 0·1)	27·2 (21·3 – 33·6)	3·0 (2·2 – 4·2)	3·7 (3·0 – 4·4)	5·6 (3·8 – 8·1)	10·3 (7·7 – 13·7)	2·4 (1·7 – 3·4)	1·4 (1·0 – 1·8)
Bermuda	29·6 (24·2 – 35·0)	0·6 (0·4 – 0·8)	5·6 (4·3 – 7·4)	7·8 (5·7 – 10·9)	1·5 (1·1 – 2·0)	27·3 (22·1 – 32·6)	17·4 (14·0 – 21·2)	2·7 (2·4 – 3·2)	0·7 (0·5 – 0·9)	2·5 (1·9 – 3·2)	3·3 (2·4 – 4·4)	1·1 (0·9 – 1·4)
Cuba	22·9 (18·3 – 28·4)	0·3 (0·2 – 0·4)	53·9 (47·9 – 59·5)	0·2 (0·1 – 0·3)	0·1 (0·0 – 0·1)	10·4 (7·4 – 13·9)	6·9 (5·1 – 9·2)	1·5 (1·3 – 1·8)	0·9 (0·7 – 1·2)	1·4 (1·1 – 1·9)	0·5 (0·3 – 0·6)	1·1 (0·8 – 1·4)
Dominica	40·9 (34·5 – 47·3)	0·5 (0·4 – 0·7)	4·5 (3·3 – 6·1)	4·5 (2·9 – 6·7)	0·1 (0·1 – 0·1)	24·9 (20·0 – 30·8)	4·7 (3·4 – 6·4)	3·7 (3·1 – 4·3)	1·8 (1·3 – 2·5)	3·9 (3·0 – 5·1)	2·9 (2·0 – 4·0)	7·6 (5·7 – 10·1)
Dominican Republic	50·5 (43·7 – 57·4)	0·5 (0·4 – 0·7)	6·3 (4·6 – 8·3)	0·2 (0·1 – 0·3)	0·1 (0·1 – 0·1)	18·9 (14·3 – 24·5)	2·0 (1·5 – 2·8)	4·7 (3·8 – 5·7)	3·1 (2·2 – 4·5)	5·6 (4·2 – 7·3)	6·2 (4·4 – 8·2)	1·9 (1·4 – 2·6)
Grenada	34·0 (26·7 – 41·4)	0·8 (0·6 – 1·1)	6·0 (4·3 – 8·0)	1·0 (0·6 – 1·6)	0·1 (0·1 – 0·1)	28·4 (21·7 – 35·7)	5·7 (4·1 – 7·7)	6·1 (5·1 – 7·3)	3·8 (2·8 – 5·2)	6·3 (4·7 – 8·3)	4·5 (3·2 – 6·3)	3·2 (2·4 – 4·2)
Guyana	23·4 (18·2 – 29·2)	0·7 (0·5 – 0·9)	16·9 (13·2 – 21·5)	1·0 (0·6 – 1·6)	0·2 (0·1 – 0·3)	27·2 (21·7 – 33·6)	9·8 (7·6 – 12·7)	7·4 (6·3 – 8·7)	2·1 (1·6 – 2·8)	5·5 (4·1 – 7·1)	3·1 (2·3 – 4·1)	2·7 (1·9 – 3·7)
Haiti	5·7 (3·8 – 8·3)	1·8 (1·3 – 2·6)	3·3 (2·5 – 4·3)	6·4 (4·3 – 9·4)	0·8 (0·5 – 1·2)	16·8 (12·2 – 22·6)	7·1 (5·3 – 9·5)	4·7 (4·0 – 5·6)	14·5 (10·9 – 19·1)	20·2 (16·0 – 24·9)	14·7 (11·4 – 18·8)	3·9 (2·9 – 5·2)
Jamaica	29·1 (24·0 – 34·6)	0·7 (0·5 – 0·9)	4·6 (3·5 – 6·2)	7·2 (5·0 – 10·3)	0·1 (0·1 – 0·2)	32·7 (27·0 – 38·3)	11·9 (9·3 – 15·1)	4·2 (3·5 – 5·0)	2·3 (1·7 – 2·9)	1·9 (1·4 – 2·6)	4·3 (3·1 – 5·7)	1·1 (0·8 – 1·4)
Puerto Rico	54·7 (49·1 – 60·5)	3·9 (2·8 – 5·5)	1·4 (1·0 – 2·0)	0·6 (0·4 – 1·1)	0·1 (0·1 – 0·1)	11·5 (8·3 – 15·7)	6·3 (4·7 – 8·2)	1·3 (1·1 – 1·5)	0·4 (0·3 – 0·6)	13·3 (10·3 – 17·0)	5·7 (4·2 – 7·4)	0·7 (0·5 – 0·9)
Saint Kitts and Nevis	12·5 (8·8 – 17·0)	0·6 (0·5 – 0·8)	9·2 (7·0 – 11·8)	1·1 (0·8 – 1·7)	0·1 (0·1 – 0·2)	27·7 (22·5 – 33·4)	6·2 (4·8 – 7·9)	19·7 (17·0 – 22·8)	1·5 (1·2 – 2·0)	3·7 (2·9 – 4·8)	2·8 (2·1 – 3·7)	14·8 (11·7 – 18·3)
Saint Lucia	14·0 (9·4 – 19·4)	0·9 (0·6 – 1·2)	6·9 (4·9 – 9·5)	0·3 (0·2 – 0·5)	0·0 (0·0 – 0·1)	39·2 (31·6 – 46·1)	5·1 (3·7 – 7·0)	7·0 (5·7 – 8·5)	9·1 (6·7 – 12·2)	10·0 (7·3 – 13·6)	2·3 (1·6 – 3·5)	5·2 (3·8 – 7·0)
Saint Vincent and the Grenadines	34·8 (29·4 – 40·2)	0·4 (0·3 – 0·5)	4·6 (3·3 – 6·5)	1·6 (1·0 – 2·6)	0·0 (0·0 – 0·0)	32·8 (27·3 – 38·6)	5·5 (4·0 – 7·4)	3·3 (2·7 – 3·8)	2·6 (1·9 – 3·4)	3·3 (2·5 – 4·3)	1·9 (1·4 – 2·7)	9·2 (6·8 – 12·2)
Suriname	19·5 (13·7 – 26·0)	0·6 (0·5 – 0·9)	4·3 (2·9 – 6·0)	1·4 (0·9 – 2·3)	0·1 (0·1 – 0·3)	60·8 (53·3 – 68·0)	3·8 (2·8 – 5·2)	2·9 (2·4 – 3·5)	1·6 (1·2 – 2·3)	2·3 (1·7 – 3·1)	0·7 (0·5 – 1·0)	1·9 (1·4 – 2·6)
Trinidad and Tobago	11·2 (8·1 – 14·9)	0·6 (0·4 – 0·8)	5·3 (3·9 – 7·3)	1·0 (0·6 – 1·6)	0·0 (0·0 – 0·1)	31·3 (25·6 – 37·7)	22·3 (17·8 – 27·1)	11·8 (9·7 – 14·4)	1·1 (0·8 – 1·5)	6·9 (5·3 – 8·9)	6·3 (4·6 – 8·3)	2·2 (1·6 – 2·9)
Virgin Islands	31·4 (25·9 – 37·0)	0·7 (0·5 – 0·9)	6·3 (4·8 – 8·3)	4·9 (3·4 – 7·0)	0·6 (0·5 – 0·9)	29·2 (24·4 – 34·8)	13·7 (10·7 – 17·1)	3·6 (3·1 – 4·1)	1·0 (0·8 – 1·4)	3·5 (2·6 – 4·4)	3·6 (2·6 – 4·7)	1·5 (1·2 – 1·9)
Central Latin America	22·9 (20·4 – 25·7)	0·9 (0·7 – 1·0)	15·6 (13·5 – 18·0)	1·7 (1·3 – 2·4)	0·1 (0·1 – 0·1)	24·2 (21·5 – 27·1)	3·1 (2·7 – 3·6)	6·3 (5·7 – 7·1)	2·4 (2·0 – 2·8)	10·5 (9·2 – 12·0)	7·9 (6·8 – 9·2)	4·4 (3·7 – 5·3)
Colombia	17·9 (13·9 – 22·5)	0·8 (0·6 – 1·1)	17·8 (14·2 – 22·2)	0·9 (0·6 – 1·4)	0·1 (0·1 – 0·1)	22·4 (18·0 – 27·4)	2·6 (1·9 – 3·5)	10·5 (9·0 – 12·2)	1·9 (1·3 – 2·7)	13·0 (10·3 – 16·0)	9·3 (7·1 – 11·8)	2·8 (2·1 – 3·7)
Costa Rica	20·0 (16·4 – 24·1)	1·2 (0·9 – 1·7)	8·6 (6·7 – 10·8)	1·4 (0·9 – 2·1)	0·0 (0·0 – 0·1)	30·4 (25·7 – 35·1)	10·6 (8·3 – 13·4)	2·7 (2·3 – 3·2)	9·7 (7·5 – 12·3)	9·1 (7·4 – 11·2)	5·2 (4·0 – 6·8)	0·8 (0·6 – 1·1)
El Salvador	52·5 (45·2 – 59·6)	1·6 (1·1 – 2·4)	5·9 (4·2 – 8·4)	1·2 (0·7 – 2·0)	0·1 (0·0 – 0·1)	17·8 (12·8 – 23·5)	2·6 (1·8 – 3·7)	2·0 (1·6 – 2·4)	1·7 (1·3 – 2·3)	9·5 (6·8 – 12·5)	4·2 (2·9 – 6·0)	0·8 (0·6 – 1·1)
Guatemala	40·0 (31·5 – 49·0)	2·5 (1·6 – 3·7)	6·6 (4·4 – 9·3)	3·9 (2·4 – 6·3)	0·1 (0·1 – 0·2)	18·3 (13·1 – 24·9)	3·9 (2·7 – 5·4)	2·7 (2·1 – 3·4)	3·5 (2·4 – 5·0)	14·5 (10·4 – 19·4)	3·2 (2·2 – 4·5)	0·8 (0·6 – 1·1)
Honduras	36·3 (29·5 – 43·5)	0·6 (0·4 – 0·8)	11·6 (8·5 – 15·4)	0·7 (0·4 – 1·2)	0·1 (0·0 – 0·1)	20·7 (15·4 – 27·3)	3·1 (2·2 – 4·3)	2·1 (1·7 – 2·4)	1·5 (1·1 – 2·2)	12·5 (9·5 – 16·1)	10·0 (7·1 – 13·5)	0·9 (0·6 – 1·1)
Mexico	20·3 (15·7 – 25·7)	0·9 (0·6 – 1·2)	16·4 (12·7 – 20·7)	2·7 (1·7 – 4·1)	0·1 (0·0 – 0·1)	25·1 (20·0 – 30·8)	2·0 (1·4 – 2·8)	6·3 (5·3 – 7·4)	2·5 (1·8 – 3·4)	9·7 (7·4 – 12·4)	7·7 (5·8 – 10·4)	6·3 (4·8 – 8·2)
Nicaragua	45·0 (38·3 – 51·5)	0·8 (0·6 – 1·1)	13·1 (10·0 – 17·0)	1·6 (1·0 – 2·7)	0·1 (0·1 – 0·1)	19·9 (15·2 – 25·3)	2·3 (1·6 – 3·2)	2·0 (1·7 – 2·5)	5·6 (3·9 – 7·4)	6·2 (4·5 – 8·4)	2·1 (1·4 – 3·0)	1·2 (1·0 – 1·7)
Panama	38·1 (33·1 – 42·9)	0·7 (0·5 – 1·0)	6·7 (5·2 – 8·5)	1·1 (0·7 – 1·7)	0·1 (0·1 – 0·2)	32·1 (27·4 – 36·9)	1·8 (1·3 – 2·3)	5·1 (4·3 – 6·0)	1·4 (1·0 – 1·9)	6·3 (4·9 – 8·0)	4·2 (3·2 – 5·6)	2·5 (1·9 – 3·2)
Venezuela	20·8 (16·3 – 25·6)	0·4 (0·3 – 0·5)	17·3 (13·8 – 20·9)	0·3 (0·2 – 0·5)	0·2 (0·1 – 0·2)	26·1 (21·4 – 31·5)	6·3 (4·7 – 8·4)	3·3 (2·8 – 3·9)	1·2 (0·9 – 1·6)	9·4 (7·3 – 11·8)	9·4 (7·3 – 12·0)	5·2 (3·9 – 6·7)
Tropical Latin America	47·1 (40·6 – 53·9)	1·4 (0·9 – 2·0)	1·4 (1·0 – 1·8)	0·4 (0·3 – 0·7)	0·1 (0·1 – 0·2)	29·4 (23·2 – 35·8)	1·8 (1·3 – 2·5)	2·1 (1·7 – 2·5)	2·2 (1·6 – 2·9)	7·3 (5·4 – 9·6)	5·7 (4·1 – 7·8)	1·1 (0·8 – 1·4)
Brazil	47·6 (41·0 – 54·5)	1·4 (0·9 – 2·0)	1·3 (0·9 – 1·7)	0·4 (0·2 – 0·7)	0·1 (0·1 – 0·2)	29·5 (23·3 – 36·0)	1·8 (1·3 – 2·5)	2·1 (1·7 – 2·5)	1·9 (1·3 – 2·6)	7·3 (5·4 – 9·6)	5·7 (4·1 – 7·8)	0·9 (0·7 – 1·3)
Paraguay	7·9 (5·6 – 11·0)	0·4 (0·3 – 0·5)	9·0 (6·7 – 11·8)	1·9 (1·2 – 2·7)	0·1 (0·0 – 0·1)	18·7 (14·5 – 23·0)	3·2 (2·3 – 4·4)	2·2 (1·9 – 2·6)	28·1 (23·2 – 33·2)	9·0 (6·9 – 11·5)	5·0 (3·7 – 6·7)	14·5 (11·5 – 18·5)
**North Africa and Middle East**	**7·7 (6·3 – 9·3)**	**1·3 (1·0 – 1·7)**	**16·1 (14·0 – 18·3)**	**0·7 (0·5 – 1·0)**	**0·0 (0·0 – 0·0)**	**27·9 (25·1 – 31·0)**	**3·7 (3·0 – 4·5)**	**2·9 (2·7 – 3·2)**	**12·1 (10·8 – 13·5)**	**5·1 (4·5 – 5·7)**	**18·9 (16·4 – 21·6)**	**3·6 (2·9 – 4·5)**
North Africa and Middle East	7·7 (6·3 – 9·3)	1·3 (1·0 – 1·7)	16·1 (14·0 – 18·3)	0·7 (0·5 – 1·0)	0·0 (0·0 – 0·0)	27·9 (25·1 – 31·0)	3·7 (3·0 – 4·5)	2·9 (2·7 – 3·2)	12·1 (10·8 – 13·5)	5·1 (4·5 – 5·7)	18·9 (16·4 – 21·6)	3·6 (2·9 – 4·5)
Afghanistan	8·8 (5·5 – 13·4)	1·2 (0·8 – 1·7)	6·4 (4·5 – 9·3)	12·4 (7·8 – 18·0)	0·2 (0·2 – 0·4)	25·1 (18·4 – 33·6)	6·4 (4·6 – 9·0)	4·3 (3·5 – 5·2)	24·5 (18·3 – 31·9)	2·9 (2·1 – 4·1)	5·1 (3·5 – 7·5)	2·6 (1·8 – 3·7)
Algeria	1·5 (0·8 – 2·9)	0·3 (0·2 – 0·4)	4·0 (2·6 – 6·0)	0·0 (0·0 – 0·0)	0·0 (0·0 – 0·0)	52·2 (44·5 – 60·2)	0·3 (0·2 – 0·5)	2·3 (1·8 – 3·0)	25·0 (19·1 – 31·5)	10·1 (7·2 – 13·8)	2·9 (2·0 – 4·2)	1·2 (0·8 – 1·8)
Bahrain	8·9 (5·5 – 13·4)	0·4 (0·3 – 0·6)	4·8 (3·2 – 6·9)	0·0 (0·0 – 0·0)	0·0 (0·0 – 0·1)	14·8 (10·3 – 19·6)	4·9 (3·4 – 6·8)	4·4 (3·5 – 5·5)	10·4 (7·6 – 13·7)	10·2 (7·2 – 13·8)	39·4 (32·4 – 46·5)	1·8 (1·2 – 2·5)
Egypt	3·4 (2·1 – 5·6)	0·6 (0·4 – 0·9)	36·5 (28·2 – 45·3)	0·6 (0·3 – 0·9)	0·0 (0·0 – 0·0)	38·7 (30·2 – 47·6)	1·9 (1·3 – 2·7)	2·8 (2·2 – 3·5)	10·6 (7·4 – 14·6)	2·2 (1·4 – 3·2)	1·1 (0·7 – 1·6)	1·7 (1·2 – 2·5)
Iran	18·0 (13·0 – 23·5)	3·7 (2·6 – 5·1)	12·9 (9·4 – 17·4)	0·3 (0·2 – 0·4)	0·0 (0·0 – 0·0)	31·6 (24·8 – 39·3)	4·0 (2·8 – 5·6)	1·9 (1·5 – 2·2)	5·1 (3·7 – 7·1)	1·8 (1·2 – 2·5)	19·8 (14·8 – 25·5)	0·9 (0·7 – 1·3)
Iraq	5·3 (3·1 – 8·4)	0·3 (0·2 – 0·4)	16·4 (11·8 – 21·6)	0·2 (0·1 – 0·4)	0·0 (0·0 – 0·0)	15·4 (11·3 – 20·5)	0·5 (0·4 – 0·7)	1·6 (1·3 – 1·9)	36·0 (29·6 – 43·1)	7·0 (4·9 – 9·7)	15·7 (11·3 – 20·5)	1·8 (1·2 – 2·5)
Jordan	7·0 (4·0 – 10·8)	0·3 (0·2 – 0·4)	25·5 (19·4 – 32·3)	0·1 (0·0 – 0·1)	0·0 (0·0 – 0·0)	11·6 (8·0 – 16·6)	0·9 (0·6 – 1·3)	2·8 (2·3 – 3·4)	26·8 (20·5 – 34·1)	14·1 (10·4 – 18·6)	9·7 (6·7 – 13·1)	1·2 (0·9 – 1·7)
Kuwait	2·2 (1·3 – 3·6)	0·7 (0·5 – 1·0)	9·5 (6·5 – 13·2)	0·0 (0·0 – 0·1)	0·0 (0·0 – 0·0)	46·9 (38·1 – 55·4)	1·0 (0·7 – 1·4)	2·8 (2·2 – 3·6)	20·3 (15·2 – 26·8)	10·8 (7·7 – 14·6)	3·0 (2·1 – 4·5)	2·7 (1·8 – 4·0)
Lebanon	6·1 (3·7 – 9·5)	0·3 (0·2 – 0·4)	25·2 (19·0 – 31·6)	0·1 (0·1 – 0·2)	0·1 (0·0 – 0·1)	16·8 (12·1 – 22·4)	4·6 (3·2 – 6·3)	1·3 (1·1 – 1·5)	3·9 (2·7 – 5·6)	22·4 (17·3 – 28·3)	17·9 (13·4 – 23·3)	1·4 (1·0 – 2·0)
Libya	4·1 (2·3 – 6·6)	0·3 (0·2 – 0·4)	9·8 (6·8 – 13·2)	0·0 (0·0 – 0·0)	0·0 (0·0 – 0·0)	8·8 (5·9 – 12·5)	0·3 (0·2 – 0·4)	1·5 (1·2 – 1·8)	34·8 (28·4 – 41·9)	33·0 (26·6 – 39·6)	2·8 (1·9 – 4·0)	4·5 (2·8 – 7·0)
Morocco	4·1 (2·4 – 6·3)	0·3 (0·2 – 0·5)	6·1 (4·1 – 8·9)	0·1 (0·1 – 0·2)	0·0 (0·0 – 0·0)	44·9 (36·8 – 53·0)	0·7 (0·4 – 1·0)	1·6 (1·3 – 2·0)	25·8 (19·8 – 32·5)	8·6 (6·1 – 11·9)	5·1 (3·5 – 7·3)	2·7 (1·8 – 3·7)
Oman	15·6 (9·9 – 23·0)	0·6 (0·4 – 0·9)	5·8 (3·9 – 8·3)	0·4 (0·2 – 0·6)	0·0 (0·0 – 0·0)	9·7 (6·6 – 13·8)	0·9 (0·6 – 1·3)	3·9 (3·1 – 4·7)	40·3 (32·8 – 48·8)	10·7 (7·6 – 14·8)	8·5 (5·8 – 12·2)	3·6 (2·6 – 5·1)
Palestine	4·2 (2·4 – 6·9)	0·3 (0·2 – 0·4)	38·1 (30·8 – 45·6)	0·1 (0·1 – 0·2)	0·0 (0·0 – 0·0)	7·7 (5·2 – 11·1)	1·8 (1·2 – 2·6)	2·2 (1·8 – 2·8)	20·8 (15·3 – 27·3)	13·2 (9·7 – 17·6)	10·6 (7·4 – 14·2)	0·9 (0·6 – 1·3)
Qatar	5·1 (3·0 – 8·3)	2·6 (1·7 – 3·7)	19·5 (13·9 – 26·1)	0·6 (0·4 – 1·0)	0·0 (0·0 – 0·0)	28·3 (20·5 – 36·3)	2·0 (1·4 – 2·9)	4·2 (3·4 – 5·3)	14·9 (10·5 – 20·5)	10·1 (7·1 – 13·7)	10·4 (7·2 – 14·6)	2·2 (1·5 – 3·0)
Saudi Arabia	0·9 (0·5 – 1·6)	0·7 (0·5 – 1·0)	16·0 (11·3 – 21·8)	0·1 (0·0 – 0·1)	0·0 (0·0 – 0·0)	38·3 (29·8 – 47·3)	0·6 (0·4 – 0·8)	3·6 (2·8 – 4·8)	28·2 (21·5 – 35·4)	7·3 (5·0 – 10·1)	2·8 (1·8 – 4·0)	1·7 (1·2 – 2·4)
Sudan	7·6 (4·5 – 12·2)	1·5 (1·0 – 2·3)	4·6 (2·9 – 7·1)	0·7 (0·4 – 1·3)	0·0 (0·0 – 0·0)	40·3 (30·9 – 49·5)	0·9 (0·6 – 1·3)	5·0 (4·0 – 6·0)	19·3 (13·6 – 26·4)	13·4 (9·6 – 18·5)	2·1 (1·4 – 3·1)	4·5 (3·0 – 6·7)
Syria	2·5 (1·5 – 4·0)	0·6 (0·3 – 0·9)	6·7 (4·6 – 9·3)	0·3 (0·2 – 0·4)	0·0 (0·0 – 0·0)	31·0 (23·4 – 38·5)	1·1 (0·7 – 1·6)	5·1 (4·0 – 6·3)	27·7 (21·7 – 34·7)	18·9 (14·0 – 24·5)	5·0 (3·4 – 6·8)	1·3 (0·8 – 1·9)
Tunisia	18·7 (13·4 – 25·6)	0·3 (0·2 – 0·5)	26·3 (20·1 – 33·4)	0·2 (0·1 – 0·3)	0·0 (0·0 – 0·0)	13·3 (9·2 – 18·3)	1·2 (0·8 – 1·8)	5·9 (4·8 – 7·1)	11·4 (8·1 – 15·2)	16·4 (12·4 – 21·3)	3·8 (2·6 – 5·4)	2·6 (1·9 – 3·7)
Turkey	3·0 (1·8 – 4·7)	0·4 (0·3 – 0·5)	15·2 (10·7 – 20·0)	0·2 (0·1 – 0·4)	0·0 (0·0 – 0·0)	11·3 (8·0 – 15·3)	7·2 (5·2 – 9·6)	4·1 (3·4 – 4·9)	3·3 (2·4 – 4·6)	3·2 (2·2 – 4·6)	43·0 (36·6 – 49·4)	9·1 (6·7 – 11·9)
United Arab Emirates	14·0 (8·8 – 20·9)	0·8 (0·5 – 1·1)	9·6 (6·5 – 13·4)	0·1 (0·1 – 0·2)	0·0 (0·0 – 0·0)	23·9 (17·4 – 31·6)	0·9 (0·6 – 1·3)	6·1 (4·9 – 7·7)	18·0 (13·3 – 23·8)	11·1 (7·8 – 15·2)	4·0 (2·7 – 6·0)	11·4 (8·4 – 15·5)
Yemen	7·5 (4·4 – 12·2)	0·9 (0·6 – 1·4)	9·7 (6·6 – 13·7)	1·0 (0·6 – 1·7)	0·0 (0·0 – 0·0)	17·5 (11·9 – 24·0)	0·4 (0·3 – 0·6)	3·1 (2·5 – 3·8)	44·2 (36·2 – 52·8)	8·7 (6·0 – 12·4)	5·0 (3·3 – 7·2)	1·9 (1·3 – 2·7)
**South Asia**	**31·7 (26·6 – 37·2)**	**12·3 (10·3 – 14·5)**	**3·2 (2·5 – 4·1)**	**0·7 (0·5 – 1·0)**	**0·0 (0·0 – 0·0)**	**6·2 (4·8 – 7·9)**	**9·4 (7·5 – 11·7)**	**2·1 (1·8 – 2·4)**	**2·8 (2·2 – 3·7)**	**15·8 (12·6 – 19·3)**	**13·7 (11·7 – 15·7)**	**2·1 (1·6 – 2·7)**
South Asia	31·7 (26·6 – 37·2)	12·3 (10·3 – 14·5)	3·2 (2·5 – 4·1)	0·7 (0·5 – 1·0)	0·0 (0·0 – 0·0)	6·2 (4·8 – 7·9)	9·4 (7·5 – 11·7)	2·1 (1·8 – 2·4)	2·8 (2·2 – 3·7)	15·8 (12·6 – 19·3)	13·7 (11·7 – 15·7)	2·1 (1·6 – 2·7)

Bangladesh	11·0 (7·2 – 15·3)	3·4 (2·3 – 4·9)	3·3 (2·3 – 4·6)	3·5 (2·0 – 5·7)	0·1 (0·0 – 0·1)	35·9 (28·4 – 44·2)	4·8 (3·4 – 6·8)	3·1 (2·6 – 3·8)	3·5 (2·4 – 5·0)	20·3 (15·4 – 25·8)	7·2 (5·1 – 10·1)	3·9 (2·7 – 5·4)
Bhutan	14·7 (10·1 – 20·5)	47·0 (38·8 – 54·9)	7·7 (5·2 – 10·8)	5·7 (3·3 – 9·0)	0·0 (0·0 – 0·0)	13·2 (8·9 – 18·3)	1·8 (1·1 – 2·7)	3·0 (2·4 – 3·8)	3·0 (2·1 – 4·3)	1·6 (1·1 – 2·2)	0·6 (0·4 – 0·9)	1·7 (1·2 – 2·3)
India	34·4 (28·4 – 41·1)	13·0 (10·8 – 15·5)	2·9 (2·1 – 3·9)	0·1 (0·0 – 0·2)	0·0 (0·0 – 0·0)	2·7 (1·8 – 4·0)	9·8 (7·7 – 12·4)	1·9 (1·6 – 2·2)	2·6 (1·8 – 3·6)	16·0 (12·2 – 20·2)	14·7 (12·6 – 17·1)	1·9 (1·3 – 2·5)
Nepal	25·6 (18·5 – 33·9)	33·8 (25·8 – 42·5)	1·4 (0·9 – 2·1)	7·4 (4·3 – 11·8)	0·3 (0·2 – 0·5)	7·5 (4·9 – 11·0)	3·3 (2·2 – 5·1)	2·9 (2·3 – 3·6)	5·0 (3·4 – 7·3)	5·1 (3·4 – 7·3)	6·0 (4·1 – 8·7)	1·5 (1·0 – 2·1)
Pakistan	24·9 (18·1 – 32·6)	1·5 (1·1 – 2·2)	12·4 (8·8 – 17·1)	3·4 (2·0 – 5·4)	0·1 (0·0 – 0·1)	12·0 (8·3 – 16·7)	15·8 (11·4 – 21·0)	5·4 (4·5 – 6·5)	5·3 (3·5 – 7·7)	5·6 (4·0 – 7·7)	10·1 (6·9 – 14·3)	3·7 (2·6 – 5·2)
**Southeast Asia, east Asia, and Oceania**	**30·6 (24·9 – 36·9)**	**7·1 (5·0 – 9·6)**	**44·4 (38·3 – 50·3)**	**0·4 (0·3 – 0·5)**	**0·0 (0·0 – 0·0)**	**6·2 (5·2 – 7·4)**	**1·3 (1·0 – 1·7)**	**1·8 (1·5 – 2·2)**	**1·9 (1·5 – 2·5)**	**3·2 (2·7 – 3·7)**	**1·2 (1·0 – 1·4)**	**1·9 (1·5 – 2·4)**
East Asia	34·2 (27·6 – 41·4)	8·0 (5·6 – 11·1)	48·4 (41·2 – 55·4)	0·0 (0·0 – 0·0)	0·0 (0·0 – 0·0)	1·7 (1·1 – 2·5)	1·0 (0·7 – 1·5)	1·8 (1·4 – 2·2)	1·7 (1·2 – 2·3)	1·4 (1·0 – 1·9)	0·2 (0·2 – 0·3)	1·6 (1·1 – 2·2)
China	34·9 (28·2 – 42·4)	8·3 (5·7 – 11·3)	47·6 (40·2 – 54·7)	0·0 (0·0 – 0·0)	0·0 (0·0 – 0·0)	1·7 (1·1 – 2·5)	1·0 (0·7 – 1·5)	1·8 (1·4 – 2·2)	1·7 (1·2 – 2·4)	1·2 (0·9 – 1·7)	0·2 (0·1 – 0·3)	1·6 (1·2 – 2·2)
North Korea	3·4 (2·2 – 5·1)	0·3 (0·2 – 0·4)	82·1 (78·2 – 85·5)	0·1 (0·0 – 0·1)	0·0 (0·0 – 0·1)	0·8 (0·5 – 1·2)	0·6 (0·4 – 0·9)	2·8 (2·3 – 3·4)	0·8 (0·5 – 1·2)	7·9 (5·4 – 10·9)	0·9 (0·6 – 1·3)	0·3 (0·2 – 0·4)
Taiwan (province of China)	17·2 (12·3 – 23·0)	1·9 (1·3 – 2·7)	69·7 (63·5 – 74·9)	0·1 (0·1 – 0·2)	0·1 (0·1 – 0·2)	2·0 (1·4 – 2·8)	1·6 (1·1 – 2·3)	1·9 (1·6 – 2·3)	0·9 (0·6 – 1·2)	3·2 (2·3 – 4·5)	0·6 (0·4 – 0·8)	0·8 (0·6 – 1·1)
Oceania	29·9 (25·3 – 35·2)	1·2 (0·9 – 1·6)	3·7 (3·0 – 4·5)	6·9 (5·5 – 9·1)	1·9 (1·5 – 2·3)	14·2 (11·7 – 17·1)	4·7 (3·9 – 5·9)	2·7 (2·4 – 3·0)	4·7 (3·5 – 6·5)	13·9 (11·2 – 17·2)	4·1 (3·4 – 5·1)	12·1 (9·5 – 15·2)
American Samoa	21·5 (16·5 – 27·2)	0·9 (0·7 – 1·2)	4·4 (3·3 – 5·8)	23·6 (18·0 – 30·0)	9·2 (6·8 – 12·2)	16·9 (12·8 – 21·7)	8·7 (6·5 – 11·5)	2·8 (2·2 – 3·3)	0·9 (0·7 – 1·1)	4·8 (3·5 – 6·4)	3·9 (2·9 – 5·1)	2·5 (1·9 – 3·1)
Cook Islands	12·7 (8·7 – 17·9)	1·5 (1·0 – 2·4)	3·4 (2·0 – 5·4)	5·3 (3·4 – 8·0)	0·5 (0·4 – 0·7)	35·8 (28·9 – 43·3)	0·6 (0·3 – 1·2)	2·4 (2·0 – 2·8)	1·9 (1·5 – 2·6)	3·3 (2·2 – 5·0)	4·0 (2·7 – 5·8)	28·4 (22·6 – 34·6)
Fiji	36·3 (31·5 – 41·2)	0·5 (0·4 – 0·6)	11·2 (8·9 – 13·9)	0·6 (0·4 – 0·9)	0·8 (0·6 – 1·1)	17·6 (14·2 – 21·4)	15·5 (12·6 – 18·7)	2·1 (1·7 – 2·5)	1·3 (1·0 – 1·6)	6·1 (4·8 – 7·7)	6·4 (4·9 – 8·1)	1·8 (1·4 – 2·4)
Guam	18·8 (14·3 – 23·5)	0·8 (0·6 – 1·0)	3·9 (2·9 – 5·1)	27·2 (21·1 – 33·5)	13·0 (9·8 – 17·0)	15·1 (11·1 – 19·7)	9·4 (7·1 – 12·4)	2·4 (2·0 – 2·9)	0·6 (0·5 – 0·8)	3·6 (2·7 – 4·8)	3·3 (2·5 – 4·3)	1·9 (1·5 – 2·4)
Kiribati	10·5 (7·1 – 14·7)	3·3 (2·2 – 4·8)	3·6 (2·6 – 5·1)	19·2 (13·5 – 26·3)	4·6 (3·0 – 6·8)	13·7 (9·7 – 18·7)	0·9 (0·7 – 1·2)	3·5 (2·9 – 4·1)	3·9 (2·6 – 5·9)	19·9 (15·2 – 25·8)	4·9 (3·4 – 6·8)	12·0 (9·0 – 15·8)
Marshall Islands	51·5 (42·7 – 59·8)	1·0 (0·7 – 1·4)	3·6 (2·5 – 5·2)	7·7 (4·9 – 11·4)	1·1 (0·6 – 1·8)	8·3 (5·3 – 12·0)	5·7 (3·4 – 8·9)	3·0 (2·4 – 3·6)	4·0 (2·7 – 5·8)	10·6 (7·7 – 14·6)	2·2 (1·3 – 3·9)	1·5 (1·0 – 2·1)
Federated States of Micronesia	31·0 (23·0 – 39·1)	1·5 (1·0 – 2·0)	5·6 (4·2 – 7·6)	4·2 (2·6 – 6·4)	0·4 (0·3 – 0·6)	18·6 (13·6 – 24·7)	2·7 (1·9 – 3·8)	4·4 (3·6 – 5·4)	4·6 (3·4 – 6·3)	14·7 (11·2 – 18·8)	5·4 (3·8 – 7·7)	7·0 (5·3 – 9·0)
Nauru	28·5 (22·2 – 35·4)	0·7 (0·6 – 1·0)	7·3 (5·0 – 10·4)	3·3 (2·0 – 5·5)	3·2 (2·2 – 4·5)	3·5 (2·5 – 4·9)	7·3 (5·2 – 10·0)	2·3 (2·0 – 2·8)	7·2 (5·5 – 9·5)	21·9 (18·0 – 26·3)	12·2 (9·2 – 15·9)	2·6 (1·9 – 3·7)
Niue	29·0 (22·1 – 36·2)	1·2 (0·9 – 1·7)	5·6 (4·2 – 7·8)	12·5 (8·5 – 18·4)	2·3 (1·6 – 3·4)	20·2 (15·1 – 26·3)	5·1 (3·9 – 6·7)	3·4 (2·8 – 4·2)	2·0 (1·5 – 2·7)	9·3 (6·8 – 12·2)	5·0 (3·7 – 6·8)	4·3 (3·3 – 5·5)
Northern Mariana Islands	15·8 (11·8 – 20·3)	0·7 (0·5 – 0·9)	3·3 (2·5 – 4·5)	29·3 (23·2 – 36·0)	16·3 (12·5 – 20·9)	13·5 (10·2 – 17·5)	10·9 (8·3 – 14·3)	2·2 (1·8 – 2·7)	0·5 (0·4 – 0·7)	2·9 (2·1 – 3·8)	3·0 (2·3 – 3·9)	1·6 (1·3 – 2·0)
Palau	5·7 (3·7 – 8·4)	0·7 (0·6 – 0·9)	4·3 (3·3 – 5·7)	5·8 (4·1 – 8·2)	3·5 (2·4 – 5·0)	50·6 (44·2 – 56·8)	2·4 (1·6 – 3·5)	3·2 (2·6 – 4·0)	0·8 (0·6 – 1·1)	3·7 (2·7 – 5·1)	0·8 (0·6 – 1·0)	18·5 (15·0 – 22·5)
Papua New Guinea	29·5 (21·9 – 38·0)	1·5 (1·0 – 2·1)	0·5 (0·4 – 0·7)	6·3 (4·0 – 9·9)	1·1 (0·7 – 1·7)	12·2 (8·3 – 17·0)	1·1 (0·8 – 1·6)	2·4 (2·0 – 2·9)	6·6 (4·6 – 9·3)	17·3 (12·9 – 22·6)	3·1 (2·1 – 4·6)	18·3 (14·0 – 23·3)
Samoa	19·0 (13·0 – 26·3)	1·2 (0·8 – 1·6)	4·1 (3·0 – 5·6)	20·6 (14·3 – 28·7)	0·4 (0·2 – 0·5)	31·6 (23·7 – 39·8)	1·6 (1·1 – 2·3)	4·9 (3·9 – 6·2)	3·4 (2·4 – 4·6)	6·7 (4·8 – 9·2)	2·7 (2·0 – 3·7)	3·8 (2·8 – 5·0)
Solomon Islands	37·2 (29·0 – 46·0)	1·5 (1·0 – 2·1)	6·7 (4·5 – 9·5)	6·5 (4·0 – 10·1)	0·4 (0·2 – 0·6)	3·8 (2·4 – 5·7)	2·8 (1·9 – 3·9)	3·9 (3·2 – 4·7)	1·8 (1·3 – 2·4)	25·1 (19·6 – 31·4)	7·8 (5·3 – 10·8)	2·6 (1·9 – 3·6)
Tokelau	32·4 (24·8 – 40·7)	1·3 (1·0 – 1·9)	5·7 (4·1 – 8·0)	8·1 (5·2 – 12·0)	1·1 (0·7 – 1·7)	20·1 (14·5 – 26·9)	3·6 (2·7 – 5·0)	3·5 (2·9 – 4·2)	2·7 (2·0 – 3·7)	11·2 (8·3 – 14·9)	5·0 (3·7 – 6·8)	5·1 (3·9 – 6·9)
Tonga	36·2 (28·4 – 44·6)	0·9 (0·6 – 1·2)	8·6 (6·0 – 11·8)	2·4 (1·4 – 3·8)	0·1 (0·1 – 0·1)	5·7 (3·7 – 8·6)	1·2 (0·8 – 1·7)	8·3 (6·8 – 10·1)	7·6 (5·4 – 10·5)	15·4 (11·6 – 19·9)	10·6 (7·6 – 14·5)	3·0 (2·2 – 4·1)
Tuvalu	18·9 (12·5 – 26·6)	1·8 (1·2 – 2·7)	6·8 (4·5 – 10·3)	8·0 (5·0 – 12·4)	0·8 (0·4 – 1·5)	7·7 (4·8 – 12·0)	1·3 (0·8 – 2·2)	5·5 (4·6 – 6·7)	5·8 (3·9 – 8·8)	27·3 (20·5 – 34·6)	4·4 (2·9 – 6·4)	11·5 (8·6 – 15·4)
Vanuatu	22·6 (16·0 – 30·3)	1·2 (0·8 – 1·8)	8·5 (5·9 – 12·2)	0·6 (0·3 – 1·0)	0·1 (0·1 – 0·1)	32·1 (24·9 – 40·3)	3·0 (2·1 – 4·2)	3·6 (2·9 – 4·4)	4·7 (2·9 – 7·3)	15·9 (12·1 – 20·2)	2·1 (1·4 – 3·1)	5·7 (4·2 – 7·6)
Southeast Asia	10·7 (9·2 – 12·4)	1·9 (1·5 – 2·3)	22·8 (19·9 – 26·1)	2·4 (1·8 – 3·2)	0·1 (0·1 – 0·1)	31·3 (27·6 – 35·5)	3·0 (2·5 – 3·6)	1·8 (1·6 – 2·0)	3·3 (2·8 – 3·9)	12·9 (11·3 – 14·9)	6·4 (5·2 – 7·5)	3·5 (2·8 – 4·4)
Cambodia	6·1 (3·8 – 10·0)	0·6 (0·5 – 0·9)	7·2 (4·9 – 10·0)	11·0 (6·9 – 16·6)	0·1 (0·1 – 0·2)	31·7 (23·5 – 40·1)	2·7 (1·9 – 3·9)	2·6 (2·1 – 3·1)	1·6 (1·1 – 2·4)	18·9 (13·9 – 24·6)	16·6 (11·8 – 21·9)	0·9 (0·7 – 1·3)
Indonesia	3·7 (2·3 – 5·6)	0·8 (0·5 – 1·2)	22·9 (17·3 – 29·6)	2·7 (1·6 – 4·3)	0·2 (0·1 – 0·3)	47·4 (39·2 – 55·2)	2·9 (2·0 – 3·9)	1·9 (1·5 – 2·3)	3·2 (2·3 – 4·5)	5·9 (4·2 – 8·2)	2·2 (1·5 – 3·1)	6·1 (4·5 – 8·2)
Laos	14·8 (9·9 – 21·3)	0·5 (0·3 – 0·7)	6·8 (4·5 – 9·6)	3·8 (2·2 – 6·0)	0·0 (0·0 – 0·0)	38·3 (30·6 – 47·4)	0·7 (0·5 – 1·0)	2·1 (1·7 – 2·6)	10·4 (7·1 – 14·9)	17·4 (12·9 – 22·8)	3·2 (2·2 – 4·5)	1·9 (1·3 – 2·7)
Malaysia	11·0 (8·0 – 14·6)	1·3 (0·9 – 1·9)	2·3 (1·5 – 3·3)	0·6 (0·3 – 0·9)	0·2 (0·1 – 0·4)	46·4 (40·0 – 52·9)	6·8 (5·1 – 8·9)	1·7 (1·5 – 2·1)	2·6 (1·8 – 3·5)	12·4 (9·5 – 15·8)	6·3 (4·6 – 8·5)	8·3 (6·4 – 11·0)
Maldives	17·0 (11·4 – 23·7)	1·2 (0·8 – 1·8)	2·4 (1·6 – 3·5)	0·6 (0·3 – 1·0)	0·0 (0·0 – 0·0)	17·9 (12·5 – 24·3)	3·4 (2·3 – 4·9)	1·9 (1·6 – 2·3)	4·8 (3·3 – 6·6)	13·4 (9·5 – 18·2)	8·2 (5·7 – 11·1)	29·3 (22·6 – 36·1)
Mauritius	8·0 (5·2 – 11·4)	0·3 (0·2 – 0·5)	3·3 (2·3 – 4·6)	2·2 (1·3 – 3·4)	0·0 (0·0 – 0·1)	24·9 (19·1 – 31·3)	7·8 (5·6 – 10·7)	1·5 (1·2 – 1·9)	1·0 (0·7 – 1·4)	17·7 (13·5 – 22·2)	21·7 (16·7 – 27·0)	11·5 (8·6 – 15·1)
Myanmar	19·4 (13·3 – 26·8)	7·3 (4·8 – 10·6)	6·0 (3·9 – 8·7)	9·0 (5·3 – 13·9)	0·0 (0·0 – 0·0)	28·2 (20·9 – 37·1)	0·5 (0·4 – 0·8)	3·5 (2·9 – 4·2)	4·0 (2·8 – 5·6)	12·5 (8·9 – 16·5)	3·1 (2·1 – 4·5)	6·6 (4·6 – 8·9)
Philippines	16·5 (11·5 – 22·5)	0·8 (0·5 – 1·1)	7·0 (4·8 – 9·6)	0·8 (0·5 – 1·3)	0·0 (0·0 – 0·1)	19·6 (14·0 – 25·4)	3·7 (2·7 – 5·4)	1·4 (1·2 – 1·7)	3·4 (2·3 – 4·9)	26·2 (20·4 – 32·4)	19·2 (14·8 – 25·1)	1·3 (1·0 – 1·8)
Seychelles	18·4 (12·9 – 24·6)	1·8 (1·2 – 2·6)	11·7 (8·4 – 15·9)	4·7 (2·8 – 7·5)	0·4 (0·2 – 0·6)	29·3 (22·5 – 37·0)	3·5 (2·5 – 4·8)	2·2 (1·9 – 2·6)	2·7 (1·9 – 3·8)	14·4 (10·5 – 19·1)	8·5 (6·0 – 11·7)	2·4 (1·8 – 3·2)
Sri Lanka	30·4 (24·3 – 36·8)	3·5 (2·4 – 4·8)	12·1 (9·0 – 15·7)	1·9 (1·1 – 3·3)	0·1 (0·0 – 0·1)	7·0 (4·7 – 10·1)	6·0 (4·3 – 8·1)	1·5 (1·3 – 1·8)	1·3 (0·9 – 1·8)	29·1 (24·0 – 35·0)	6·8 (4·8 – 9·2)	0·4 (0·3 – 0·6)
Thailand	26·2 (20·0 – 33·6)	6·3 (4·4 – 8·9)	12·3 (9·2 – 16·4)	3·7 (2·3 – 5·6)	0·0 (0·0 – 0·0)	38·3 (31·2 – 45·6)	1·3 (0·9 – 1·8)	1·8 (1·5 – 2·1)	3·8 (2·7 – 5·3)	3·0 (2·2 – 4·1)	2·3 (1·6 – 3·3)	0·9 (0·6 – 1·2)
Timor-Leste	4·6 (2·6 – 7·6)	2·0 (1·3 – 3·1)	7·7 (5·1 – 11·0)	15·6 (10·0 – 23·3)	0·4 (0·2 – 0·6)	9·7 (6·3 – 14·4)	0·5 (0·3 – 0·8)	11·7 (9·5 – 14·1)	22·0 (16·2 – 28·8)	13·4 (9·4 – 18·2)	3·4 (2·3 – 4·7)	9·1 (6·4 – 12·7)
Vietnam	5·9 (3·6 – 8·9)	0·6 (0·4 – 0·9)	46·5 (39·5 – 52·9)	0·1 (0·1 – 0·2)	0·0 (0·0 – 0·0)	7·8 (5·3 – 11·0)	3·4 (2·4 – 4·9)	1·5 (1·2 – 1·8)	3·0 (2·0 – 4·4)	21·1 (16·1 – 26·7)	9·4 (6·8 – 12·7)	0·6 (0·5 – 0·9)
**Sub-Saharan Africa**	**10·9 (9·3 – 12·7)**	**1·6 (1·4 – 1·9)**	**3·6 (3·3 – 4·0)**	**11·3 (9·5 – 13·4)**	**0·6 (0·5 – 0·7)**	**23·1 (21·1 – 25·1)**	**4·3 (3·8 – 4·9)**	**5·0 (4·8 – 5·3)**	**10·2 (9·4 – 11·1)**	**19·9 (18·6 – 21·3)**	**4·5 (4·0 – 5·0)**	**5·0 (4·6 – 5·4)**
Central sub-Saharan Africa	1·4 (1·0 – 1·8)	0·7 (0·5 – 0·8)	1·5 (1·2 – 2·0)	1·4 (1·0 – 1·9)	0·4 (0·3 – 0·6)	5·9 (4·8 – 7·3)	4·7 (3·7 – 6·0)	3·7 (3·3 – 4·1)	11·7 (9·6 – 14·4)	53·8 (49·5 – 57·7)	7·7 (5·6 – 10·3)	7·2 (5·9 – 8·8)
Angola	1·5 (1·1 – 2·0)	1·5 (1·1 – 1·9)	7·7 (5·6 – 10·4)	3·5 (2·4 – 5·1)	0·4 (0·3 – 0·7)	19·2 (14·6 – 24·1)	3·0 (2·1 – 4·4)	6·1 (5·2 – 7·1)	16·2 (13·2 – 19·9)	32·0 (26·9 – 37·7)	2·3 (1·8 – 3·0)	6·7 (5·2 – 8·6)
Central African Republic	2·2 (1·5 – 3·4)	0·8 (0·6 – 1·1)	0·9 (0·7 – 1·2)	1·2 (0·8 – 1·9)	0·4 (0·3 – 0·6)	12·1 (8·9 – 16·5)	4·5 (3·2 – 6·2)	3·8 (3·2 – 4·4)	19·7 (15·1 – 25·1)	48·2 (41·4 – 54·6)	2·3 (1·6 – 3·3)	3·9 (2·8 – 5·3)
Congo (Brazzaville)	0·5 (0·3 – 0·6)	0·3 (0·2 – 0·4)	0·4 (0·3 – 0·5)	0·2 (0·1 – 0·3)	0·1 (0·0 – 0·1)	3·4 (2·4 – 4·7)	4·6 (3·4 – 6·2)	2·4 (2·0 – 2·8)	6·7 (4·8 – 9·3)	68·6 (62·8 – 73·4)	5·4 (3·8 – 7·5)	7·6 (5·9 – 9·6)
DR Congo	1·3 (0·9 – 2·0)	0·6 (0·4 – 0·7)	0·8 (0·6 – 1·1)	1·2 (0·8 – 1·9)	0·4 (0·3 – 0·7)	3·4 (2·5 – 4·8)	5·1 (3·7 – 6·8)	3·4 (2·9 – 4·0)	10·5 (7·7 – 14·1)	56·2 (50·2 – 61·7)	9·5 (6·8 – 12·9)	7·6 (5·8 – 9·8)
Equatorial Guinea	5·1 (3·2 – 8·0)	1·3 (0·9 – 1·8)	3·8 (2·6 – 5·4)	0·7 (0·4 – 1·1)	0·0 (0·0 – 0·1)	7·7 (5·6 – 10·7)	1·1 (0·8 – 1·6)	9·7 (8·1 – 11·7)	20·9 (15·9 – 27·2)	32·0 (25·5 – 38·6)	3·5 (2·4 – 5·1)	14·1 (10·5 – 18·4)
Gabon	1·7 (1·0 – 2·7)	0·3 (0·2 – 0·4)	0·4 (0·3 – 0·5)	0·1 (0·1 – 0·1)	0·1 (0·0 – 0·1)	6·1 (4·4 – 8·3)	3·8 (2·8 – 5·4)	2·2 (1·8 – 2·7)	11·0 (8·3 – 14·4)	64·3 (59·0 – 69·4)	3·0 (2·1 – 4·2)	7·0 (5·5 – 8·7)
Eastern sub-Saharan Africa	8·8 (7·6 – 10·1)	1·4 (1·3 – 1·7)	4·0 (3·4 – 4·7)	9·9 (8·4 – 11·6)	0·6 (0·5 – 0·8)	20·1 (17·9 – 22·7)	3·6 (3·2 – 4·2)	5·5 (5·0 – 6·0)	13·3 (12·0 – 14·8)	21·5 (20·3 – 24·0)	5·3 (4·6 – 6·3)	5·4 (4·8 – 6·0)
Burundi	2·7 (1·7 – 4·2)	2·2 (1·6 – 3·1)	5·0 (3·4 – 7·2)	12·8 (8·3 – 18·9)	0·7 (0·5 – 1·1)	6·6 (4·5 – 9·4)	2·6 (1·9 – 3·7)	5·1 (4·1 – 6·0)	9·8 (6·9 – 13·9)	43·5 (36·1 – 51·6)	6·6 (4·6 – 9·3)	2·4 (1·8 – 3·2)
Comoros	6·0 (3·9 – 9·1)	0·9 (0·6 – 1·2)	1·9 (1·4 – 2·4)	3·2 (2·0 – 4·8)	0·1 (0·1 – 0·2)	13·2 (9·4 – 17·8)	1·6 (1·1 – 2·2)	4·4 (3·7 – 5·3)	25·2 (19·5 – 31·4)	25·8 (20·4 – 31·9)	11·5 (8·4 – 15·5)	6·2 (4·6 – 8·3)
Djibouti	3·0 (2·1 – 4·2)	2·3 (1·7 – 2·9)	3·3 (2·6 – 4·4)	6·5 (4·7 – 8·8)	0·6 (0·4 – 0·8)	45·2 (37·9 – 52·3)	2·9 (1·9 – 4·4)	8·2 (6·8 – 10·0)	16·7 (12·7 – 21·7)	7·0 (4·9 – 10·0)	1·6 (1·2 – 2·1)	2·6 (2·0 – 3·4)
Eritrea	2·6 (1·6 – 4·2)	1·4 (1·0 – 2·0)	4·1 (2·7 – 5·8)	2·0 (1·2 – 3·2)	0·2 (0·1 – 0·3)	11·5 (7·7 – 16·1)	1·7 (1·2 – 2·4)	6·1 (4·9 – 7·3)	53·2 (45·6 – 60·9)	12·8 (9·4 – 16·6)	1·6 (1·1 – 2·3)	2·9 (2·1 – 3·9)
Ethiopia	5·7 (3·6 – 8·9)	2·3 (1·7 – 3·3)	6·0 (4·1 – 8·9)	13·6 (9·0 – 19·8)	1·3 (0·8 – 2·0)	27·5 (20·3 – 36·1)	1·6 (1·1 – 2·2)	9·3 (7·9 – 11·0)	10·5 (7·7 – 14·0)	15·0 (11·1 – 19·5)	2·7 (2·0 – 3·7)	4·2 (3·0 – 5·8)
Kenya	11·4 (7·8 – 16·4)	1·0 (0·7 – 1·3)	9·6 (6·8 – 12·9)	5·2 (3·3 – 8·3)	0·2 (0·1 – 0·4)	22·0 (16·4 – 28·6)	1·4 (1·1 – 2·0)	5·6 (4·7 – 6·5)	11·5 (8·4 – 15·6)	25·8 (20·8 – 31·3)	1·5 (1·1 – 2·2)	4·5 (3·4 – 6·0)
Madagascar	4·2 (2·6 – 6·2)	0·9 (0·7 – 1·1)	2·0 (1·4 – 2·9)	9·4 (6·1 – 13·9)	0·5 (0·4 – 0·8)	11·4 (8·1 – 15·6)	1·9 (1·4 – 2·5)	2·9 (2·5 – 3·4)	9·1 (6·7 – 12·5)	51·5 (44·2 – 58·6)	4·3 (3·1 – 6·0)	1·9 (1·4 – 2·4)
Malawi	22·1 (15·6 – 29·5)	1·3 (1·0 – 1·7)	1·8 (1·3 – 2·7)	25·4 (18·2 – 33·4)	0·7 (0·5 – 1·1)	12·0 (8·3 – 17·3)	7·1 (5·0 – 9·8)	4·2 (3·5 – 5·0)	3·3 (2·4 – 4·5)	10·1 (7·2 – 13·6)	6·4 (4·4 – 9·0)	5·6 (4·0 – 7·6)
Mozambique	5·5 (3·6 – 8·2)	1·4 (1·1 – 1·9)	2·9 (2·2 – 3·8)	16·5 (11·7 – 22·7)	0·5 (0·3 – 0·7)	30·4 (24·3 – 37·3)	8·3 (5·9 – 11·3)	5·1 (4·4 – 6·0)	14·1 (10·5 – 18·7)	6·5 (5·0 – 8·5)	1·4 (1·0 – 1·9)	7·5 (5·7 – 10·0)
Rwanda	4·8 (3·0 – 7·6)	1·4 (1·0 – 2·0)	1·8 (1·2 – 2·6)	5·2 (3·3 – 7·8)	0·4 (0·3 – 0·7)	6·2 (4·3 – 9·2)	1·3 (0·9 – 1·8)	3·9 (3·2 – 4·7)	12·6 (8·9 – 17·4)	48·2 (40·4 – 56·2)	12·4 (8·8 – 16·9)	1·8 (1·3 – 2·5)
Somalia	2·2 (1·5 – 3·2)	2·2 (1·6 – 3·1)	2·1 (1·5 – 2·8)	3·0 (1·9 – 4·6)	2·0 (1·3 – 3·0)	5·7 (4·0 – 7·8)	1·8 (1·3 – 2·5)	14·0 (11·3 – 16·8)	58·3 (51·5 – 65·2)	4·8 (3·4 – 6·4)	2·1 (1·4 – 2·9)	1·8 (1·3 – 2·5)
South Sudan	3·3 (2·1 – 5·3)	3·2 (2·2 – 4·4)	2·2 (1·5 – 2·9)	3·5 (2·3 – 5·2)	0·4 (0·3 – 0·5)	4·8 (3·3 – 7·3)	4·6 (3·3 – 6·5)	9·0 (7·6 – 10·4)	26·7 (20·8 – 34·0)	34·2 (27·8 – 41·6)	3·0 (2·1 – 4·2)	5·2 (3·9 – 6·8)
Uganda	12·1 (8·3 – 17·0)	1·4 (1·0 – 1·8)	2·3 (1·7 – 3·2)	8·2 (5·5 – 11·7)	0·4 (0·3 – 0·6)	14·0 (10·6 – 18·7)	5·1 (3·6 – 6·8)	4·0 (3·5 – 4·6)	17·5 (13·4 – 22·9)	22·0 (18·0 – 27·1)	5·0 (3·6 – 6·9)	8·0 (5·9 – 10·5)
Tanzania	10·8 (7·4 – 15·4)	1·0 (0·7 – 1·3)	2·8 (2·1 – 3·8)	4·4 (2·8 – 6·7)	0·3 (0·2 – 0·4)	20·8 (15·8 – 26·7)	4·0 (2·9 – 5·5)	3·4 (2·9 – 4·0)	12·9 (9·6 – 17·2)	21·3 (17·2 – 25·9)	11·1 (7·9 – 15·7)	7·1 (5·4 – 9·4)
Zambia	8·3 (5·4 – 11·9)	1·0 (0·7 – 1·3)	2·1 (1·5 – 2·8)	1·6 (1·0 – 2·5)	0·3 (0·2 – 0·4)	30·1 (23·3 – 37·2)	5·6 (4·0 – 7·4)	3·7 (3·1 – 4·4)	15·4 (11·3 – 20·3)	12·5 (9·5 – 16·1)	9·7 (7·0 – 13·3)	9·7 (7·4 – 12·4)
Southern sub-Saharan Africa	21·2 (17·0 – 26·0)	2·1 (1·5 – 3·0)	3·3 (2·6 – 4·1)	21·3 (16·8 – 26·9)	0·5 (0·3 – 0·7)	36·2 (31·1 – 41·5)	4·5 (3·4 – 6·0)	2·7 (2·4 – 3·0)	2·0 (1·6 – 2·4)	1·9 (1·5 – 2·5)	2·2 (1·7 – 2·8)	2·1 (1·8 – 2·5)
Botswana	9·4 (6·3 – 13·7)	0·9 (0·6 – 1·3)	17·6 (13·5 – 22·2)	1·9 (1·2 – 3·0)	0·1 (0·1 – 0·2)	40·6 (33·2 – 47·9)	6·2 (4·4 – 8·6)	3·5 (2·9 – 4·1)	5·5 (4·0 – 7·4)	11·0 (8·3 – 14·2)	1·2 (0·8 – 1·7)	2·0 (1·4 – 2·7)
Eswatini	18·1 (13·1 – 24·1)	1·6 (1·1 – 2·1)	6·9 (5·0 – 9·5)	6·1 (4·1 – 8·8)	0·2 (0·1 – 0·3)	27·4 (21·2 – 34·6)	3·4 (2·5 – 4·9)	6·2 (5·3 – 7·2)	8·9 (6·9 – 11·4)	7·1 (5·3 – 9·4)	5·2 (3·7 – 7·2)	8·9 (6·9 – 11·4)
Lesotho	9·5 (6·6 – 13·5)	1·7 (1·2 – 2·3)	7·3 (5·4 – 9·4)	6·5 (4·7 – 9·1)	0·3 (0·2 – 0·4)	35·1 (28·5 – 42·2)	6·6 (4·9 – 8·8)	6·5 (5·5 – 7·6)	4·5 (3·4 – 5·9)	4·0 (3·0 – 5·1)	11·0 (7·7 – 15·3)	7·1 (5·3 – 9·6)
Namibia	20·9 (16·1 – 26·4)	1·3 (1·0 – 1·7)	5·2 (4·0 – 6·6)	13·3 (10·2 – 16·9)	0·3 (0·2 – 0·4)	30·8 (25·1 – 36·6)	3·6 (2·6 – 5·1)	9·3 (7·7 – 11·1)	3·1 (2·4 – 4·0)	4·1 (3·1 – 5·6)	0·9 (0·7 – 1·2)	7·1 (5·5 – 9·0)
South Africa	23·9 (18·9 – 29·5)	2·4 (1·6 – 3·4)	3·2 (2·5 – 4·2)	25·1 (19·8 – 31·6)	0·5 (0·4 – 0·8)	31·1 (25·6 – 37·3)	4·8 (3·6 – 6·7)	2·5 (2·1 – 2·9)	1·5 (1·2 – 1·9)	1·8 (1·3 – 2·5)	1·5 (1·1 – 2·1)	1·6 (1·2 – 2·0)

Zimbabwe	5·3 (3·4 – 7·7)	0·7 (0·5 – 0·9)	1·7 (1·2 – 2·5)	1·1 (0·7 – 1·8)	0·1 (0·1 – 0·2)	70·6 (64·6 – 75·9)	1·9 (1·3 – 2·6)	2·3 (1·9 – 2·9)	4·2 (3·0 – 5·9)	1·6 (1·1 – 2·3)	6·2 (4·1 – 8·9)	4·2 (3·1 – 5·6)
Western sub-Saharan Africa	3·5 (3·0 – 4·1)	1·6 (1·4 – 1·8)	4·6 (3·9 – 5·5)	3·3 (2·8 – 4·0)	0·7 (0·6 – 0·9)	17·0 (15·0 – 18·9)	4·8 (4·1 – 5·7)	8·7 (8·2 – 9·4)	17·4 (15·5 – 19·6)	26·1 (24·0 – 28·4)	4·8 (4·1 – 5·7)	7·5 (6·6 – 8·5)
Benin	2·5 (1·6 – 3·9)	1·1 (0·8 – 1·5)	2·6 (1·8 – 3·7)	2·8 (1·7 – 4·4)	0·5 (0·4 – 0·8)	5·3 (3·6 – 7·6)	3·4 (2·4 – 5·0)	4·4 (3·7 – 5·2)	6·9 (4·7 – 9·8)	42·5 (35·6 – 49·7)	22·3 (16·5 – 29·1)	5·7 (4·2 – 7·5)
Burkina Faso	2·3 (1·5 – 3·5)	1·6 (1·1 – 2·2)	4·8 (3·2 – 6·8)	5·7 (3·5 – 9·1)	2·4 (1·5 – 3·7)	23·3 (17·2 – 30·8)	7·9 (5·8 – 10·4)	6·1 (5·1 – 7·2)	8·3 (6·1 – 11·1)	32·9 (26·6 – 39·4)	1·7 (1·2 – 2·3)	3·0 (2·2 – 4·1)
Cape Verde	25·8 (19·7 – 32·5)	0·5 (0·4 – 0·6)	6·5 (4·9 – 9·0)	3·2 (2·2 – 4·7)	0·1 (0·0 – 0·1)	36·4 (30·2 – 43·1)	4·4 (3·2 – 6·0)	2·7 (2·2 – 3·2)	1·9 (1·3 – 2·7)	13·4 (10·2 – 17·1)	2·4 (1·8 – 3·3)	2·7 (2·1 – 3·5)
Cameroon	3·5 (2·2 – 5·6)	0·7 (0·5 – 0·9)	1·2 (0·9 – 1·7)	0·8 (0·5 – 1·2)	0·1 (0·1 – 0·2)	4·2 (3·0 – 5·7)	2·9 (2·2 – 4·0)	4·7 (3·9 – 5·6)	18·5 (13·3 – 24·4)	54·0 (46·6 – 61·2)	4·0 (2·8 – 5·8)	5·3 (3·9 – 6·9)
Chad	3·6 (2·3 – 5·6)	1·5 (1·0 – 2·1)	1·5 (1·0 – 2·1)	2·8 (1·8 – 4·3)	0·6 (0·4 – 0·8)	5·1 (3·6 – 7·3)	2·2 (1·5 – 3·2)	6·1 (5·0 – 7·3)	55·0 (47·6 – 62·1)	16·9 (12·8 – 21·6)	1·6 (1·1 – 2·3)	3·2 (2·3 – 4·4)
Côte d'Ivoire	1·2 (0·8 – 1·7)	0·9 (0·7 – 1·2)	1·1 (0·8 – 1·6)	1·1 (0·7 – 1·8)	0·2 (0·1 – 0·3)	14·8 (11·1 – 19·5)	4·4 (3·0 – 6·3)	6·3 (5·3 – 7·4)	9·9 (7·0 – 13·7)	51·3 (44·7 – 58·0)	1·0 (0·7 – 1·3)	7·8 (6·0 – 10·5)
The Gambia	5·4 (3·5 – 8·1)	15·6 (11·2 – 21·0)	13·0 (9·2 – 17·9)	2·6 (1·6 – 4·1)	0·3 (0·2 – 0·4)	18·5 (13·3 – 25·3)	1·9 (1·4 – 2·6)	8·8 (7·5 – 10·2)	8·7 (6·5 – 11·9)	13·3 (10·2 – 17·1)	2·5 (1·7 – 3·6)	9·4 (6·8 – 12·4)
Ghana	4·6 (2·9 – 7·1)	1·1 (0·8 – 1·5)	2·9 (2·1 – 3·9)	2·1 (1·3 – 3·4)	0·3 (0·2 – 0·4)	19·6 (14·6 – 26·0)	4·1 (3·0 – 5·6)	14·5 (12·4 – 16·8)	9·8 (7·0 – 13·6)	33·0 (26·9 – 39·1)	4·5 (3·3 – 6·1)	3·5 (2·6 – 4·6)
Guinea	2·4 (1·6 – 3·6)	1·7 (1·3 – 2·2)	2·2 (1·6 – 2·9)	3·9 (2·7 – 5·6)	0·5 (0·3 – 0·7)	11·5 (8·5 – 14·9)	3·1 (2·1 – 4·4)	8·0 (6·5 – 9·7)	43·9 (36·1 – 52·0)	15·1 (11·6 – 19·2)	2·1 (1·5 – 2·9)	5·7 (4·2 – 7·2)
Guinea-Bissau	1·9 (1·3 – 2·7)	0·9 (0·6 – 1·2)	34·3 (28·2 – 40·5)	1·5 (1·1 – 2·2)	0·8 (0·5 – 1·2)	5·8 (4·3 – 7·5)	5·7 (3·7 – 8·5)	5·2 (4·6 – 6·1)	22·8 (17·5 – 29·0)	15·7 (12·2 – 19·7)	1·1 (0·8 – 1·5)	4·4 (3·3 – 5·7)
Liberia	9·5 (6·5 – 13·8)	1·8 (1·3 – 2·4)	5·7 (4·2 – 7·5)	5·2 (3·5 – 7·5)	1·9 (1·3 – 2·7)	41·4 (34·6 – 48·7)	2·9 (2·0 – 4·2)	8·2 (7·0 – 9·6)	4·1 (3·0 – 5·4)	13·0 (9·9 – 16·3)	2·2 (1·6 – 2·9)	4·2 (3·2 – 5·4)
Mali	3·5 (2·3 – 5·4)	1·7 (1·2 – 2·3)	2·9 (2·0 – 4·2)	4·8 (3·1 – 7·3)	1·0 (0·7 – 1·5)	29·8 (23·3 – 36·8)	2·6 (1·8 – 3·6)	7·0 (6·0 – 8·3)	18·8 (14·3 – 24·4)	16·4 (12·7 – 21·2)	1·9 (1·4 – 2·7)	9·6 (7·3 – 12·5)
Mauritania	4·2 (2·6 – 6·5)	2·3 (1·6 – 3·4)	7·1 (4·8 – 10·4)	2·1 (1·3 – 3·4)	0·2 (0·1 – 0·3)	21·6 (15·6 – 28·9)	2·2 (1·6 – 3·1)	11·1 (9·4 – 13·0)	14·0 (10·3 – 19·3)	8·1 (5·9 – 10·8)	22·2 (16·3 – 29·5)	4·8 (3·5 – 6·6)
Niger	2·0 (1·2 – 3·1)	1·1 (0·8 – 1·6)	1·9 (1·3 – 2·8)	6·1 (3·8 – 9·7)	1·1 (0·7 – 1·8)	30·5 (23·2 – 39·1)	1·2 (0·8 – 1·7)	4·4 (3·7 – 5·4)	27·6 (21·0 – 34·7)	2·7 (1·9 – 3·8)	1·1 (0·8 – 1·5)	20·3 (14·9 – 26·8)
Nigeria	3·1 (2·1 – 4·5)	2·0 (1·5 – 2·5)	5·8 (4·2 – 8·1)	3·0 (1·9 – 4·6)	0·4 (0·3 – 0·6)	13·1 (9·9 – 17·1)	6·4 (4·8 – 8·7)	10·9 (9·7 – 12·4)	19·9 (14·9 – 25·5)	19·9 (15·8 – 25·2)	6·8 (5·2 – 8·9)	8·7 (6·8 – 11·0)
São Tomé and Príncipe	2·8 (2·0 – 3·8)	1·6 (1·1 – 2·3)	4·0 (2·9 – 5·4)	8·9 (6·2 – 13·1)	0·3 (0·2 – 0·4)	49·4 (42·4 – 56·0)	4·5 (2·9 – 6·9)	5·6 (4·7 – 6·7)	5·7 (4·3 – 7·4)	9·8 (7·6 – 12·6)	1·3 (0·9 – 1·7)	6·1 (4·6 – 7·9)
Senegal	4·2 (2·7 – 6·5)	1·9 (1·3 – 2·5)	10·1 (7·2 – 13·7)	2·6 (1·7 – 3·8)	0·5 (0·3 – 0·8)	23·8 (18·1 – 30·8)	3·0 (2·2 – 4·1)	7·6 (6·3 – 8·9)	7·8 (5·6 – 10·4)	14·9 (11·4 – 19·0)	2·0 (1·4 – 2·8)	21·7 (16·8 – 27·9)
Sierra Leone	3·5 (2·3 – 5·1)	2·0 (1·5 – 2·8)	5·0 (3·7 – 6·7)	10·0 (7·2 – 13·5)	1·7 (1·2 – 2·4)	38·6 (31·9 – 45·8)	2·5 (1·8 – 3·4)	7·6 (6·5 – 8·8)	13·5 (9·9 – 17·9)	5·3 (4·0 – 7·1)	1·9 (1·5 – 2·6)	8·4 (6·4 – 11·4)
Togo	3·2 (2·1 – 4·6)	1·1 (0·8 – 1·5)	2·8 (1·9 – 4·0)	1·7 (1·1 – 2·6)	0·5 (0·3 – 0·7)	5·5 (3·9 – 7·9)	4·2 (3·1 – 5·8)	6·7 (5·6 – 8·0)	14·1 (10·2 – 18·8)	52·9 (46·2 – 59·4)	4·4 (3·1 – 6·2)	2·9 (2·1 – 3·8)

Supplementary Table 18. Contraceptive methods in use as a share of contraceptive prevalence, 1970

Notes: Diaphragm, emergency contraception, and other modern methods that were estimated separately were combined into a single column (other modern method) since these prevalence estimates tend to be small (prevalence <5%)

References

1. United Nations Population Division. SDG Indicator 3.7.1 on Contraceptive Use. United Nations.
2. Bradley SEK, Croft TN, Fishel JD, Westoff CF. Revising unmet need for family planning. 2012; published online Jan 1. https://dhsprogram.com/publications/publication-as25-analytical-studies.cfm (Last accessed 2022 Mar 14).
3. Demographic and Health Surveys. Demographic and Health Surveys Phase 7 Model Woman's Questionnaire. 2018; published online Dec 17. https://dhsprogram.com/pubs/pdf/DHSQ7/DHS7-Womans-QRE-EN-17Dec2018-DHSQ7.pdf (Last accessed 2022 Mar 14).
4. Demographic and Health Surveys. Demographic and Health Surveys Phase 6 Model Woman's Questionnaire. 2012; published online Nov 5. https://dhsprogram.com/pubs/pdf/DHSQ6/DHS6_Questionnaires_5Nov2012_DHSQ6.pdf (Last accessed 2022 Mar 14).
5. Center for Disease Control and Prevention. Contraception Resources from the CDC: 2016 U.S. Medical Eligibility Criteria for Contraceptive Use. 2016. https://www.cdc.gov/reproductivehealth/contraception/unintendedpregnancy/pdf/MEC_SlideSet_2016.pdf (Last accessed 2022 Mar 14).
6. GBD 2019 Risk Factors Collaborators. Global burden of 87 risk factors in 204 countries and territories, 1990–2019: a systematic analysis for the Global Burden of Disease Study 2019. The Lancet 2020; 396: 1223–49. doi: https://doi.org/10.1016/S0140-6736(20)30752-2
7. GBD 2019 Demographics Collaborators. Global age-sex-specific fertility, mortality, healthy life expectancy (HALE), and population estimates in 204 countries and territories, 1950–2019: a comprehensive demographic analysis for the Global Burden of Disease Study 2019. The Lancet 2020; 396: 1160–203. doi: https://doi.org/10.1016/S0140-6736(20)30977-6
8. Murray CJL, Ezzati M, Flaxman AD, *et al*. GBD 2010: design, definitions, and metrics. *Lancet* 2012; 380: 2063-6. doi: https://doi.org/10.1016/S0140-6736(12)61899-6
9. London Summit on Family Planning, July 2020. Technical Note: Data sources and methodology for developing the 2012 baseline, 2020 objective, impacts and costings. 2012. https://fp2030.org/sites/default/files/2013_01-04_FP_Summit_technical_note_15_June.pdf (Last accessed 2022 Mar 14).
10. Zheng P, Barber R, Sorensen RJ, Murray CJ, Aravkin AY. Trimmed constrained mixed effects models: formulations and algorithms. Journal of Computational and Graphical Statistics. 2021; 1-13. https://www.tandfonline.com/doi/full/10.1080/10618600.2020.1868303
11. Cahill N, Sonneveldt E, Stover J, Weinberger M, Williamson J, Wei C, et al. Modern Contraceptive Use, Unmet Need, and Demand Satisfied Among Women of Reproductive Age Who Are Married or In A Union in the Focus Countries of the Family Planning 2020 Initiative: A Systematic Analysis Using the Family Planning Estimation Tool. The Lancet. 2017; 391(10123): 870–882.
12. Wheldon MC, Kantorová V, Ueffing P, Dasgupta ANZ. Methods for estimating and projecting key family planning indicators among all women of reproductive age. New York (NY): United Nations, Department of Economic and Social Affairs, Population Division; 2018. Technical Paper No. 2. https://www.un.org/en/development/desa/population/publications/pdf/technical/TP2018-2.pdf (Last accessed 2022 Mar 14).
13. Alkema L, Kantorová V, Menozzi C, Biddlecom A. "National, regional, and global rates and trends in contraceptive prevalence and unmet need for family planning between 1990 and 2015: a systematic and comprehensive analysis," Lancet. 2013; 381: 1642–1652. pmid:23489750
